# Lyn and Fyn function as molecular switches that control immunoreceptors to direct homeostasis or inflammation

**DOI:** 10.1038/s41467-017-00294-0

**Published:** 2017-08-15

**Authors:** Sanae Ben Mkaddem, Amaya Murua, Héloise Flament, Dimitri Titeca-Beauport, Carine Bounaix, Luca Danelli, Pierre Launay, Marc Benhamou, Ulrich Blank, Eric Daugas, Nicolas Charles, Renato C. Monteiro

**Affiliations:** 1 INSERM U1149, Centre de Recherche sur l’Inflammation, Paris, France; 2 CNRS ERL8252, Paris, France; 3 Université Paris Diderot, Sorbonne Paris Cité, Faculté de Médecine, Site Xavier Bichat, Paris, France; 4 Inflamex Laboratory of Excellence, Paris, France; 5 Service d’Immunologie, DHU Fire, Hôpital Bichat-Claude Bernard, Assistance Publique de Paris, Paris, France; 6 Service de Néphrologie, DHU Fire, Hôpital Bichat-Claude Bernard, Assistance Publique-Hôpitaux de Paris, Paris, France

## Abstract

Immunoreceptors can transduce either inhibitory or activatory signals depending on ligand avidity and phosphorylation status, which is modulated by the protein kinases Lyn and Fyn. Here we show that Lyn and Fyn control immune receptor signaling status. SHP-1 tyrosine 536 phosphorylation by Lyn activates the phosphatase promoting inhibitory signaling through the immunoreceptor. By contrast, Fyn-dependent phosphorylation of SHP-1 serine 591 inactivates the phosphatase, enabling activatory immunoreceptor signaling. These SHP-1 signatures are relevant in vivo, as Lyn deficiency exacerbates nephritis and arthritis in mice, whereas Fyn deficiency is protective. Similarly, Fyn-activating signature is detected in patients with lupus nephritis, underlining the importance of this Lyn–Fyn balance. These data show how receptors discriminate negative from positive signals that respectively result in homeostatic or inflammatory conditions.

## Abstract (web summary)

Src-family kinases Fyn and Lyn are signaling components downstream of ITAM-bearing antigen receptors. Here the authors show that by phosphorylating SHP-1 at different residues, Lyn and Fyn can have opposing regulatory effects on ITAM receptors.

## Introduction

The immune system is controlled by a finely tuned network of regulatory mechanisms that maintain homeostasis or can initiate inflammatory responses^1^. An important axis of regulation comprises immunoreceptor tyrosine-based activation motif (ITAM)-containing immunoreceptors, such as the T-cell receptors (TCR) and B-cell receptors (BCR), Fc receptors (FcR)^2^. ITAMs are defined by two consecutive Yxx[L/I] sequences separated by 6 to 12 amino acids, and are present in the cytoplasmic domains of several transmembrane adapter molecules, such as the common γ subunit of FcR (FcRγ), the Igα and Igβ subunits of the BCR, the γ, δ, ε, and ζ subunits of the TCR-associated CD3 complex^1, 3^, and in FcγRIIA^4^. Cellular responses after FcR triggering depend on ligand avidity. Receptor clustering mediated by high avidity ligand interaction induces phosphorylation on ITAM tyrosine residues by membrane-anchored and receptor-associated Src-family kinases (SFK). Phosphorylated ITAMs are docking sites for recruitment of the tyrosine kinases Syk or Zap70, which launch inflammatory responses and restore homeostasis. However, in case of dysregulation or chronic stimulation, ITAM signal can also result in autoimmune and inflammatory diseases^1, 5^. Both in innate and adaptive immunity, the activation of ITAMs-bearing immune receptors is actively counteracted by the action of ITIM-bearing inhibitory receptors such as FcγRIIB with the ITIM being defined by a single [I/V/L/S]xYxx[L/V] sequence. This regulation generally involves co-aggregation of inhibitory and targeted activated receptors and is promoted through recruitment of relevant phosphatases such as Src homology region 2 domain-containing inositol 5′ phosphatases (SHIP-1 and SHIP-2)^6^. In addition to this inhibitory feedback targeting co-aggregated activated receptors, a continuously active inhibitory mechanism generated by ITAM-bearing receptors following low avidity interactions has been described that acts towards a whole array of activating receptors without the requirement for co-aggregation^7–12^. This mechanism has been named inhibitory ITAM (ITAMi) and is thought to be involved in the maintenance of homeostasis^5, 13^. Various FcRs, such as FcαRI, FcγRIIA, and FcγRIIIA can function as such bi-functional receptors to trigger inhibitory signals, a property that can be exploited to reduce the susceptibility to autoimmune and inflammatory diseases^7–11^.

Induction of FcR ITAMi signals by weakly binding ligands with low avidity depends on the recruitment of the Src homology region 2 domain-containing tyrosine phosphatase SHP-1^13, 14^. BCRs and TCRs have been also reported to recruit SHP-1 upon interaction with low avidity ligands^15, 16^. Moreover, deletion of SHP-1 in haematopoietic lineages, including T cells, neutrophils, and dendritic cells, is associated with a variety of pathologies^17–20^. Together, these evidences support an important role of SHP-1 in the maintenance of immune homeostasis.

SFKs, such as Lyn and Fyn, are implicated in the initiation of ITAM-receptor-mediated signaling. These kinases are responsible for ITAM phosphorylation upon receptor aggregation leading to Syk recruitment initiating further signal propagation via downstream effectors such as PI3-kinase and phospholipase C-γ^13, 21^. In B cells, Lyn was reported to have both positive and negative roles in BCR-mediated signaling^22^. Aged Lyn-deficient mice have high levels of serum immunoglobulins (including autoantibodies) and their B cells are hyper-responsive to IL-4 and CD40 engagement^23–25^ demonstrating a defective homeostasis due to a deficient negative regulation. Therefore, the individual function and coordination of Lyn and Fyn in the control of ITAM signaling pathways mediated by the engagement of FcRs or BCRs by either high or low avidity ligands is unclear.

Here we report that Lyn and Fyn have important and non-redundant roles in the ligand-sensing threshold between ITAM and ITAMi signals mediated by immunoreceptors, able to differentially translate outside ligand interactions into opposite signals. Our results show that, although Lyn is crucial in coupling to ITAMi signals, Fyn is the essential effector molecule coupling to ITAM signals following FcR, but also BCR, engagement. This ITAM switch involves a capacity to differentially control SHP-1 by shifting its phosphorylation status. These signaling signatures are confirmed in inflammatory and auto-immune diseases involving an imbalance between ITAM and ITAMi signals.

## Results

### Lyn and Fyn differentially regulate FcR-ITAM signals

**Fig. 1:**
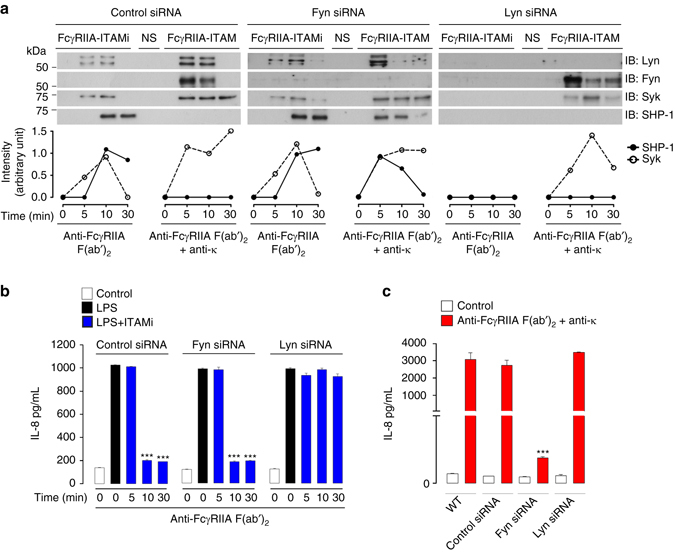
Differential regulation of FcR-ITAM signals by Lyn and Fyn. **a** After induction of FcγRIIA-ITAMi or ITAM signalling in THP-1-CD14^+^-FcγRIIA^+^ cells transfected with indicated siRNAs, immunoprecipitation (IP) and immunoblots (IB) were performed with indicated Abs. Quantification of the indicated band using ImageJ software relative to total corresponding protein levels in cell lysates (see Supplementary Fig. 1) is indicated at the *bottom* of each panel, representing one out of at least three experiments. **b** Modulation of LPS-mediated IL-8 production by Lyn and Fyn during FcγRIIA-ITAMi induction. THP-1-CD14^+^-FcγRIIA^+^ cells transfected with indicated siRNAs were stimulated for indicated time points to induce either ITAMi or ITAM signals followed by stimulation with LPS (10 ng/ml) for 1 h. Then, supernatant was collected for cytokine measurement. **c** Modulation of IL-8 production by Lyn and Fyn during FcγRIIA-ITAM induction for 18 h. Data are presented as the mean ± s.e.m. ***P < .001; Student’s unpaired *t-*test

In order to address whether SFKs play a role in the switch between ITAMi or ITAM signaling, we first downregulated Fyn or Lyn expression in representative human monocytic cell lines by a siRNA strategy. The cells were then stimulated for ITAMi signaling by divalent targeting, or for ITAM signals by multivalent crosslinking of FcγRIIA or FcαRI as described previously^7, 11^. The ITAMi molecular signature was characterized by transient Syk recruitment followed by stable and prolonged SHP-1 recruitment. This required the presence of Lyn but not Fyn. In contrast, multivalent crosslinking of these receptors inducing an ITAM activation signal molecular signature with a stable recruitment of Syk (but not SHP1), required the recruitment of Fyn (Fig. 1a and, Supplementary Figs. 1a and 2a, left panel). Fyn silencing had no effect on ITAMi signaling but reversed the ITAM to an ITAMi signature by the recruitment of SHP-1 to the receptors despite their multivalent crosslinking (Fig. 1a and, Supplementary Figs. 1 and 2a, middle panel). Lyn silencing completely abolished the ITAMi signaling without affecting the ITAM activation signal (Fig. 1a and, Supplementary Figs. 1 and 2a, right panel). Syk silencing did not alter the recruitment of Lyn or Fyn to the FcγRIIA (Supplementary Fig. 3a) but affected SHP-1 recruitment under ITAMi conditions as previously shown^11^. Functional consequences of individual SFK silencing were then evaluated. Lyn but not Fyn was essential for the ITAMi-dependent FcR-mediated inhibition of LPS-induced IL-8 production (Fig. 1b and Supplementary Fig. 2b). By contrast, Fyn but not Lyn was essential for ITAM-dependent cell activation as measured by IL-8 production after multivalent engagement of FcγRIIA (Fig. 1c) or FcαRI (Supplementary Fig. 2c). To address whether other SFK could compensate for ITAM signals, we first silenced both Lyn and Fyn expression in transfected monocytic cell line THP-1-CD14^+^-FcγRIIA^+^. This abolished both the ITAMi-signal-inhibiting heterologous TLR4 receptor and the ITAM-mediated IL8 production following crosslinking of FcγRIIA (Supplementary Fig. 4a–c). This was confirmed by Western blot analysis of tyrosine phosphorylated proteins in THP-1 whole cell lysates after ITAMi or ITAM induction (Supplementary Fig. 4g, h) ruling out a compensation by other SFK of Lyn/Fyn absence. In agreement, silencing expression of Hck, Fgr, or both, had no significant effect on ITAMi and ITAM signaling (Supplementary Fig. 4d–h). These results reveal opposing roles of the SFK Fyn and Lyn, transducing, respectively, activating or inhibitory signals depending on the type of ligand interaction.

### Distinct SFKs differentially regulate BCR-ITAM signals

**Fig. 2:**
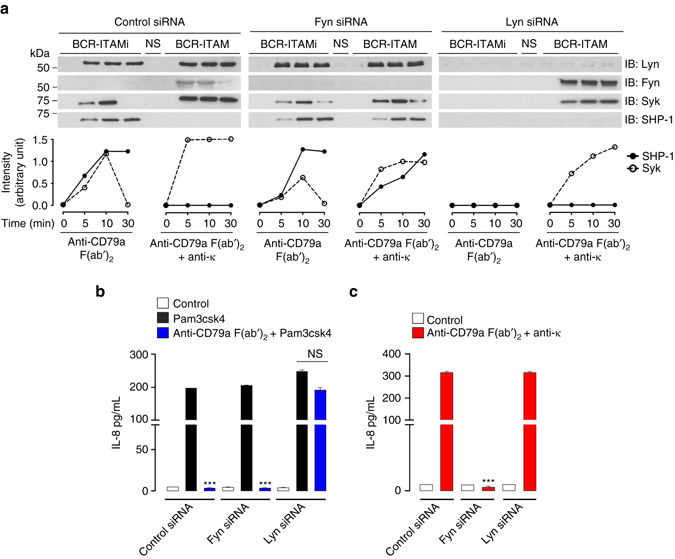
Differential regulation of BCR-ITAM signals by Lyn and Fyn. **a** After induction of BCR-ITAMi or ITAM signalling in transfected Ramos B cells, immunoprecipitation (IP) and immunoblots (IB) were performed with indicated Abs. Quantification of the indicated band using ImageJ software relative to total corresponding protein levels in cell lysates is shown at the *bottom* of each panel, representing one out of at least three experiments. **b** Modulation of Pam3csk4-mediated IL-8 production by Lyn and Fyn during BCR-ITAMi signalling on transfected Ramos B cells were stimulated for 30 min to induce ITAMi signal followed by stimulation with Pam3csk4 (1 µg/ml) for 6 h. Supernatants were collected for cytokine measurement. **c** Modulation of IL-8 production by Lyn and Fyn on transfected Ramos B cells after induction of BCR-ITAM signaling for 6 h. Supernatants were collected for cytokine measurement. For all, data are presented as the mean ± s.e.m. *n* = 3. ***P < .001; Student’s unpaired *t*-test. NS, not stimulated

To investigate whether other Lyn/Fyn-associated ITAM-bearing receptors could also deliver such opposite signals, divalent or multivalent targeting of BCR were performed using anti-CD79a F(ab′)_2_ fragments alone or complexed with anti-κ light chain antibodies in representative lymphocytic cell lines expressing siRNA for Lyn or Fyn. Similar to FcRs, divalent targeting of BCR resulted in typical ITAMi molecular signatures, while multivalent crosslinking led to the expected molecular signature of ITAM activation signal (Fig. 2a and Supplementary Fig. 1b). Furthermore, in agreement with the results obtained with FcRs, BCR-mediated ITAMi signals required Lyn, whereas Fyn was essential for ITAM activation signals. Since Syk is recruited with SHP-1 during the ITAMi signaling, we knocked-down Syk expression by using siRNA to determine its role in SHP-1 recruitment to the BCR. The silencing of Syk expression impaired the recruitment of SHP-1, but not of Lyn, to the BCR induced by anti-CD79a F(ab′)_2_ fragments. However, Syk silencing had no effect on Fyn recruitment under BCR-ITAM induction (Supplementary Fig. 3b). Altogether these findings suggest that BCR-ITAMi inhibitory signaling pathway sequentially involves Syk and SHP-1, as previously described for ITAMi signaling of FcRγ-associated Fc receptors^7, 11^. We next assessed whether BCR could also induce ITAMi functional signals following low avidity engagement. Thus, we examined whether they could inhibit functional responses induced by heterologous receptors such as TLR-induced cytokine production. The results clearly show that BCR-divalent targeting inhibited cytokine production induced by heterologous receptors, and this required Lyn (Fig. 2b). By contrast, Fyn was required for autologous cytokine production after BCR multivalent engagement (Fig. 2c). These results demonstrate that, similar to FcRs, ITAMi signaling by BCR is able to induce an inhibitory crosstalk with other receptors thereby dampening inflammatory responses without co-ligation between heterologous receptors. They explain previous observations concerning the triggering of negative signals by the binding of low avidity ligand or antibodies to the BCR^26^.

### SFKs differentially control SHP-1 phosphorylation

**Fig. 3:**
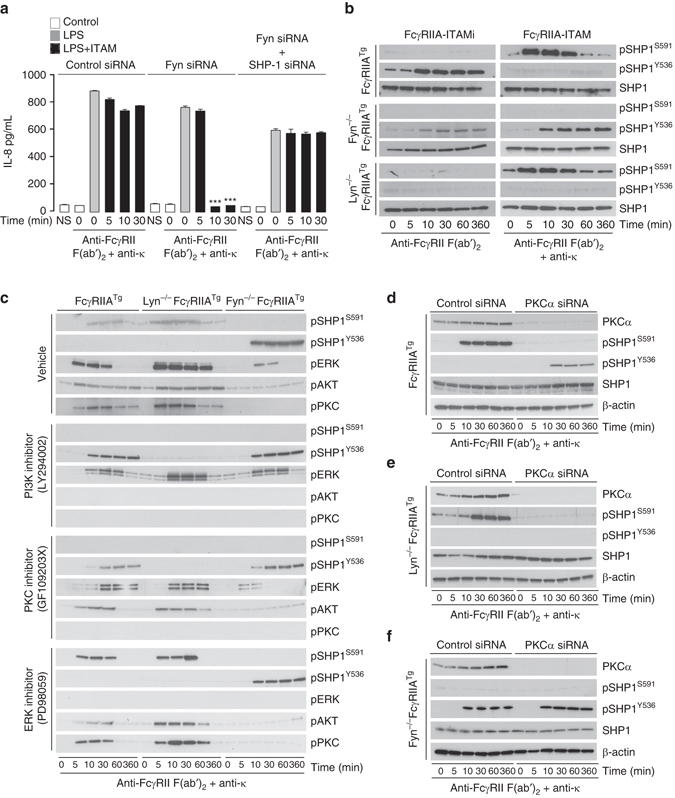
Fyn–PI3K–PKCα axis inactivates SHP-1-mediated ITAMi signaling. **a** Modulation of LPS-mediated IL-8 production by Lyn or Fyn after induction of FcγRIIA-ITAM signal on transfected THP-1. Cells were stimulated with LPS for 1 h at 37 °C after induction of ITAM as described in Fig. 1a. Data are presented as the mean ± s.e.m. ***P < .001; Student’s unpaired *t*-test. **b** After induction of FcγRIIA-mediated ITAMi or ITAM signals on BMDM derived from FcγRIIA transgenic mice or from FcγRIIA^Tg^ under Lyn-deficient or Fyn-deficient backgrounds. Cell lysate samples were subjected to SDS-PAGE and immunoblots were performed using anti-phospho (p) SHP-1 serine 591 (S591) or tyrosine 536 (Y536) Abs. **c** Effect of PI3K, PKC, and ERK inhibitors on SHP-1 phosphorylation driven by ITAM signals in BMDMs. Cells were pre-treated with inhibitors as indicated and Western blotting was performed on cell lysates using antibodies anti-phosphorylated (p) kinases and phosphatases as indicated. Total protein contents in cell lysates were shown in Supplementary Fig. 3,b. **d**–**f** Involvement of PKCα on serine SHP-1 phosphorylation following FcγRIIA-ITAM signal. BMDMs from FcγRIIA^Tg^ (**d**), Lyn-deficient FcγRIIA^Tg^ (**e**), or Fyn-deficient FcγRIIA^Tg^ (**f**) mice were transfected with indicated siRNAs before induction of FcγRIIA-ITAM as described in Fig. 1a. **b**–**f** Are representative of three experiments. NS, not stimulated

To address the mechanism by which Lyn regulate ITAMi signaling, we took advantage of our findings that Fyn deletion reverses ITAM activation signals into ITAMi signals to explore a possible link between SHP-1 and Fyn under ITAM-activating configuration. Although Fyn silencing resulted in an inhibitory signal generated by multivalent targeting of FcγRIIA, the silencing of both SHP-1 and Fyn abolished it (Fig. 3a and Supplementary Fig. 5a), indicating that Fyn abrogates an SHP-1-mediated inhibitory signal. Previously, phosphorylation of SHP-1 on Y536 and S591 residues has been associated with its activation and inactivation, respectively^27, 28^. Hence we analyzed whether Lyn and Fyn could control SHP-1 function through differential phosphorylation of this phosphatase. Stimulation of bone marrow-derived macrophages (BMDM) from FcγRIIA^Tg^ (R131 isoform) mice^29^ under ITAMi conditions showed that Lyn induced SHP-1 Y536 phosphorylation. By contrast, receptor multivalent aggregation induced a Fyn-dependent SHP-1 S591 phosphorylation (Fig. 3b). Under these stimulation conditions if Fyn was absent, a Lyn-dependent Y536 phosphorylation of SHP-1 was observed instead, thus mimicking an ITAMi signal (Fig. 3b). To understand how Fyn, a tyrosine kinase, could promote SHP-1 serine phosphorylation, we performed FcγRIIA-ITAM multivalent aggregation in the presence or absence of ERK, PKC, and PI3K inhibitors^30, 31^. Whereas PI3K and PKC inhibitors completely blocked both SHP-1^S591^ and PKC phosphorylation, the ERK inhibitor had no effect (Fig. 3c and Supplementary Fig. 5b). In addition, both PI3K and PKC inhibitors favored SHP-1^Y536^ phosphorylation under conditions of FcγRIIA-ITAM activation signaling, and this preference required the presence of Lyn (Fig. 3c and Supplementary Fig. 5b). Interestingly, silencing of the PKCα isoform also abrogated SHP-1^S591^ phosphorylation under conditions of FcγRIIA multimeric aggregation and favored SHP-1^Y536^ phosphorylation (Fig. 3d), which was Lyn-dependent (Fig. 3e). However, PKCα silencing had no effect on the phosphorylation status of SHP-1 observed in the absence of Fyn (Fig. 3f). Moreover, PI3K inhibition or PKC-α silencing did not impair Syk activation following FcγRIIA-ITAM induction in the presence or absence of Lyn or Fyn (Supplementary Fig. 3c). Together, these results indicate that during ITAM-induced activation signals, Fyn inactivates SHP-1 through phosphorylation of the S591 involving a PI3K–PKCα axis, thereby blocking its activation by Lyn. Our results demonstrate that Lyn is crucial to maintain ITAMi-mediated homeostasis, whereas Fyn is essential for ITAM-mediated cell activation by inducing the PI3K–PKCα signaling axis that inactivates SHP-1 during inflammatory responses for most immunoreceptors.

### Opposing control of SHP-1 activity regulates inflammation

**Fig. 4:**
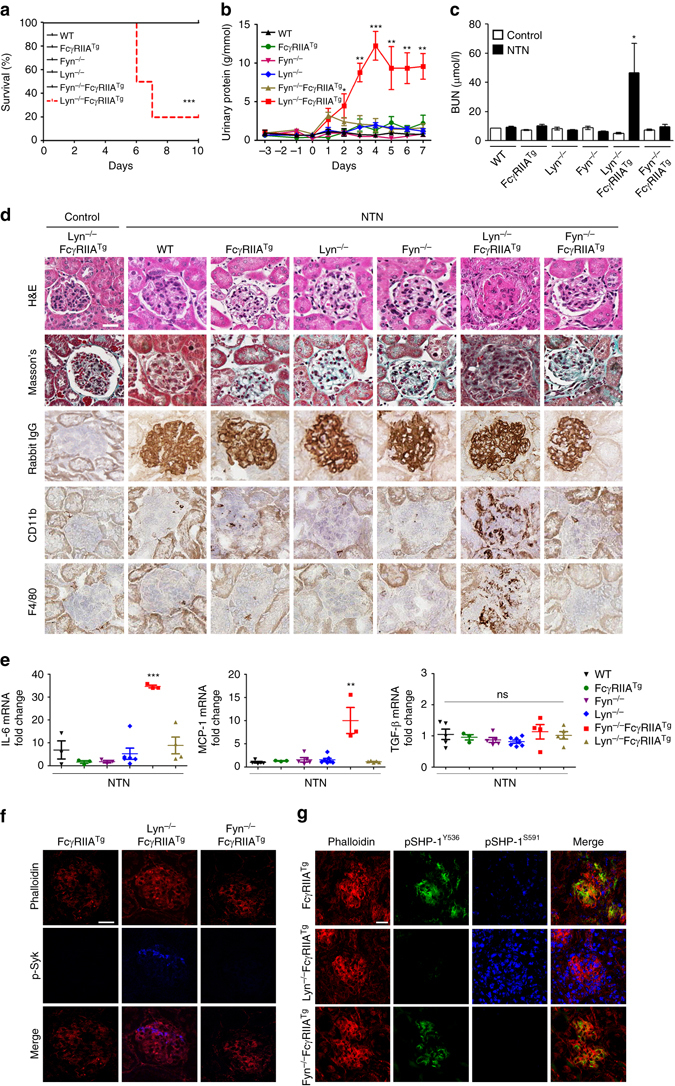
Lyn-SHP-1^Y536^ axis protects mice against lethal nephritis. **a** Survival curves, **b** proteinuria, and **c** serum blood urea nitrogen (BUN) after NTN induction. **d** Rabbit IgG deposit quantification by immunohistochemistry (IHC) (*top panels*), haematoxylin & eosin (H&E), and fibrosis Masson’s stain (*middle panels*) of kidney sections from one representative out of nine mice. Immunostaining for CD11b^+^ and F4/80^+^ cells in kidney sections of the indicated mouse lines (*bottom panels*). **e** Relative gene expression of indicated cytokines assessed by q-PCR of independent kidney tissue RNA samples. *P < .05, **P < .01, ***P < .001; Mann–Whitney test. Non significant, ns. Data are from at least five mice per group. **f** Representative photomicrographs of glomeruli stained for phalloidin and p-Syk-Alexa 647. **g** Representative photomicrographs of glomeruli stained for phalloidin, p-SHP-1^S591^-Alexa 647 and pSHP-1^Y536^-Alexa 488. *Scale bars*: 200 μm

To investigate the functional role of Lyn and Fyn in the regulation of ITAM signals in vivo, we employed a mild immune-complex nephrotoxic nephritis (NTN) model via administration of a rabbit anti-mouse glomerular basement membrane (anti-GBM) serum. A mild NTN model was chosen because FcγRIIA^Tg^ and Lyn-deficient mice have a pre-established autoimmune phenotype^23, 25, 29^. Consistent with the above-described role of Lyn in maintaining ITAMi-mediated immune homeostasis, i.p. administration of the NTN serum led to a severe acute nephritis associated with high mortality at day 7 in Lyn-deficient FcγRIIA^Tg^ recipients, whereas mice deficient for Lyn or Fyn and Fyn-deficient FcγRIIA^Tg^ mice did not develop significant disease despite similar glomerular rabbit antibody deposits and no significant differences in mouse IgG anti-rabbit IgG responses (Fig. 4a and Supplementary Fig. 6a). Renal disease development in Lyn-deficient FcγRIIA^Tg^ mice was characterized by a marked increase in urinary protein (Fig. 4b) and blood urea nitrogen concentration (BUN) (Fig. 4c). They also exhibited severe renal injury involving extensive mesangial and capillary (subendothelial or even intracapillary) deposits associated with mild mesangial and endocapillary plus extra-capillary proliferation (Fig. 4d and Supplementary Fig. 6b–d). Glomerular lesions were characterized by an intense macrophage infiltrate and cytokine production (Figs. 4d, e and Supplementary Fig. 6e). These effects involved ITAM activation signaling, as demonstrated by in situ phosphorylation of the Y525 residue in Syk (Fig. 4f). This was associated with a strong in situ phosphorylation of SHP-1 on the inhibitory S591 residue but not on Y536 (Fig. 4g, middle panels). Moreover, no involvement of SHIP-1-mediated inhibitory signaling by macrophage FcγRIIB was observed in situ after NTN induction in FcγRIIA^Tg^ mice, as glomerular immunofluorescence stainings with anti-phospho SHIP-1 antibody were completely negative (Supplementary Fig. 7). By contrast, NTN induction in Fyn-deficient FcγRIIA^Tg^ mice led to in situ phosphorylation of SHP-1 on Y536 but failed to induce S591 phosphorylation, and this was associated with no renal inflammation despite the presence of antibody deposits on the glomerular membrane (Fig. 4g, bottom panels). Interestingly, the same observations were made in 8-week-old FcγRIIA^Tg^ mice (Fig. 4g, top panels). These results suggest that the absence of Fyn in vivo may protect against autoimmune disease development favoring the activating phosphorylation of SHP-1 on tyrosine residue.

**Fig. 5:**
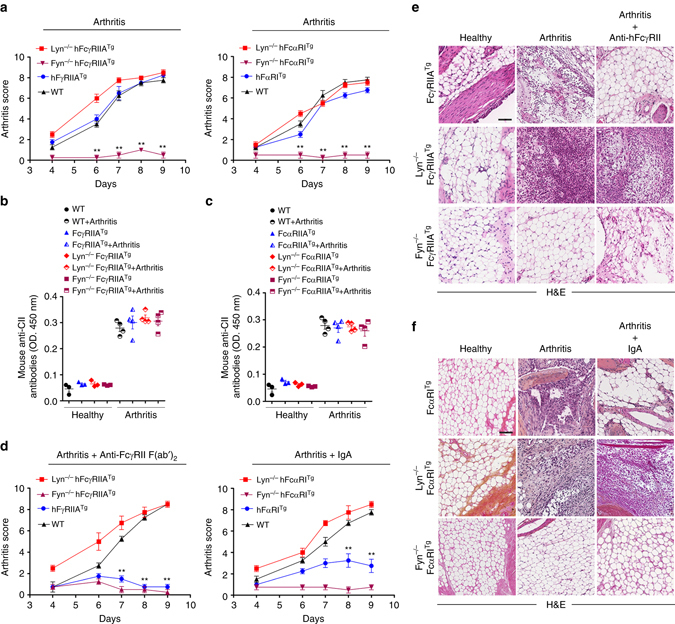
Fyn axis is essential for arthritis development. **a** Fyn-deficient mice failed to develop autoimmune arthritis following induction of the CAIA model both in hFcγRIIA^Tg^ and hFcαRI^Tg^ background. Arthritis score was graded blind as 0 (normal), 3 (mild), 6 (moderate), or 9 (severe). **b**, **c** Circulating levels of CII-specific antibodies in individual sera from WT, FcγRIIA^Tg^ (**b**) and from FcαRI^Tg^ (**c**) mice on the indicated backgrounds at day 10 after injection of antibodies. Mean ± s.e.m. (*n* = 4) (**d**) Lyn is essential for FcR-ITAMi-mediated protection of arthritis. Arthritis score evaluated as in **a**. **e**, **f** H&E staining of hind paws from representative indicated mice at day 10. *Scale bars*: 200 µm. Mean ± s.e.m (*n* = 8). **P < .01; two-way ANOVA test

**Fig. 6:**
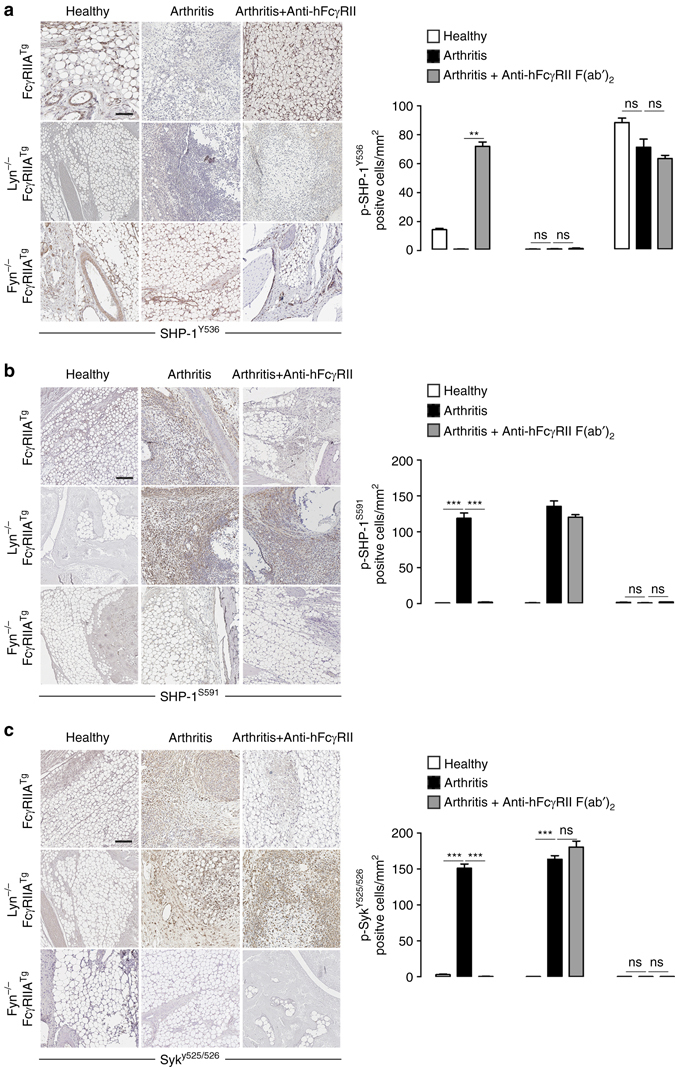
Inactive SHP-1^S591^ is linked to arthritis development. **a**–**c** Immunostaining for the detection of phospho SHP-1^Y536^ (**a**), phospho SHP-1^S591^ (**b**), and phospho Syk^Y525–526^ (**c**) in hind leg sections of indicated mice subjected or not to the CAIA model. The corresponding quantifications of positive cells for phospho-SHP-1 on Y^536^ or S^591^ and phospho-Syk Y^525/526^ are shown on the *right* of each panel. *Scale bars*: 200 µm. **P < .01, ***P < .001; non significant ns, Mann–Whitney test. Sections of three mice/group were automatically quantified with the software CaloPix piloted in a blind manner. Data are presented as the mean ± s.e.m (*n* = 5)

To investigate the aggravating role of Fyn in inflammatory disease development, we took advantage of another autoimmune disease model, namely the collagen antibody-induced arthritis (CAIA) using two transgenic animals expressing either hFcγRIIA or hFcαRI. The aggravating role of Fyn was highlighted by the absence of lesions in both Fyn-deficient FcγRIIA^Tg^ and Fyn-deficient FcαRI^Tg^ mice despite similar serum levels of injected mouse anti-collagen antibodies (Fig. 5a–f). By contrast, Lyn-deficient FcγRIIA^Tg^ and Lyn-deficient FcαRI^Tg^ mice showed enhanced arthritis development characterized by leukocyte infiltration (Fig. 5a–f). To determine whether targeting ITAMi could have a therapeutic value, we treated transgenic animals expressing human FcγRIIA or FcαRI for ITAMi signaling with anti-FcγRII F(ab′)_2_ or monomeric hIgA, respectively, as previously described^11, 32^. ITAMi induction prevented disease development, and this protection required the presence of Lyn but not Fyn (Fig. 5d–f). We next addressed the role of the different phosphorylated forms of kinases and phosphatases in joints of arthritic FcγRIIA^Tg^ mice. While Lyn protected the host against autoimmunity by inducing a constitutive phosphorylation of SHP-1^Y536^ residue, Fyn favored autoimmunity by inactivation of SHP-1 through the phosphorylation of the SHP-1^S591^ residue associated with induction of Syk^Y525–526^ phosphorylation (Fig. 6a–c). Moreover, induction of ITAMi by anti-FcγRIIA targeting was associated with in situ detection of Lyn-dependent pSHP-1^Y536^, but not pSHP-1^S591^ (Fig. 6a–c). Altogether, these results support that non-redundant SFKs are crucial in dictating ITAMi/ITAM balance that controls homeostasis as well as inflammatory and autoimmune disease development.

### Fyn-SHP-1^S591^ axis is linked to lupus nephritis activity

**Fig. 7:**
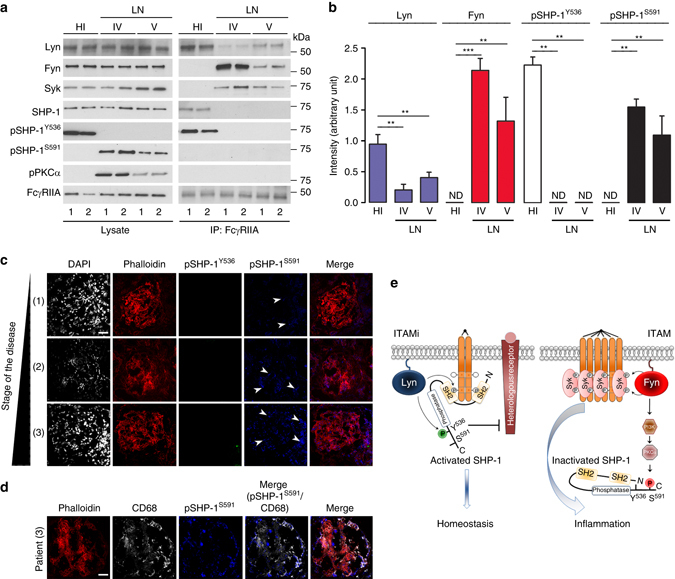
Fyn-SHP-1^S591^ axis as a biomarker of lupus nephritis activity. **a** Analysis of phosphorylated ITAM effectors in blood leukocyte samples from 4 lupus nephritis (LN) patients (1 and 2 are class IV; 3 and 4 are class V) and four healthy individuals (HI). *Left panel*, cell lysates were immunoblotted using indicated Abs. *Right panel*, FcγRIIA immunoprecipitation (IP) with an anti-FcγRIIA monoclonal antibody (IV.3) followed by immunoblotting using indicated antibodies. **b** Quantification of the indicated band using ImageJ software relative to total levels of the corresponding protein in cell lysates. *P < .05, **P < .01, ***P < .001; Mann–Whitney test. Not detectable (ND). Data are mean ± s.e.m (*n* = 5). No significant differences in blood phagocyte counts were observed between healthy controls and patients as followed: Monocytes 0.50 ± 0.16 vs. 0.49 ± 0.30; Neutrophils 4.0 ± 0.42 vs. 3.4 ± 2.4, ×10^6^/ml, respectively. **c** Representative photomicrographs of glomeruli stained from biopsies of lupus nephritis patients for phalloidin (*red*), p-SHP-1^S591^-Alexa 647 (*blue*), and pSHP-1^Y536^-Alexa 488 (*green*). *Scale bars*: 200 μm. **d** Representative photomicrographs of glomeruli stained from biopsies of LN patients for phalloidin (*red*), p-SHP-1^S591^-Alexa 647 (*blue*), and CD68-Alexa 405 (*white*). *Scale bars*: 200 μm. **e** Predicted model for the Lyn and Fyn switch controlling the balance between inhibitory or activating ITAM signals in immunoreceptors. *Left panel*: upon divalent targeting of immunoreceptors, Lyn is recruited to the receptor leading to receptor partial phosphorylation on tyrosine (e.g., Y^304^ in the case of FcγRIIA^11^). Simultaneously, Lyn phosphorylates SHP-1 on Y^536^, inducing a conformational change leading to SH2 domain recruitment to phospho-ITAM, thereby lifting inhibition of the phosphatase domain by the N-SH2 domain. This enables SHP-1 to inactivate signal effectors recruited by heterologous receptors. *Right panel*: multivalent crosslinking of immunoreceptors results in full phosphorylation of ITAM tyrosines by Fyn. These phosphotyrosines serve as “docking” sites for Syk, inducing cell activation and inflammatory responses. Fyn simultaneously activates the PI3K–PKCα pathway, leading to SHP-1 phosphorylation on S^591^. The N-SH2/phosphatase domains are maintained in a closed conformation, blocking both recruitment and activation of SHP-1. Under chronic stimulation, this may lead to aggravation of inflammatory or autoimmune diseases

To examine whether Fyn-mediated inhibitory SHP-1^S591^ phosphorylation were associated with immune complex-mediated disease via the FcγRIIA in patients with a given inflammatory disease, we analyzed blood leukocytes from untreated patients with lupus nephritis at different stages of renal involvement morphologically classified as class IV-A (severe nephritis with immune deposits and leukocyte infiltration) and pure class V (membranous immune deposits only). As shown in Fig. 7a, b, pSHP-1^S591^ and pPKCα were exclusively observed in patient cell lysates, and were not associated with FcγRIIA. Consistent with the role of Lyn/SHP-1 axis in homeostasis, Lyn and pSHP-1^Y536^ were strongly associated with FcγRIIA in healthy individuals only, underlining the inhibitory ITAM homeostatic phenotype, whereas Fyn and Syk were exclusively associated with FcγRIIA in patients highlighting the deleterious role of ITAM signaling in this inflammatory disease. Moreover, tissue analysis of phosphorylated phosphatases in situ of renal biopsies from untreated patients with lupus nephritis at different stages of disease show that the phosphorylation status of SHP-1^S591^ residue was linked to disease activity in proximity of phalloidin^+^ renal cells (Fig. 7c). No phosphorylation of SHP-1^Y536^ was detected on patient kidney biopsies. However, in control experiments, pSHP-1^Y536^ intracellular immunofluorescence staining was observed in THP-1 cells after FcγRIIA-ITAMi induction using the same antibody and, inversely, pSHP-1^S591^ was only detected after FcγRIIA-ITAM-induction (Supplementary Fig. 8). As some pSHP-1^S591^-positive glomerular areas were negative for phalloidin, we next examined leukocyte markers for pSHP-1^S591^ co-expression in biopsies from three patients with lupus nephritis with high disease activity. Figure 7d shows a representative biopsy revealing that significant areas of pSHP-1^S591^ positivity were located in CD68^+^ cells but also in CD68^−^ cells. These results indicate that macrophages, which are present in the inflamed glomeruli express inactive SHP-1 in their cytosol. Moreover, in situ phosphorylation of SHP-1^S591^, but not of SHP-1^Y536^, was associated with spontaneous severe nephritis in 1-year-old pro-autoimmune R131 FcγRIIA^Tg^ mice (Supplementary Fig. 9a–f). This end-stage chronic kidney disease was characterized by enhanced body weight, increased BUN levels, glomerular IgG deposits, sclerotic glomeruli, fibrosis, and kidney interstitial infiltration by CD11b + , F4/80 + and CD3 + cells and increased expression of mRNA coding for proinflammatory cytokines (Supplementary Fig. 9a–e). In this model pSHP-1^S591^ was detected in situ, whereas pSHP-1^Y536^ was detected in the kidney of WT mice (Supplementary Fig. 9f). Altogether, these results support that the type of the phosphorylation of SHP-1 (Y536 vs. S591 residues) determines the shift between the healthy and autoimmune status and can be used as novel biomarkers for lupus disease activity.

## Discussion

Here we characterize the mechanism by which SFKs and SHP-1 control the balance between the activating and inhibitory ITAM signaling. Initially described for their activating function, evidences have accumulated that FcRs bearing an ITAM motif could generate opposite signals, known as ITAMi. Yet, a black box remains in regard to the inhibitory signaling mechanism involved. In this study, we show that low valency aggregations of BCR by anti-CD79a F(ab′)_2_ fragments induced an ITAMi signal characterized by the recruitment of the phosphatase SHP-1, whereas highly multivalent aggregation induced a conventional ITAM activation signal extending the concept that dual ITAM functionality of BCR depends on the valency of the ligand. CD79a-mediated ITAMi inhibitory signals by the BCR is in agreement with previously described pathways to maintain B-cell anergy in which chronic B-cell stimulation results in ITAM monophosphorylation inducing an inhibitory signaling circuit involving SHIP-1 and Dok-1^33^. Interestingly, monophosphorylation of FcγRIIA ITAM is also associated with ITAMi signals but with SHP-1, rather than SHIP-1, recruitment^11^. Whether the number of BCR adapters containing monophosphorylated ITAM is involved in the differential recruitment of phosphatases remains unclear. Our results may also explain a previous observation in which SHP-1 was found to be implicated in the regulation of BCR signaling and in the maintenance of tolerance as B cell-targeted ablation of SHP-1 led to lupus-like disease^19^. Taken together, these findings support the conclusion that immune receptors associated with an ITAM motif, including antigen receptors, can play a dual role as they can induce either activating (ITAM) or inhibitory (ITAMi) signaling depending on ligand avidity.

Our study further demonstrates that single receptors switch between ITAM and ITAMi signals using distinct SFKs fulfilling opposite signaling functions, such as described here for FcR and BCR. Upon divalent targeting of immunoreceptors Lyn, but not Fyn, is recruited to the receptor, leading to ITAM partial phosphorylation on tyrosine, as we previously reported for FcγRIIA^11^, and of SHP-1 recruitment. In contrast, upon multivalent crosslinking of FcγRIIA, Fyn but not Lyn, plays a crucial role in FcγRIIA-ITAM-mediated cytokine production. This Lyn/Fyn switch is not affected by other SFK such as Hck and Fgr. Interestingly, it has been shown that FcγRs can mediate a well-established ITAM-dependent function, the phagocytosis, in the absence of Lyn, Hck, and Fgr^34, 35^. Our data highlight that Lyn and Fyn are essential for ITAM responses as silencing of both SFK render the cell unresponsive for FcγRIIA activating or inhibitory stimuli.

The mechanism by which Lyn and Fyn alternatively switch ITAM-bearing receptor function relies on their control of SHP-1 phosphorylation status. It is now clear that SHP-1 phosphorylation at Y536 induces a conformational change easing SHP-1 recruitment through its SH2 domains to phosphotyrosine residues, thereby lifting the inhibition of the phosphatase domain by its N-SH2 domain^36^. Of note, the two SH2 domains of SHP-1 can cooperate to bind phosphotyrosines on adjacent molecules^37^, a configuration expected to be that of ITAMi. This recruitment would enable ITAMi-mediated SHP-1 phosphatase activity to inactivate signal effectors mobilized by heterologous receptors^38^. In contrast, multivalent crosslinking of immunoreceptors results in the recruitment of the SFKs Lyn and Fyn to the receptor leading to full phosphorylation of the ITAMs. These would serve as “docking” sites for Syk preventing SHP-1 recruitment, which is associated with its phosphorylation on S591 in the cytosol. Thus, Fyn but not Lyn redirects SHP-1 towards its inactivated form allowing simultaneously full ITAM phosphorylation and receptor activation signaling by Syk. As Fyn is a tyrosine, but not a serine, kinase we explored the mechanism involved. SHP-1 is constitutively associated with PKCα^27^. Upon cell activation, PKCα phosphorylates SHP-1 on S591 in its C terminus thereby negatively regulating the activity of this phosphatase^27^. Using a specific inhibitory and siRNA strategy, we found that a Fyn–PI3K–PKCα axis induced the phosphorylation of SHP-1 on S591 upon crosslinking of FcγRIIA in the presence of Fyn, despite the recruitment of Lyn. Since S591 phosphorylation on SHP-1 keeps the phosphatase in a closed conformation^36^, our results suggest that while S591 residue is phosphorylated by Fyn, the Y536 residue is inaccessible to Lyn. Moreover, this study demonstrates also that the absence of Fyn favors the phosphorylation of SHP-1 on Y536 in the presence of Lyn, despite the extensive crosslinking of FcγRIIA. These results indicate that the selective absence or inhibition of Fyn may abolish inflammation during autoimmune and proinflammatory processes. Whether Fyn–PI3K–PKCα axis is involved in previously described Fyn-dependent humoral responses^39^ remains to be demonstrated.

R131H FcγRIIA polymorphism has a strong correlation with the pathogenesis, development and increased susceptibility to several autoimmune diseases, in particular systemic lupus erythematosus (SLE) and rheumatoid arthritis^4, 40, 41^. Interestingly, similar to Lyn-deficient mice, R131H-FcγRIIA^Tg^ mice develop spontaneously autoimmunity at old age, with a complex spectrum of symptoms similar to those found in human rheumatoid arthritis (erosive pannus) and SLE (antinuclear antibodies, glomerulonephritis with immune complex deposition in the renal basement membrane, pneumonitis and non-erosive arthritis)^42^. Several studies have reported that Lyn, but not Fyn, controls IgG-mediated or IgE-mediated systemic anaphylaxis^43, 44^. Our data elucidate the role of these SFKs in the development of autoimmune and proinflammatory disease. Indeed, Fyn and Lyn deletion had opposing effects on the regulation of inflammation observed in R131H-FcγRIIA^Tg^ mice. In the mild model of NTN, ablation of Fyn had no effect on young mice, but that of Lyn induced lethal nephritis associated with the activation of Syk and in situ phosphorylation of SHP-1 on S591. It is unlikely that SHIP-1 plays a negative role in this model through a putative engagement of FcγRIIB on infiltrating macrophages by mouse anti-rabbit Ig antibodies anti-GBM, as no phosphorylation of SHIP-1 was observed in situ. In addition, FcγRIIB function is Lyn dependent^45^ and our results show that Lyn-deficient mice did not develop any severe nephritis as compared to WT mice after NTN induction. The ablation of Fyn, however, protected the R131H-FcγRIIA^Tg^ or FcαRI^Tg^ mice against spontaneous renal autoimmune disease and arthritis development. In agreement with our in vitro data, this was associated with the phosphorylation of SHP-1 on Y536 but not on S591 and with the inactivation of Syk. Consistent with the role of Lyn in the ITAMi-mediated homeostasis, Lyn was strongly associated with FcγRIIA in leukocytes of healthy individuals. In contrast, in lupus nephritis patients, while Lyn weakly associated to FcγRIIA, Fyn, and Syk were highly associated to the receptor. According to our in vitro and in vivo data, the recruitment of Lyn to FcγRIIA was associated with the pSHP-1^Y536^ in healthy individuals. On the opposite, blood leukocytes from lupus nephritis patients display a strong recruitment of Fyn to FcγRIIA, which was associated with pSHP-1^S591^, pPKCα and weak recruitment of Lyn. The selective targeting of Fyn activity may thus become a challenge to open new therapeutic strategies for autoimmune diseases. Moreover, the inactive form of SHP-1 (pSHP-1^S591^) was linked to the severity of inflammatory glomerular lesions in biopsies from lupus nephritis patients and was observed in kidneys of FcγRIIA^Tg^ mice with severe spontaneous nephritis. Interestingly, intra-glomerular CD68^+^ macrophages in class IV lupus nephritis patients with high disease activity expressed pSHP-1^S591^ suggesting that SHP-1 status may contribute to disease pathogenesis by failing to downregulate FcγRIIA activity. We cannot determine from our study whether the pathogenic role for pSHP-1^S591^ involved infiltrating or kidney resident macrophages, as described recently^46^.

This study uncovers how a given receptor discriminates negative or positive signals leading to homeostatic or inflammatory conditions. SHP-1 emerges as a master molecule translating the sensing by distinct SFKs into opposite, activating, or inhibitory, signals (Fig. 7e). This mechanism is at stake in a number of inflammatory and autoimmune diseases, and its targeting could have far-reaching therapeutic value.

## Methods

### Humans

Eighteen individuals (8 healthy individuals and 10 patients with SLE) were studied. The SLE group was composed of 10 patients attending or referred to the Bichat’s Hospital specialist nephrology unit between July 2014 and January 2016 meeting at least four ACR SLE criteria^47^ presenting with active disease with nephritis proven by kidney biopsy (5 at class IV and 5 at class V) and in whom peripheral blood by venepuncture was obtained immediately prior to immunosuppressive therapy administration. All patients were female with age varying between 25 and 42. Ethical approval for this study was obtained from the Bichat Hospital Local Research Ethics Committee and informed consent was obtained from all patients enrolled. Data collection and analyses were performed anonymously.

### Mice

C57BL/6 human FcγRIIA^Tg^ mice expressing the WT human FcγRIIA on CD11b-positive cells were from Jackson Laboratory (JAX, Bar Harbor, ME, USA). Fyn-deficient FcγRIIA^Tg^, Lyn-deficient FcγRIIA^Tg^ were obtained by the intercross of FcγRIIA^Tg^ mice with mice knockout for Fyn (JAX) or for Lyn (previously described in ref. ^44^). All mice were of C57BL/6 strain, female gender between 8–10 weeks old. All mice carrying the FcγRIIA transgene were used as heterozygous animals. Mice were bred and maintained at the mouse facilities of the Bichat Medical School campus. All experiments were performed in accordance with the French Council of Animal Care guidelines and national ethical guidelines of INSERM Animal Care Committee (Animal Use Protocol number 75-1596).

### NTN Mouse Model

NTN was induced by i.p. injection (200 μl/20 g body weight) of rabbit anti-mouse GBM in 8–10-week-old mice. Briefly, mice were preimmunized i.p. with normal rabbit IgG (0.5 mg/20 g body weight) in CFA 5 days prior to i.p. administration of NTN serum. Blood samples were collected and animals were killed at day 7 following NTN injection. Renal function parameters (urinary proteins and BUN), histological and immunohistological parameters were studied.

### CAIA model

Arthritis was induced as described^11, 32^ using the Arthrogen-CIA Arthritogenic Monoclonal Antibody kit (Chondrex, Inc.). Mice were injected i.v. with anti-CII Ab cocktail (Day 0) followed by LPS (i.p.) 3 days later. Animals were injected i.p. with 10 mg/20 g body weight of 500 μg serum human IgA (purchased from Biomedicals)/20 g body weight or 100 µg AT-10 F(ab′)_2_ or irrelevant mAb F(ab′)_2_ (clone 320) for 10 days at 2-day intervals. The first dose was administered 2 days prior to anti-CII Ab cocktail injection. Paw thickness was measured with a pocket thickness gauge. On day 10, animals were sacrificed and hind paws and knees were fixed in formalin or snap-frozen.

### Cells and reagents

THP-1 (ATCC, catalog # TIB-202) and THP1-FcγRIIA-R131^+^-CD14^+^ cell lines (kindly provided by Novimmune)^48^ were maintained in RPMI-1640, 10% FCS and 50 µM β-mercaptoethanol or supplemented with 200 µg/ml Zeocin, 10 µg/ml blasticidin and 2 µg/ml puromycin (Invitrogen, France). Ramos human cell line (Invivogen, catalog # rms-sp) was maintained in RPMI-1640 supplemented with 10% FCS and antibiotics. FCS was removed from the culture medium immediately before stimulation. Human blood samples (12 ml) were first submitted to red cell lysis and pellets of 10^7^ leukocytes were subjected RIPA buffer treatment (see below). BMDM from 6-week-old to 8-week-old mice were obtained after a 7-day culture with M-CSF (R&D systems). Cell lines (THP-1 and Ramos as well as transfectants) were studied. For reagents: Mouse mAbs anti-human FcγRII (clones IV.3 and AT-10), anti-human CD79a (clone ZL7-4) were purchased from Santa Cruz and used in their F(ab′)_2_ fragment forms. Mouse mAb anti-human FcαRI (clone A77) and control mAb (320) were purified in-house and were used as F(ab′)_2_, as previously described^7, 11^. For biochemical studies, rabbit polyclonal anti-Syk, anti–SHP-1, anti-Lyn, anti-Fyn, anti-ERK (all from Santa Cruz Biotechnology, catalog # sc-1077, sc-287, sc-15, sc-16 and sc-153, respectively) at dilution 1/500 in 5% w/v nonfat dry milk, anti-SHP1 (phospho–Y536) (ECM Biosciences, catalog # SP1571) at dilution 1/1000 in 5% w/v nonfat dry milk, anti-SHP1 (phospho–S591) (Abcam, catalog # ab41436) at 1/1000 in 5% w/v BSA and rabbit polyclonal anti-phosphotyrosine (Upstate, catalog # 06-427) antibodies were used. Anti-pERK, anti-pAKT, anti-pPKCα, anti-AKT, and anti-PKCα were from Cell Signalling and catalog # 4377, 4060, 9375, 9272, and 2056, respectively.

### Cell stimulation

For ITAMi signaling, 5 × 10^6^ of monocytic cell lines (THP-1-CD14^+^-FcγRIIA^+^ or THP-1-CD14^+^), and Ramos B cell lines (transfected with different siRNA) were incubated for 30 min with 10 µg/ml of anti-FcγRIIA (clone IV.3) or anti-CD79a (clone ZL7-4) F(ab′)_2_ fragments at 37 °C, respectively. Cells were then incubated with or without LPS (10 ng/ml) as described^8^ or Pam3csk4 (1 µg/ml) for 1 h for monocytic cell lines and 18 h for Ramos. For ITAM signaling, cells were incubated with 10 µg/ml of anti-FcγRIIA (clone IV.3) or anti-CD79a (clone ZL7-4) F(ab′)_2_ at 4 °C followed by an anti-κ light chain F(ab′)_2_ at 37 °C for 18 h for cytokine measurement. For kinase inhibitor experiments, cells were incubated overnight with inhibitors for kinases such as PI3K (LY294002), ERK (PD98059) (both from Cell Signaling Technology), and PKC (GF109203X) (from Sigma Aldrich) or with vehicle alone (1:1000 diluted DMSO in PBS), followed by induction of FcγRIIA-mediated ITAM signals.

### Immunoprecipitation and immunoblotting

Cells (5 × 10^6^ to 10^7^) were solubilized in RIPA lysis buffer containing 1% Nonidet P-40/0.1% sodium dodecyl sulfate (SDS) as described^8^. For immunoprecipitation, cell lysates were incubated with 2 μg/ml of IV.3 anti-FcγRIIA, A77 anti-FcαRI or ZL7-4 anti-CD79a mAbs and immunoprecipitated overnight at 4 °C with Protein G-Sepharose (GE Healthcare). Samples were resolved by SDS polyacrylamide gel electrophoresis (10%), transferred to nitrocellulose membranes and immunoblotted with rabbit antibodies followed by goat anti-rabbit IgG (GE Healthcare) coupled to horseradish peroxidase. Membranes were developed by enhanced chemical luminescence treatment (Amersham Biosciences). All uncut Western blots are available in Supplementary Fig. 10.

### Enzyme-linked immunosorbent assay

IL-8 was measured in the supernatants of stimulated cells using enzyme-linked immunosorbent assay (ELISA) kits (R&D Systems) according to the manufacturer’s instructions. Anti-type I and II collagen mouse IgG antibodies were measured by an ELISA kit with TMB according to the manufacturer’s instructions (Chondrex, Inc, Catalog # 2036 T).

### Real-time PCR

RNA purification from homogenized kidneys was performed by using RNAble (Eurobio). cDNA was obtained by reverse transcription using Moloney murine leukaemia virus (Invitrogen). Samples were analyzed by real-time PCR with Taq Man Gene Expression Master Mix (Applied Biosystem). Primers were purchased from Eurofins. Gene quantification was performed using a Chrom o4 Real-Time PCR Detection System (Bio-Rad Laboratories). Data were normalized to β-actin values. For primers and probe sequences see Supplementary Table 1.

### siRNA transfections

Experiments were performed using predesigned HP GenomeWide (Qiagen, Courtaboeuf, France) siRNAs. For targets DNA sequences and siRNAs sense and anti-sense see Supplementary Table 2. Single-strand sense and antisense RNA nucleotides were annealed to generate an RNA duplex according to the manufacturer’s instructions. Cell lines were incubated with 5–10 nM of each siRNA tested and 2 µl of Lipofectamine RNAiMAX prepared according to the manufacturer’s instructions (Invitrogen, Saint Aubin, France) for 48 or 72 h at 37 °C before use. BMDMs were incubated at day 4 during M-CSF-induced differentiation with 20 nM of each siRNA tested and 2 µl of Lipofectamine RNAiMAX prepared according to the manufacturer’s instructions (Invitrogen, Saint Aubin, France) for 48 h at 37 °C before use.

### Histological and immunofluorescence analyses

For the kidney, paraffin-embedded sections 4 µm in thickness were stained with PAS for morphological analysis. For immunohistochemistry, frozen kidney sections were incubated with biotinylated antibodies against rabbit IgG or monoclonal antibodies anti-mouse CD11b, anti-mouse F4/80, anti-mouse CD3, and anti-mouse Ly6G (Becton Dickinson) for 1 h at room temperature. When necessary, the primary antibody incubation was followed by incubation with anti-rabbit IgG or anti-goat IgG (Southern Biotech Associates). Slides were mounted with the Eukitt mounting medium (Electron Microscopy Sciences) and read with an upright microscope (DM2000; Leica) using the IM50 software (Leica). For colocalization experiments, frozen kidney sections were incubated overnight successively with each antibody (anti-phospho Syk^Y525–526^ AF488 (Cell Signalling) and anti-phalloidin AF568 (Life Technologies) followed by incubation with streptavidin-Alexa Fluor 488 (Life Technologies) at room temperature. Tissue sections were mounted with Vectashield (Vector Laboratories). Slides were read with a laser-scanning confocal microscope (LSM 510; Carl Zeiss) using a 63× objective and the LSM Image Browser (Carl Zeiss). For formalin-fixed hind paws and mouse knees, the samples were decalcified and embedded in paraffin. Sagittal sections were stained with haematoxylin and eosin. Histological score of peri-articular exudates, synovitis and tissue loss were graded as 0 (normal), 3 (mild), 6 (moderate), or 9 (severe). The cumulative score of all features was used as histological score to represent overall disease severity. Analyses were made in a blind manner. Sections were incubated with rabbit primary antibodies against mouse phospho (p)-Syk (Y525-526)^49^, mouse p-SHP1 (S591) (both from Abcam), or p-SHP1 (Y536) (Assay Biotech). The presence of antibodies was detected with biotinylated anti-rabbit antibody (Southern Biotech) followed by peroxidase streptavidin and diaminobenzidine successive stains. Positive cells were quantified in hind paw sections with analysis software CaloPix (TRIBVN, Chatillon, France) after scanning with Aperio ScanScope CS (Leica Biosystems, Nanterre, France). For immunofluorescence studies, frozen kidney slides or cytospined cells were incubated in blocking buffer (PBS, 0.3% saponine, 1% BSA) for 30 min, and then with anti-phospho SHP-1^Y536^ AF488, SHP-1^S591^ AF647 (Bioss Antibodies, 1/50 dilution), anti-CD68 AF405 (Life Technologies 1/50 dilution), and anti-Phaloidin AF568 (Life Technologies,1/100 dilution) for 2 h. Fluorescence was detected by confocal laser scanning microscopy (CLSM-510-META, Zeiss).

### Statistical analysis

All data were expressed as mean ± SEM. Statistical significance between two groups was examined by the Student’s *t*-test or the Mann–Whitney test, while the one-way and two-way analysis of variance (ANOVA) with Bonferroni’s, Holm–Sidak’s, or Newman–Keuls multiple comparisons test were used to evaluate multiple groups. Sample sizes were higher than five per group. *P*-values of 0.05 were considered significant; values <0.05 are indicated in the figure legends.

### Data availability

The data that support the findings of this study are available from the corresponding authors on request.

## Electronic supplementary material

Supplementary Information
