# Supplementary material for: Lyn and Fyn function as molecular switches that control immunoreceptors to direct homeostasis or inflammation
Source: Nat Commun. 2017 Aug 15;8:246. doi: 10.1038/s41467-017-00294-0 (PMC5557797; doi:10.1038/s41467-017-00294-0)
Supplement: Supplementary file 1 — Supplementary Information [file 41467_2017_294_MOESM1_ESM.pdf]

File Name: Supplementary Information
Description: Supplementary Figures, Supplementary Tables and Supplementary References

Supplementary Table 1.

Related primers and probe sequences used for RT-qPCR were:

Gene	Accession Number		5’-3’ Sequence	Location
IL6	NM_031168	F	TCCTACCCCAATTTCCAATGC	526-546
		R	TGAATTGGATGGTCTTGGTCCT	608-587
		P	CAGATAAGCTGGAGTCACAGAAGGAGTGG	555-583
MIP2	NM_009140	F	TGACTTCAAGAACATCCAGATCTT	181-204
		R	CTTGAGAGTGGCTATGACTTCTGTCT	262-237
		P	TGACGCCCCCAGGACCCCA	210-228
MCP1	NM_011333	F	CTTCTGGGCCTGCTGTTCA	107-125
		R	CCAGCCTACTCATTGGGATCA	233-213
		P	CTCAGCCAGATGCAGTTAACGCCCC	160-180
KC	NM_008176	F	TCCCCAAGTAACGGAGAAAGAA	282-303
		R	TGTCAGAAGCCAGCGTTCAC	350-331
		P	AGACTGCTCTGATGGCACCGTCT	307-329
TGF-β	NM_011577	F	TGACGTCACTGGAGTTGTACGG	1610-1630
		R	GGTTCATGTCATGGATGGTGC	1461-1482
		P	TTCAGCGCTCACTGCTCTTGTGACAG	1522-1547
β-actin	NM_007393	F	AGAGGGAAATCGTGCGTGAC	694-713
		R	CAATAGTGATGACCTGGCCGT	831-811
		P	CACTGCCGCATCCTCTTCCTCCC	764-786

Gene, accession number, sequence and location of primers and probes. F indicates a forward primer, R indicates a reverse primer, and P indicates a FAM-TAMRA probe.

Supplementary Table 2.

Name of the gene	Target DNA sequence (5'-3')	Catalog #
LYN	CACTGTTATTAACAGATAATA	SI00605577
FYN	CTCAGAATGTATGTCCCAGAA	SI00605451
SYK	CCCGCTCTTAAAGATGAGTTA	SI02223144
PTPN6	CCGGAACAAATGCGTCCCATA	SI02658726
PKCα	ATGAACTGTTTCAGTCTATAA	SI02738190
	CAGGAGCAAGCACAAGTTCAA	SI02713634
	CAGCTGGTCATTGCTAATATA	SI01388604
	AAGCATTATCTTAGTGGATGA	SI01388583
HCK	CCGGGATAGCGAGACCACTAA	SI02665327
	CGGCAGGGAGATACCGTGAAA	SI02665320
	CGGGAGCACATCAGAGGCTTA	SI02659986
	CCCAGGGATGTCAAACCCTGA	SI04435312
FGR	CACCACACGGGTTCAGTTCAA	SI03057047
	CACGTGGAACGGCAGCACTAA	SI02634807
	TTGATTCTGTAAATAAGTAAA	SI00074494
	CAGACCTTGTCTAGTTATTTA	SI00074473
Control siRNA	AATTCTCCGAACGTGTCACGT	SI03650325

Predesigned HP GenomeWide (Qiagen, Courtaboeuf, France) siRNAs

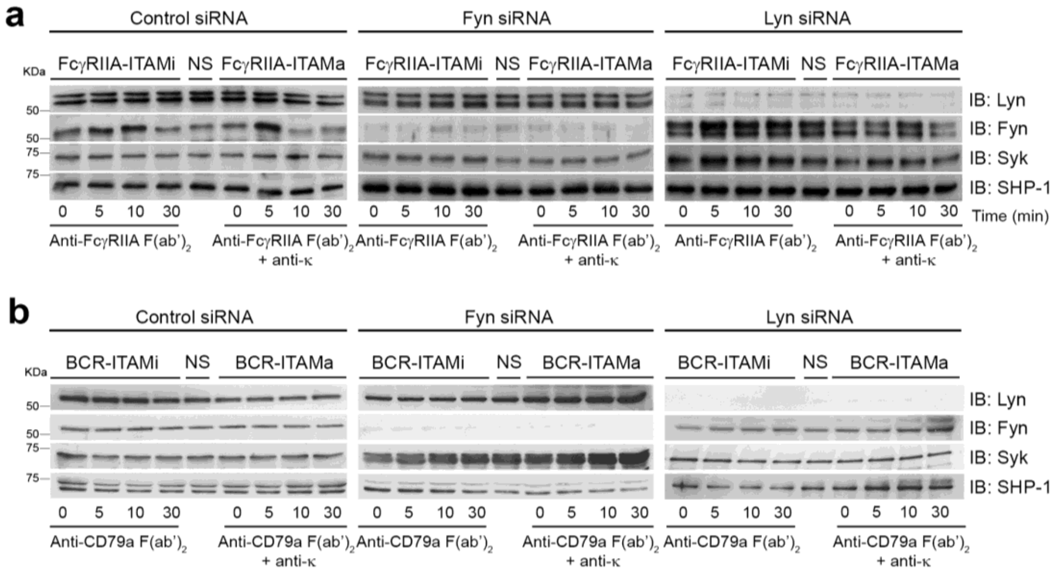

Supplementary Figure 1. Immunoblotting analysis of total kinase or phosphatase protein contents in cell lysates presented in Figure 1a. After induction of FcγRIIA- or BCR-ITAMi or ITAM signalling in THP-1-CD14^{+}-FcγRIIA^{+} cells (a) and Ramos (b) transfected with indicated siRNAs, cells were lysed, lysates subjected to SDS-PAGE and immunoblots (IB) were performed using indicated Abs. NS: not stimulated.

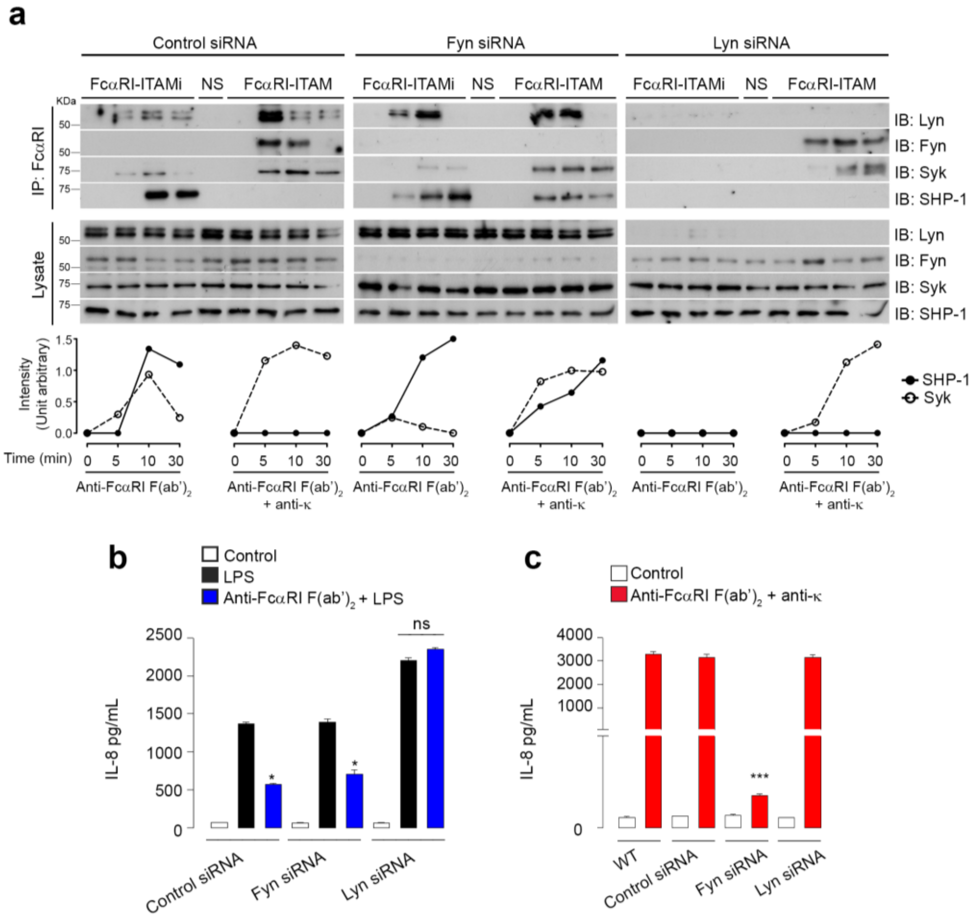

Supplementary Figure 2. Differential regulation of FcαRI-ITAM signals by Lyn and Fyn. (**a**) After induction of FcαRI-ITAMi or ITAM signalling in transfected THP-1 cells, immunoprecipitation (IP) and immunoblots (IB) were performed with indicated Abs. Quantification of the indicated band using ImageJ software relative to total corresponding protein levels in cell lysates is indicated at the bottom of each panel, representing one out of at least three experiments. (**b**) Modulation of LPS-mediated IL-8 production by Lyn and Fyn during FcαRI-ITAMi induction. THP-1 cells transfected with indicated siRNAs were stimulated for 30 min to induce ITAMi signal followed by stimulation with LPS (10 ng/ml) for 1 h. Supernatant was collected for cytokine measurement. (**c**) Modulation of IL-8 production by Lyn and Fyn during FcαRI-ITAM induction for 18 h. Data are presented as the mean ± s.e.m. ***P<.001; Student's unpaired t-test.

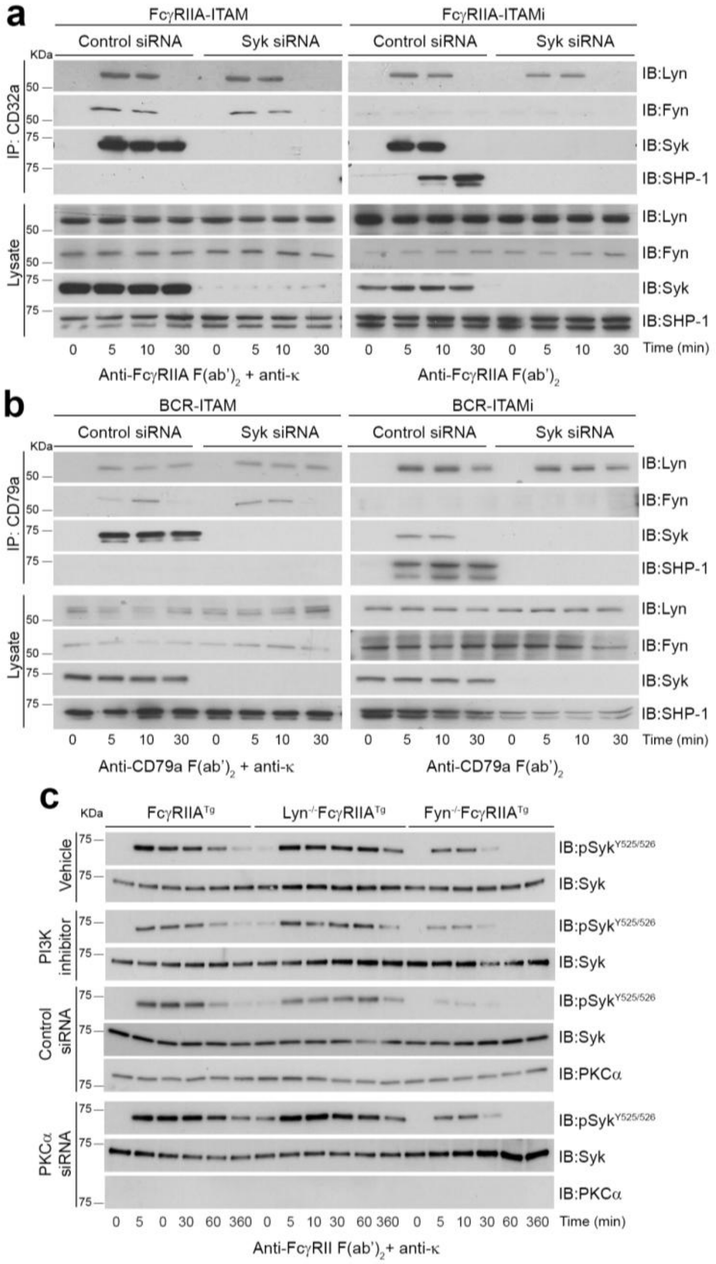

Supplementary Figure 3. Syk is essential for ITAMi signalling but not for Src kinase recruitment. (**a**) After induction of FcγRIIA-ITAMi or ITAM signalling in THP-1-CD14$^+$-FcγRIIA$^+$ cells transfected with indicated siRNAs, immunoprecipitation (IP) and immunoblots (IB) were performed with indicated Abs. Corresponding protein levels in cell lysates are shown in the bottom. (**b**) After induction of BCR-ITAMi or ITAM signalling in transfected Ramos B cells, immunoprecipitation (IP) and immunoblots (IB) were performed with indicated Abs. Corresponding protein levels in cell lysates are shown at the bottom of each panel. (**c**) After induction of FcγRIIA-mediated ITAM signals, BMM derived from FcγRIIA transgenic mice or from FcγRIIA^{Tg} under Lyn- or Fyn-deficient backgrounds were either treated with PI3K inhibitor or transfected with indicated siRNAs. Cell lysate samples were subjected to SDS-PAGE and immunoblots were performed using an anti-phospho (p) Syk (Y525/526) Ab. Corresponding protein levels in cell lysates are shown at the bottom of each panel. Data are representative of three experiments.

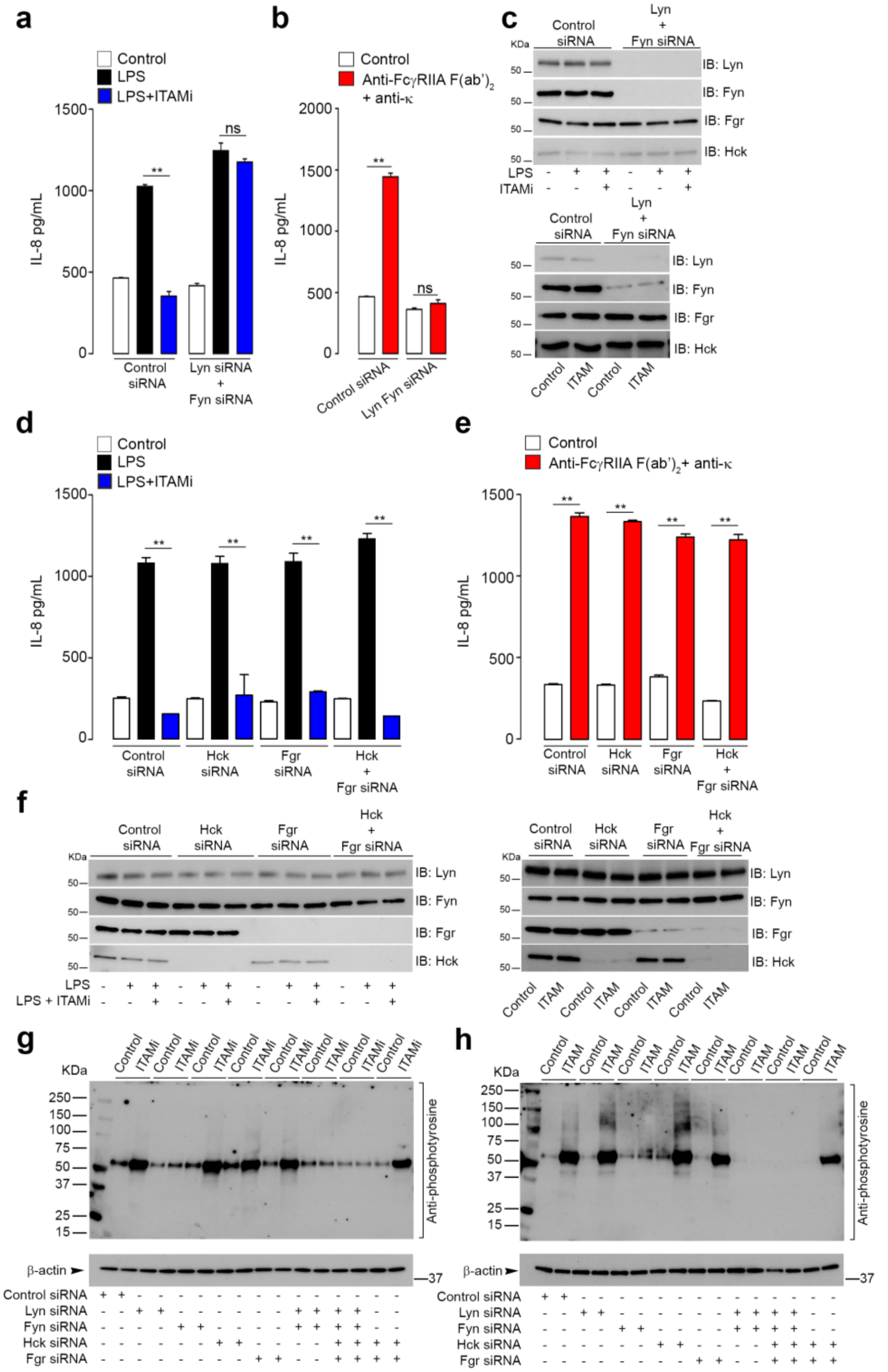

Supplementary Figure 4. Lyn and Fyn, but not Hck or Fgr, are essentials in the control of the balance between ITAM and ITAMi signals. (**a**) THP-1-CD14$^+$-FcγRIIA$^+$ cells transfected with indicated siRNAs were stimulated for indicated time points to induce ITAMi signal followed by stimulation with or without LPS (10 ng/ml) for 1 h. Then, supernatant was collected for cytokine measurement. (**b**) THP-1-CD14$^+$-FcγRIIA$^+$ cells transfected with indicated siRNAs were stimulated for indicated time points to induce ITAM signal for 18 hours. Data are presented as the mean ± s.e.m. ***P<.001; Student's unpaired t-test. (**c**) THP-1-CD14$^+$-FcγRIIA$^+$ cells transfected with indicated siRNAs were stimulated for indicated time points to induce either ITAMi or ITAM signals followed by stimulation with or without LPS (10 ng/ml) for 1 h. Then, cell lysates were subject to immunblot analysis using the indicated antibodies. (**d**) Absence of modulation of LPS-mediated IL-8 production by Hck and Fgr during FcγRIIA-ITAMi induction. THP-1-CD14$^+$-FcγRIIA$^+$ cells transfected with indicated siRNAs were stimulated for indicated time points to induce either ITAMi signals followed by stimulation with or not by LPS (10 ng/ml) for 1 h. Then, supernatant was collected for cytokine measurement. (**e**) Absence of modulation of IL-8 production by Hck and Fgr during FcγRIIA-ITAM induction for 18 hours. Data are presented as the mean ± s.e.m. ***P<.001; Student's unpaired t-test. (**f**) THP-1-CD14$^+$-FcγRIIA$^+$ cells transfected with indicated siRNAs were stimulated for 30 min to induce ITAMi (left) signals followed by stimulation with or without LPS (10 ng/ml) for 1 h, or for 18 h for ITAM (right) induction. Then, cell lysates were subject to immunblot analysis using the indicated antibodies. (**g** and **h**) After induction of ITAMi (**g**) or ITAM (**h**) as described in a and b, siRNA-transfected THP-1-CD14$^+$-FcγRIIA$^+$ cell lysates were subject to SDS-4-16% PAGE followed by immunoblot analysis using an anti-phosphotyrosine antibody. Molecular weight are indicated on the left of each panel. Data are representative of three experiments.

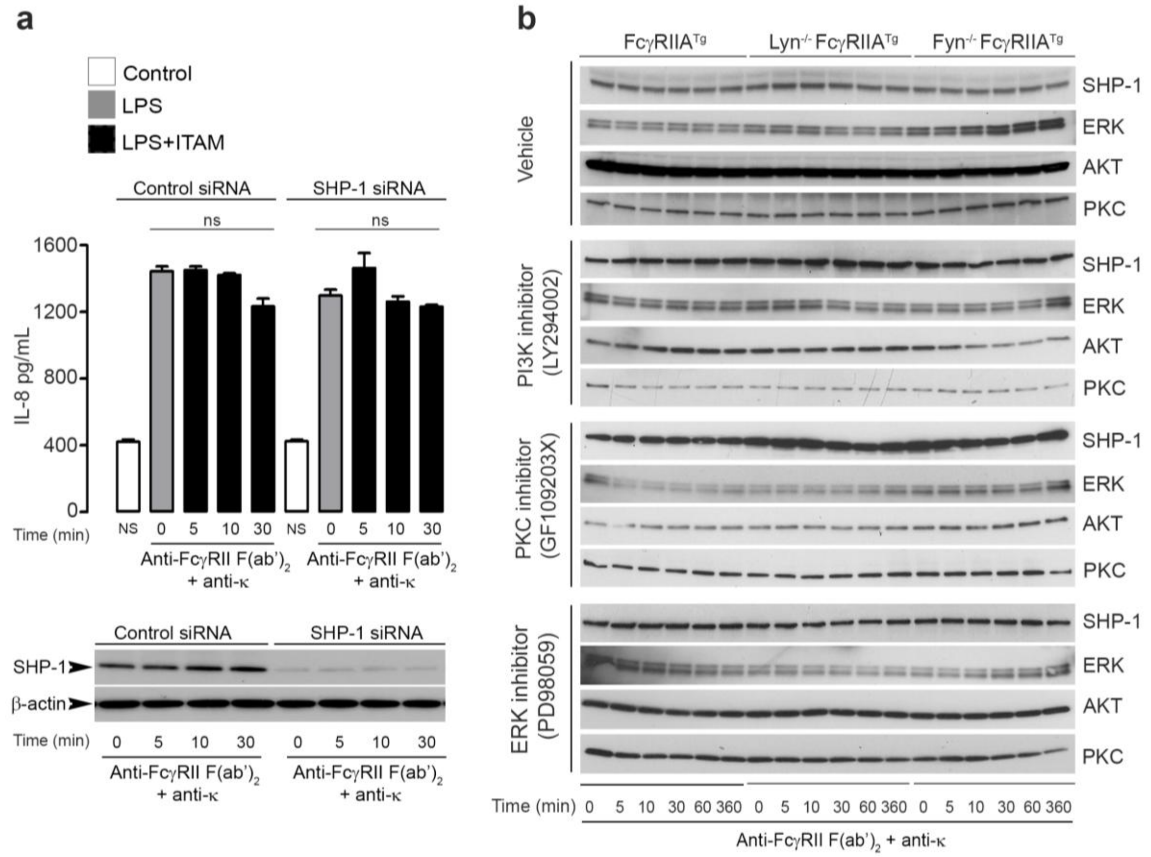

Supplementary Figure 5. Immunoblotting analysis of total kinase or phosphatase protein contents in cell lysates presented in Figure 3c. (**a**) Modulation of LPS-mediated IL-8 production by SHP-1 silencing after induction of FcγRIIA-ITAM signal in THP-1-$CD14^+$-$Fc\gamma RIIA^+$ cells transfected with indicated siRNAs. Cells were stimulated with LPS for 1 h at 37°C after induction of ITAM and IL-8 was measured in the supernatant as described in Figure. 1b. Data are presented as the mean ± s.e.m.; ns, not significant; Student's unpaired t-test. (**b**) BMM derived from FcγRIIA transgenic mice or from $Fc\gamma RIIA^{Tg}$ under Lyn- or Fyn-deficient backgrounds were incubated overnight with PI3K, PKC and ERK inhibitors or with vehicle, followed by induction of FcγRIIA-mediated ITAM signals. BMM lysate samples were subjected to SDS-PAGE followed by immunoblotting (IB) using indicated Abs.

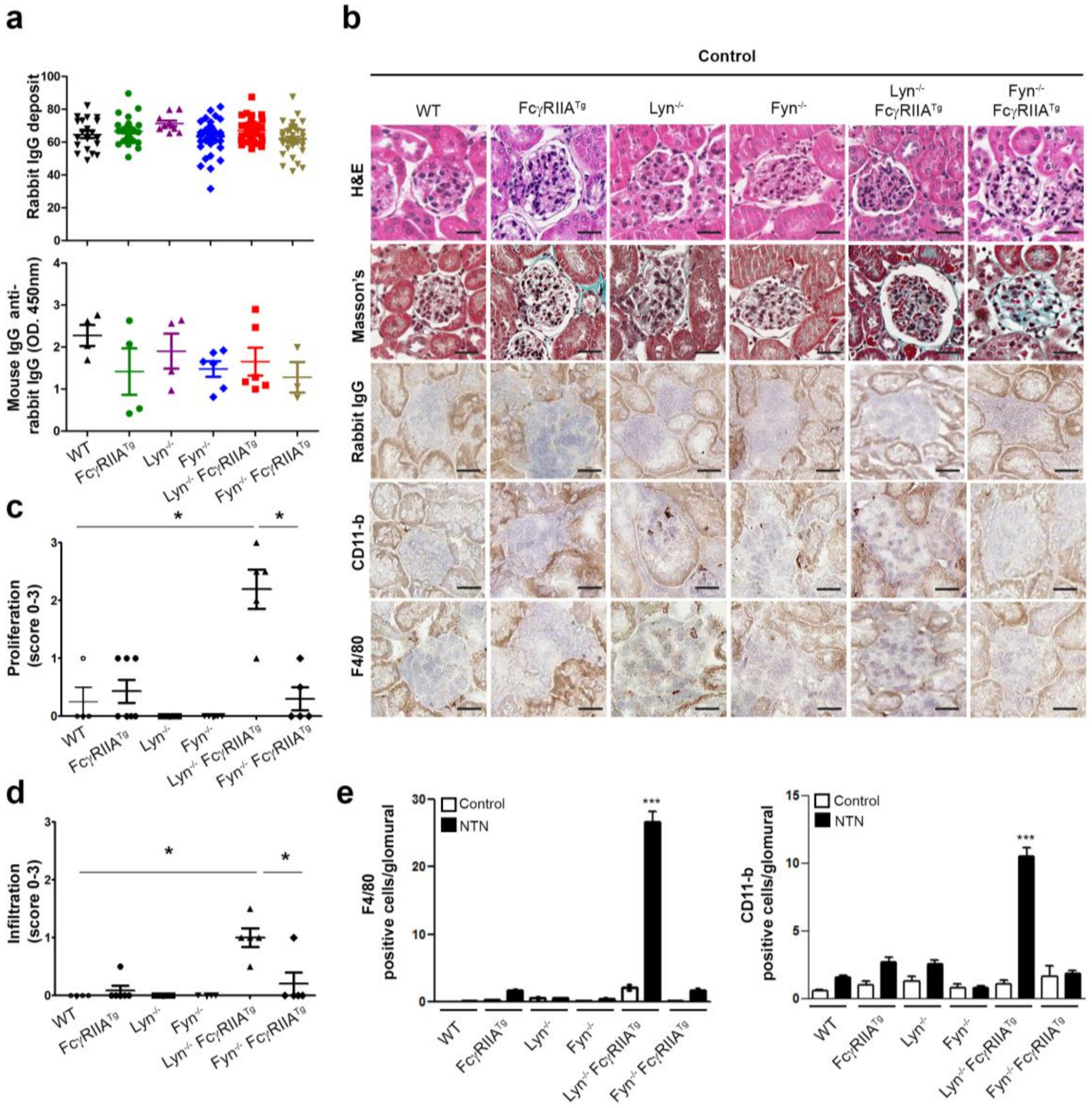

Supplementary Figure 6. Effect of SFK deficiency on nephritis development following NTN mouse model induction. (**a**) Quantification of rabbit IgG deposits on the kidney sections after NTN induction. Sections of five animals/group were automatically quantified with the software CaloPix piloted by an independent pathologist. Proteinuria after NTN injection in indicated strains of mice. (**b**) Circulating levels of CII-specific antibodies in individual sera from WT and FcγRII^{Tg} on different background mice as indicated. (n=3) at day 6. Means ± s.e.m. (**c, top**) H&E and fibrosis Masson's stain of kidney sections from representative mice. Scale bars: 200 µm. (**c, bottom**) Immunostaining of CD11b and F4/80 in kidney sections of indicated strains of mice. Bars: 200 µm. (**d**) Quantification of glomerular cell proliferation and leukocyte infiltration by an independent renal pathologist. **P*<.05; Mann-Whitney test. Data are presented as the mean ± s.e.m (n=8 or 10). (**e**) Quantification of positive cells for F4/80 and CD11b, respectively (*p<.01; two-way ANOVA test). Sections of five animals/group were automatically quantified with the software CaloPix piloted by an independent pathologist.

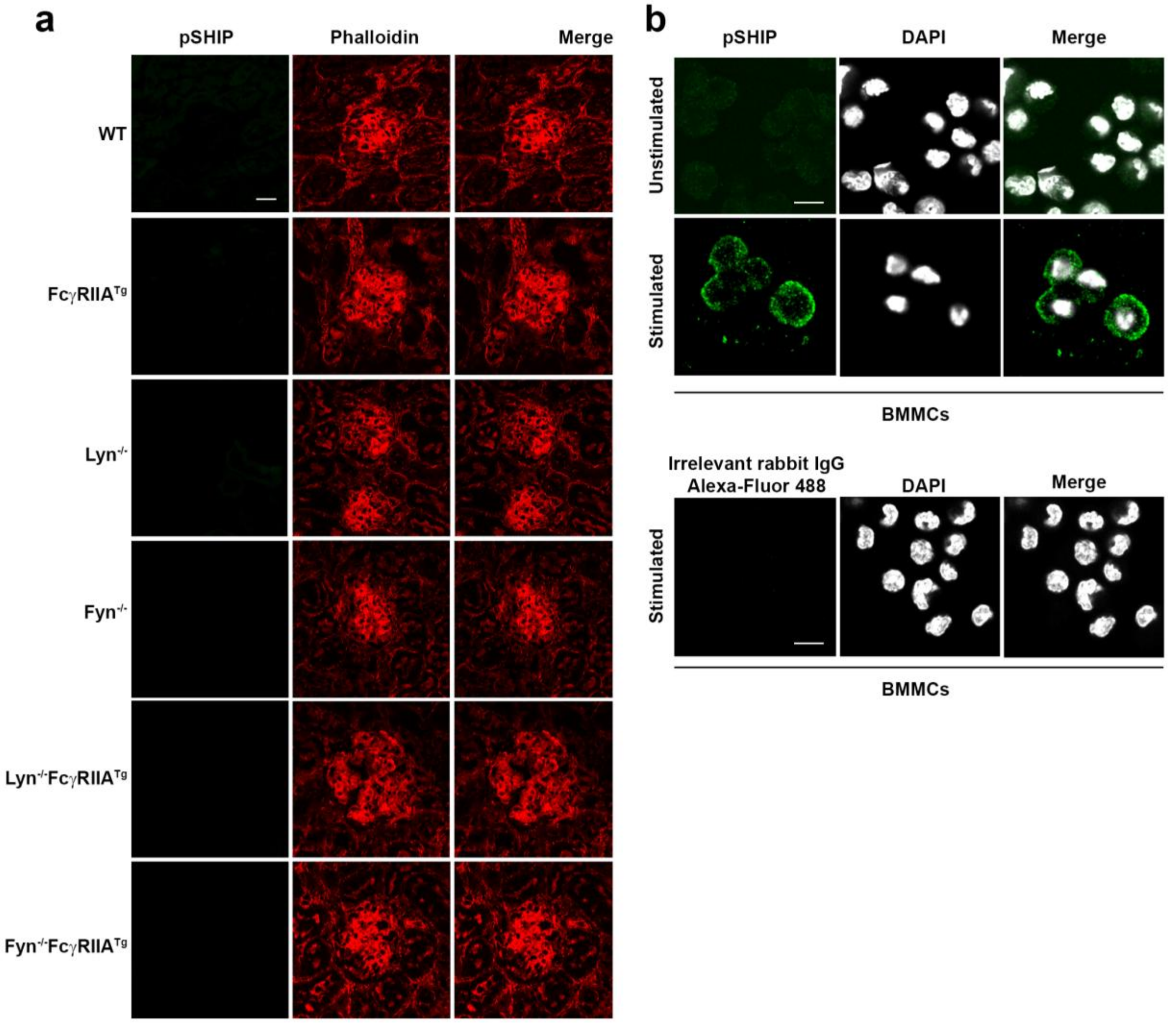

Supplementary Figure 7. Absence of pSHIP activation on the glomerular area following NTN induction. (**a**) Immunoflurescence staining on frozen kidney tissues from indicated mice 6 days after NTN induction using Alexa Fluor 488 conjugated anti-pSHIP antibody or Alexa Fluor 547 conjugated anti-phalloidin to identify the glomeruli. (**b**) Positive control for the Alexa Fluor 488 conjugated anti-pSHIP antibody using bone marrow derived mouse mast cells (BMMCs). Cells were stimulated or not for 5 min with pre-formed immune complexes containing 2.4G2 mAb rat anti-mouse FcγRII/III (κ chain$^+$), mouse monoclonal IgE (κ chain$^+$), and anti-κ chain (rat + mouse) polyclonal antibody. Data are representative of four experiments.

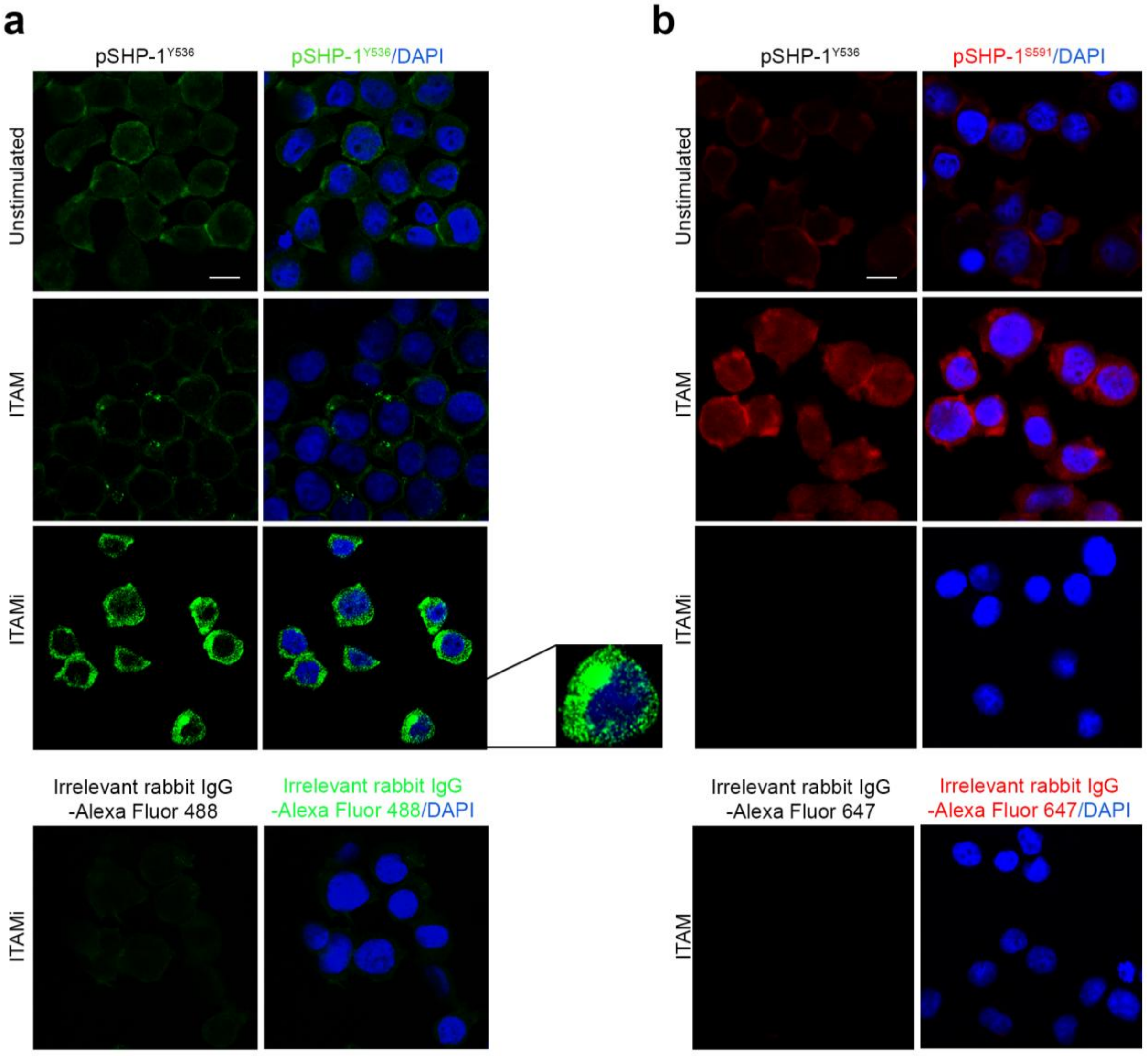

Supplementary Figure 8. Detection of pSHP-1^{Y536} and pSHP-1^{S591} on THP-1 CD32A^{+} cells by immunoflurorescence. Unstimulated or stimulated cells were cytospined, fixed and stained with Alexa Fluor 488 anti-pSHP-1^{Y536} or anti-SHP-1^{S591}, or with DAPI. (**a**) A positive staining for anti-pSHP-1^{Y536} was observed in cells stimulated for 1h by IV.3 F(ab')$_2$ (FcγRIIA-ITAMi conditions), but not under FcγRIIA-ITAM conditions (IV.3 F(ab')$_2$ plus anti-κ chain F(ab')$_2$). Insert indicates ITAMi intracellular inhibisome clusters as described previously [1]. (**b**) On the contrary, positive staining with anti-pSHP-1^{S591} was observed after FcγRIIA-ITAM induction. This was representative of three experiments.

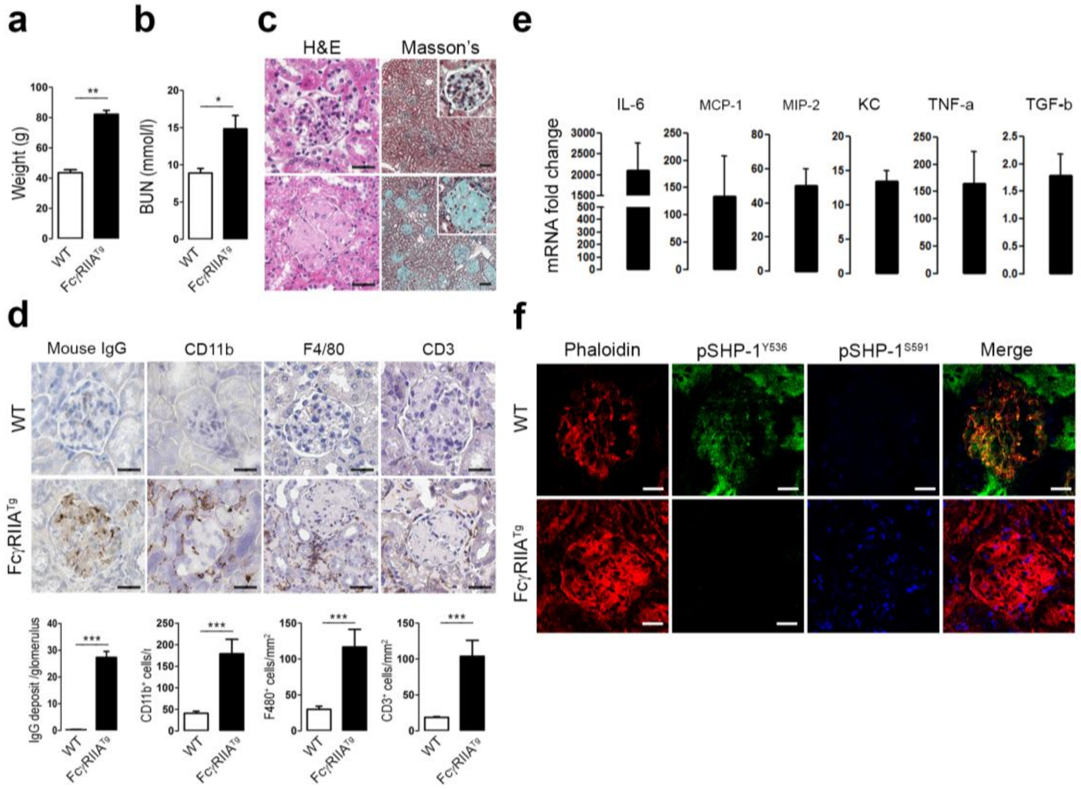

Supplementary Figure 9. Glomerular in situ SHP-1^{S591} phosphorylation is associated with spontaneous development of severe autoimmune nephritis in FcγRIIA^{Tg} mice. (**a**) Body weight. (**b**) Serum BUN. (**c**) Representative photographs of 1 year-old mice showing severe glomerulopathy with marked fibrosis. Scale Bars: 200 µm. (**d**) Detection of mouse IgG, CD11b^{+}, F4/80^{+} and CD3^{+} cells in kidney sections of indicated mice. Quantification of positive cells is indicated on the bottom. Scale Bars: 200 µm. (**P*<.01; Mann-Whitney test). Sections of five animals/group were automatically quantified with the software CaloPix piloted by an independent pathologist. (**e**) Relative mRNA expression of the indicated cytokines assessed by q-PCR on independent kidney tissue RNA samples (n = 5). **P*<.05, ***P*<.01, ****P*<.001; Mann-Whitney test. ns, non significant. Data are presented as the mean ± s.e.m (n=5). (**f**) Representative photomicrographs of glomeruli stained for phalloidin (red), p-SHP-1^{S591}-Alexa 647 (blue) and pSHP-1^{Y536}-Alexa 488 (green). Scale bars: 200 µm.

Supplementary Figure 10: All uncut Western blots for each figure panel are included below

Figure 1a

IP:FcγRIIA, IB: Lyn (control siRNA)

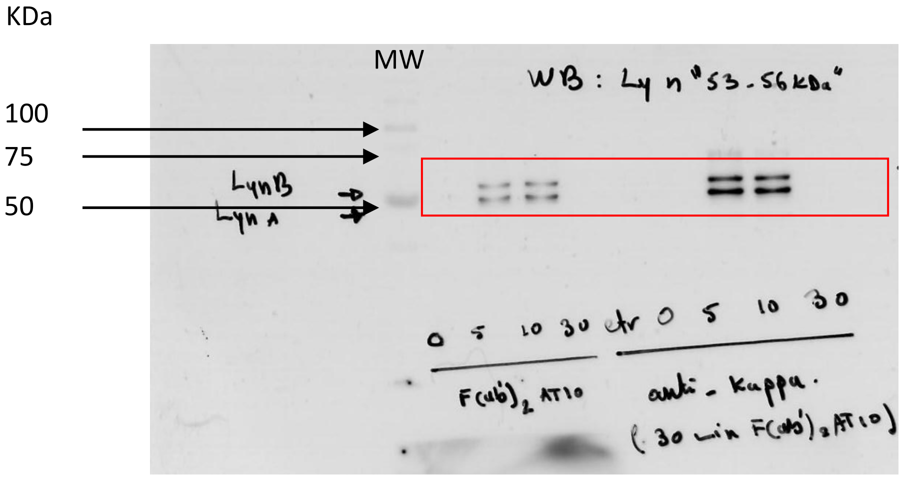

MW: Molecular Weight

Figure 1a

IP:FcγRIIA, IB: Syk (control siRNA)

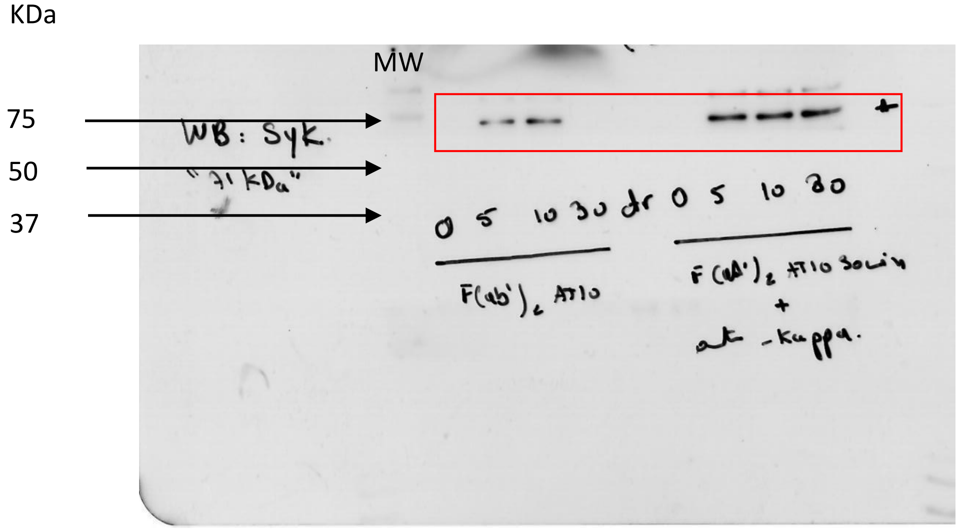

MW: Molecular Weight

Figure 1a

IP:FcγRIIA, IB:SHP1 (control siRNA)

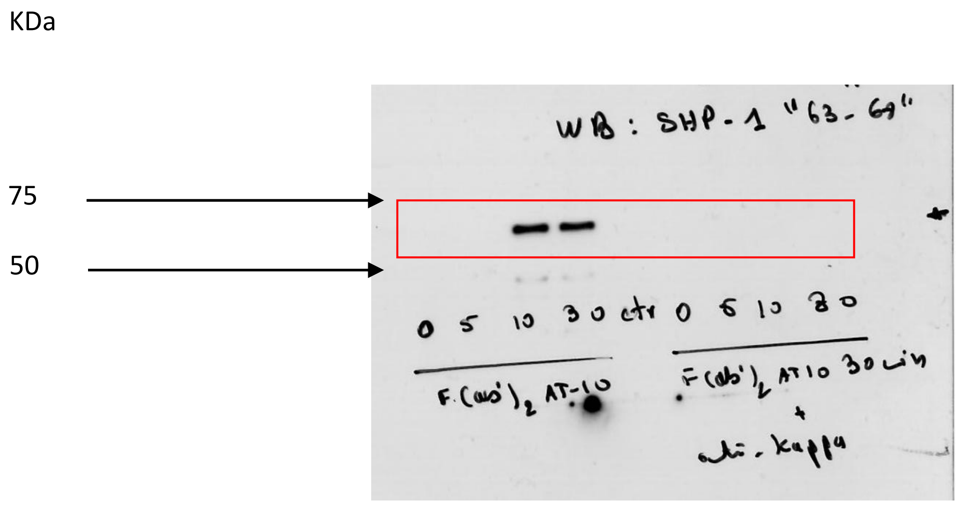

Figure 1a

IP:FcγRIIA, IB:Fyn (control siRNA)

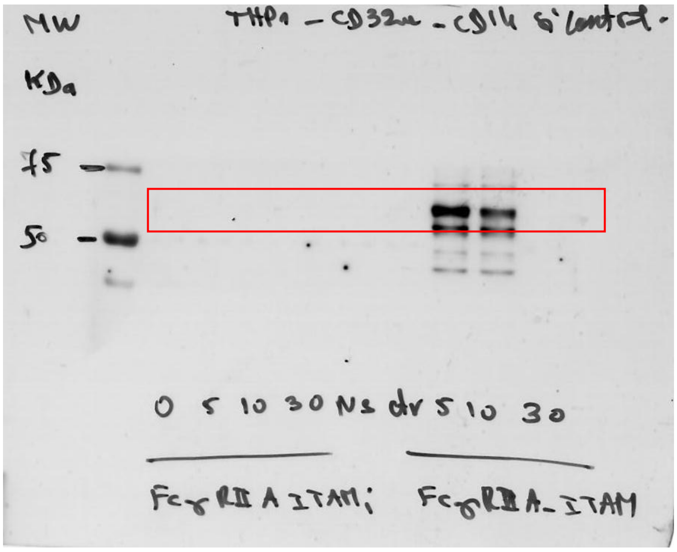

Figure 1a

IP: FcγRIIA, IB:SHP-1 (Fyn siRNA)

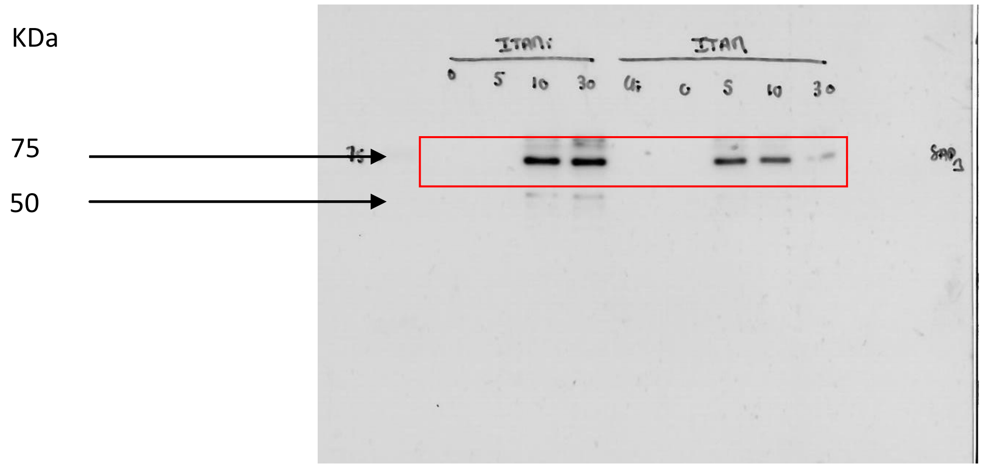

Figure 1a

IP: FcγRIIA, IB:SHP-1 (Lyn siRNA)

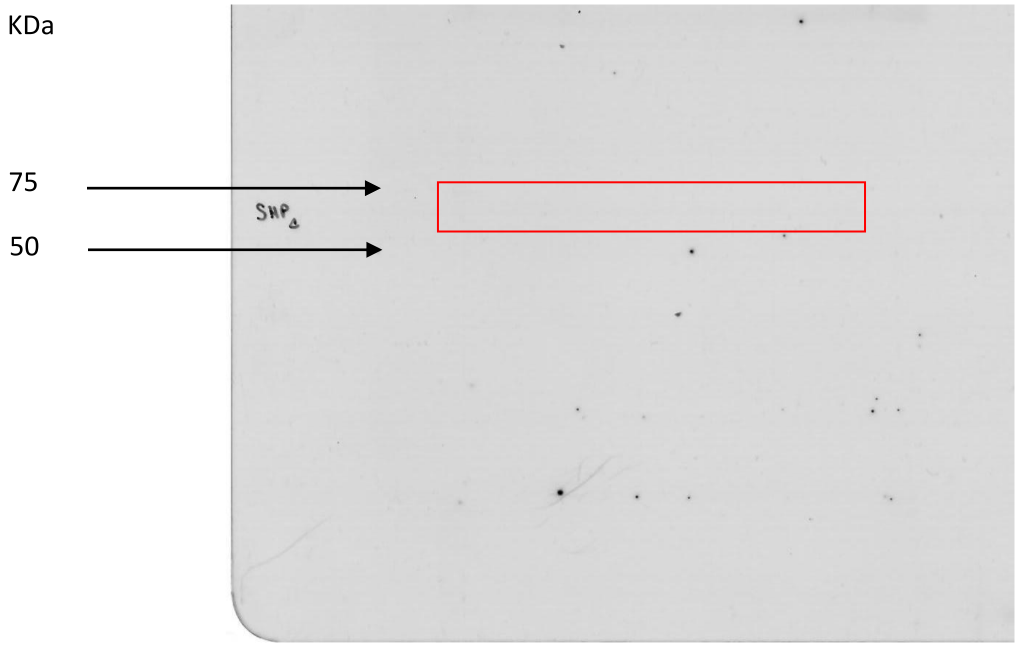

Figure 1a

IP: FcγRIIA, IB:Syk (Fyn siRNA)

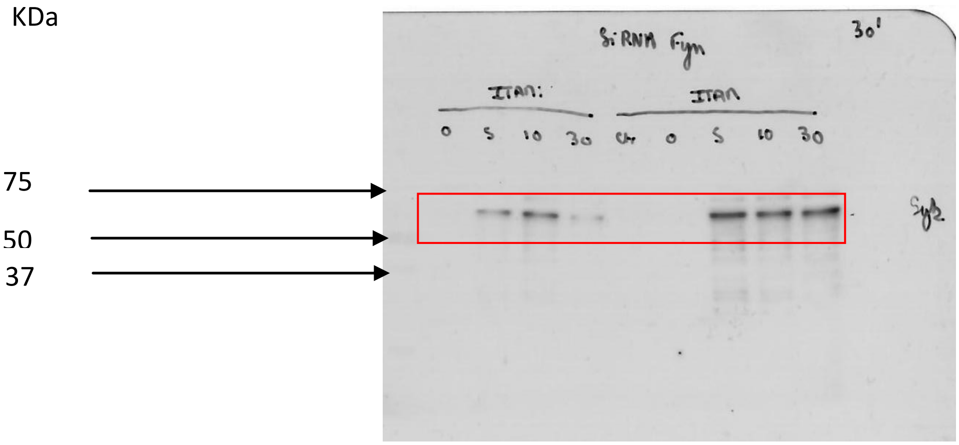

Figure 1a

IP: FcγRIIA, IB: Lyn (Lyn siRNA)

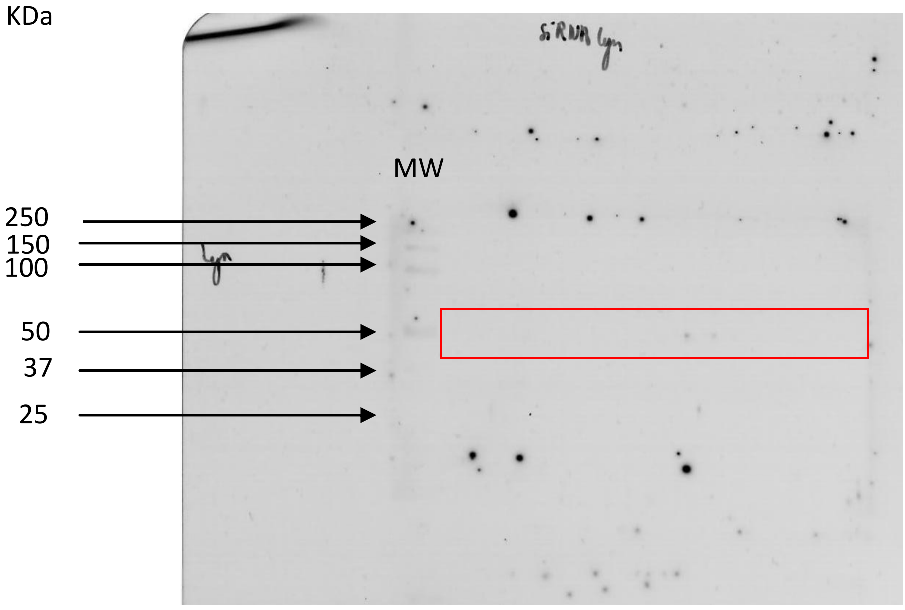

Figure 1a

IP: FcγRIIA, IB: Syk (Lyn siRNA)

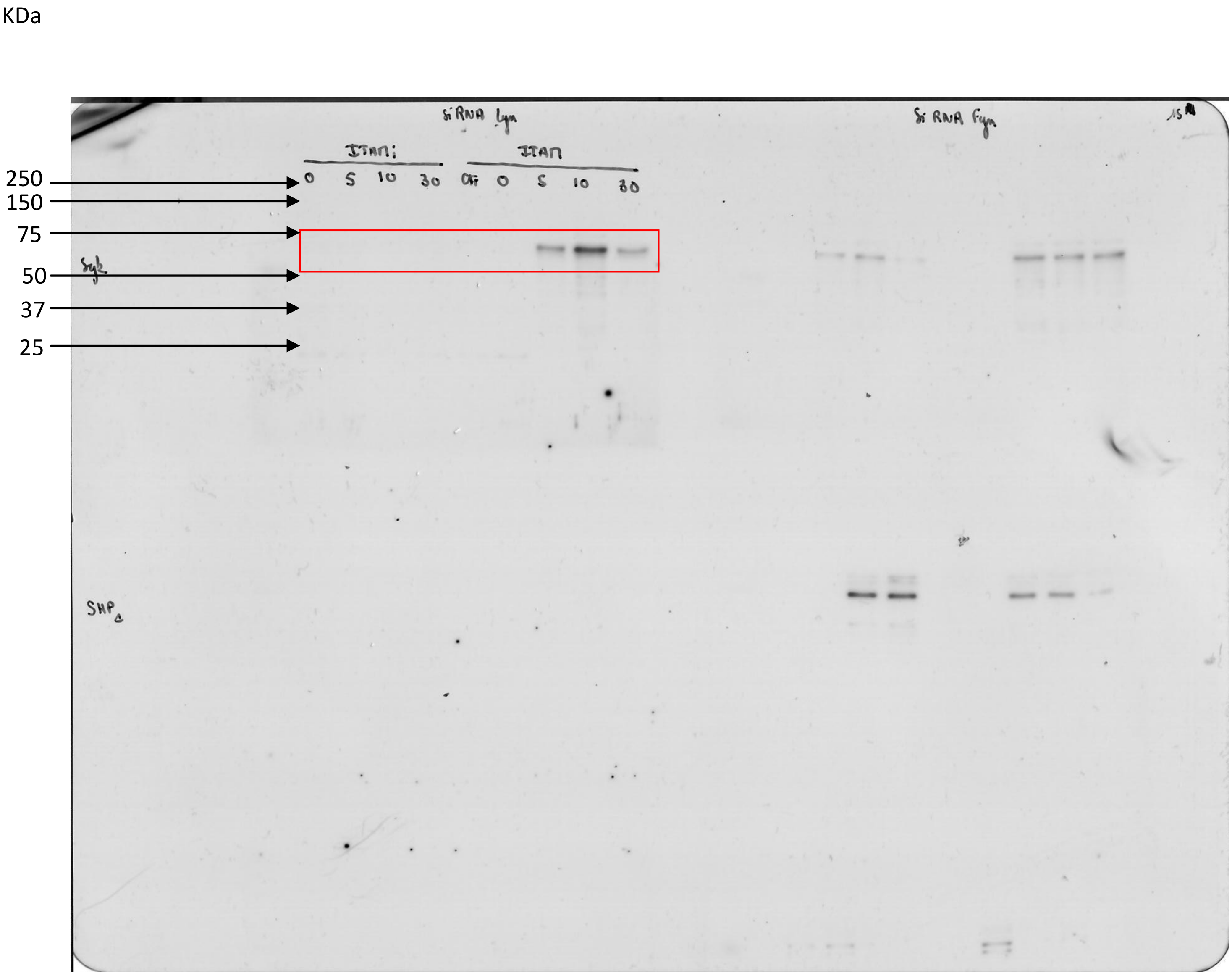

Figure 1a

IP: FcγRIIA, IB: Lyn (Fyn siRNA)

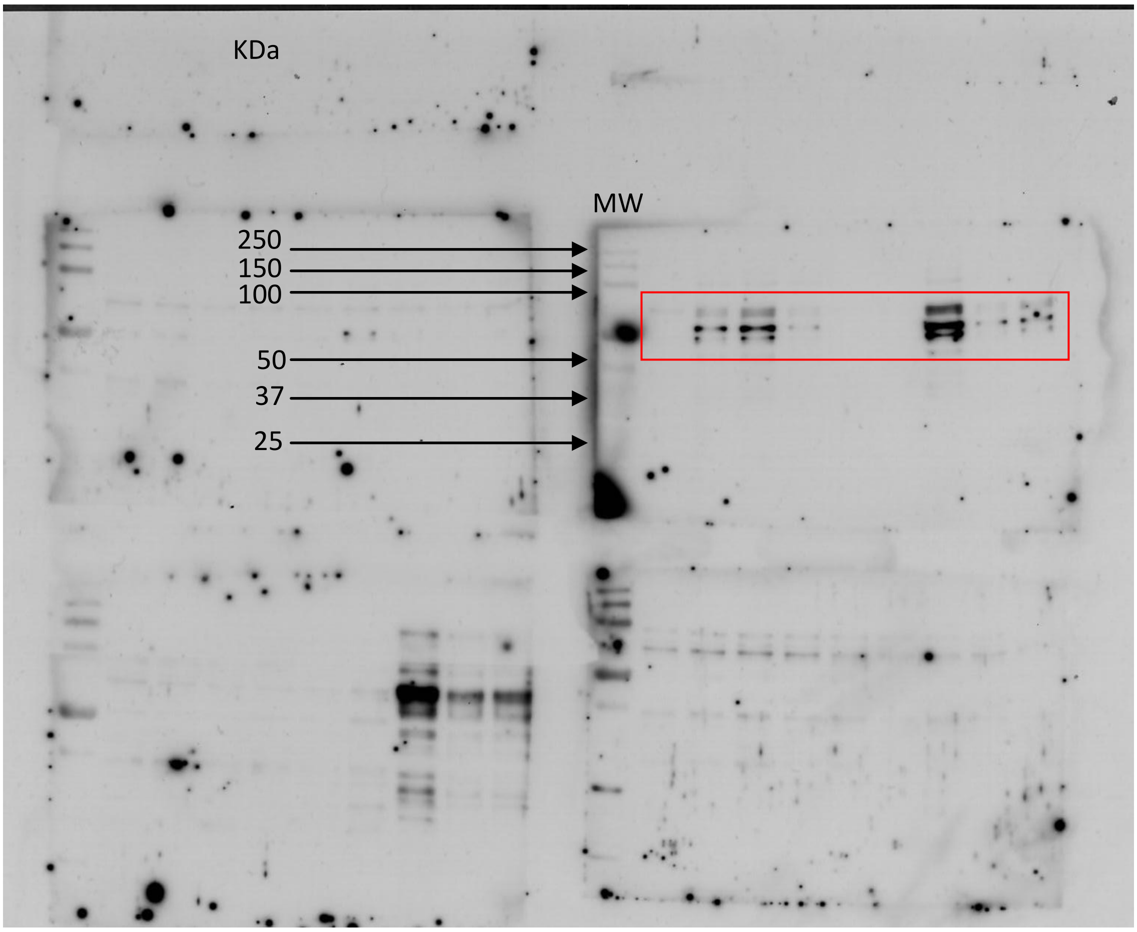

Figure 1a

IP: FcγRIIA, IB: Fyn (Fyn siRNA)

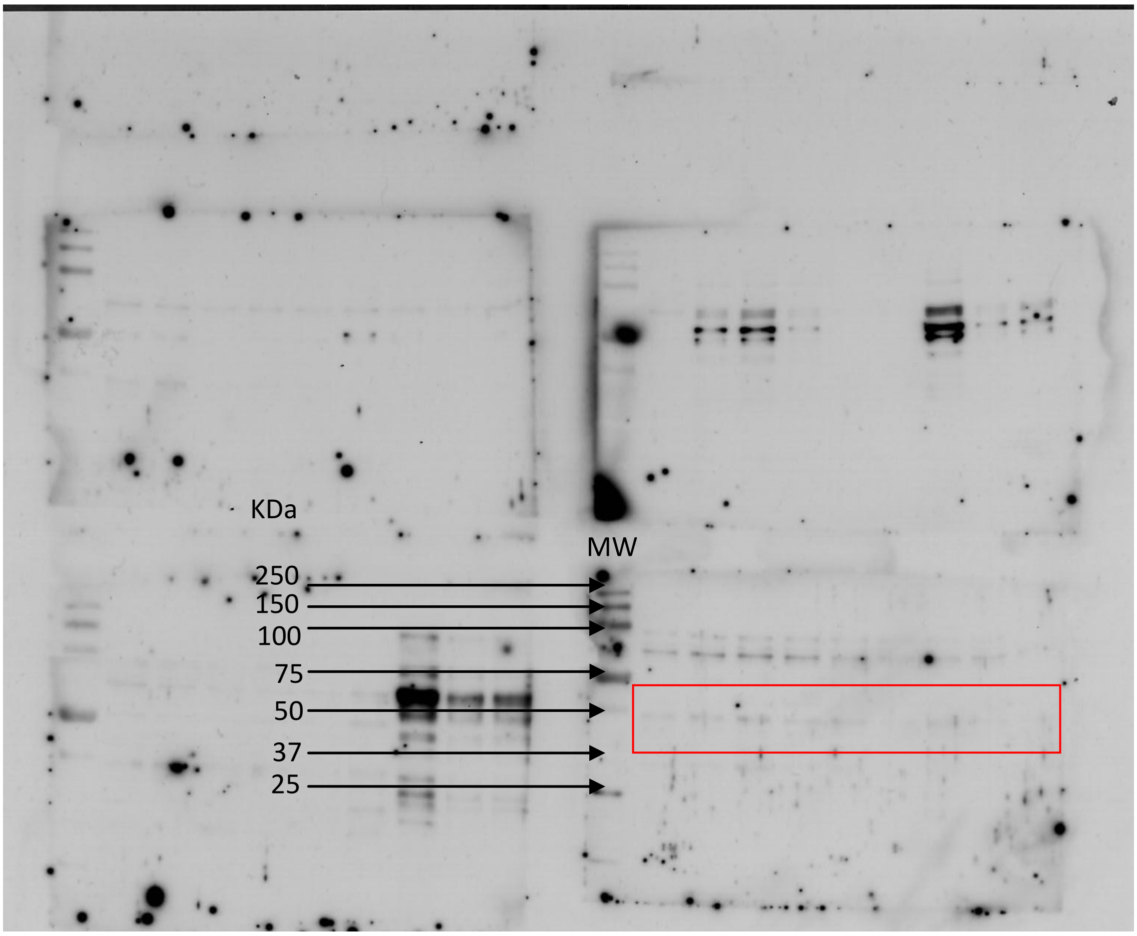

Figure 1a

IP: FcγRIIA, IB: Fyn (Lyn siRNA)

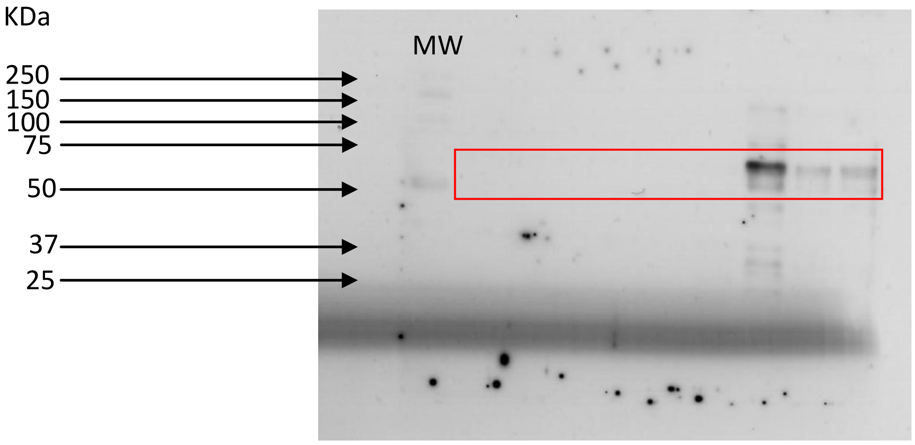

Figure 2a

IP: CD79a, IB: Fyn (Control siRNA)

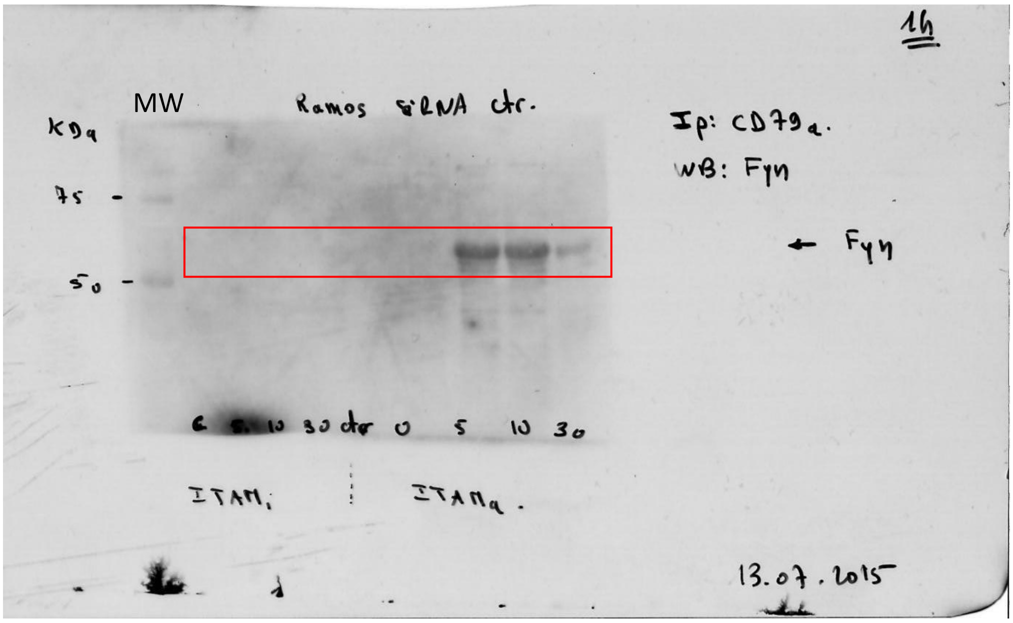

Figure 2a

IP: CD79a, IB: Lyn (Fyn siRNA)

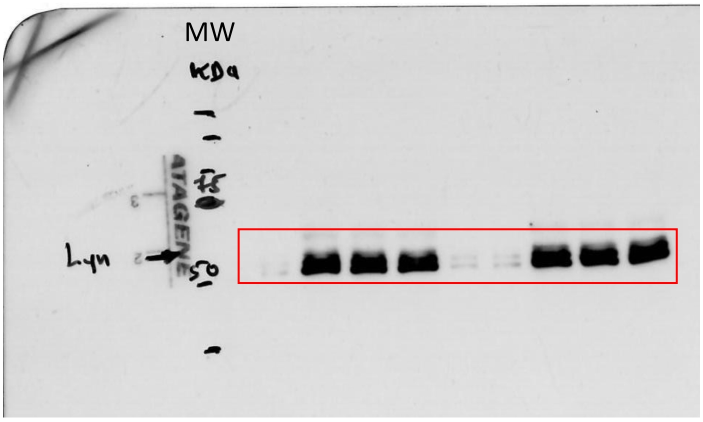

Figure 2a

IP: CD79a, IB: Fyn (Fyn siRNA)

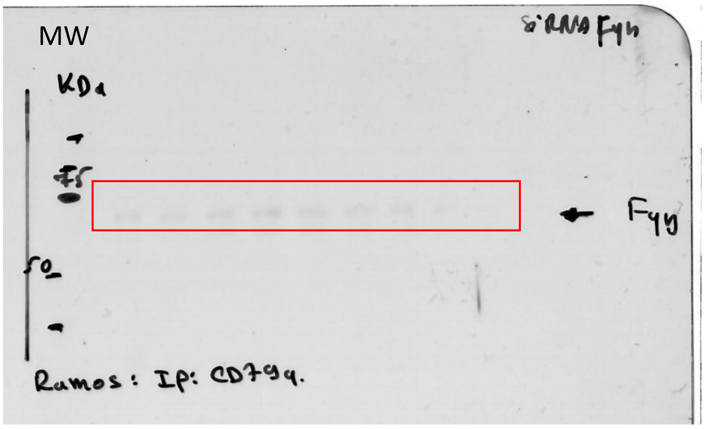

Figure 2a

IP: CD79a, IB: Lyn (Lyn siRNA)

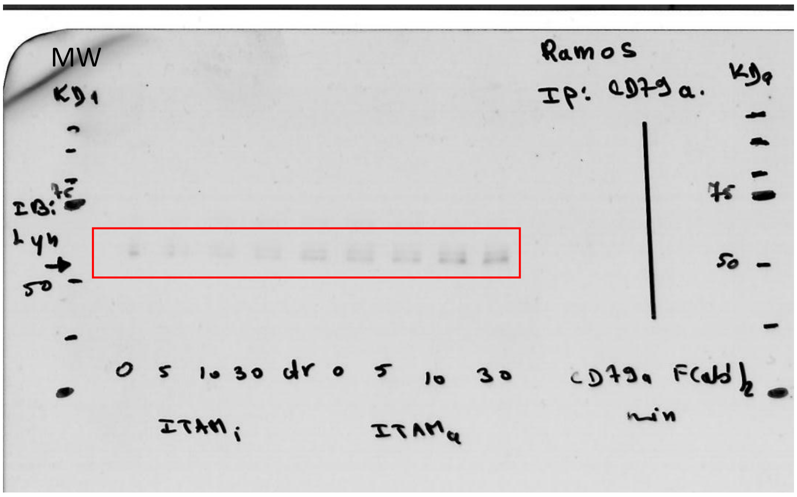

Figure 2a

IP: CD79a, IB: SHP-1 (Lyn siRNA)

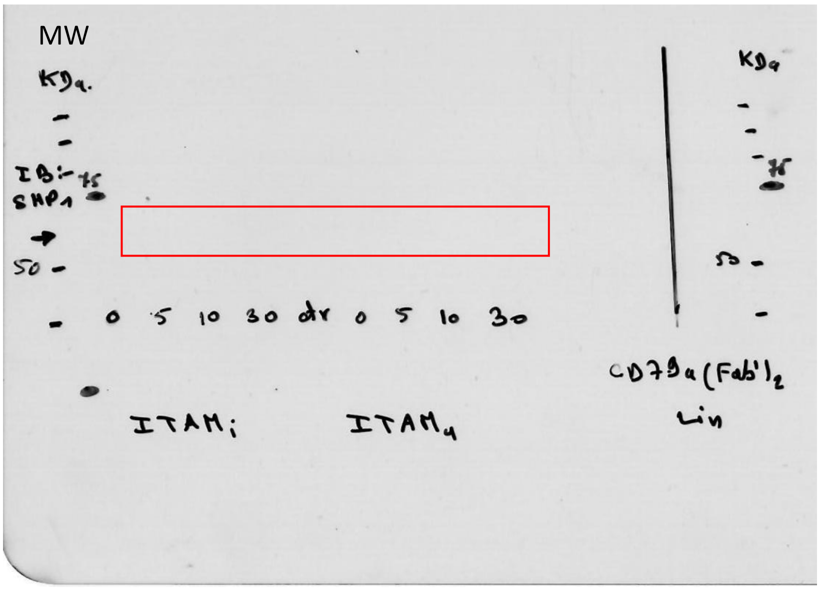

Figure 2a

IP: CD79a, IB: Lyn (Control siRNA)

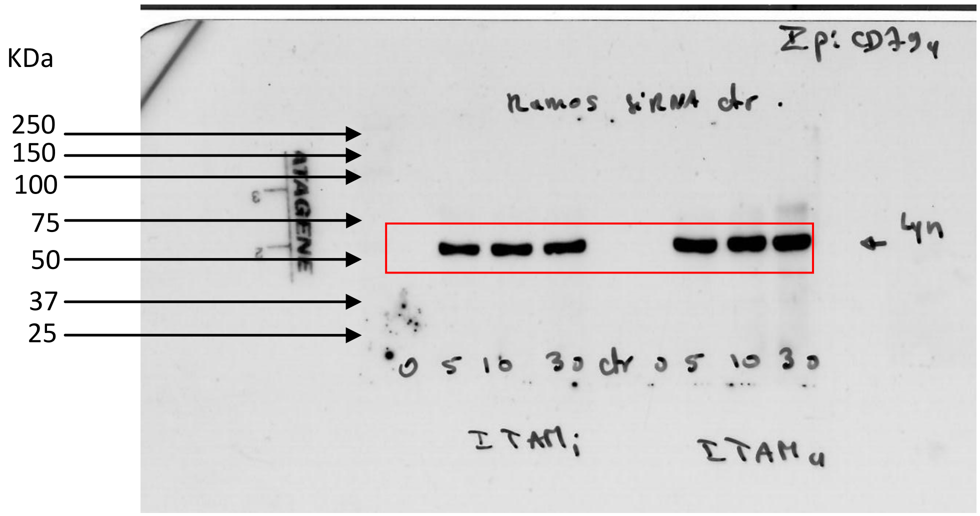

Figure 2a

IP: CD79a, IB: SHP-1 (Fyn siRNA)

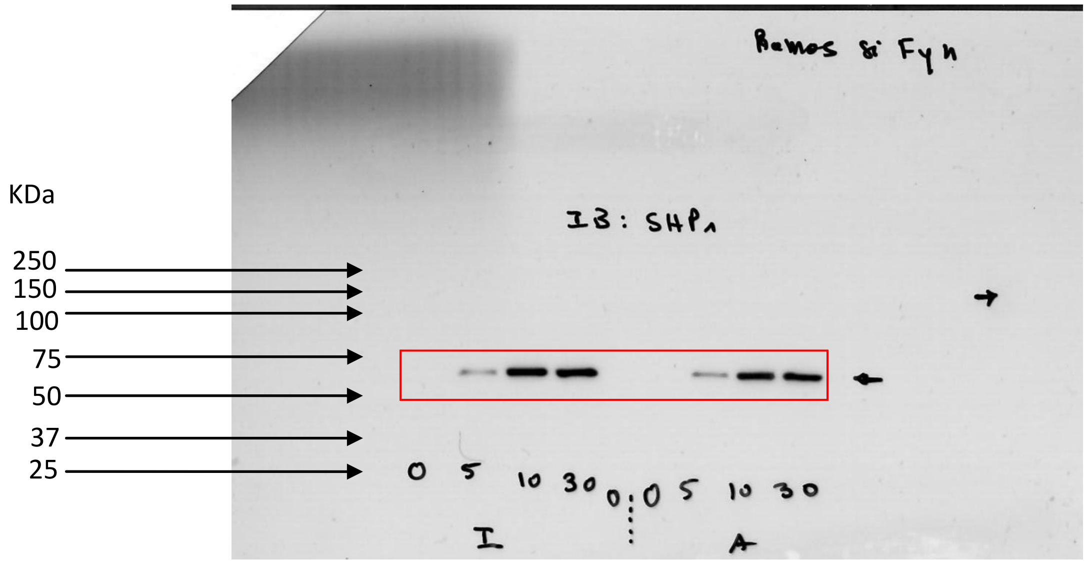

Figure 2a

IP: CD79a, IB: Syk (Fyn siRNA)

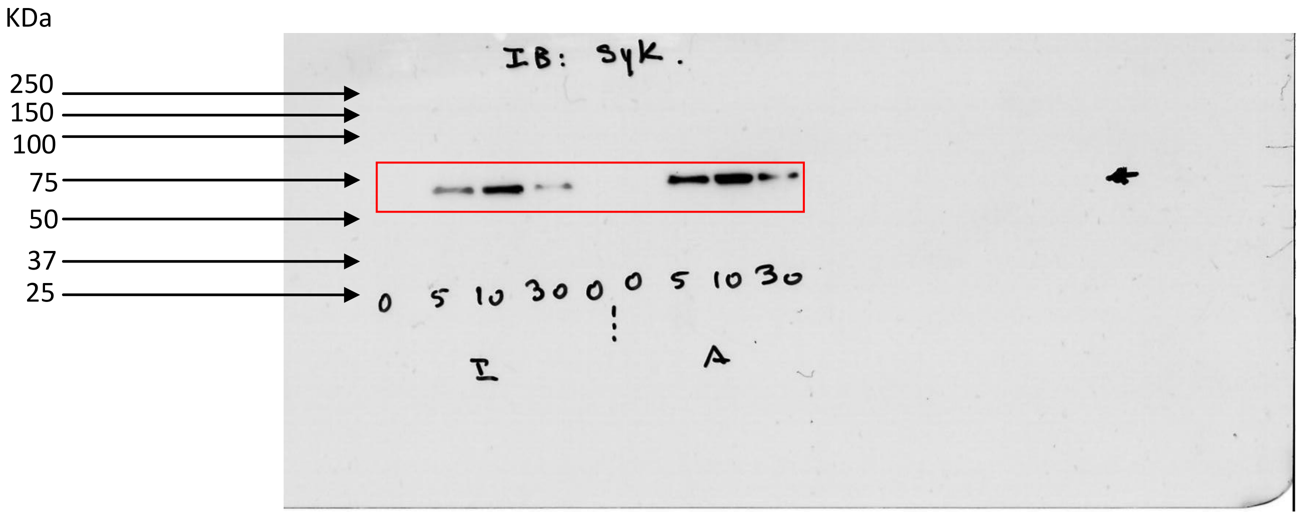

Figure 2a

IP: CD79a, IB: Syk (Lyn siRNA)

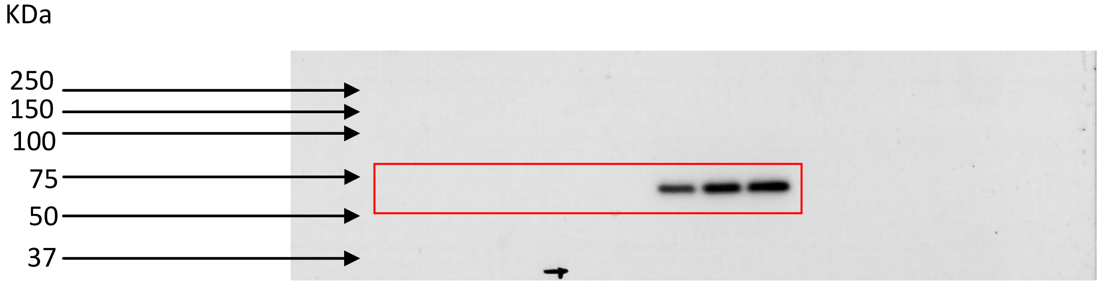

Figure 2a

IP: CD79a, IB: Fyn (Lyn siRNA)

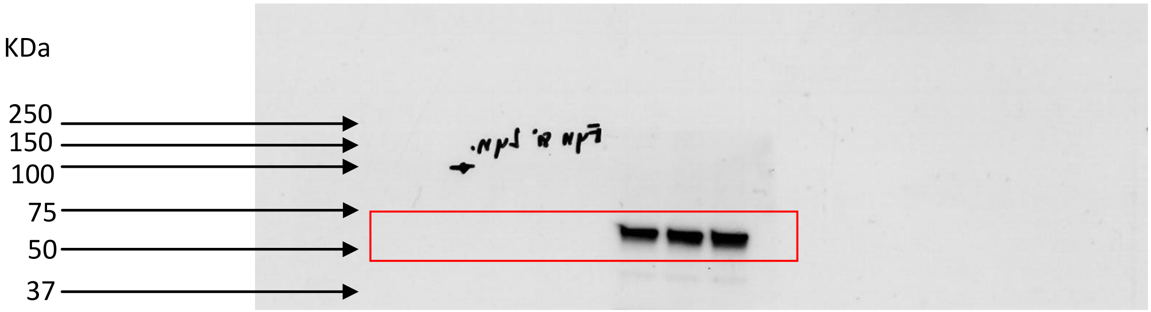

Figure 2a

IB: Syk, Control siRNA

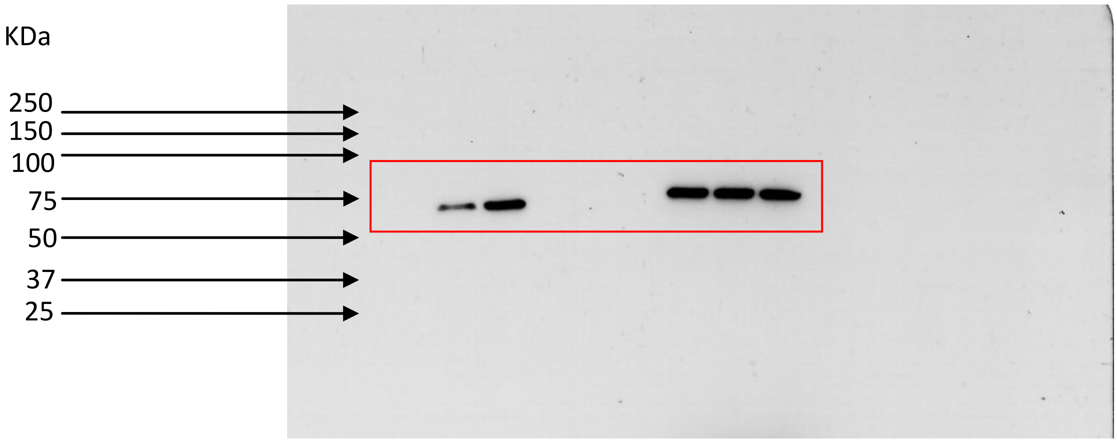

Figure 2a

IB: SHP-1, Control siRNA

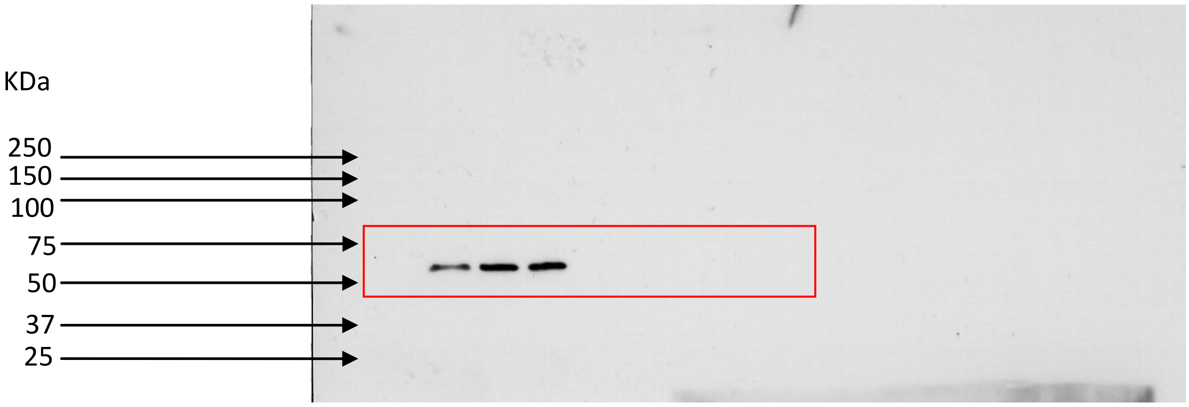

Figure 3b

IB : SHP-1^{S591} (Fyn$^{-/-}$FcγRIIA^{Tg,} ITAMi)

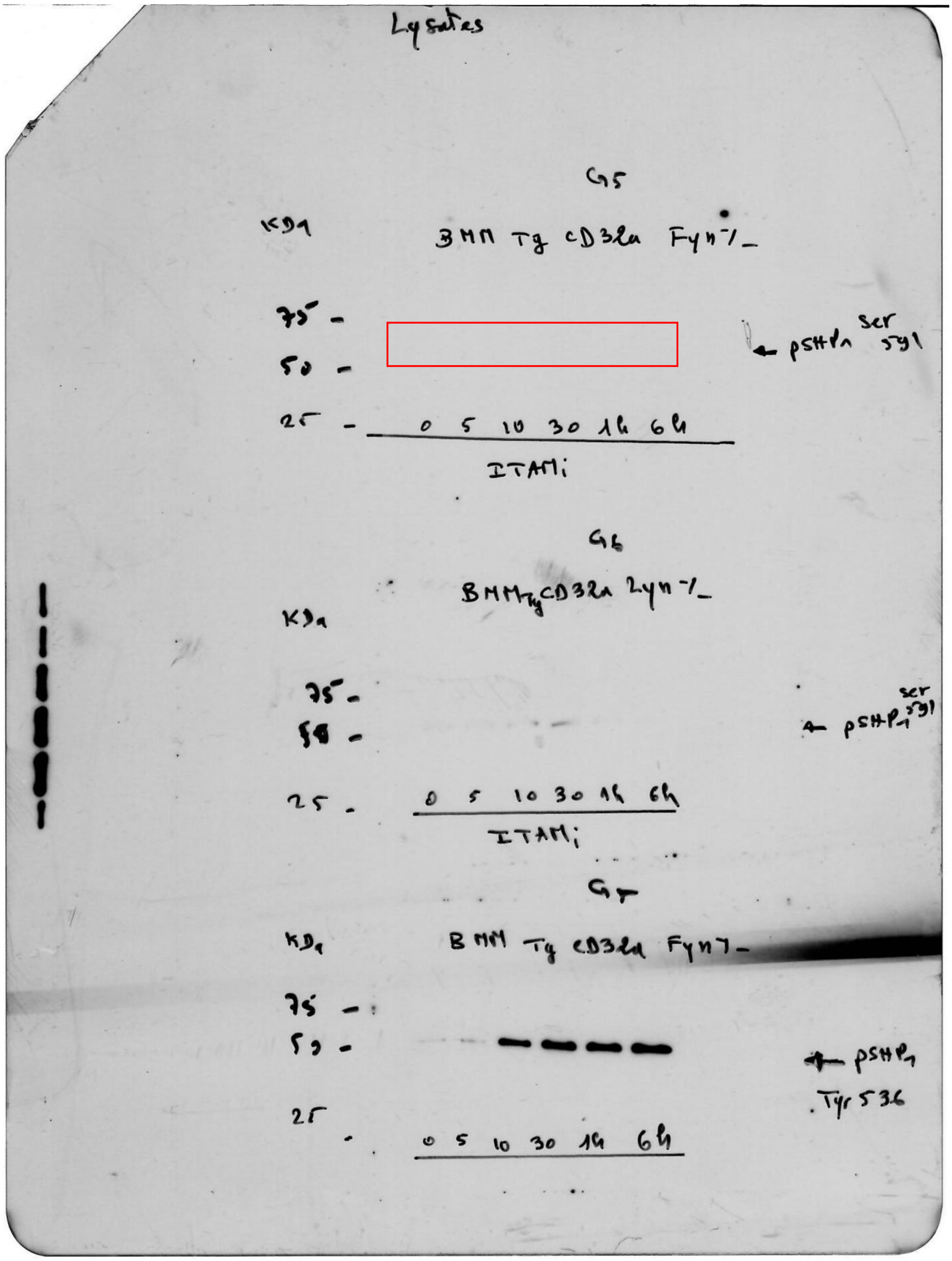

Figure 3b

IB : SHP-1^{S591} (Lyn$^{-/-}$FcγRIIA^{Tg}, ITAMi)

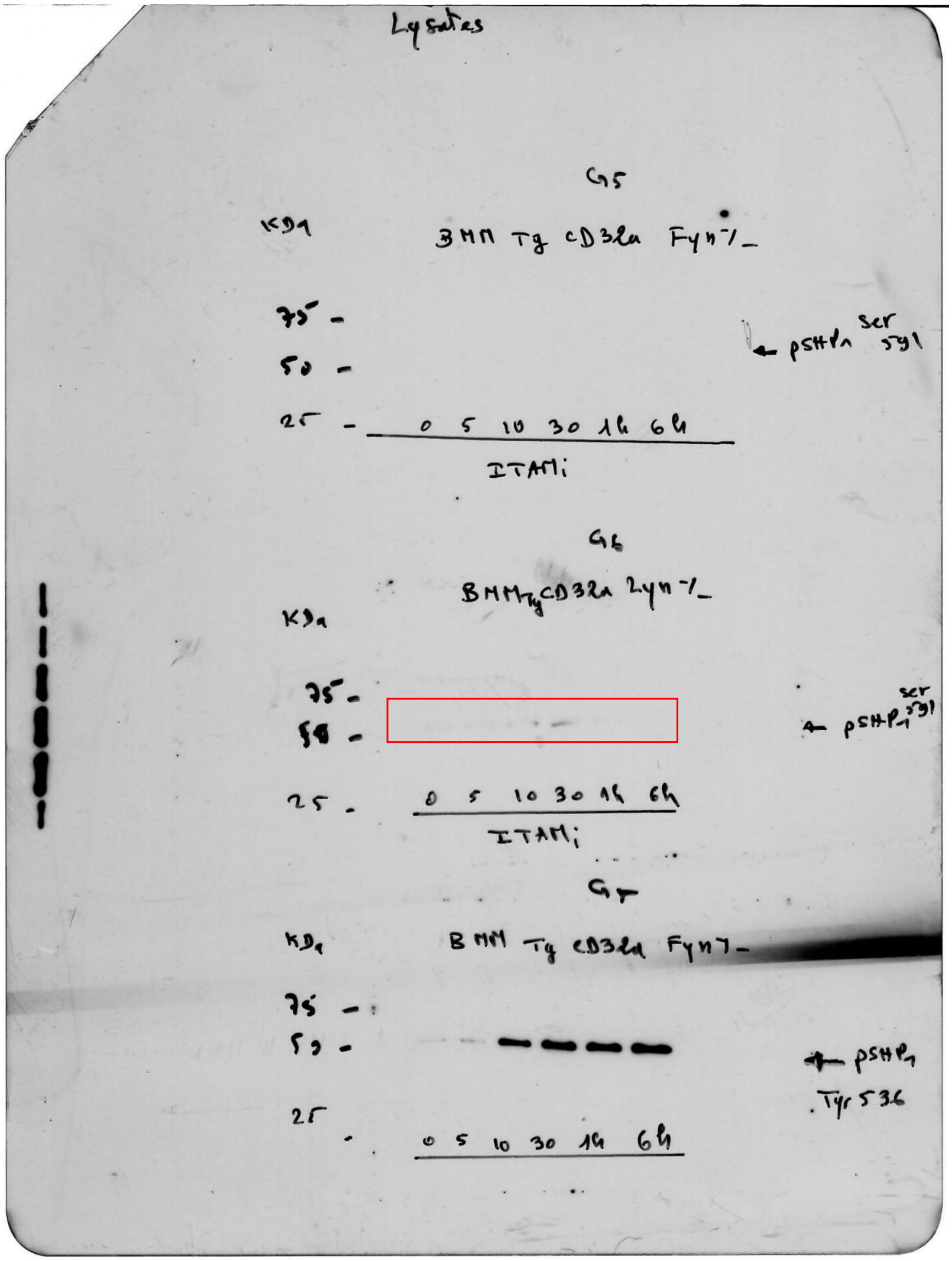

Figure 3b

IB : SHP-1^{Y536} (Fyn$^{-/-}$FcγRIIA^{Tg}, ITAM)

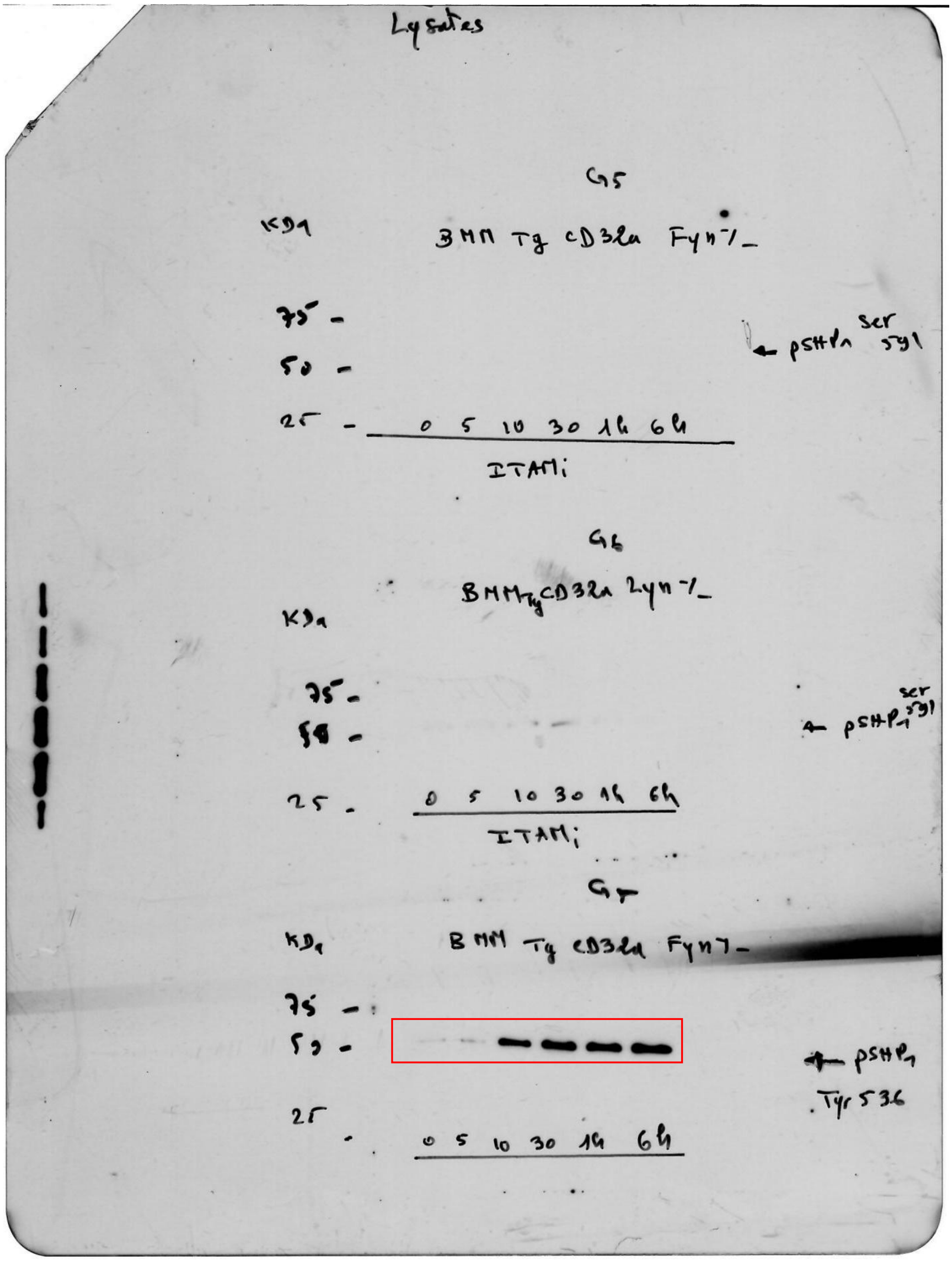

Figure 3b

IB : SHP-1^{S591} (FcγRIIA^{Tg}, ITAMi)

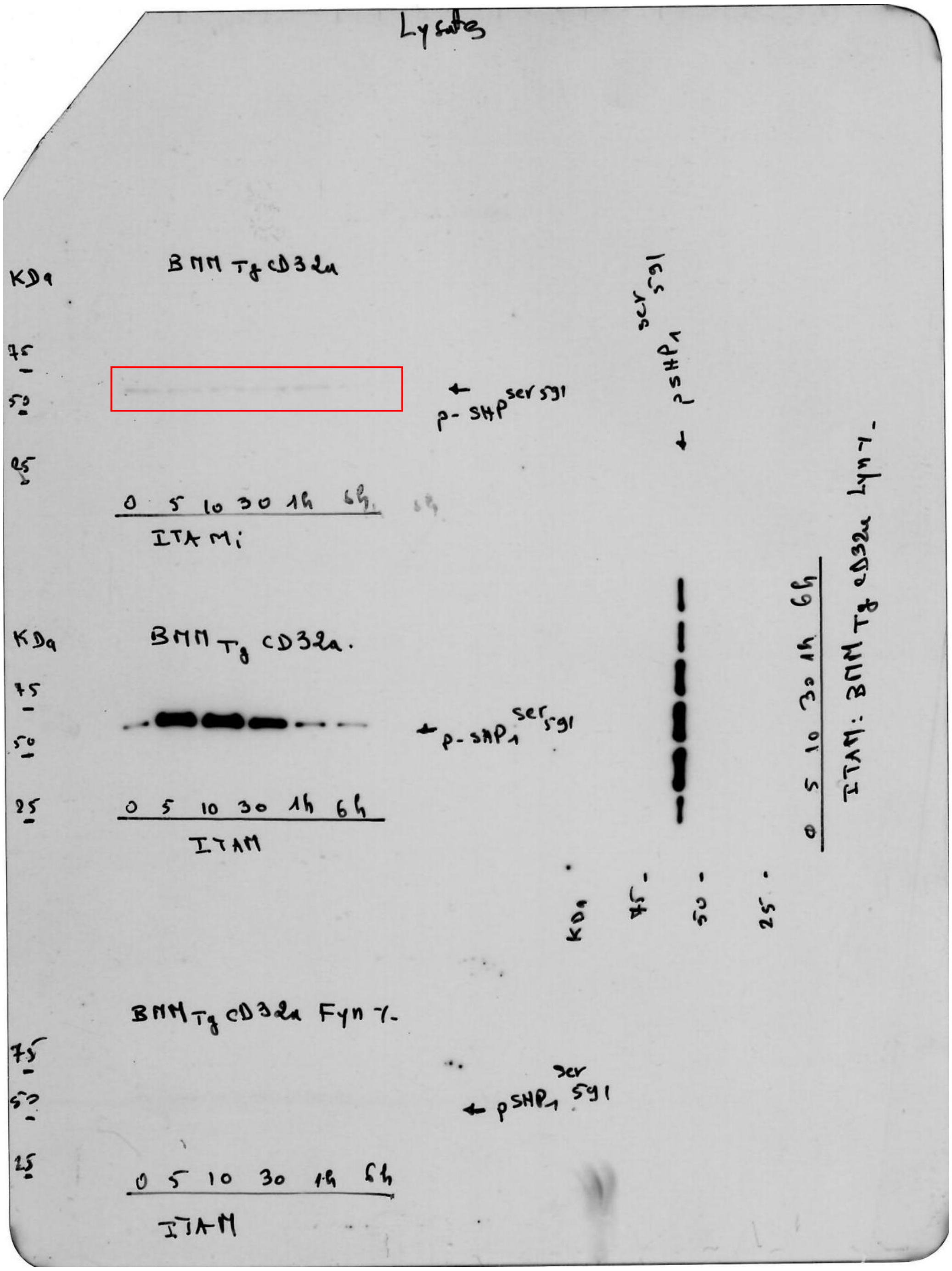

Figure 3b

IB : SHP-1^{Y591} (FcγRIIA^{Tg}, ITAM)

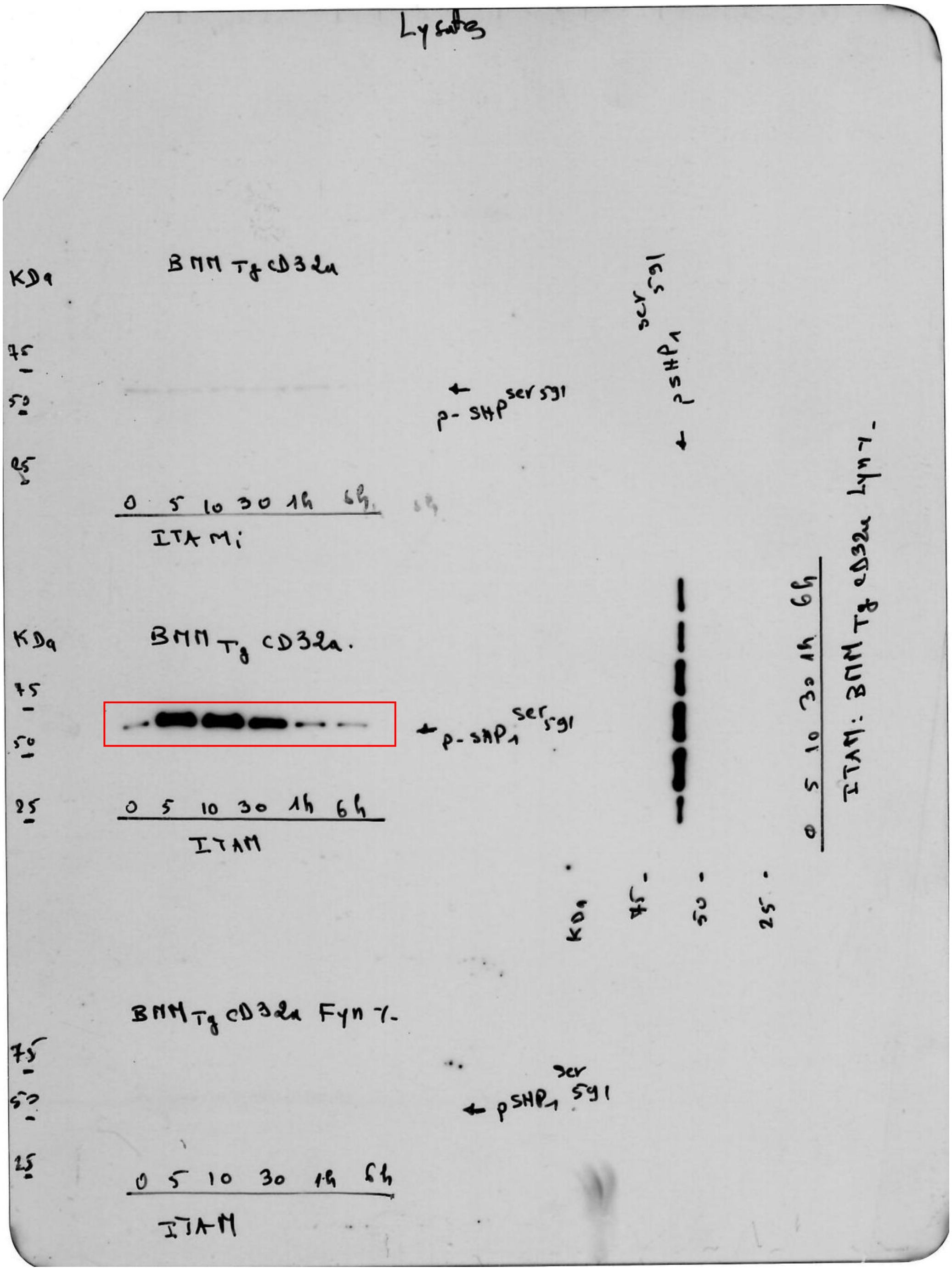

Figure 3b

IB : SHP-1^{Y536} (Fyn$^{-/-}$FcγRIIA^{Tg}, ITAM)

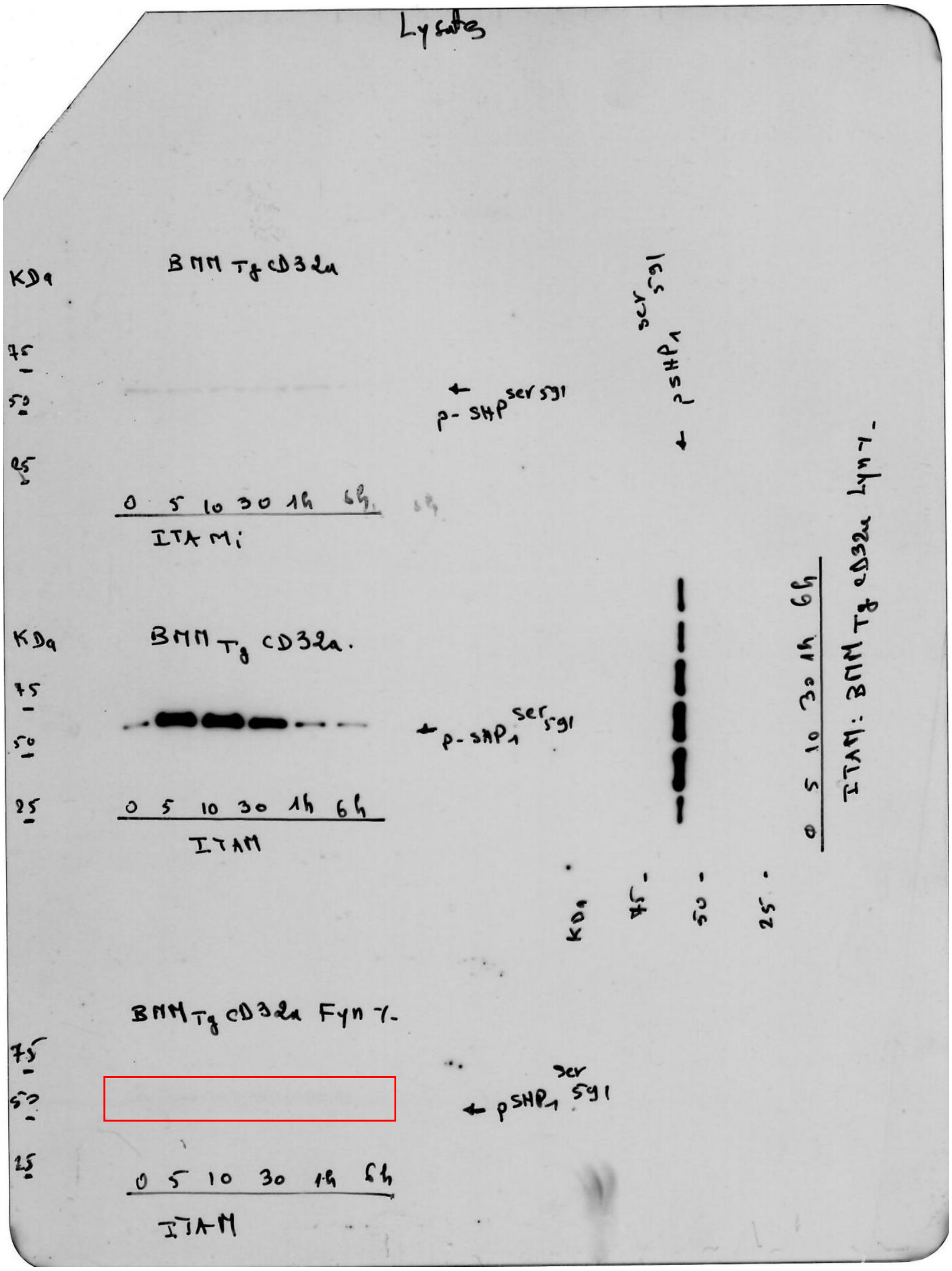

Figure 3b

IB : SHP-1^{S591} (Lyn$^{-/-}$FcγRIIA^{Tg}, ITAM)

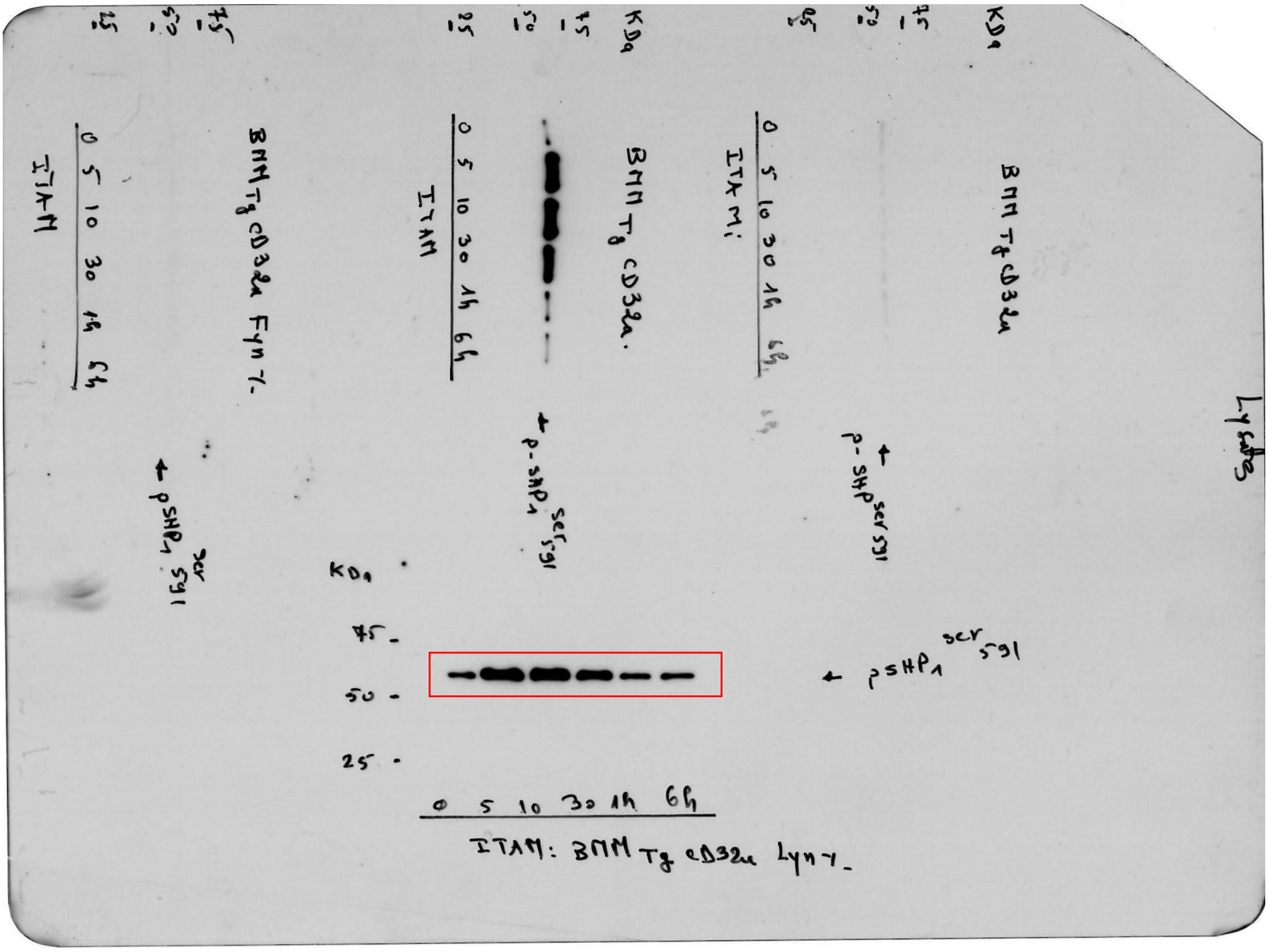

Figure 3b

IB : SHP-1(FcγRIIA^{Tg}, ITAMi)

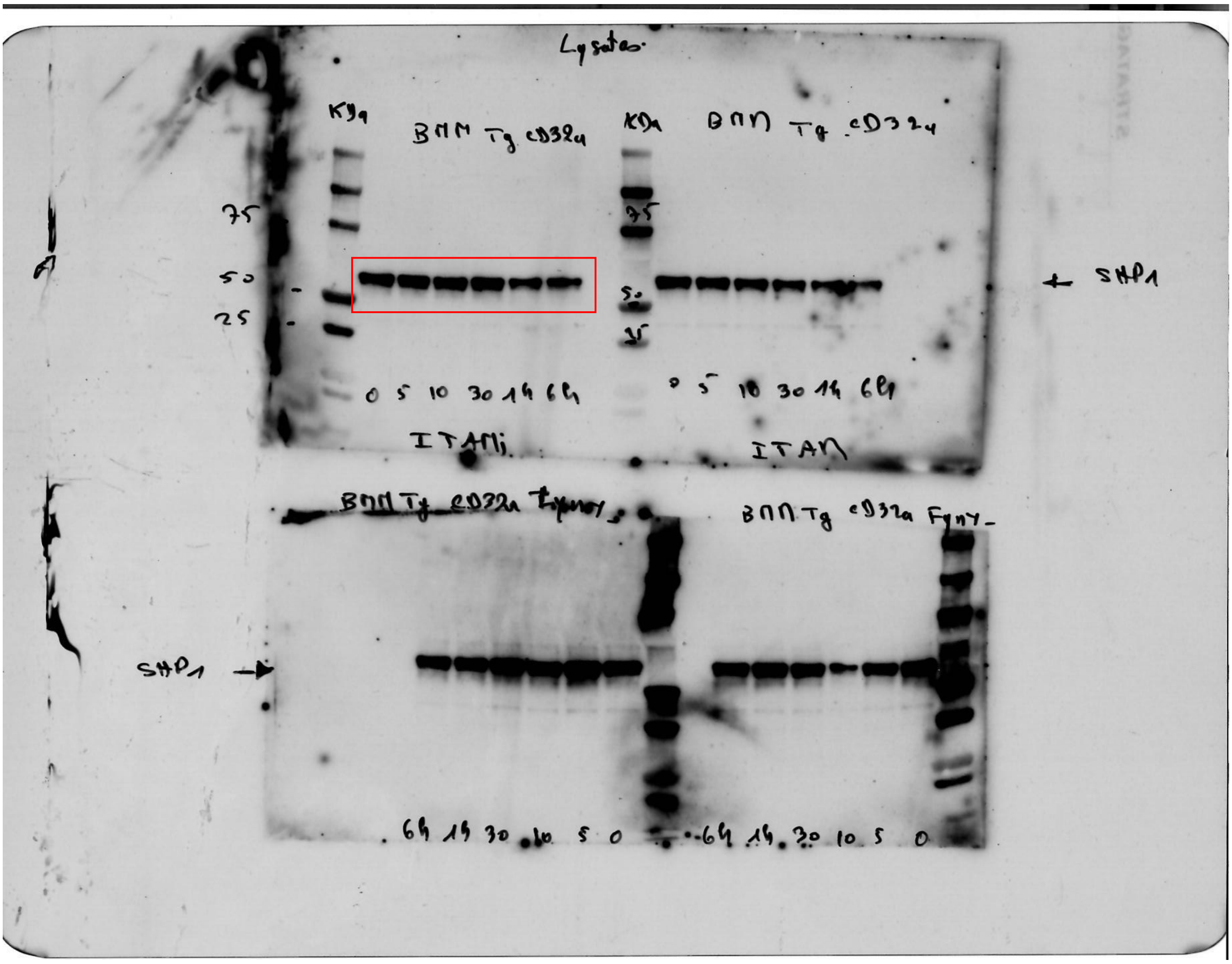

Figure 3b

IB : SHP-1(FcγRIIA^{Tg}, ITAM)

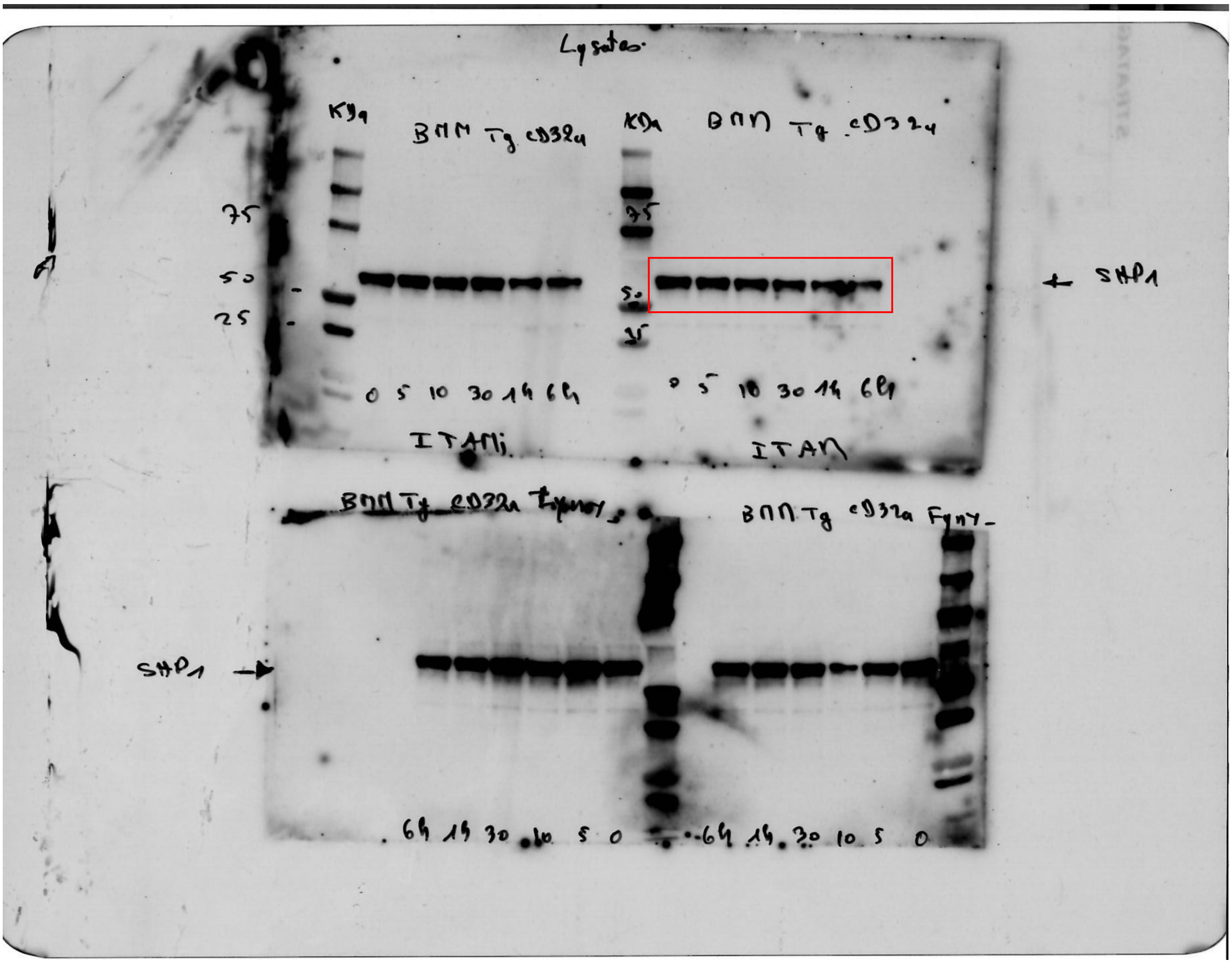

Figure 3b

IB : SHP-1^{Y536} (FcγRIIA^{Tg}, ITAMi)

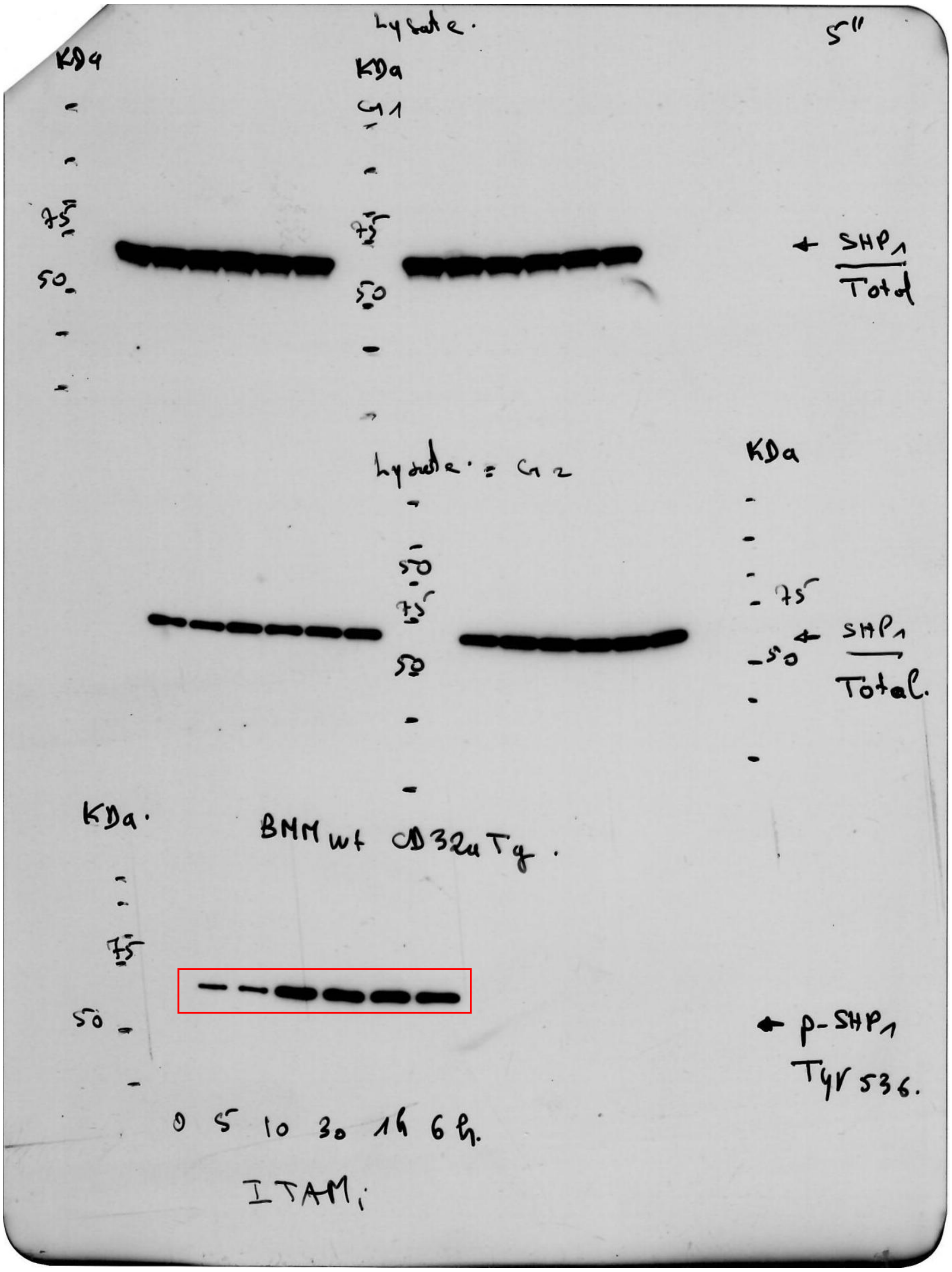

Figure 3b

IB : SHP-1 (Fyn$^{-/-}$FcγRIIA^{Tg}, ITAM)

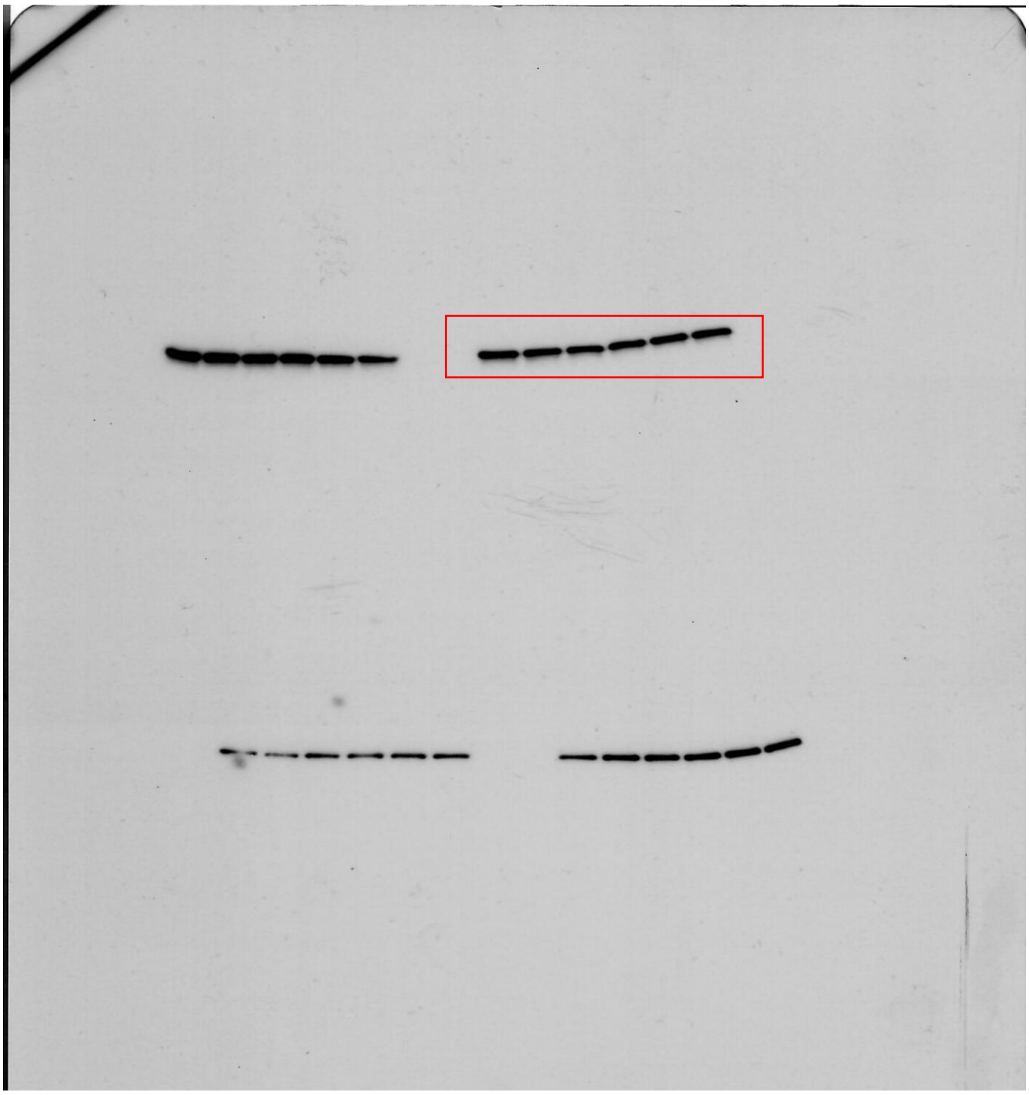

Figure 3b

IB : SHP-1 (Fyn$^{-/-}$FcγRIIA^{Tg}, ITAMi)

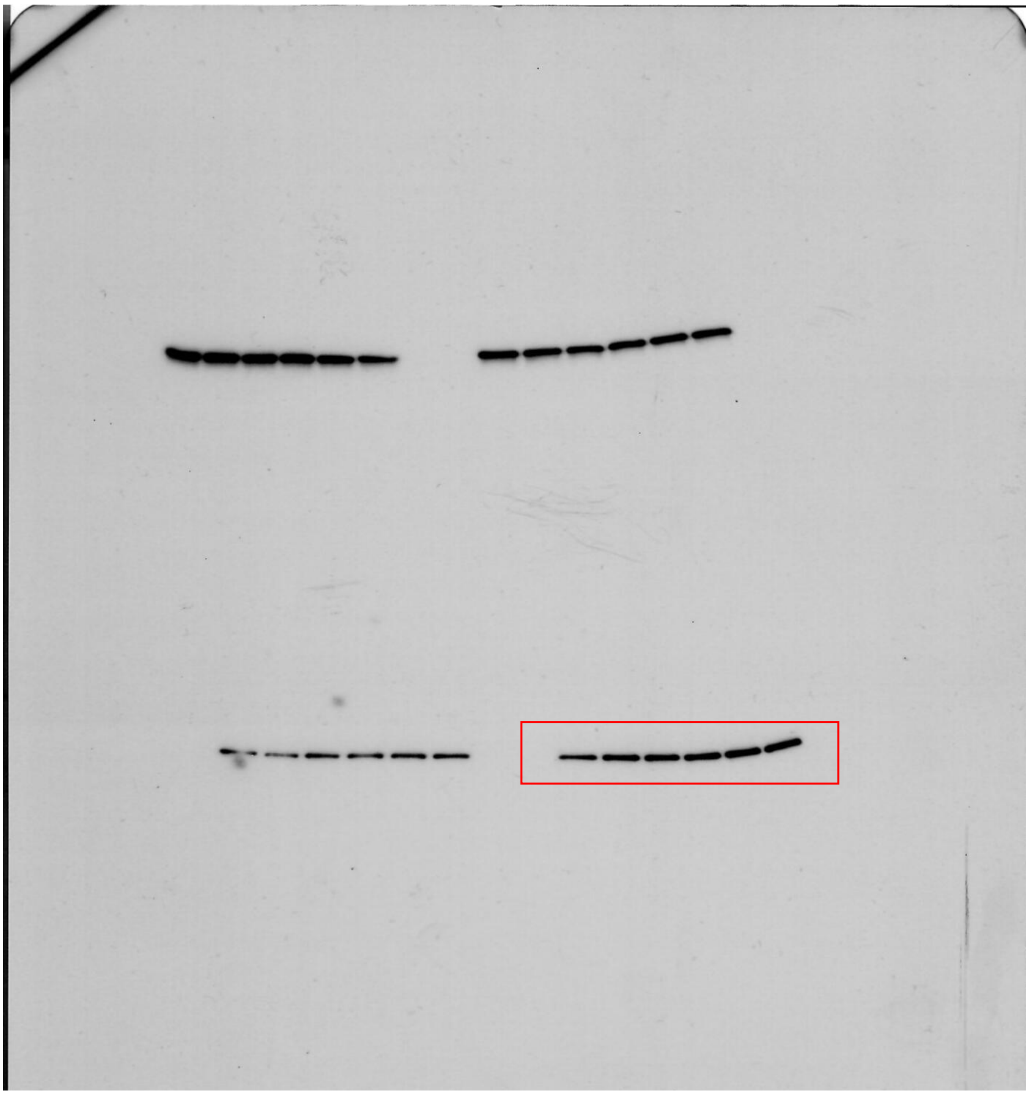

Figure 3b

IB : SHP-1^{Y536} (Lyn$^{-/-}$FcγRIIA^{Tg}, ITAM)

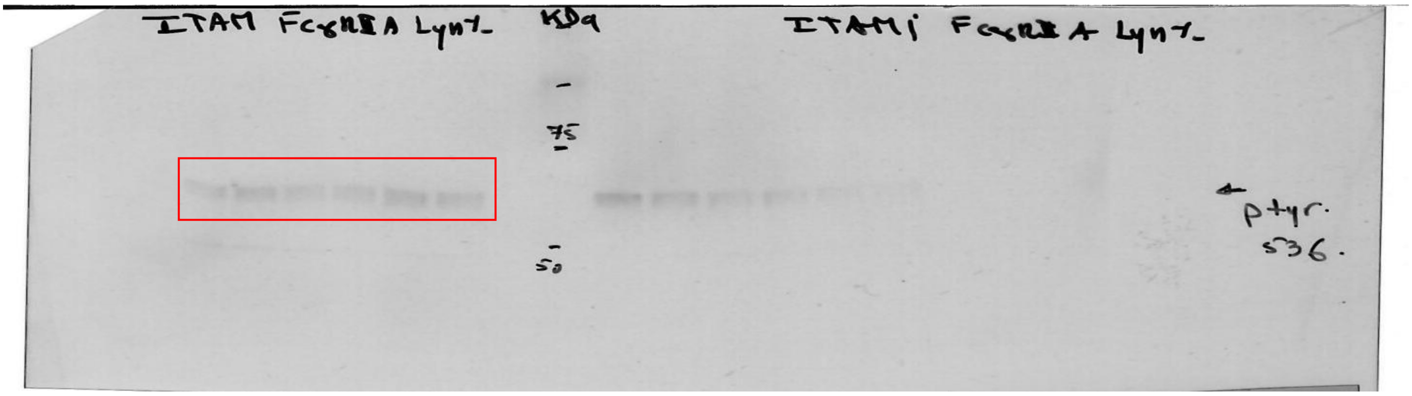

Figure 3b

IB : SHP-1^{Y536} (Lyn$^{-/-}$FcγRIIA^{Tg}, ITAMi)

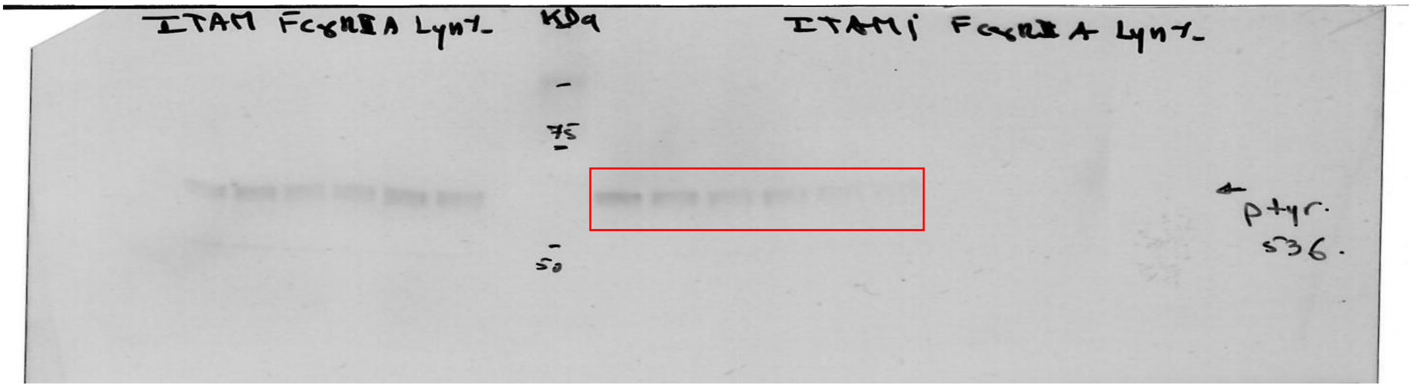

Figure 3b

IB : pSHP-1^{Y536} (FcγRIIA^{Tg}, ITAMi)

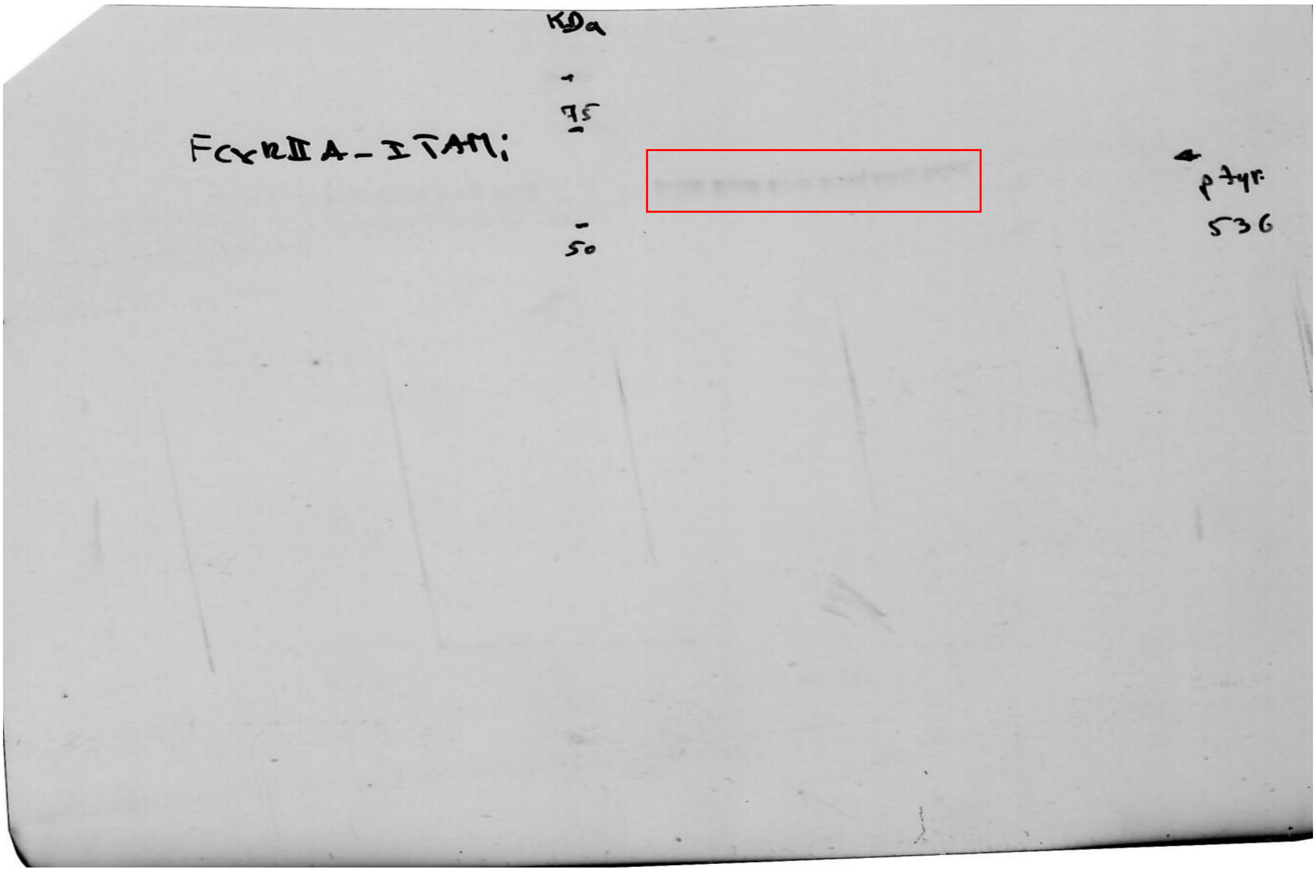

Figure 3C

IB: pSHP ^{S591} (PI3K inhibitor)

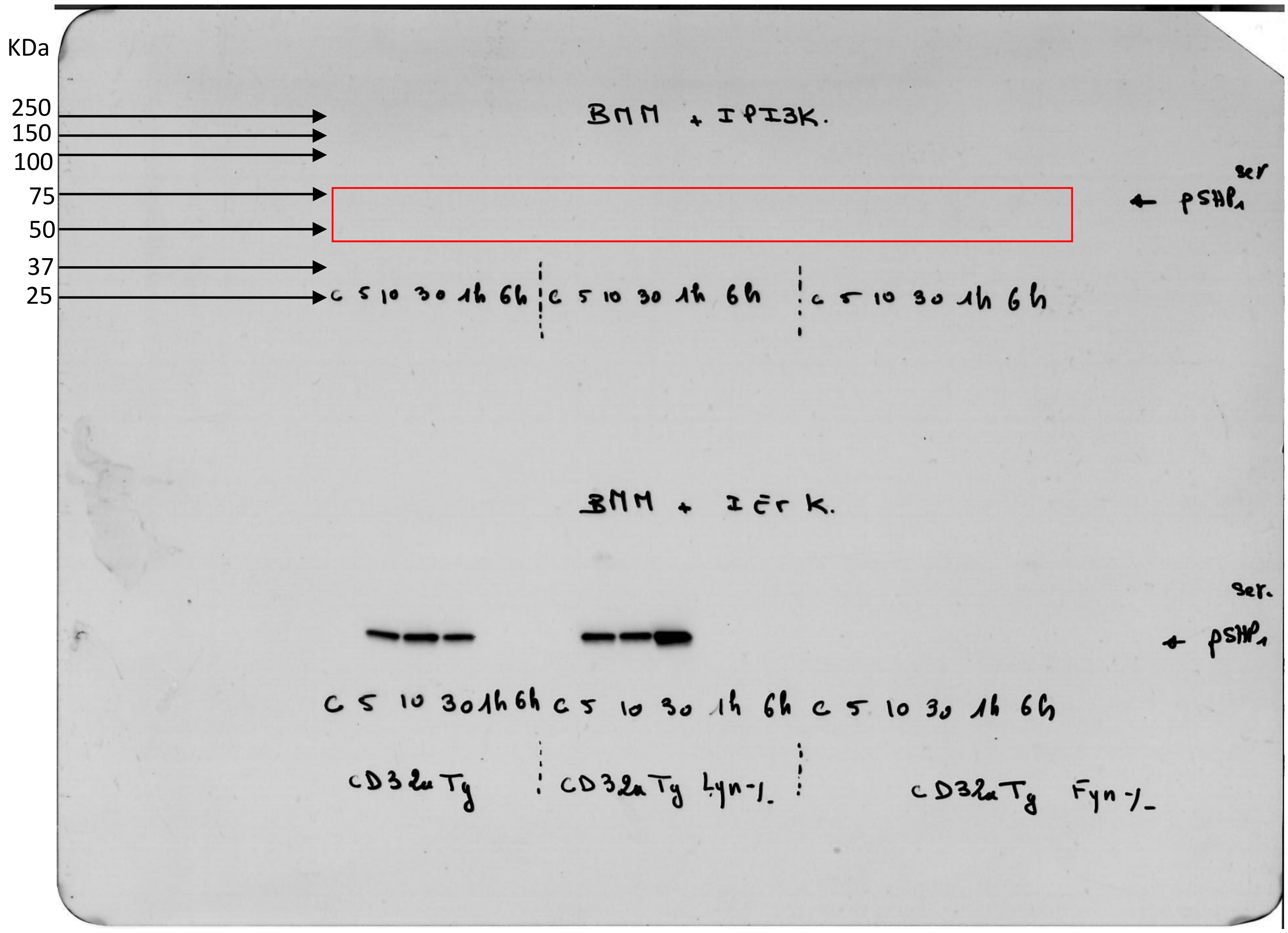

Figure 3C

IB: pSHP^{S591} (ERK inhibitor)

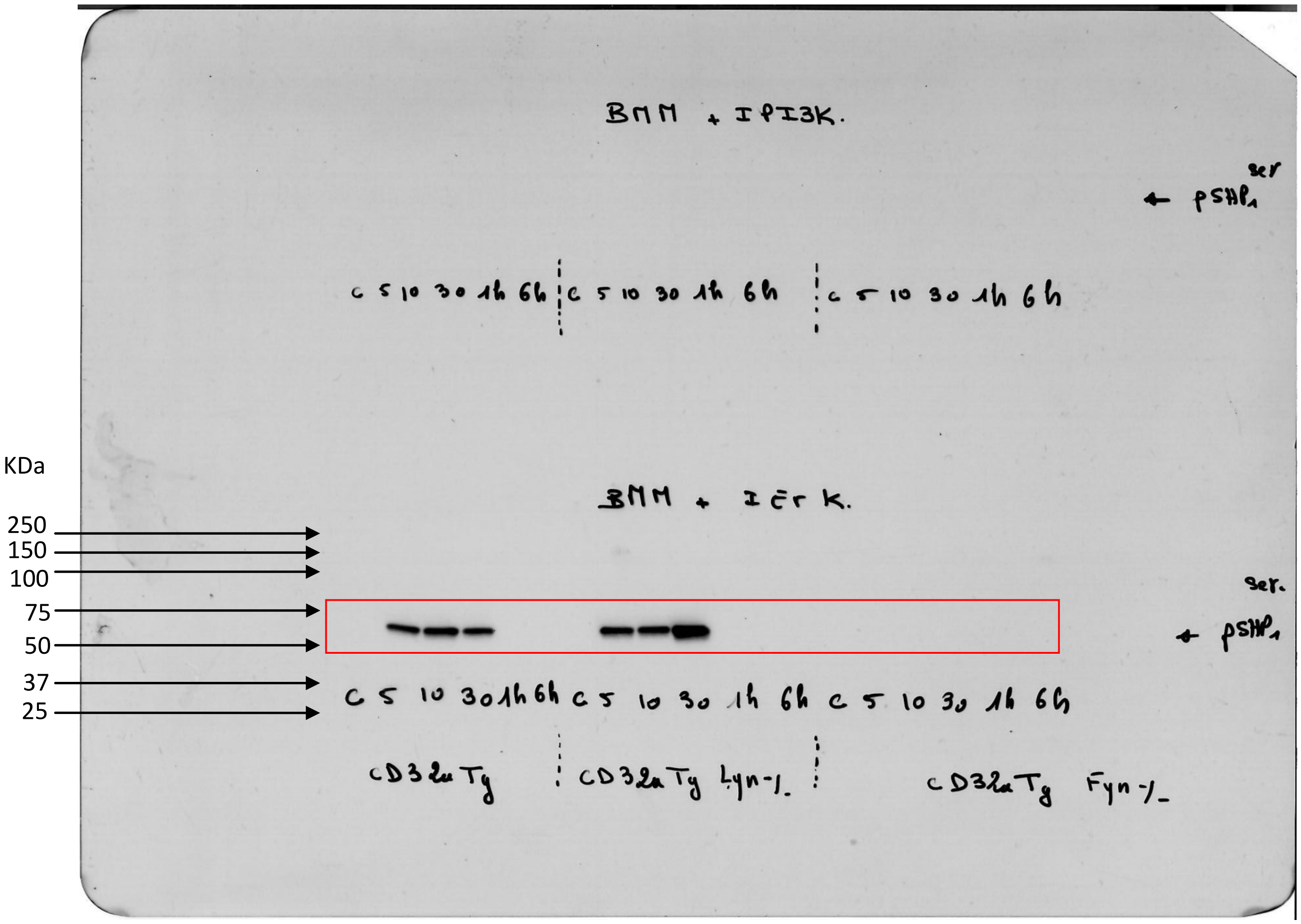

Figure 3c

IB: pAKT (ERK inhibitor)

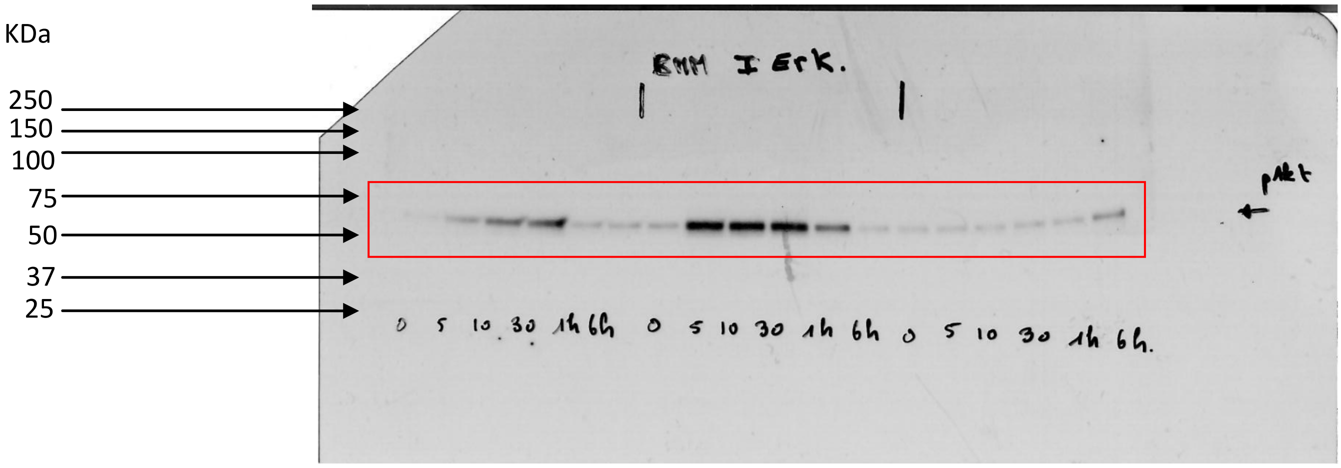

Figure 3c

IB: pAKT (PKC inhibitor)

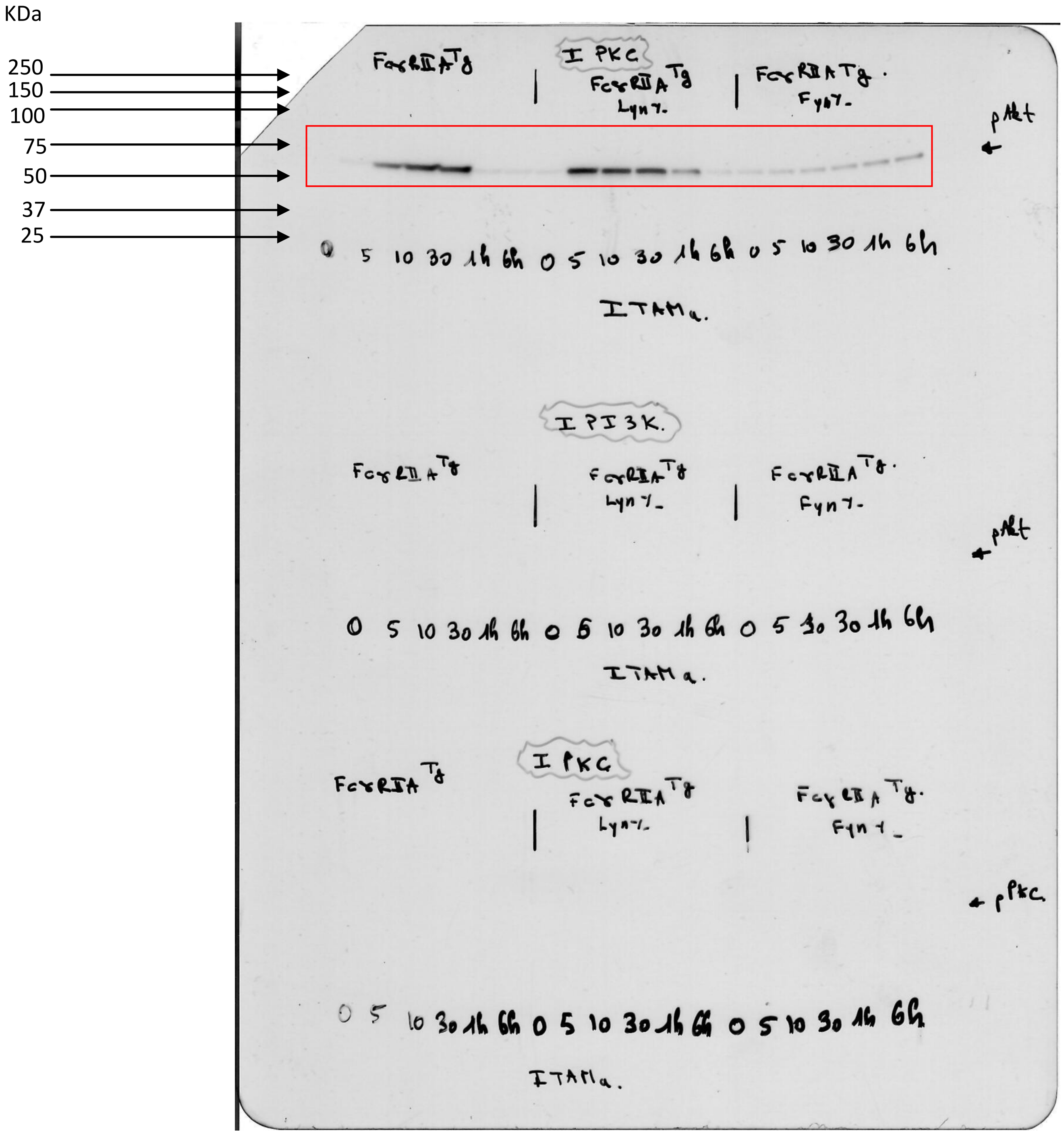

Figure 3c

IB: pAKT (PI3K inhibitor)

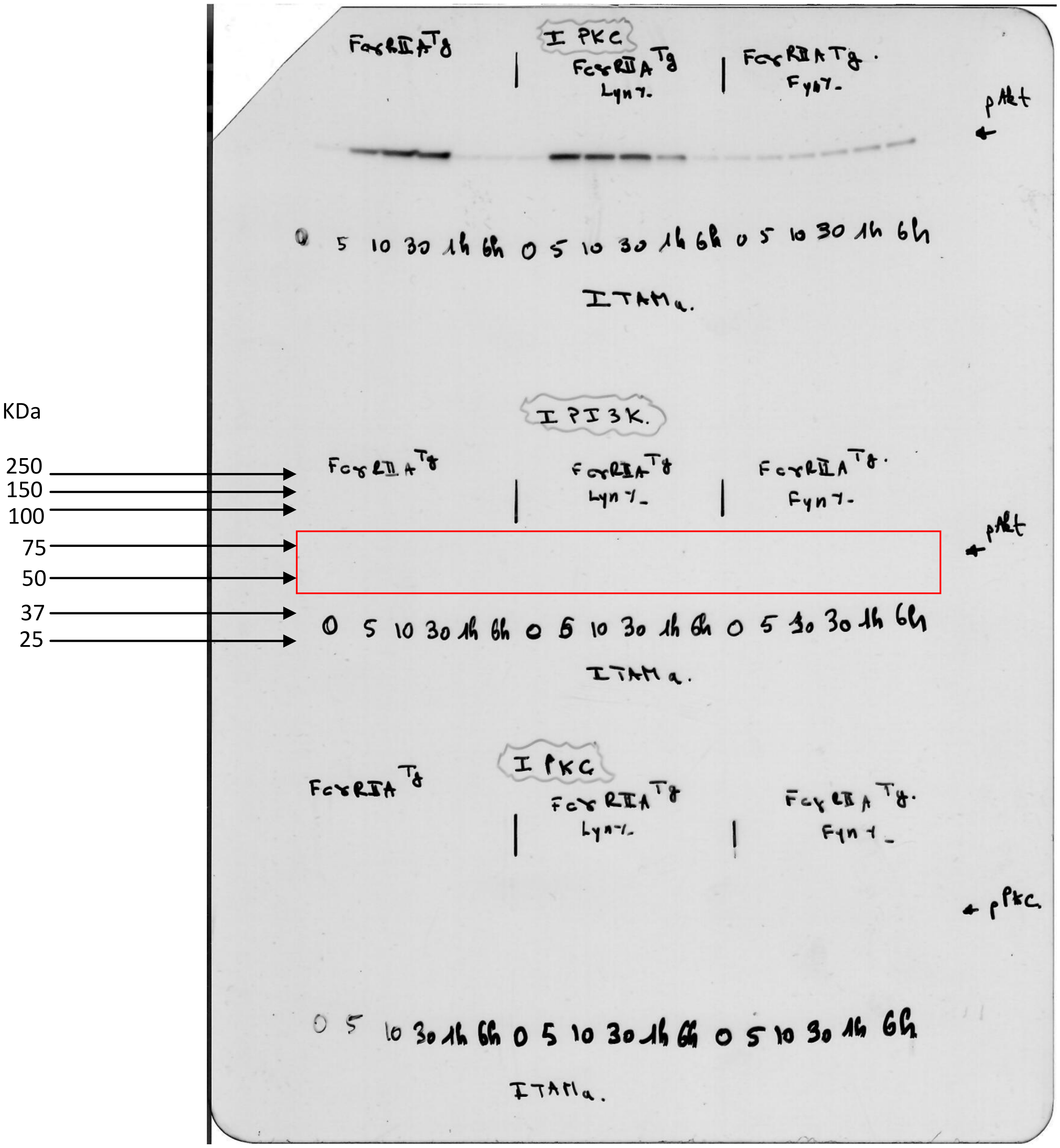

Figure 3c

IB: pPKC (PKC inhibitor)

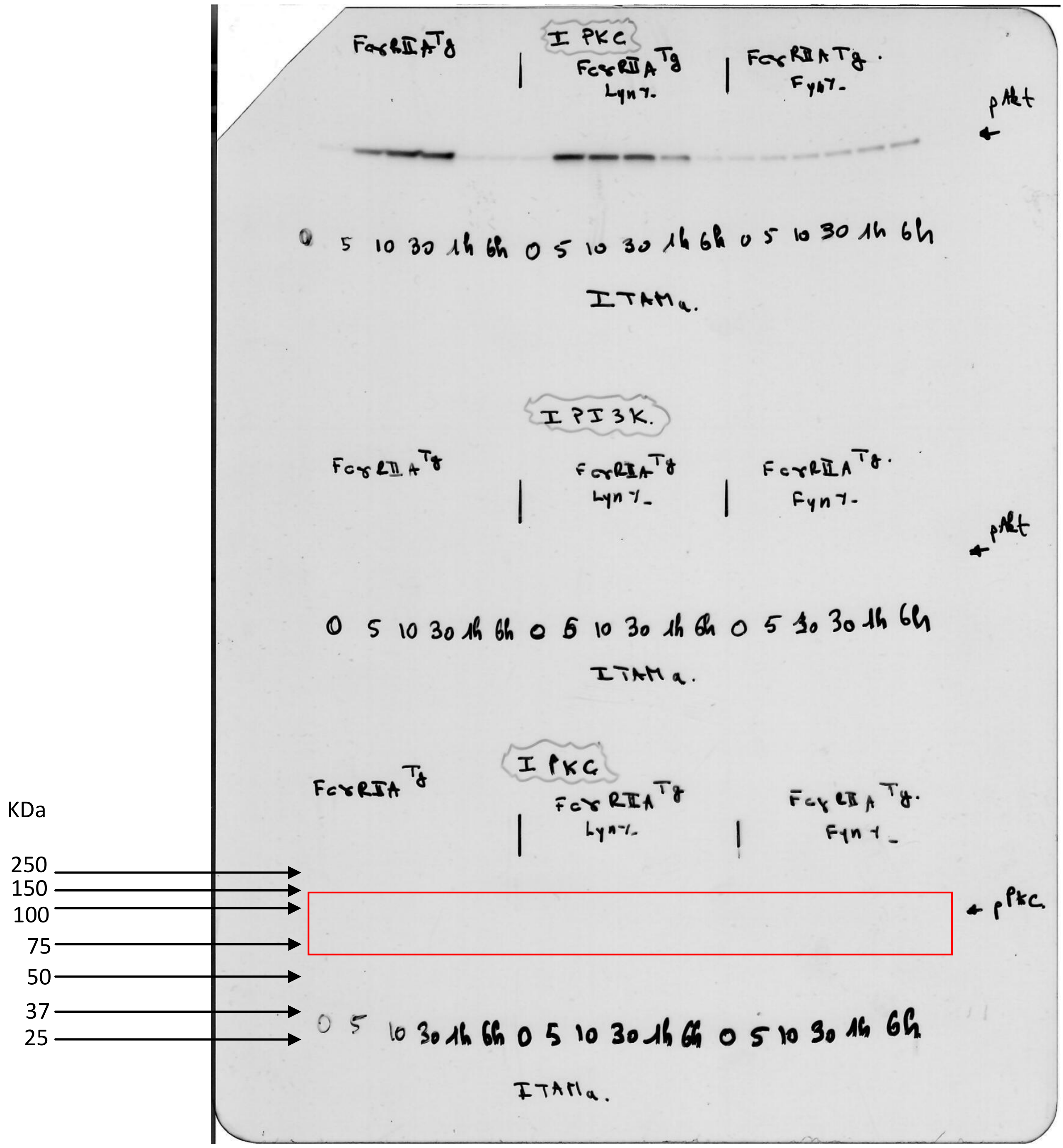

Figure 3c

IB: pERK (PKC inhibitor)

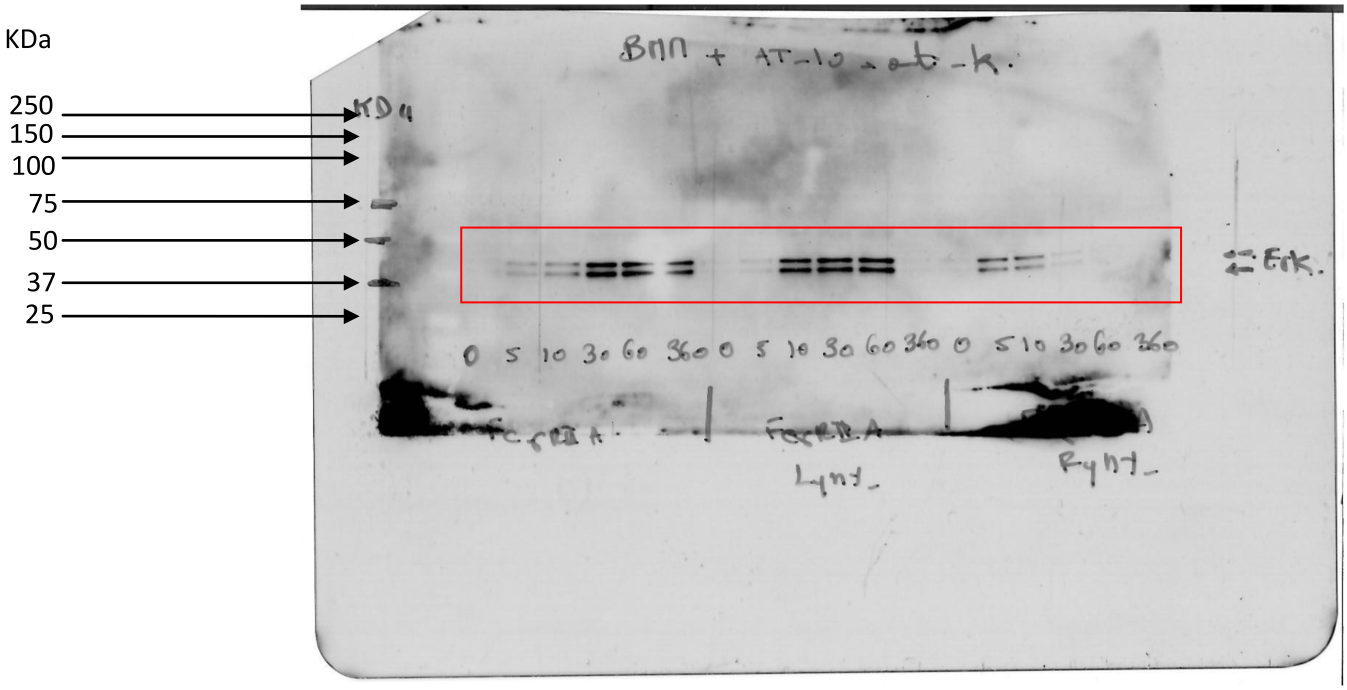

Figure 3c

IB: pSHP-1^{S591} (PKC inhibitor)

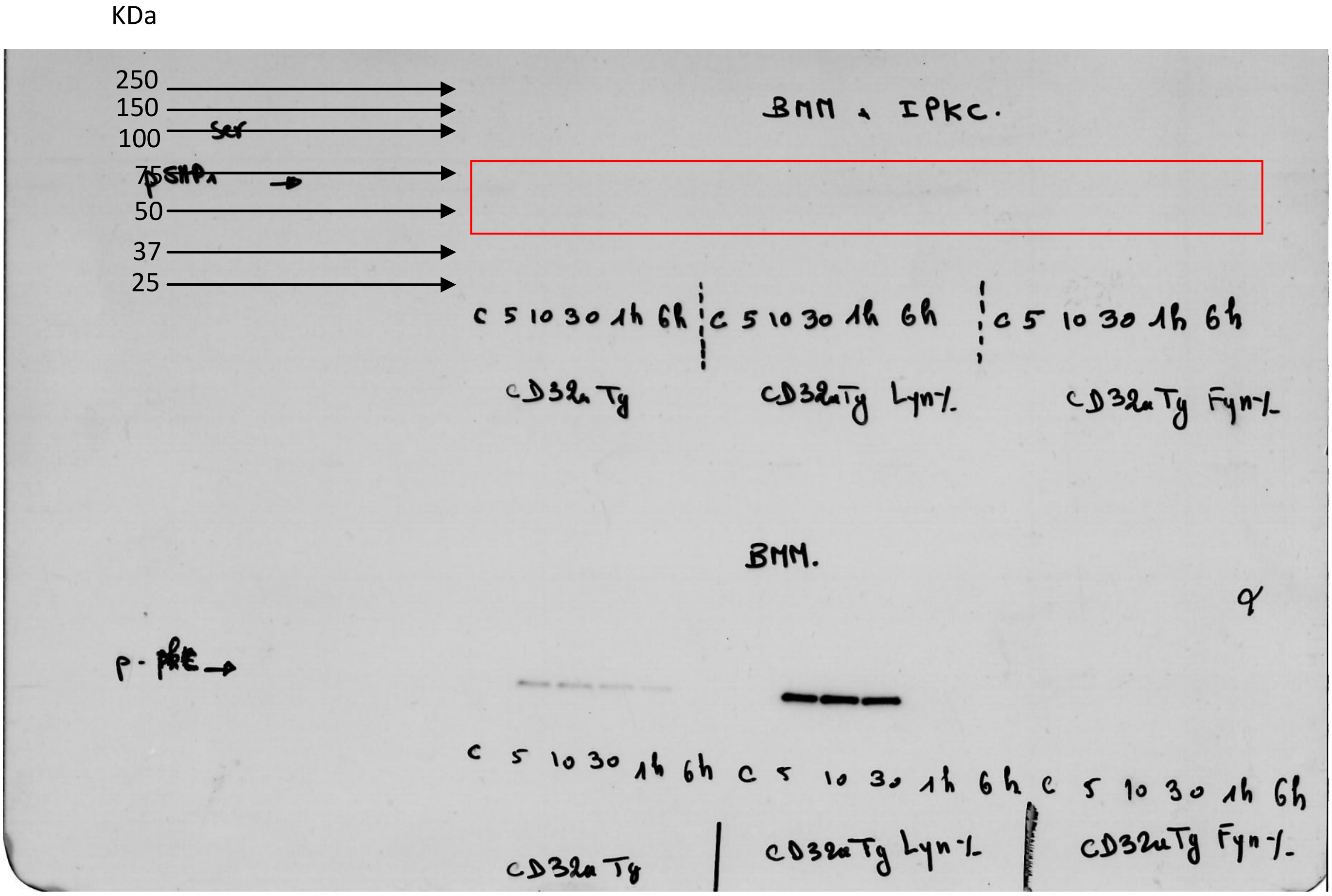

Figure 3c

IB: pPKC (Vehicle)

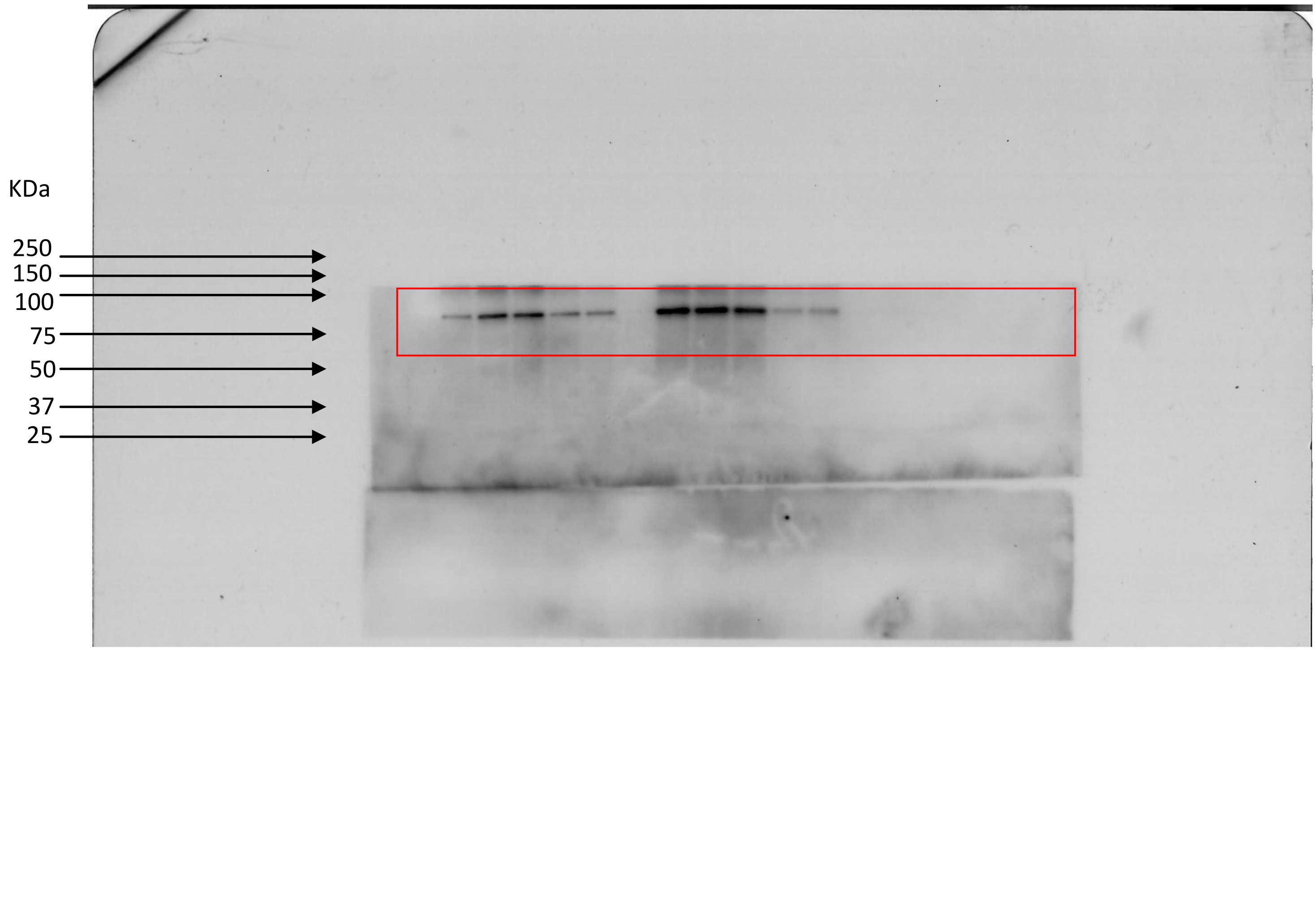

Figure 3c

IB: SHP-1^{S591} (Vehicle)

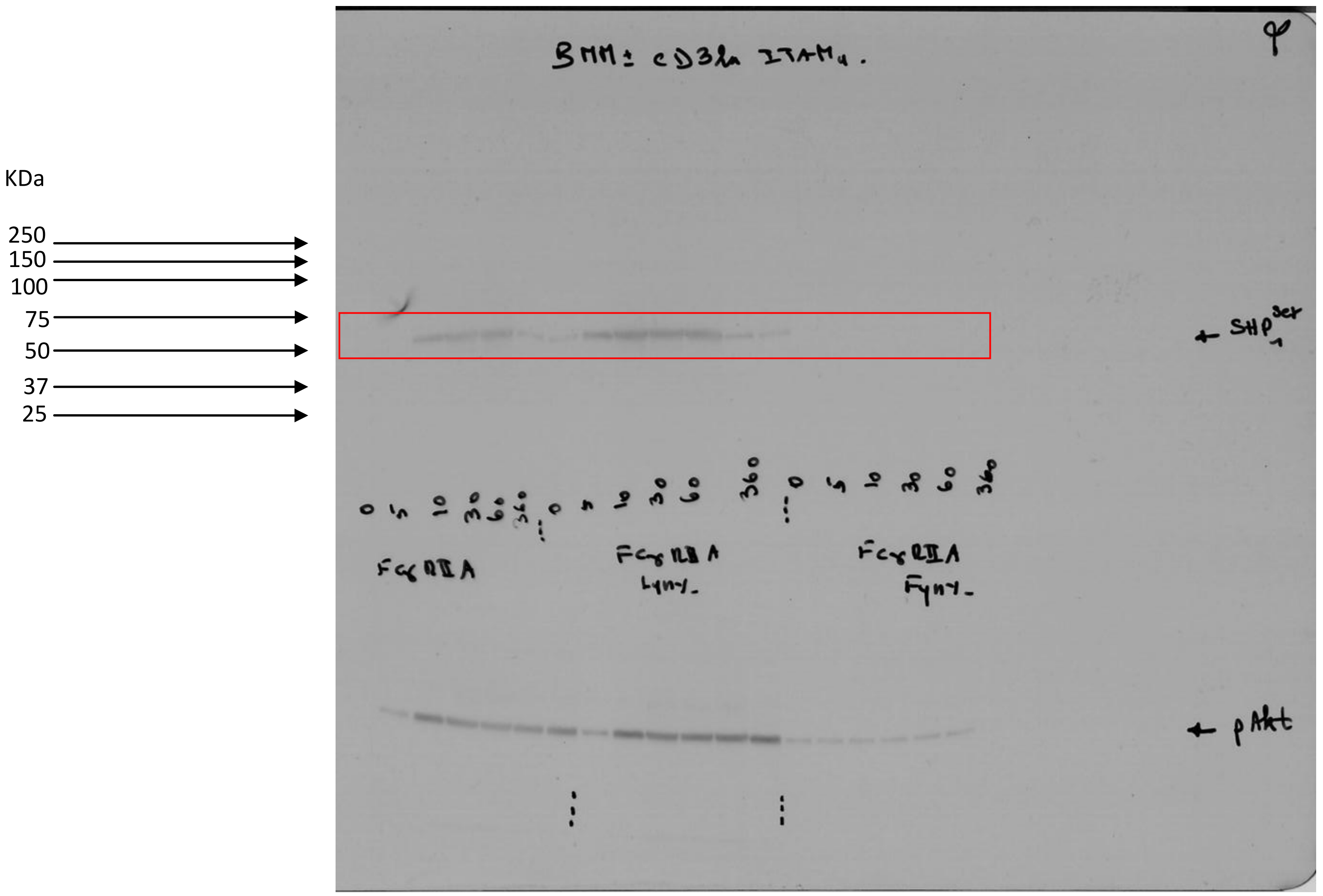

Figure 3c

IB: pAKT (Vehicle)

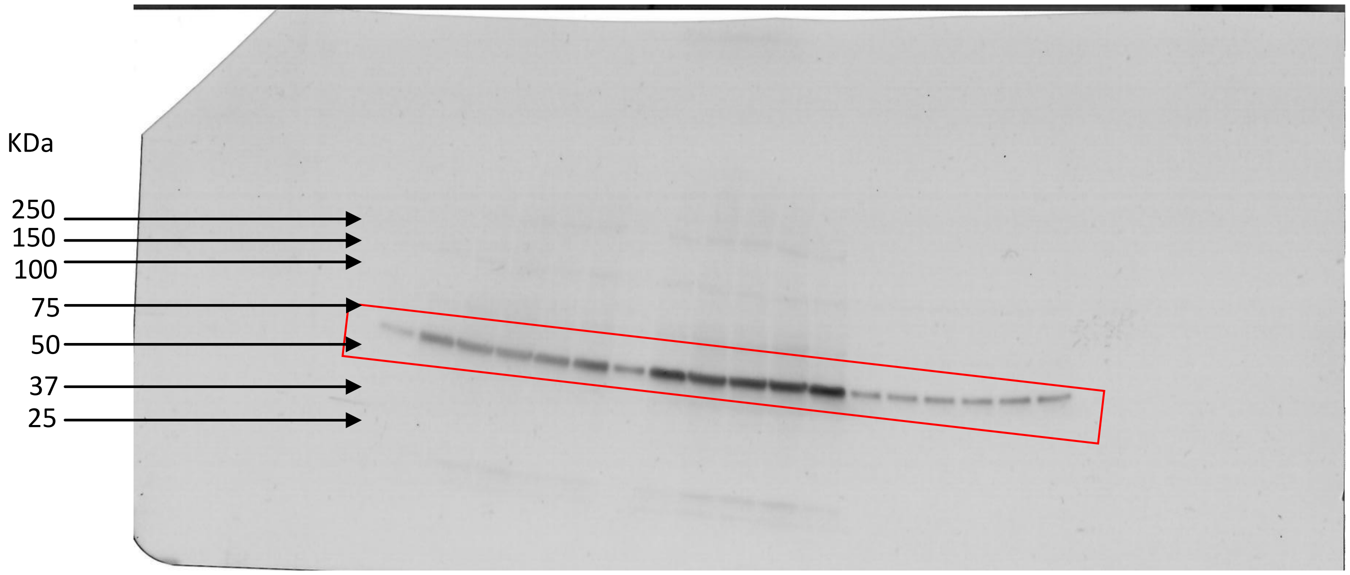

Figure 3c

IB: pPKC (IP3K inhibitor)

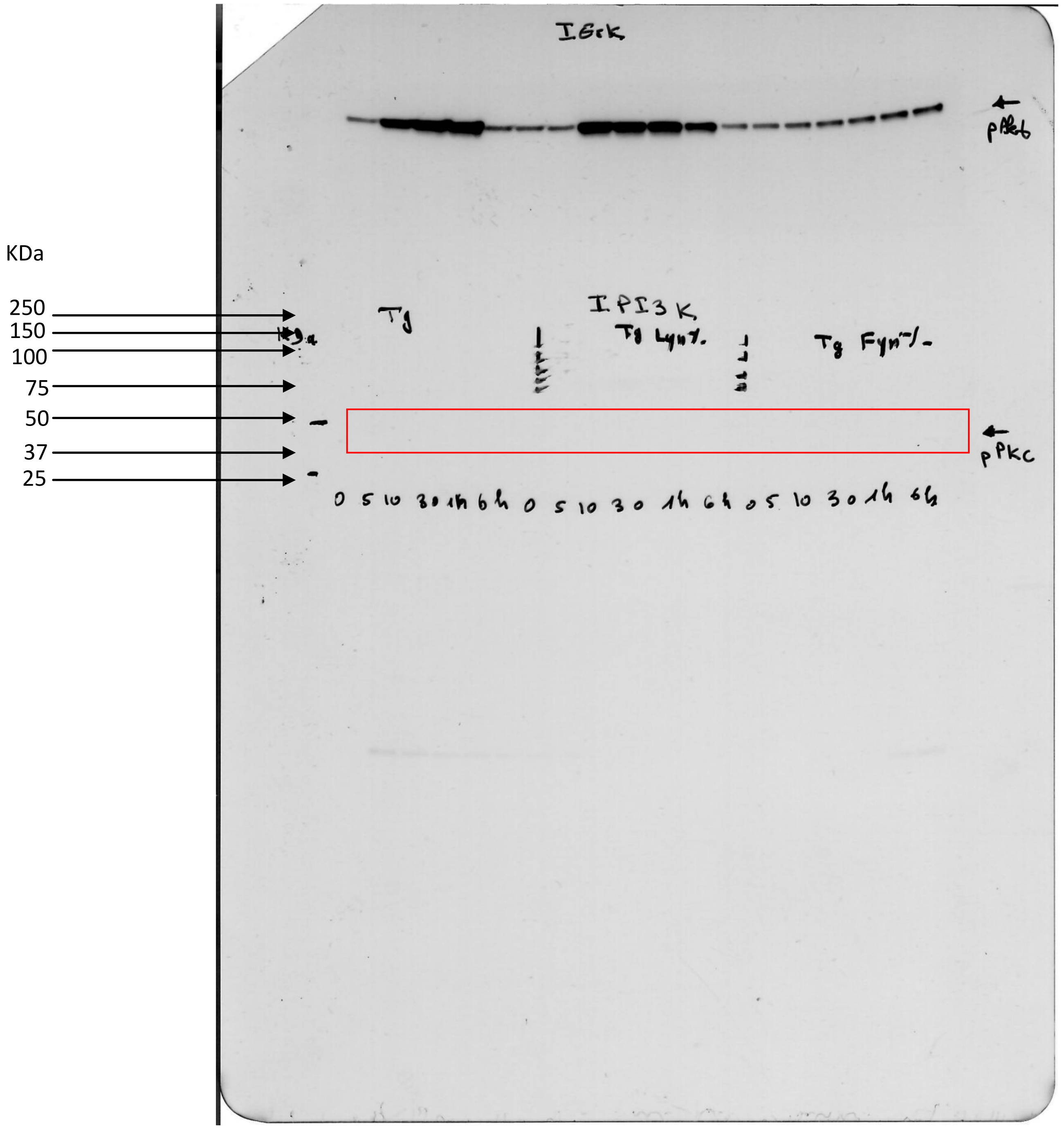

Figure 3c

IB : pPKC (ERK inhibitor)

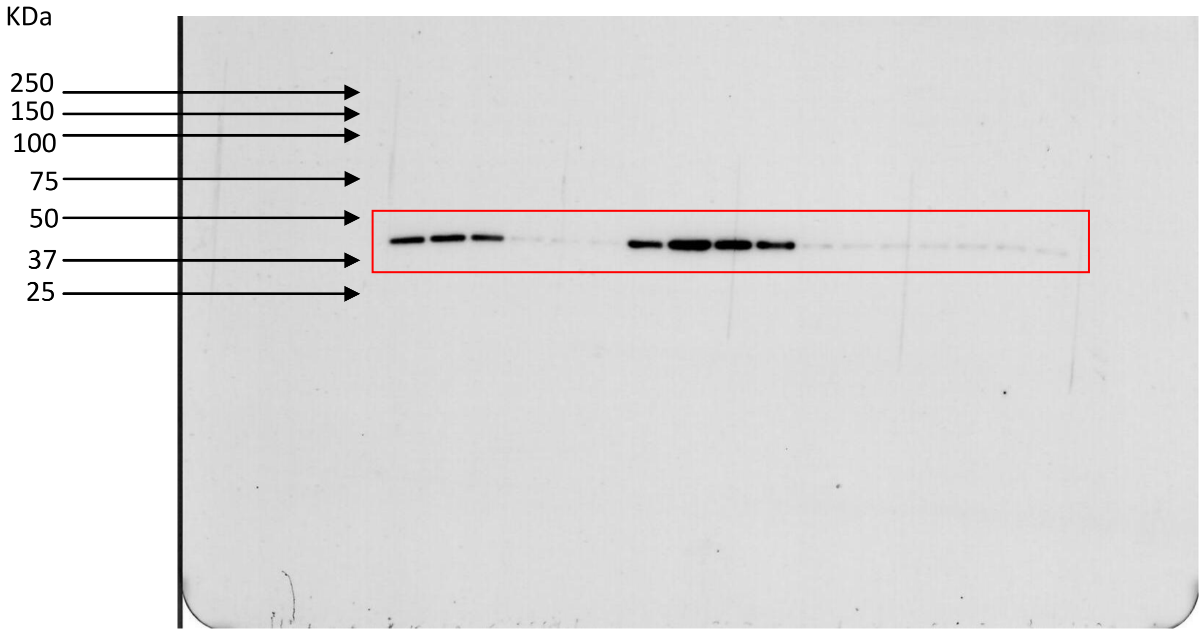

Figure 3c

IB: pAKT (PKC inhibitor)

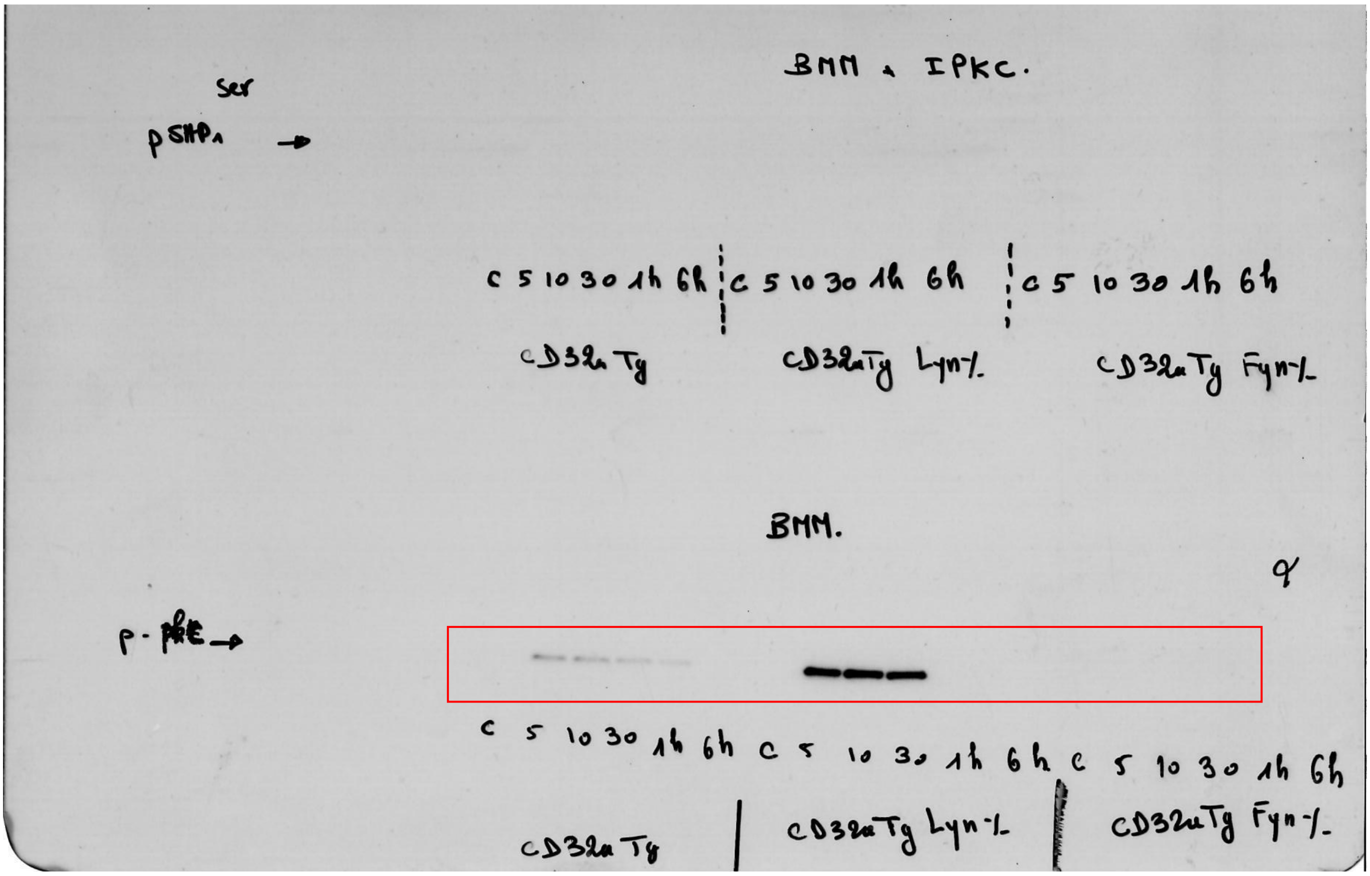

Figure 3c

IB: pERK (PI3K inhibitor)

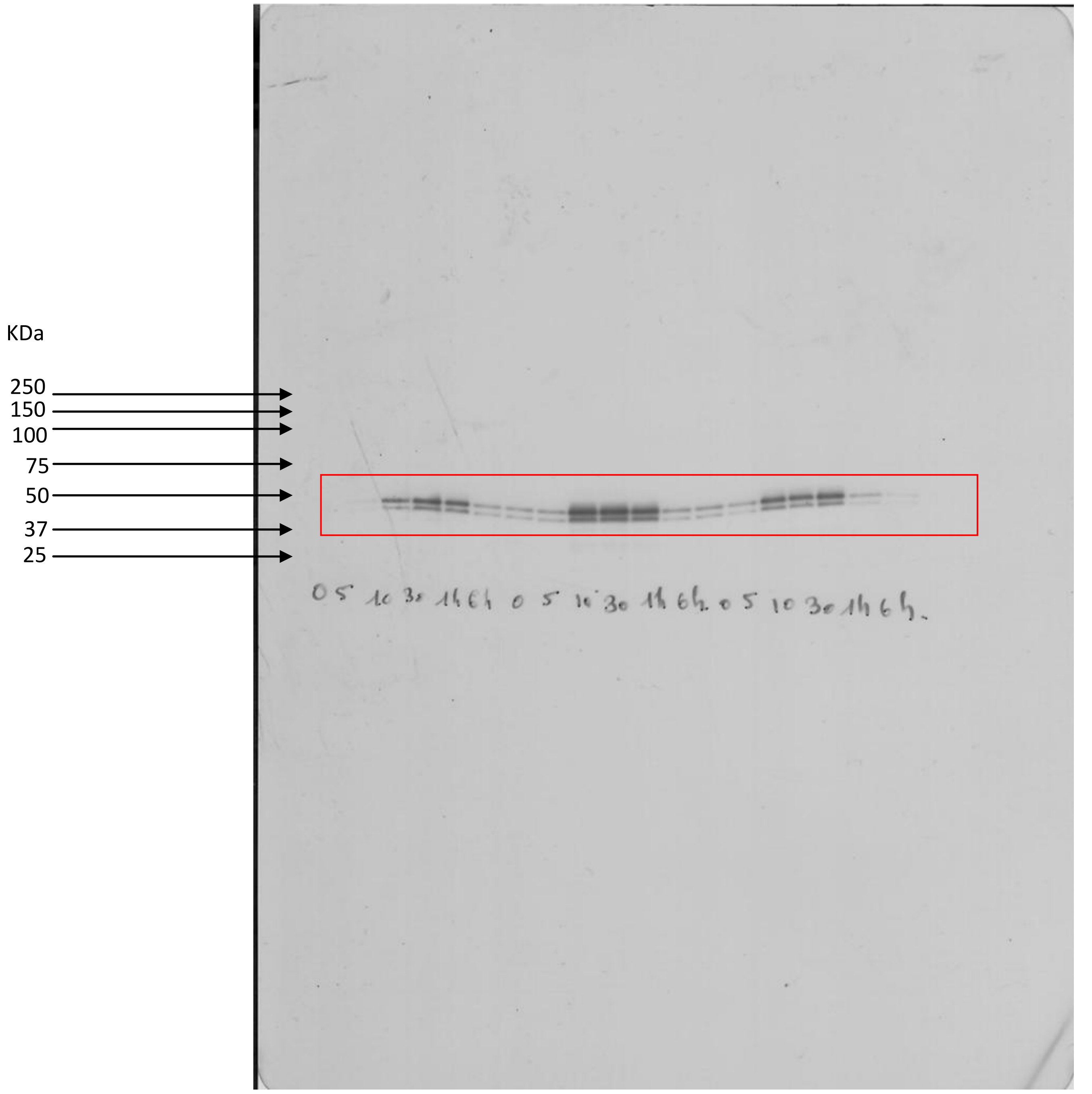

Figure 3c

IB: pERK (Vehicle)

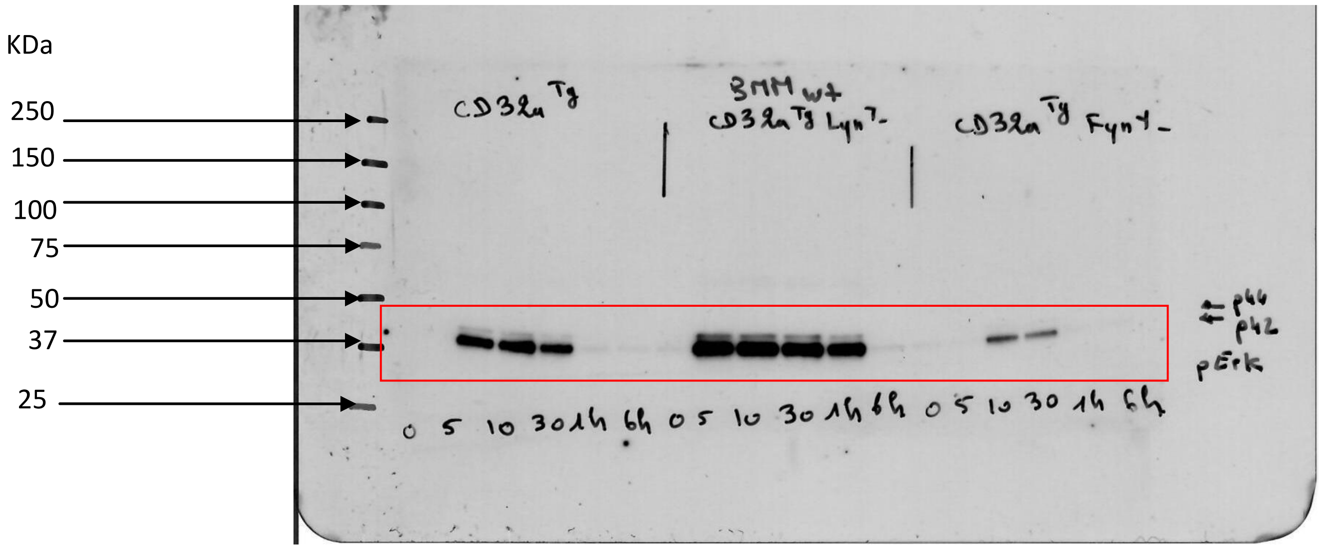

Figure 3c

IB: pSHP-1^{Y536} (Vehicle)

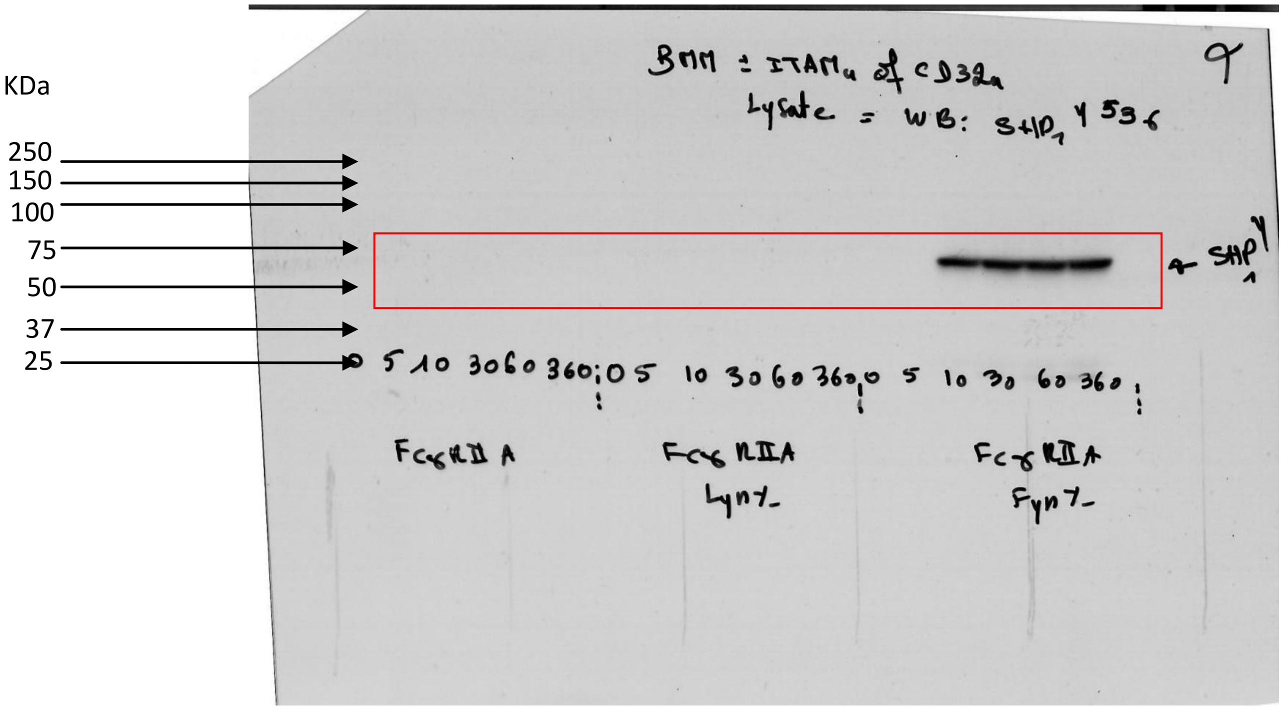

Figure 3c

IB : SHP-1^{Y536} (PI3K inhibitor)

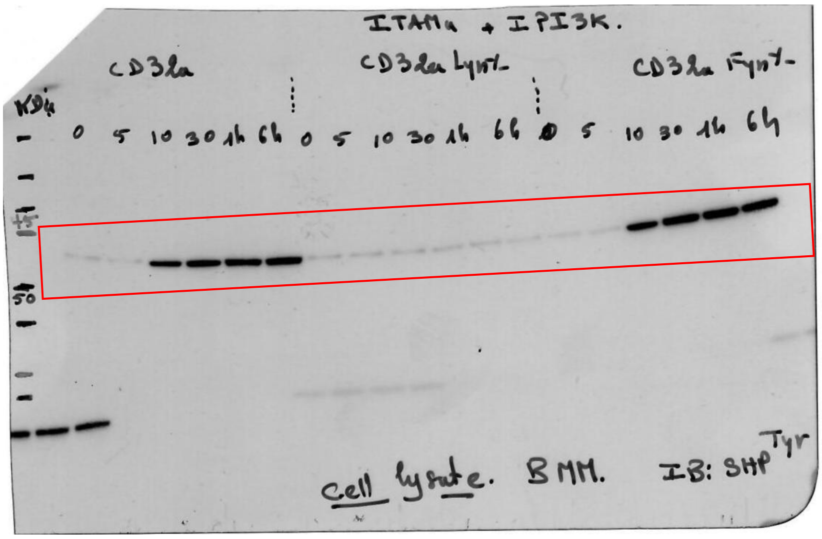

Figure 3c

IB : pERK (ERK inhibitor)

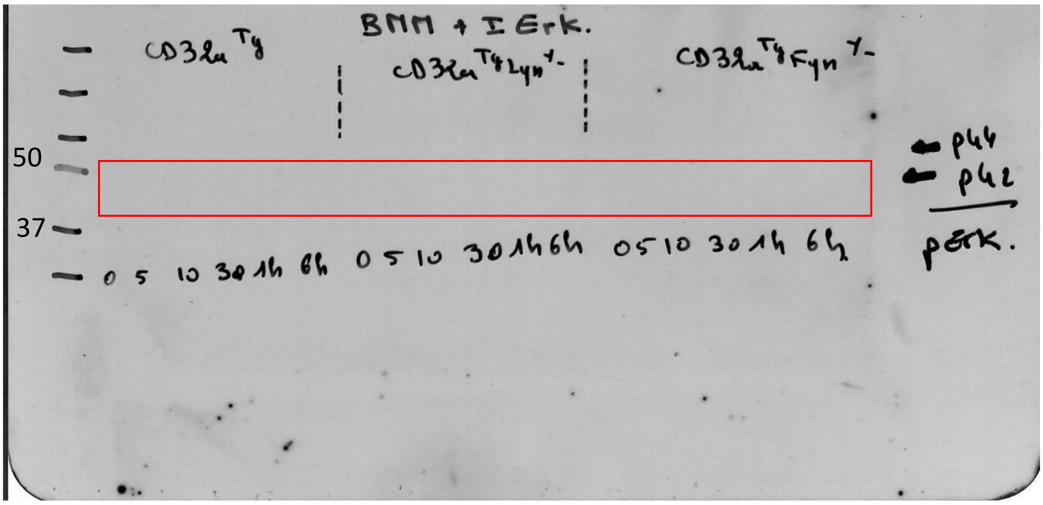

Figure 3c

IB : pSHP-1^{Y536} (ERK inhibitor)

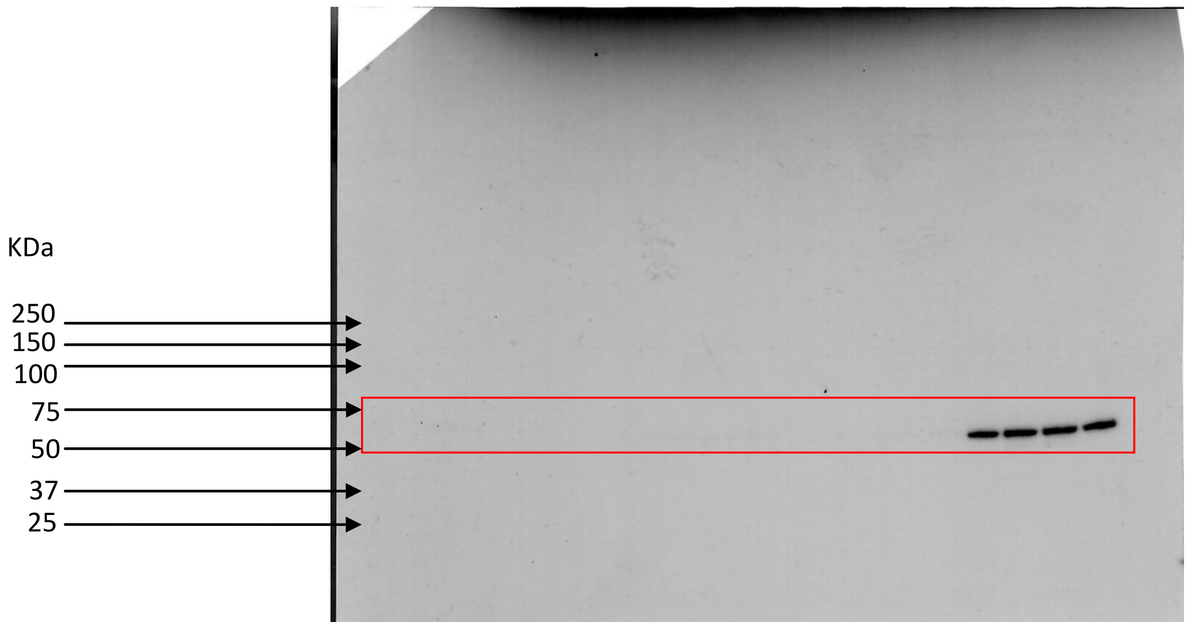

Figure 3d

IB: actin

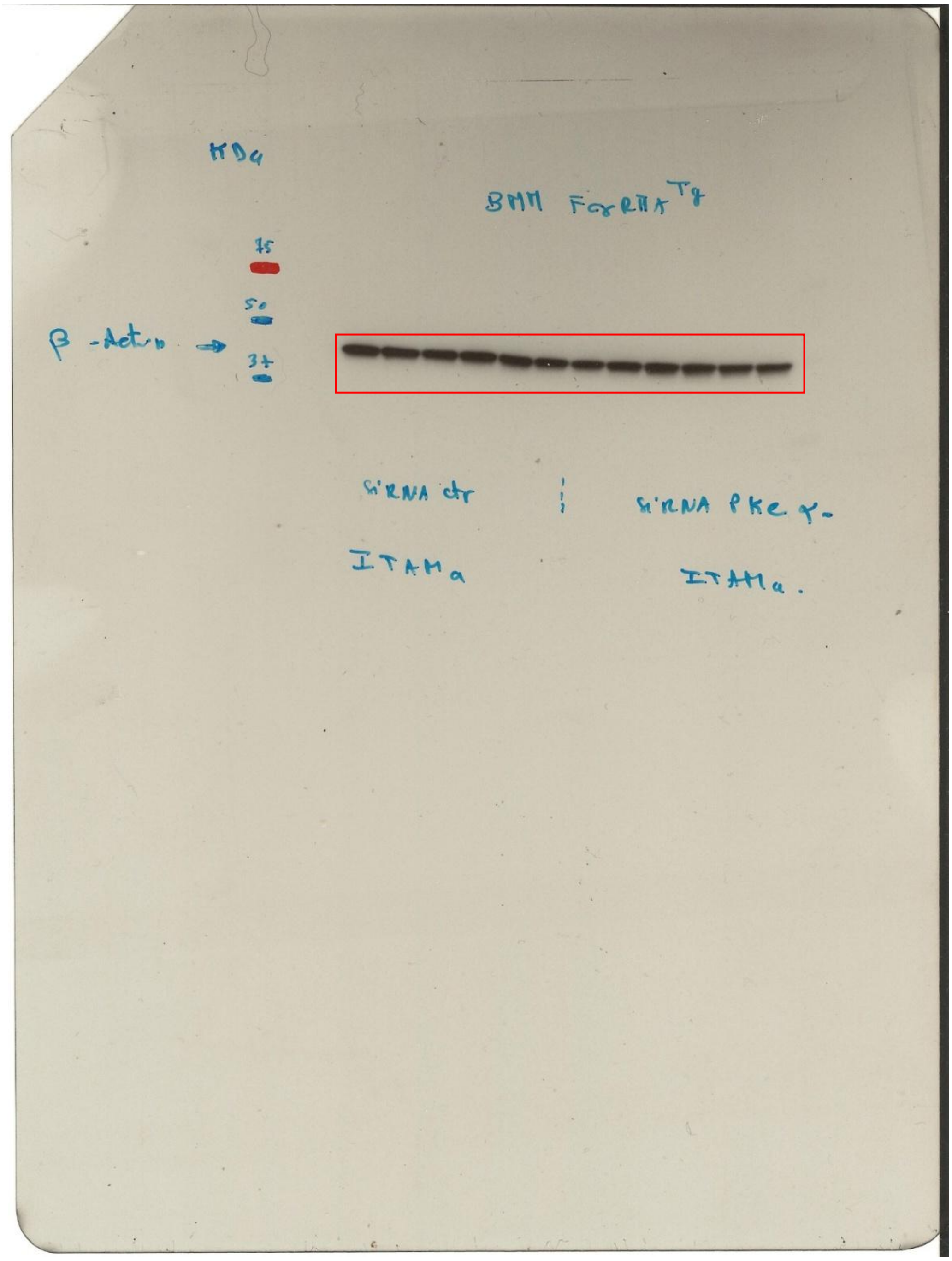

Figure 3d

IB: SHP-1

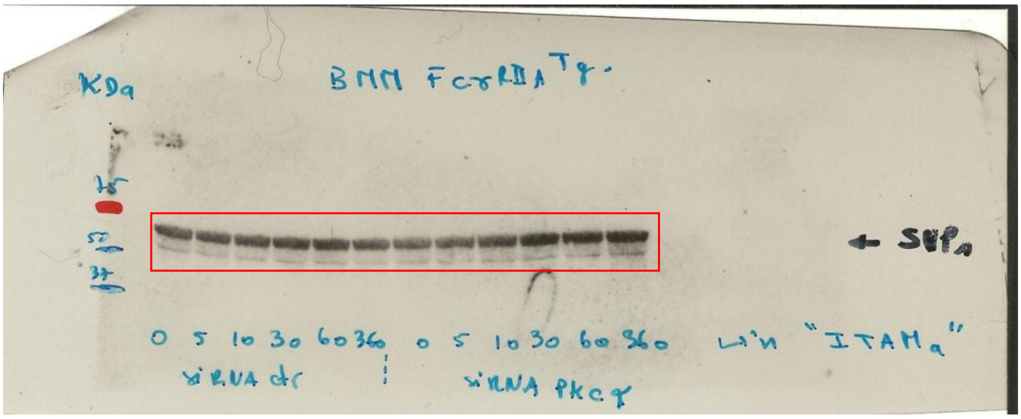

Figure 3d

IB: SHP-1^{Y536}

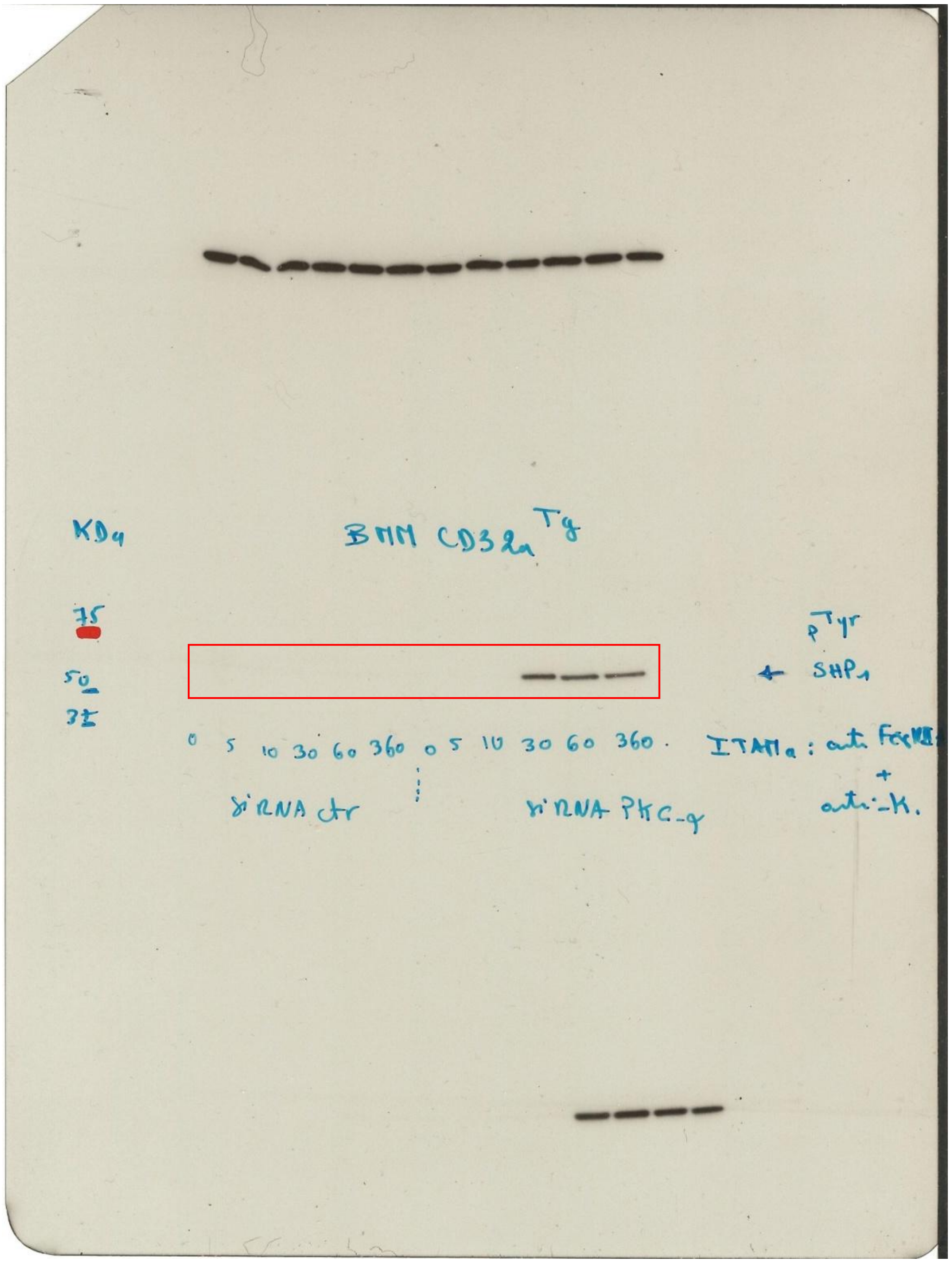

Figure 3d

IB: SHP-1^{S591}

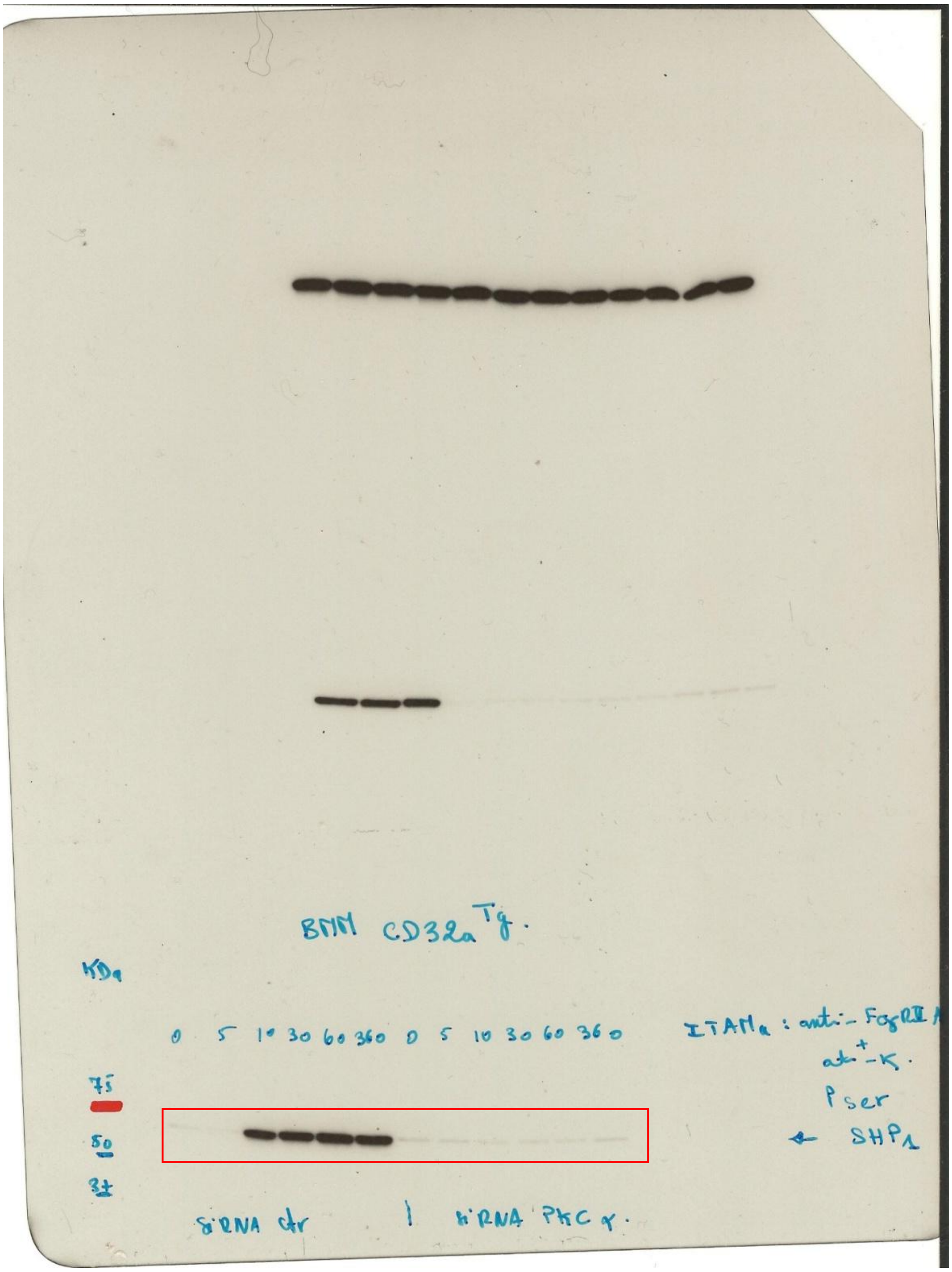

Figure 3d

IB: PKCα

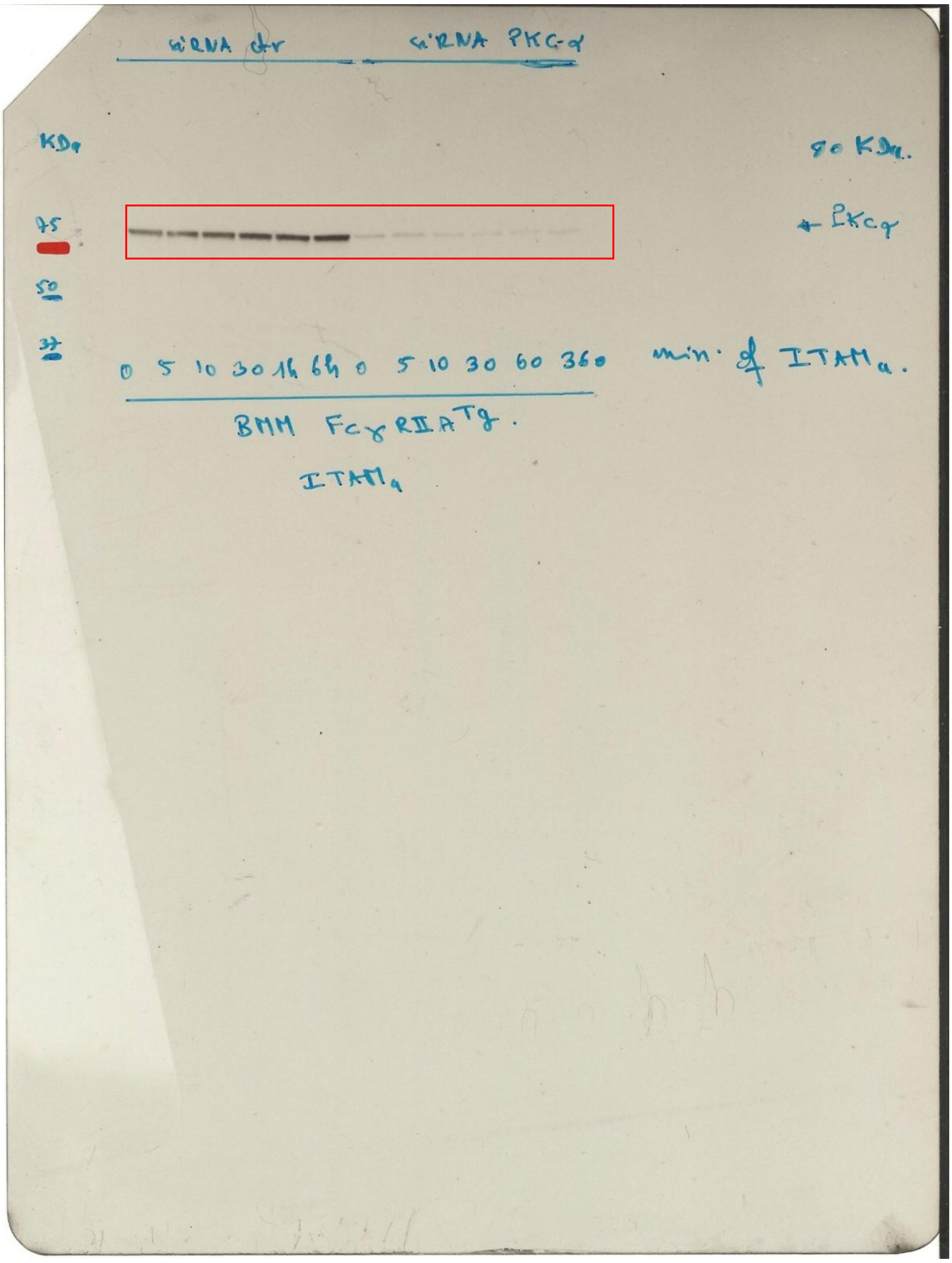

Figure 3e

IB: PKCα

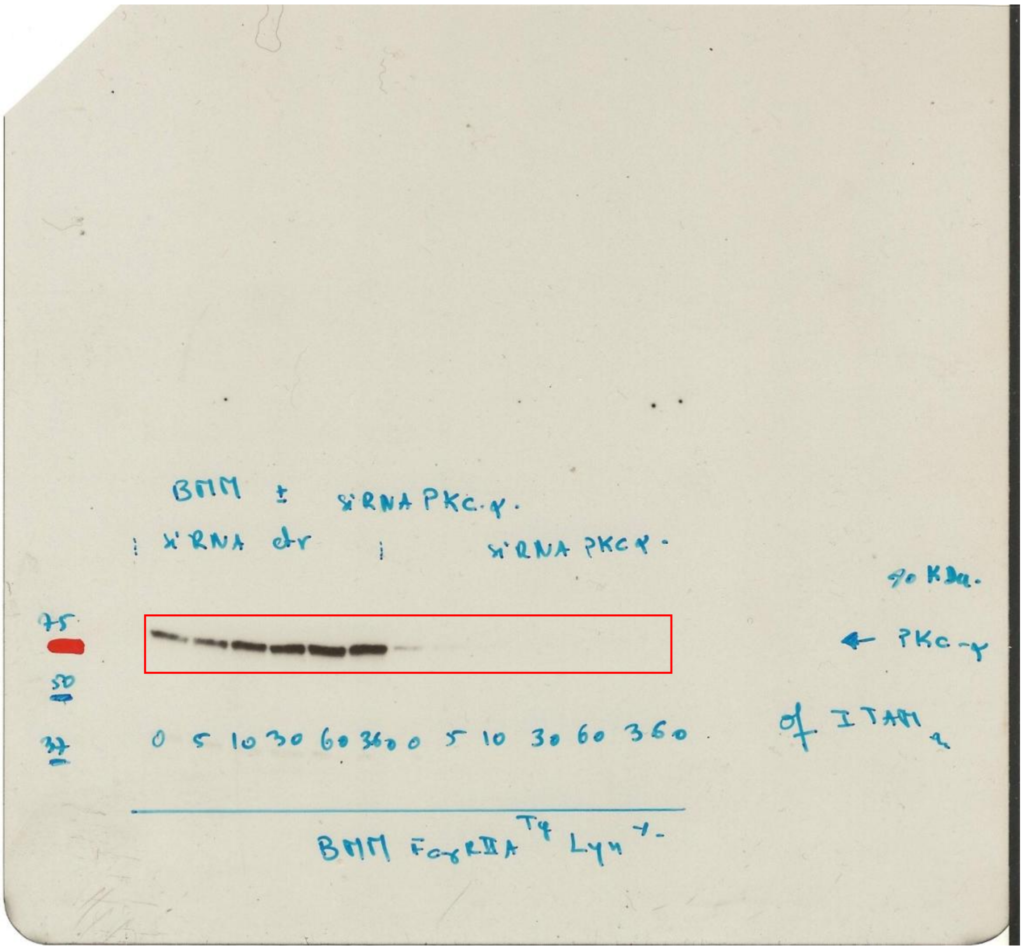

Figure 3e

IB: pSHP-1^{S591}

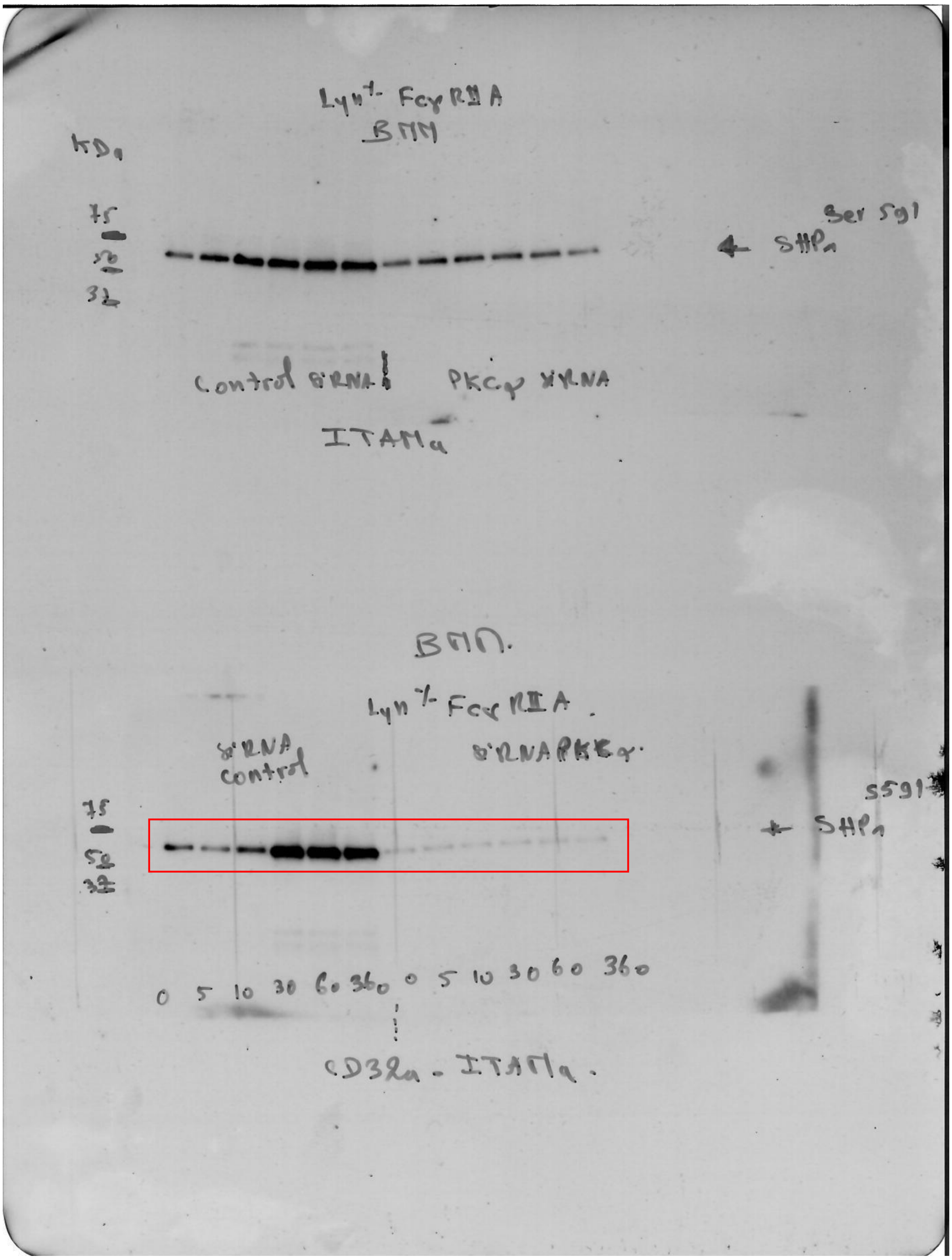

Figure 3e

IB: pSHP-1^{Y536}

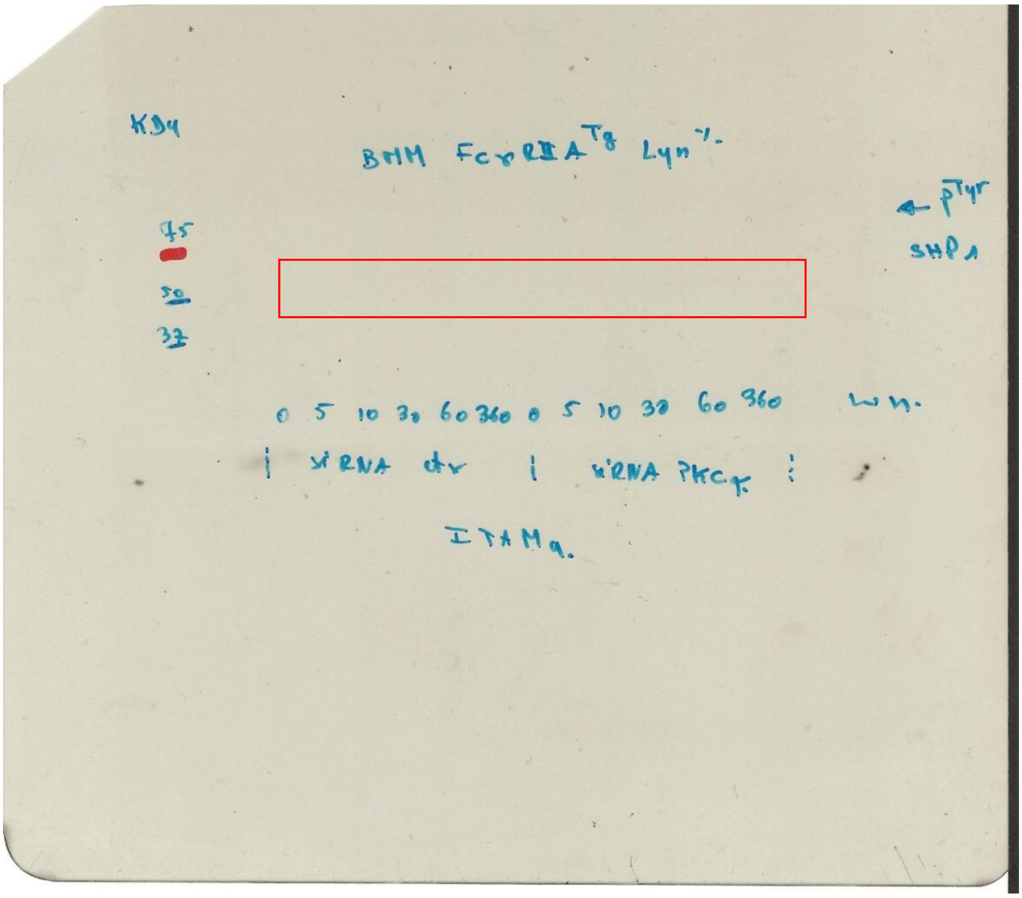

Figure 3e

IB: SHP-1

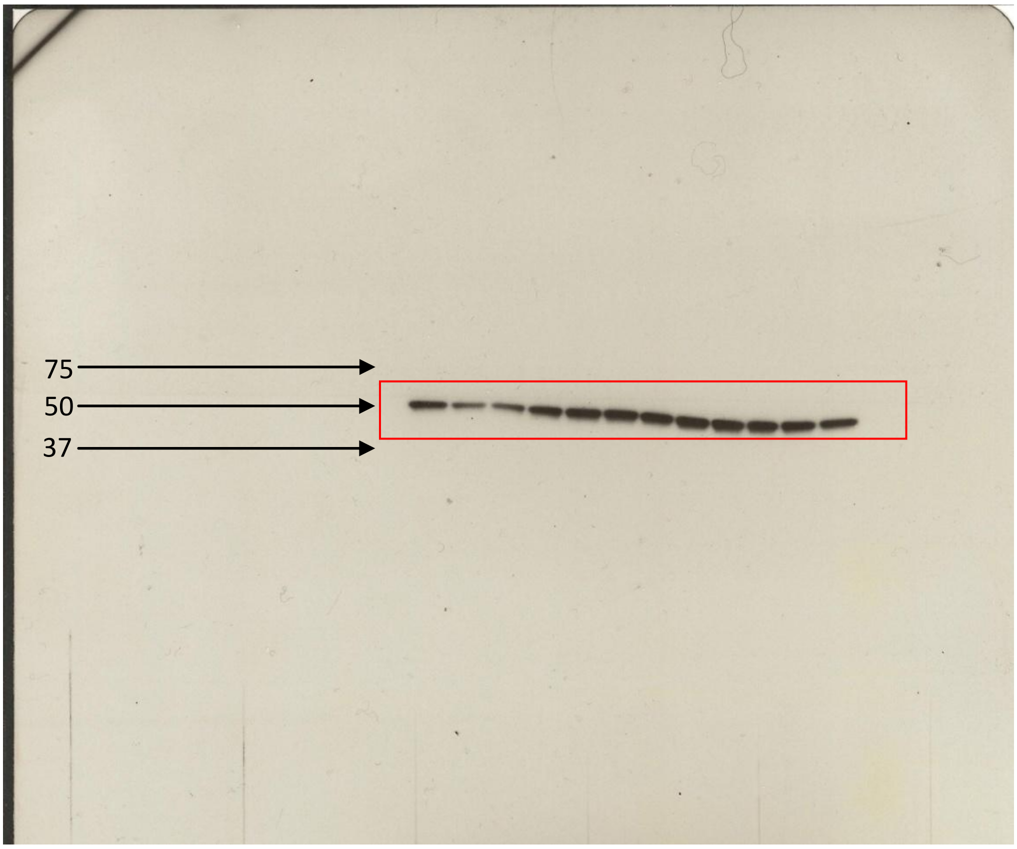

Figure 3f

IB: PKCα

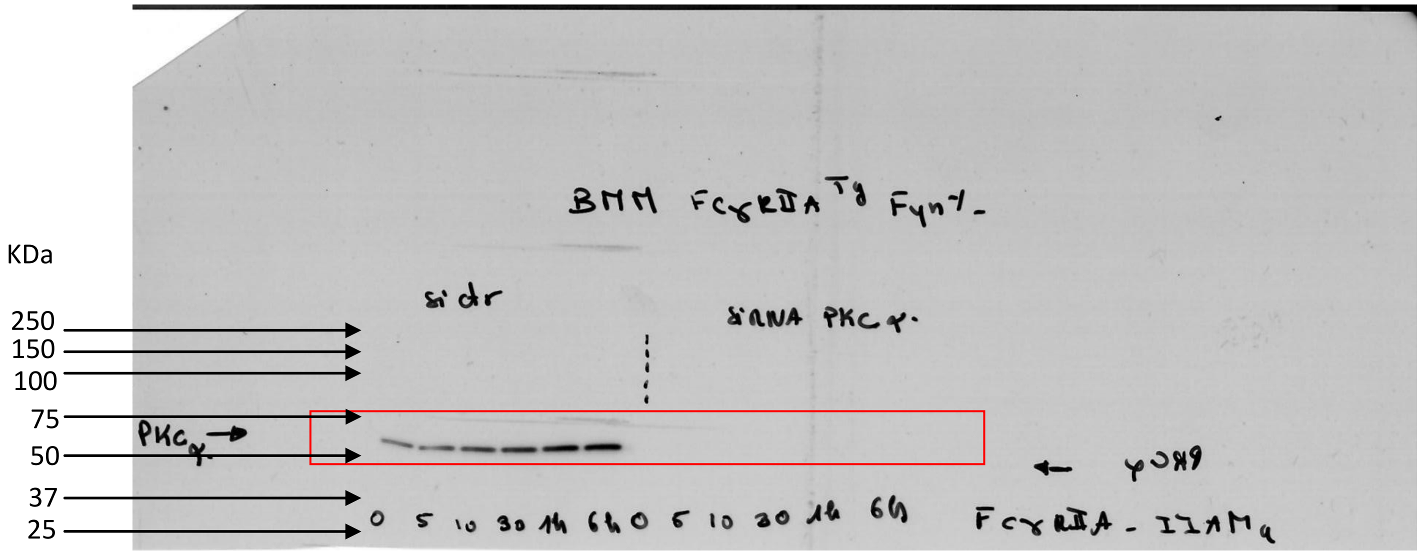

Figure 3f

IB: SHP-1^{Y536}

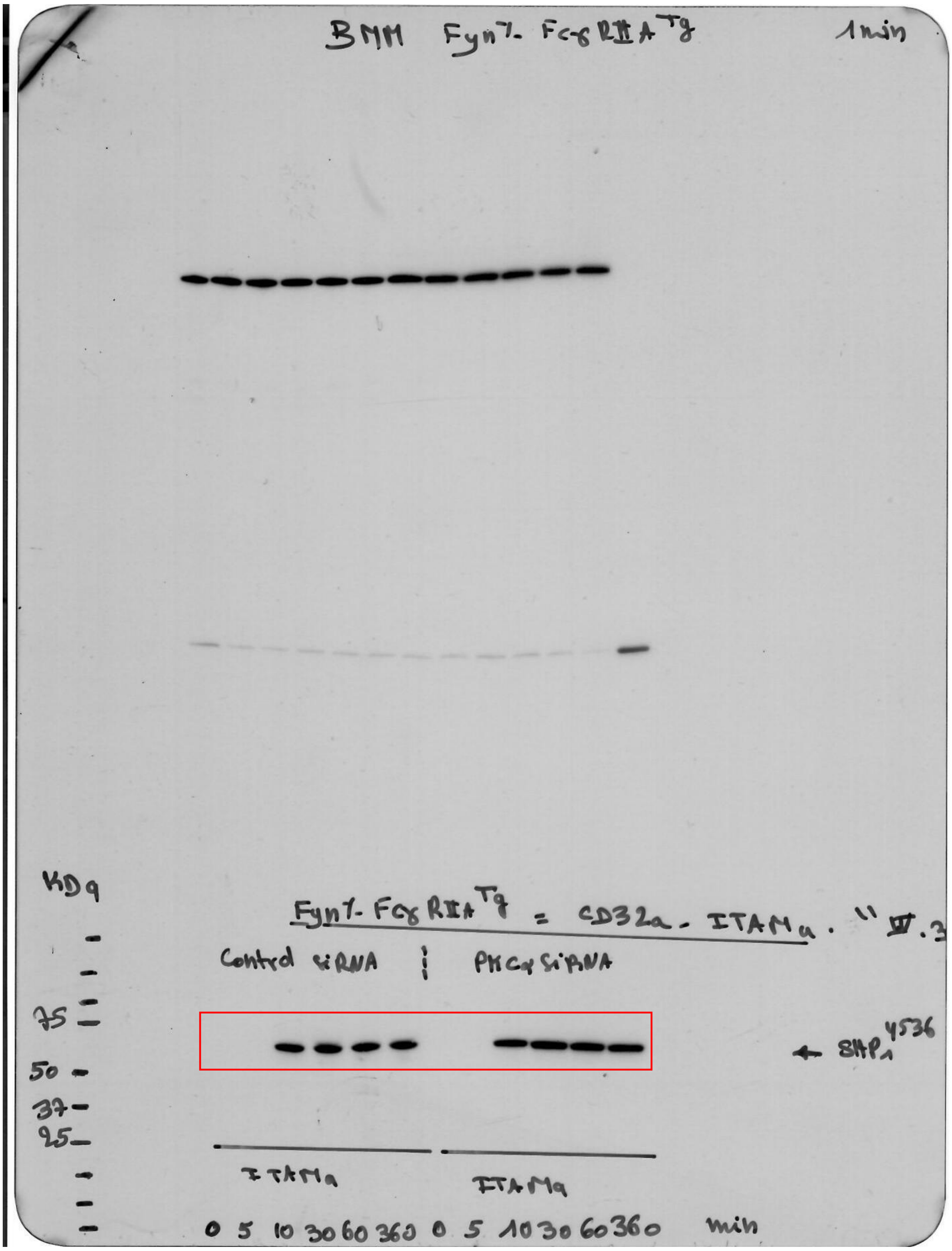

Figure 3f

IB: SHP-1

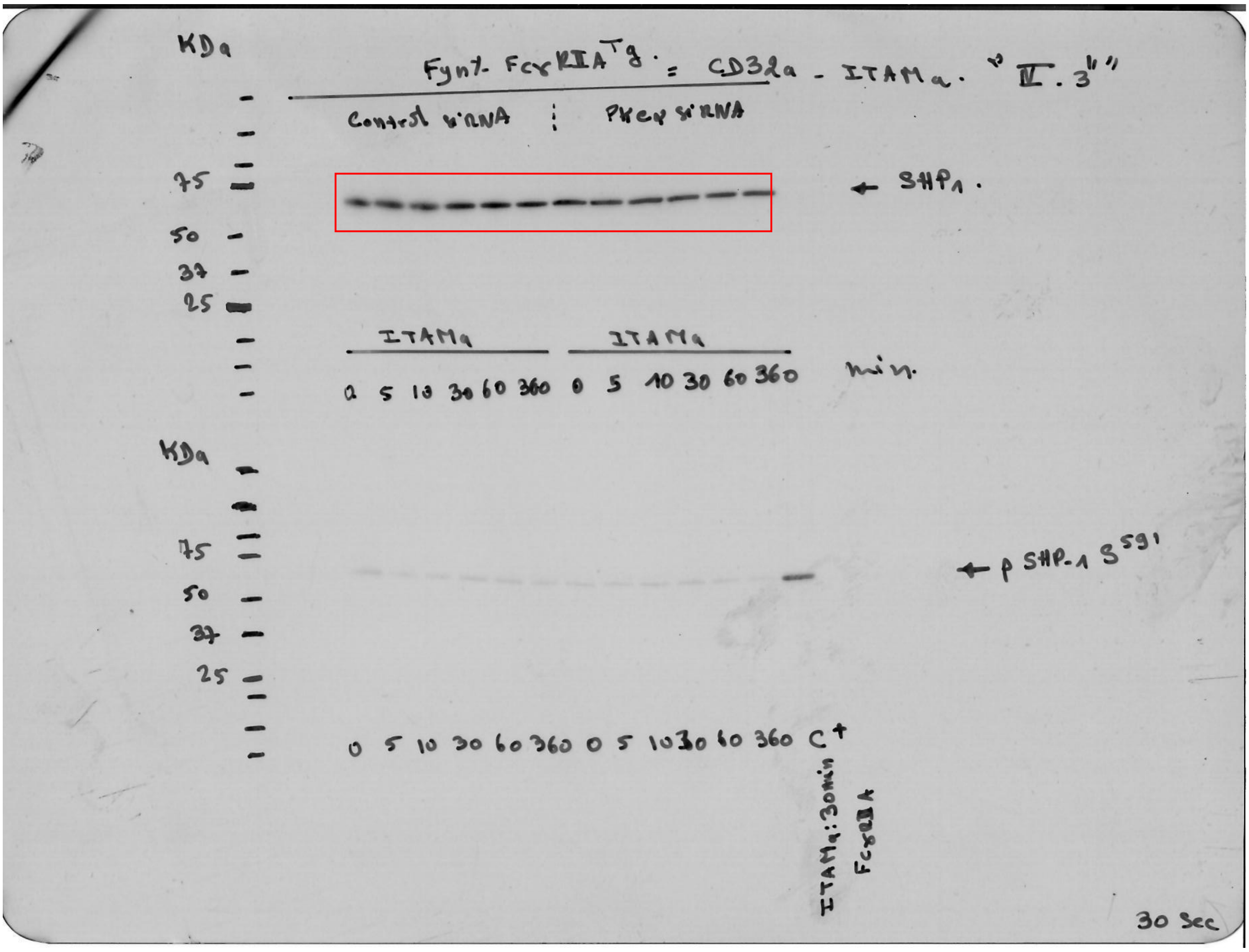

Figure 3f

IB: pSHP-1^{S591}

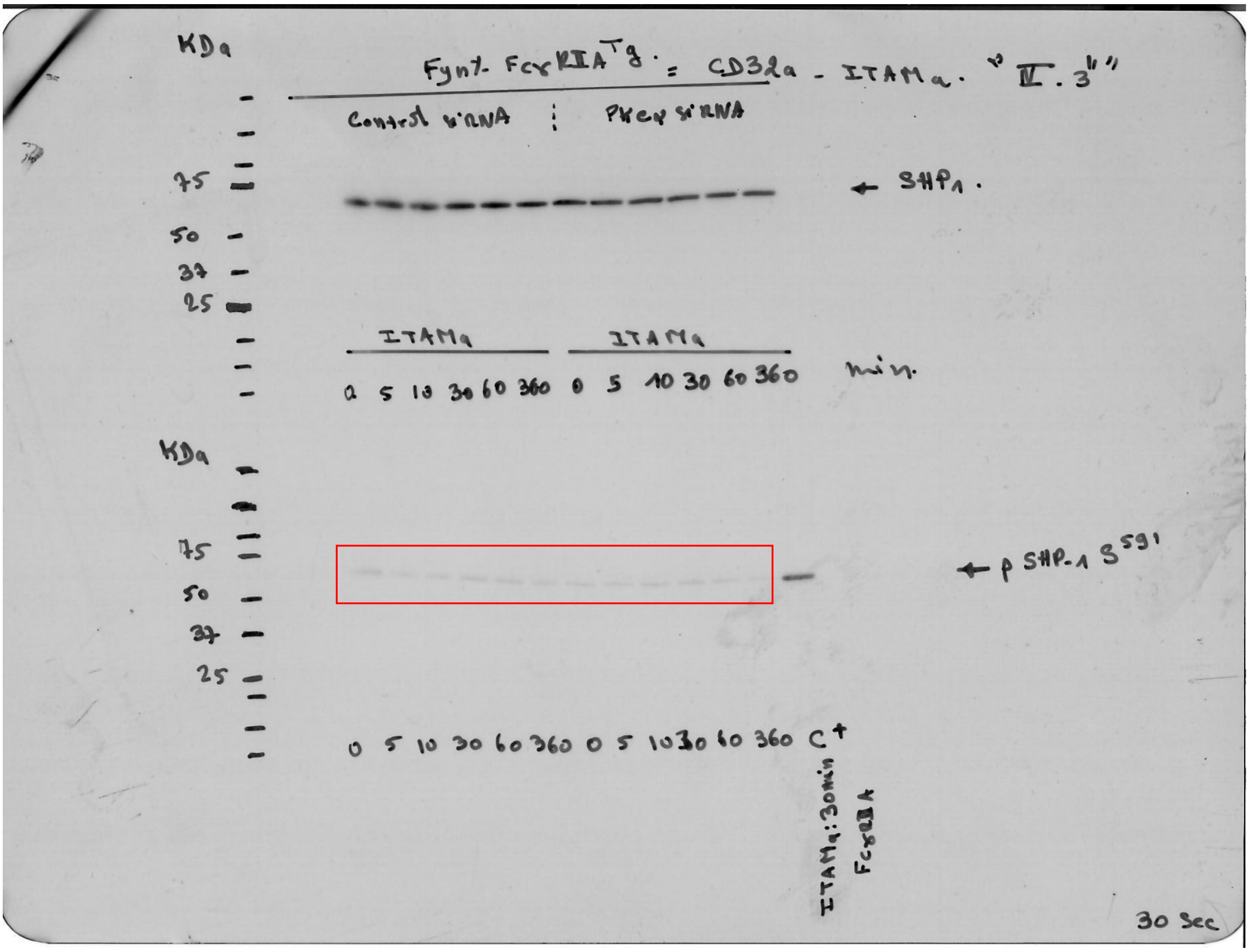

Figure 3f

IB: actin

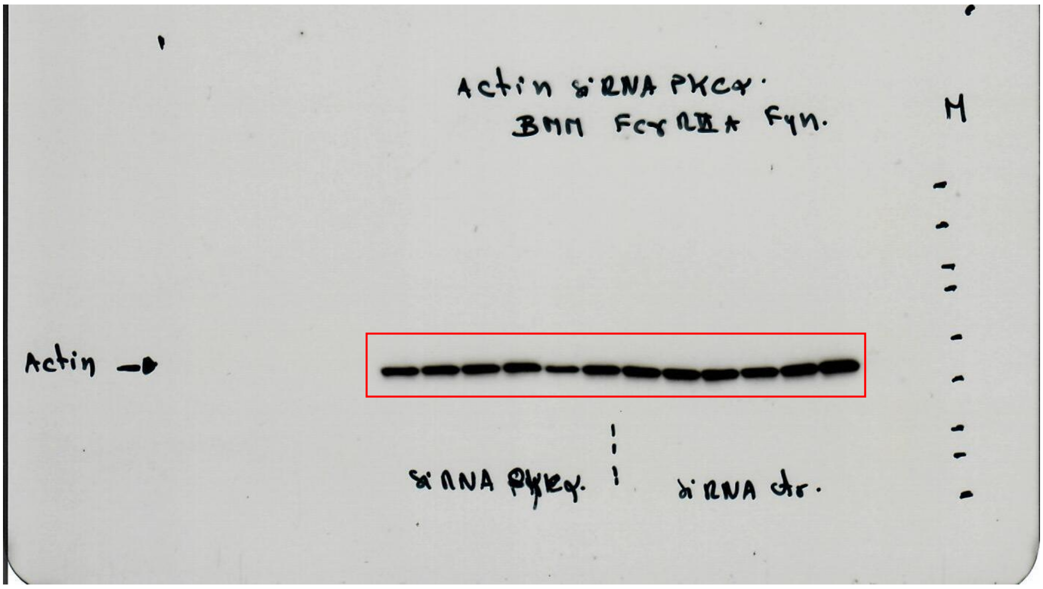

Figure 7a

IB: Fyn, Lysate

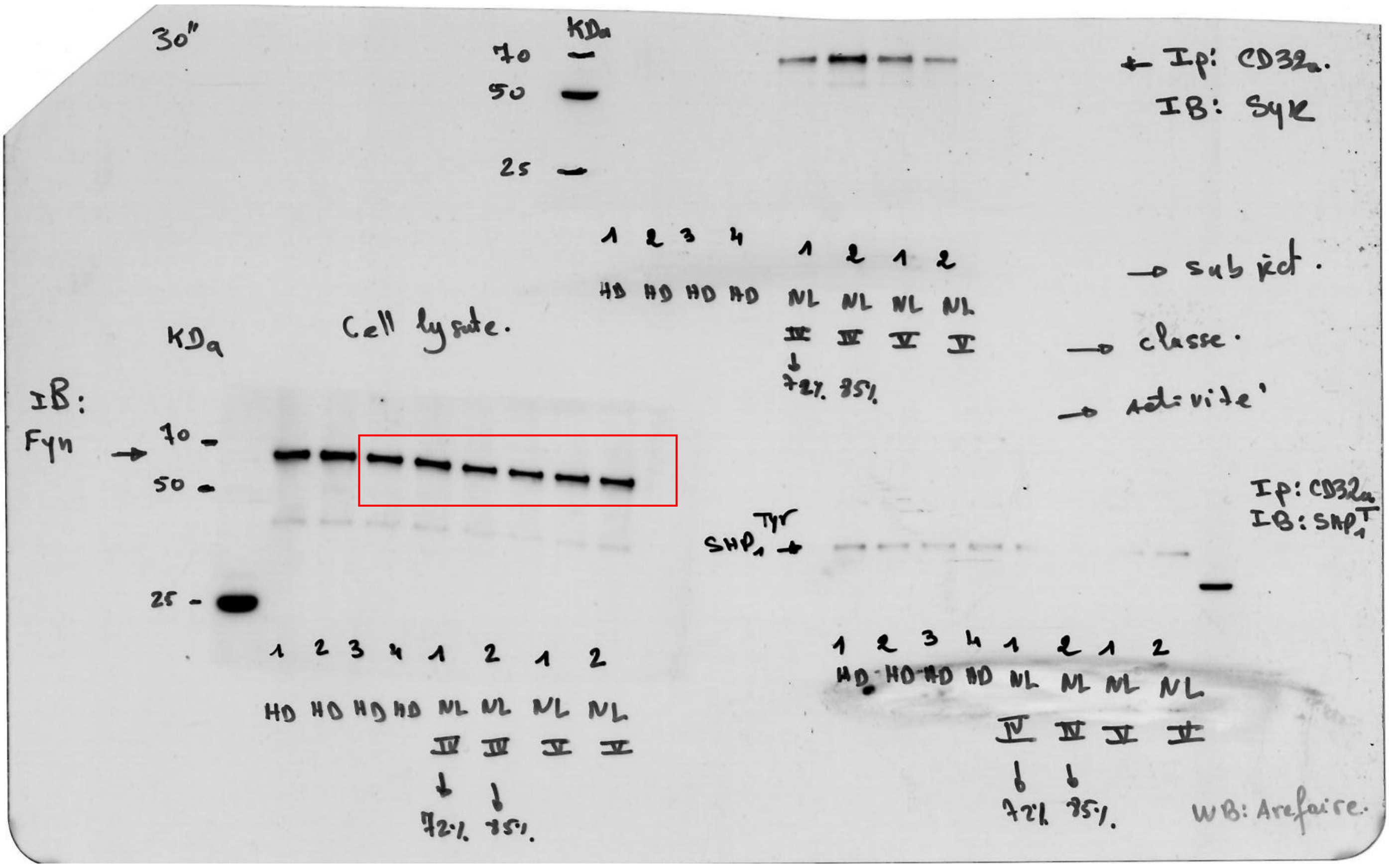

Figure 7a

IB: Syk, IP: FcγRIIA

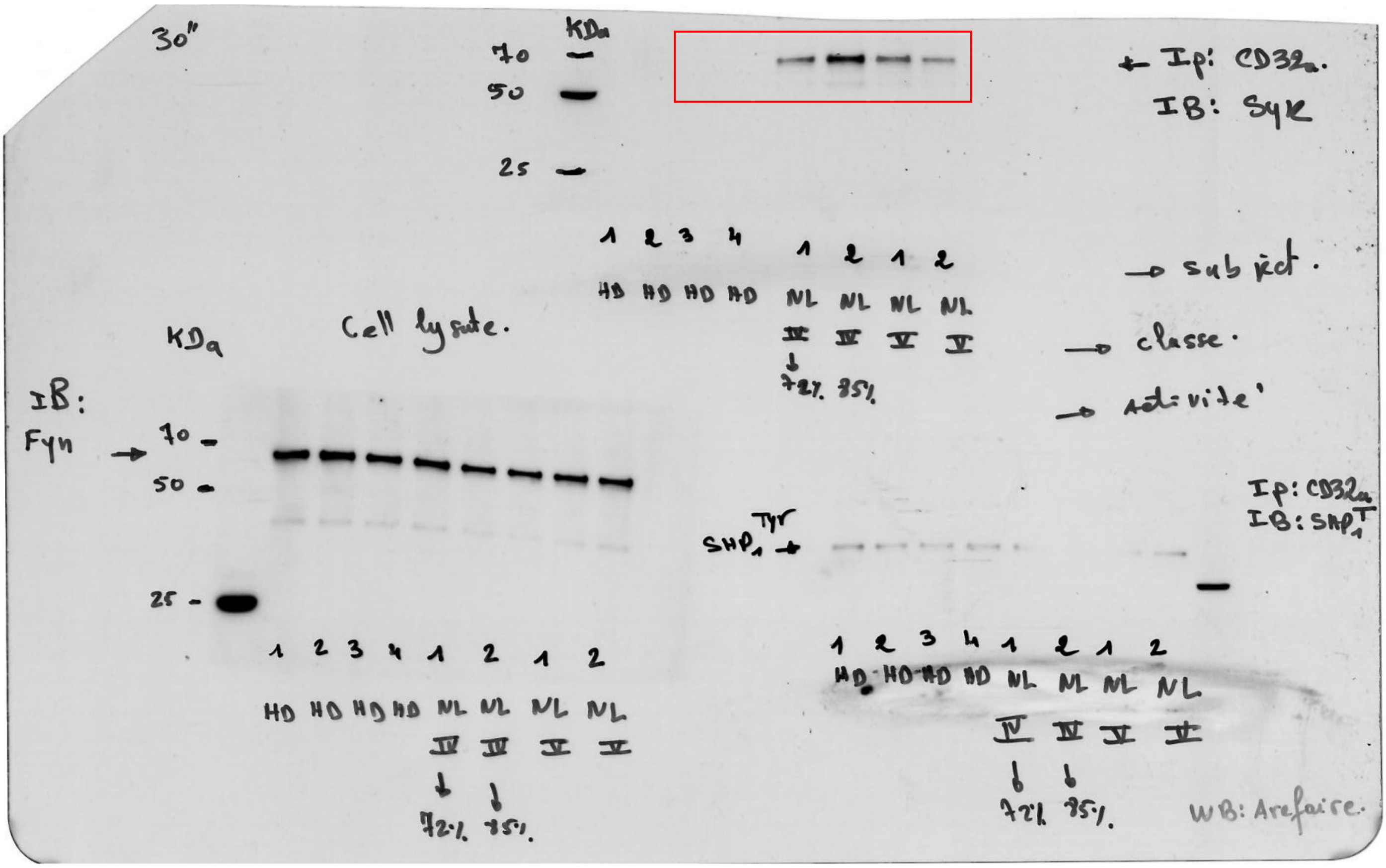

Figure 7a

IB: pPKCα, IP: FcγRIIA

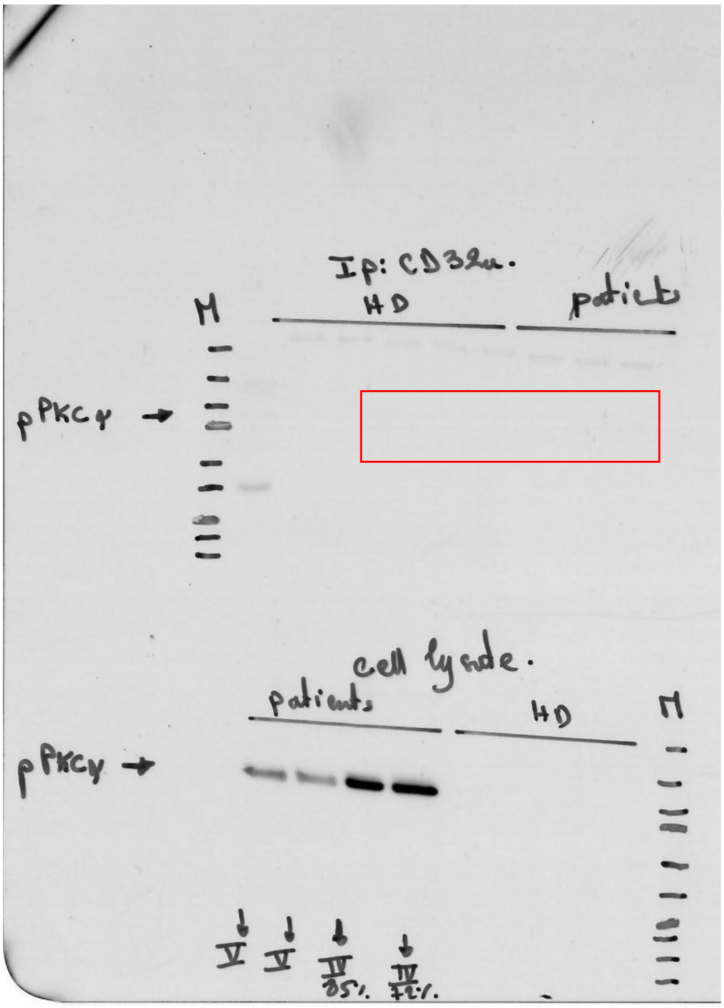

Figure 7a

IB: pPKCα, Lysate

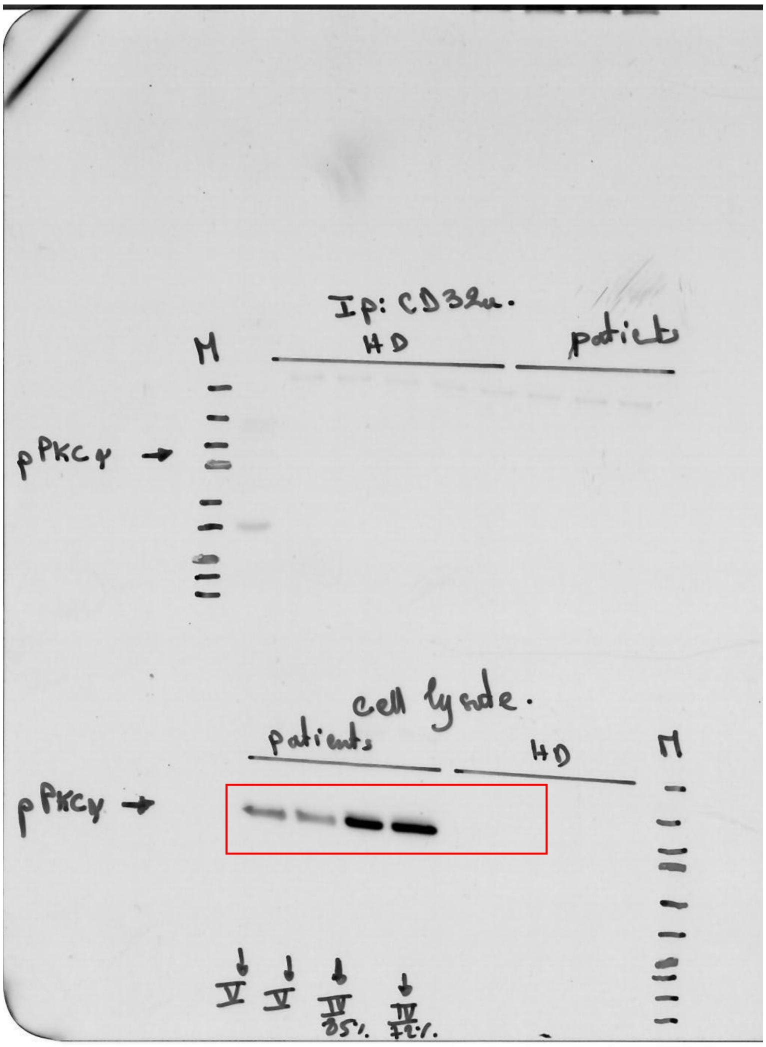

Figure 7a

IB: pSHP-1^{S591}, IP: FcγRIIA

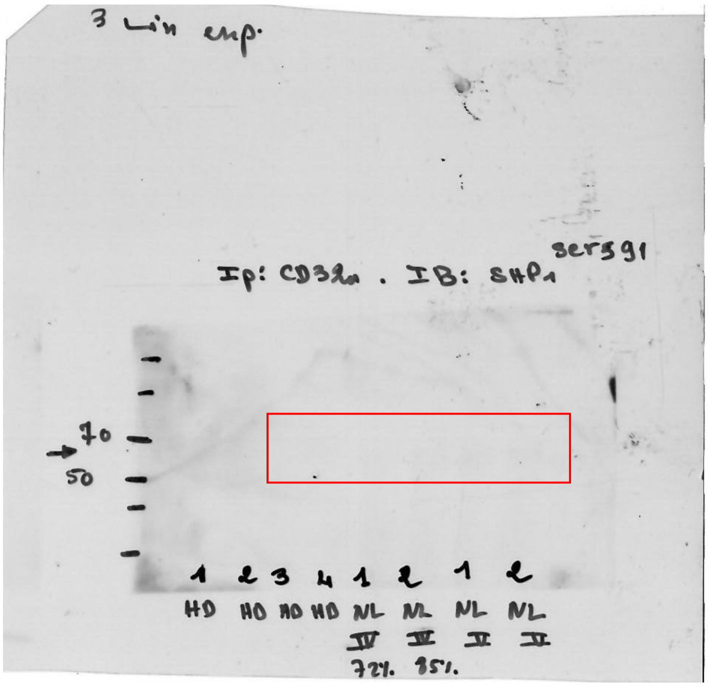

Figure 7a

IB: Fyn, IP: FcγRIIA

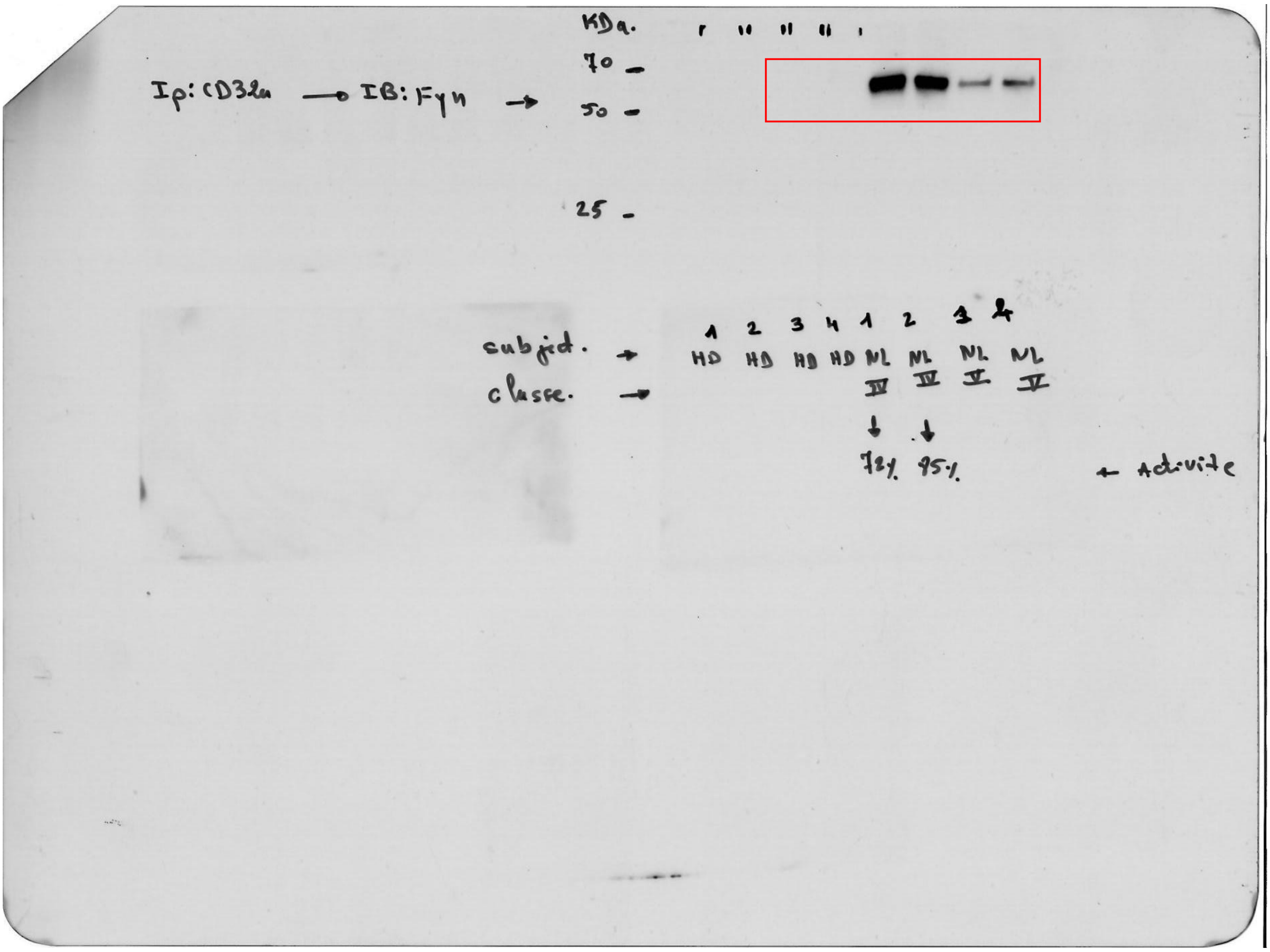

Figure 7a

IB: pSHP-1^{Y536}, IP: FcγRIIA

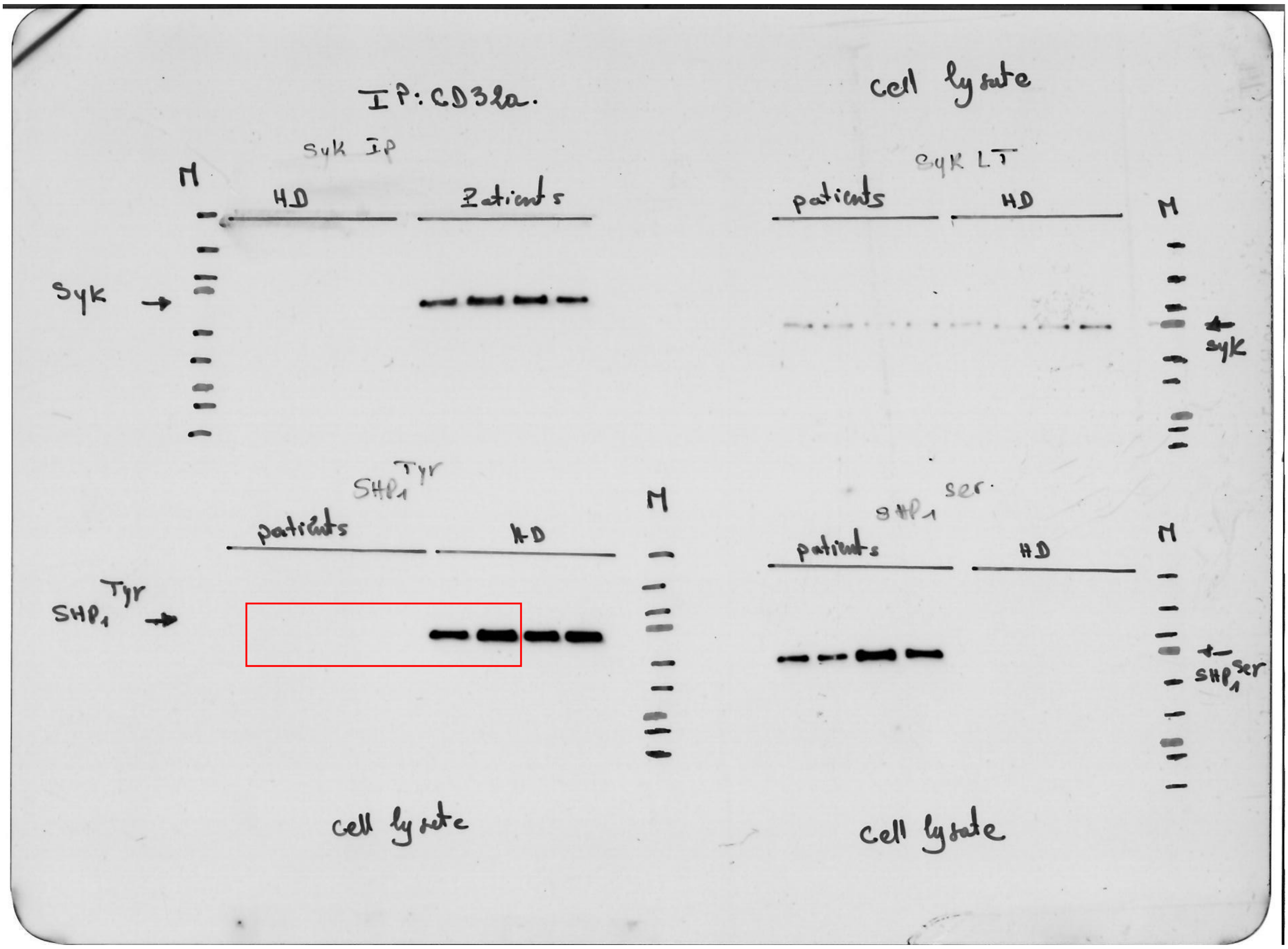

Figure 7a

IB: pSHP-1^{S591}, Lysate

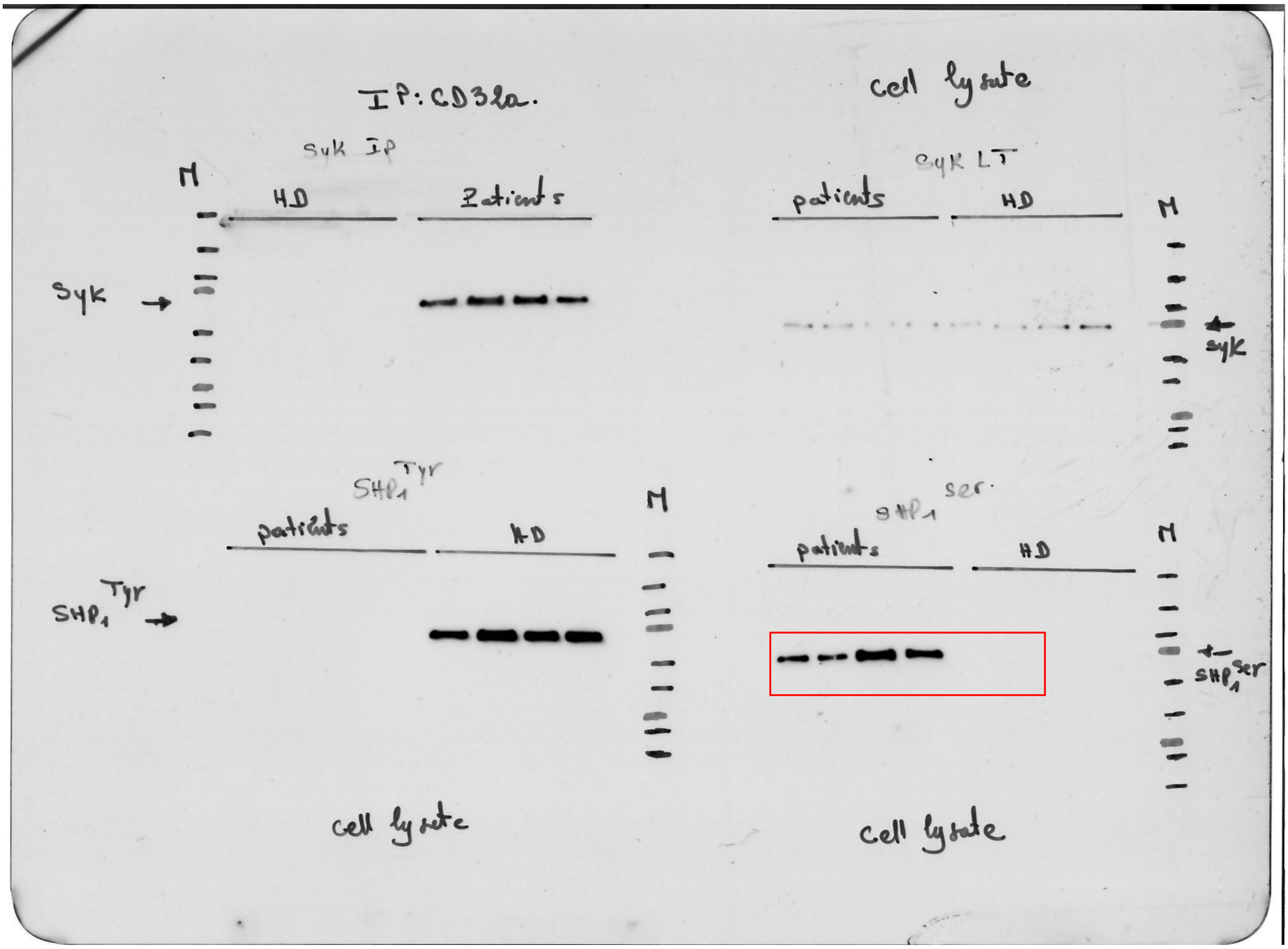

Figure 7a

IB: Syk, Lysate

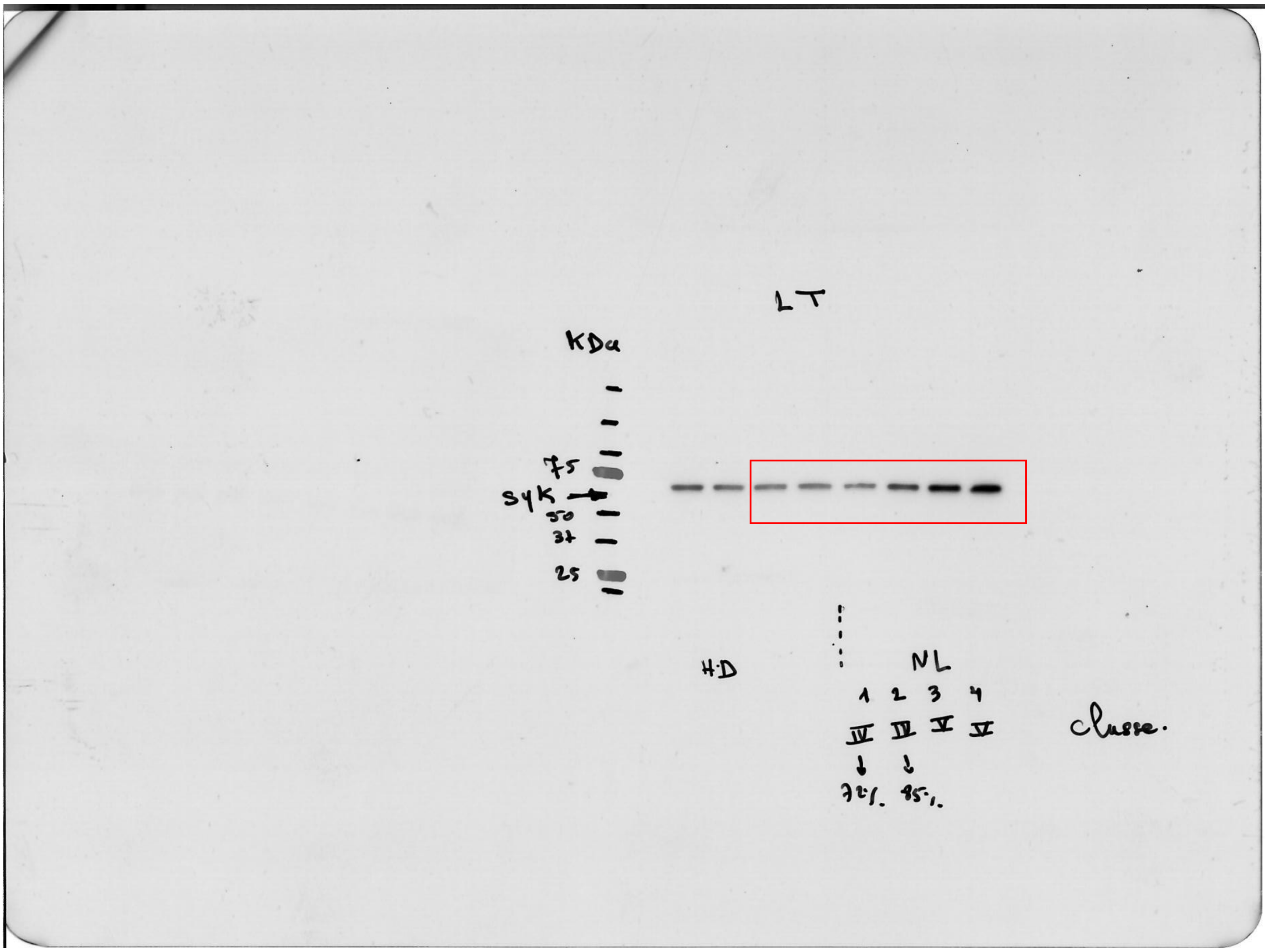

Figure 7a

IB: FcγRIIA, IP: FcγRIIA

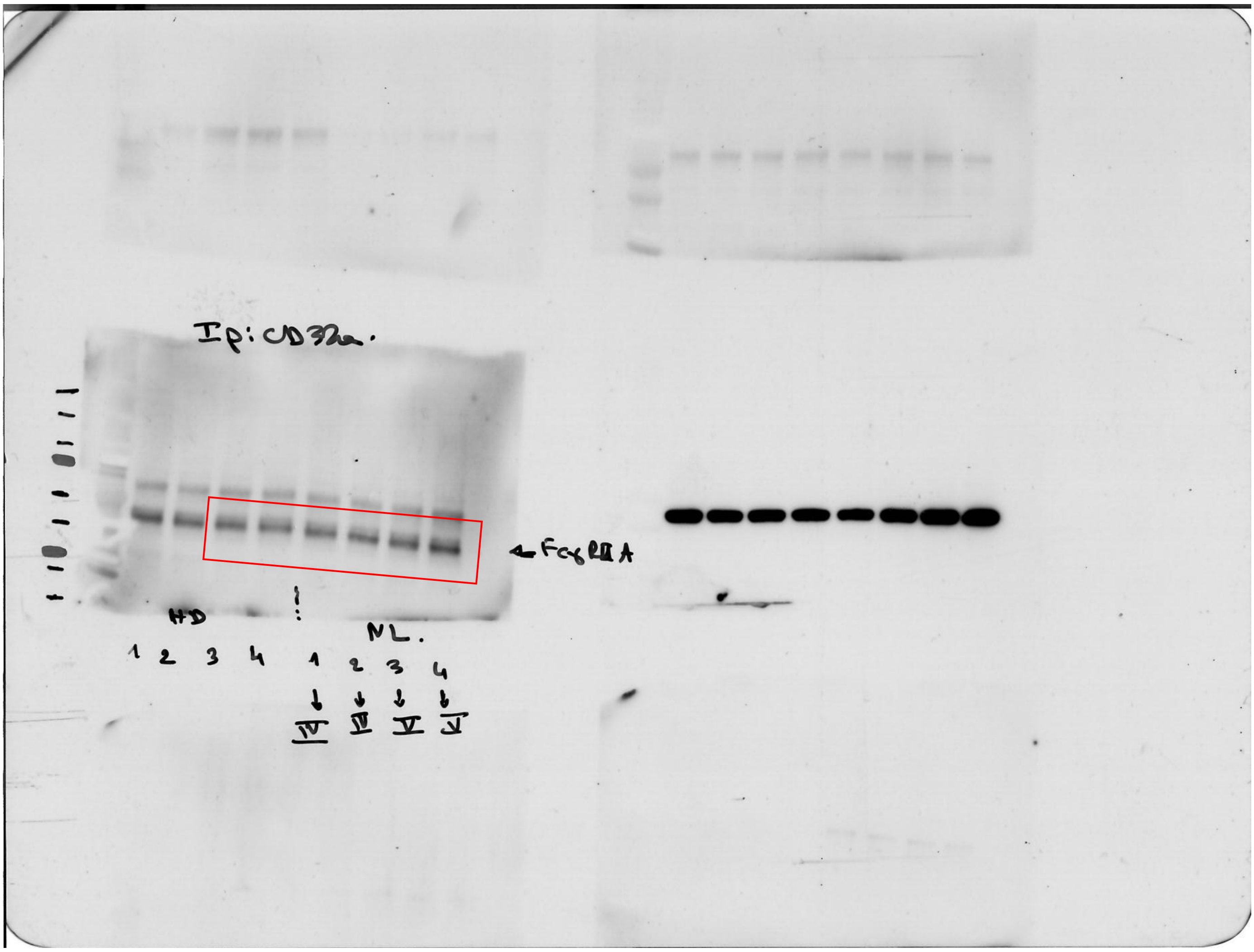

Figure 7a

IB: Lyn, Lysate

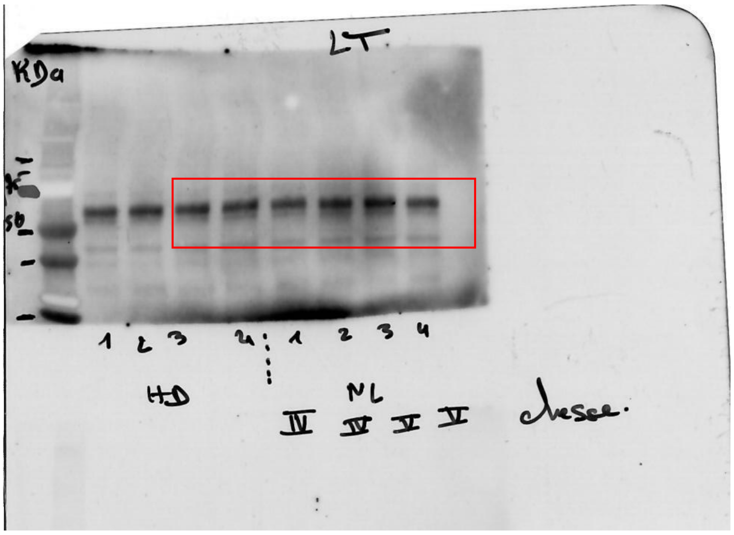

Figure 7a

IB: Lyn, IP: FcγRIIA

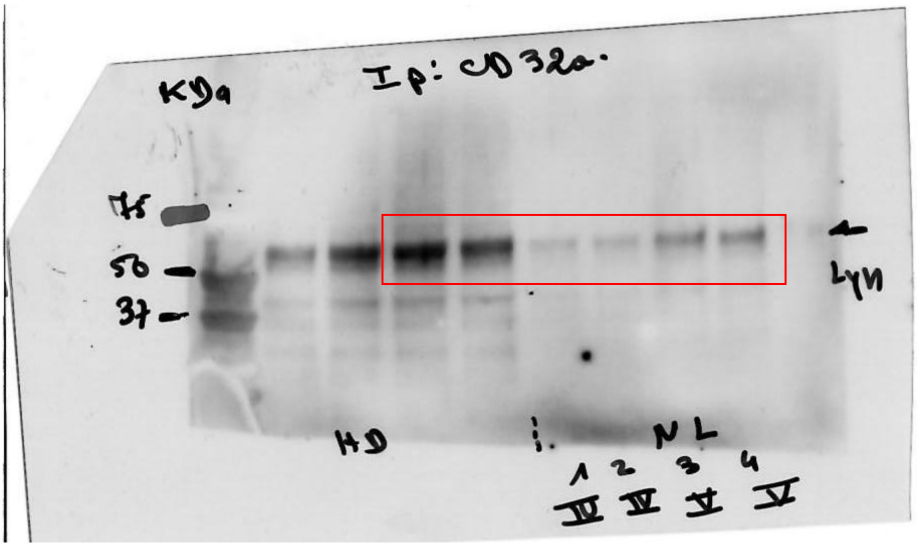

Figure 7a

IB: SHP-1, IP: FcγRIIA

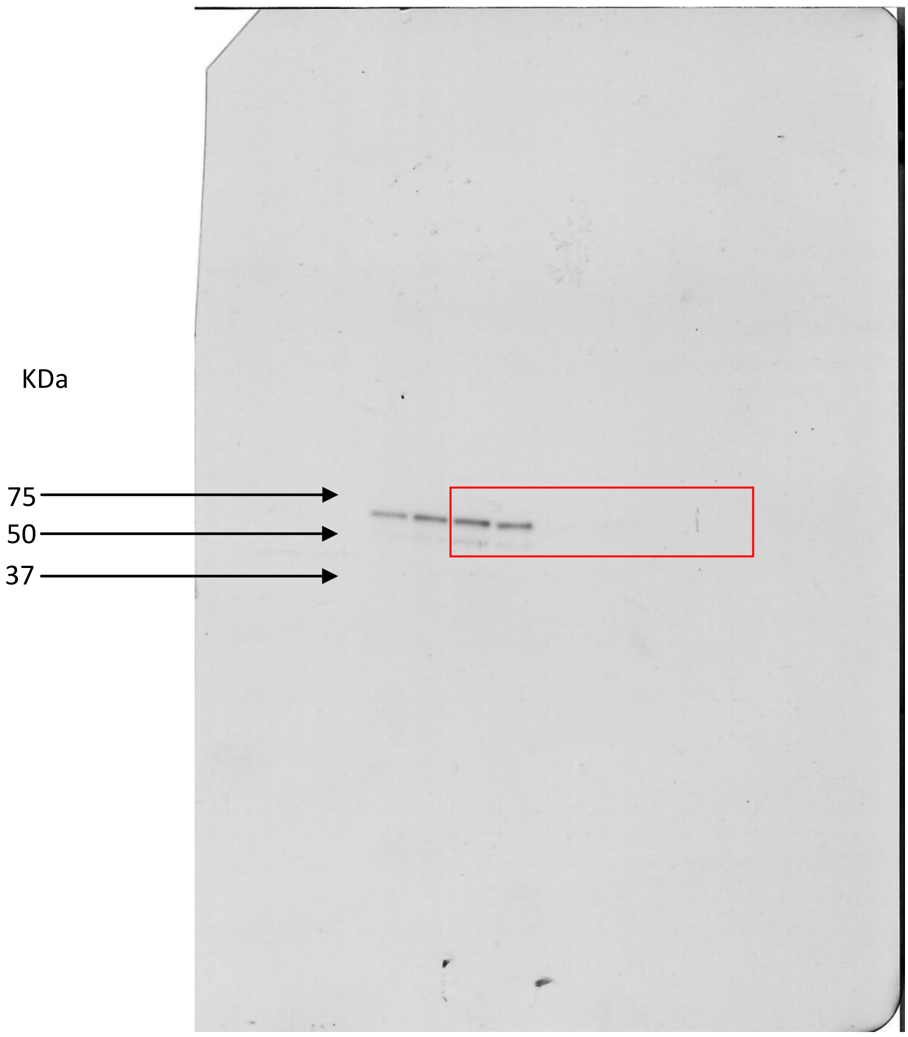

Figure 7a

IB: pSHP-1^{Y536}, Lysate

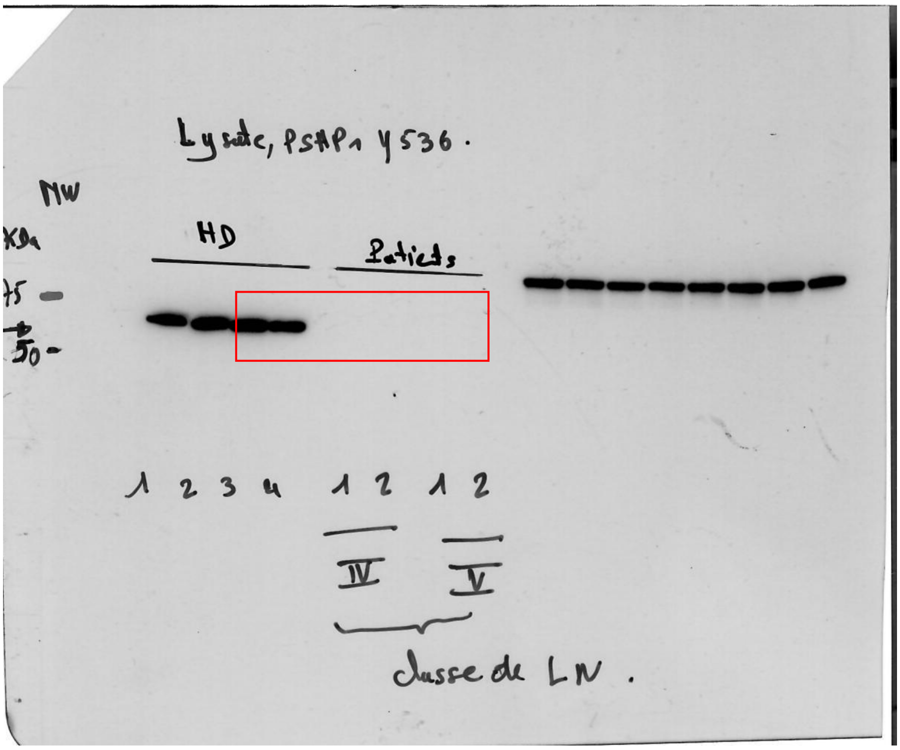

Figure 7a

IB: FcγRIIA, Lysate

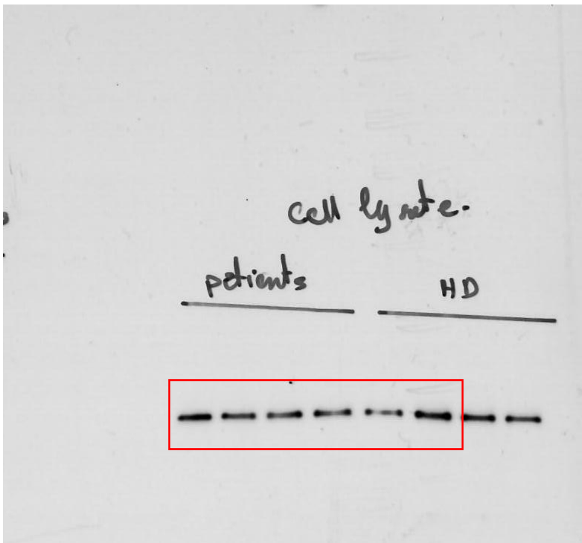

Figure 7a

IB: SHP-1, Lysate

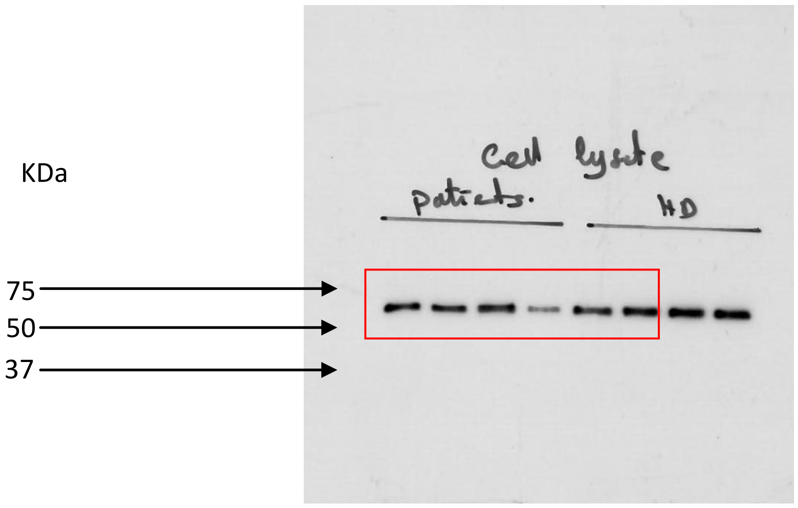

Supplementary Figure 1a

Lysate, IB: Fyn (control siRNA)

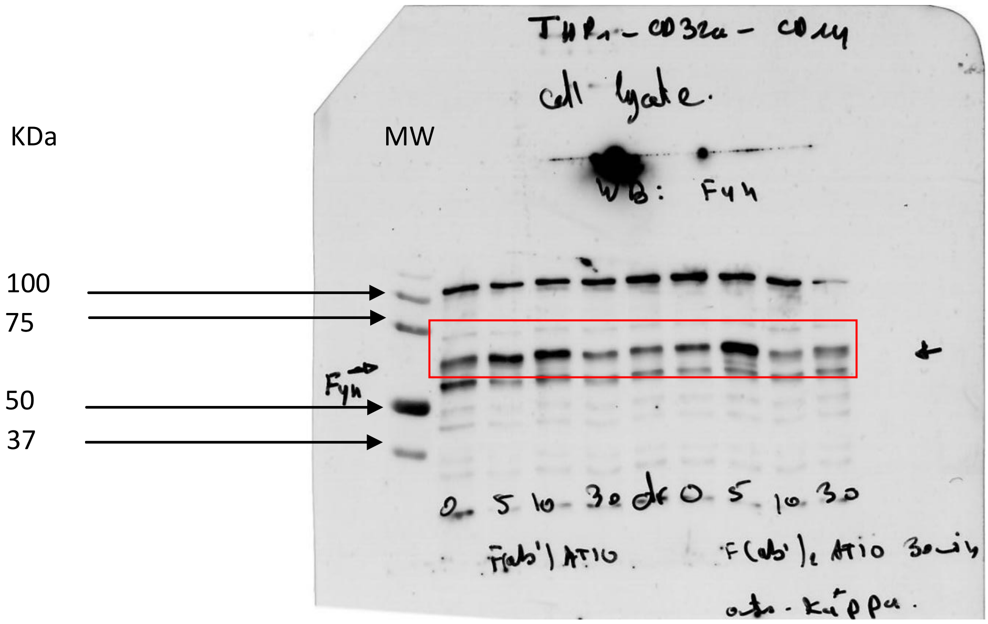

Supplementary Figure 1a

Lysate, IB: Lyn (control siRNA)

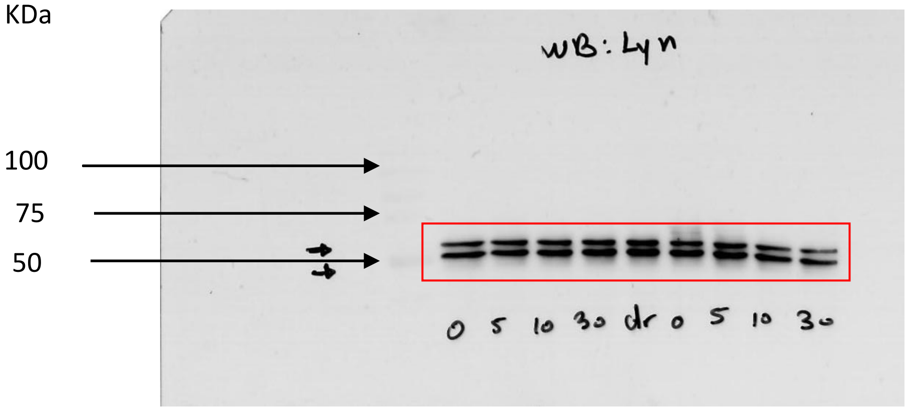

Supplementary Figure 1a

Lysate, IB: Syk (control siRNA)

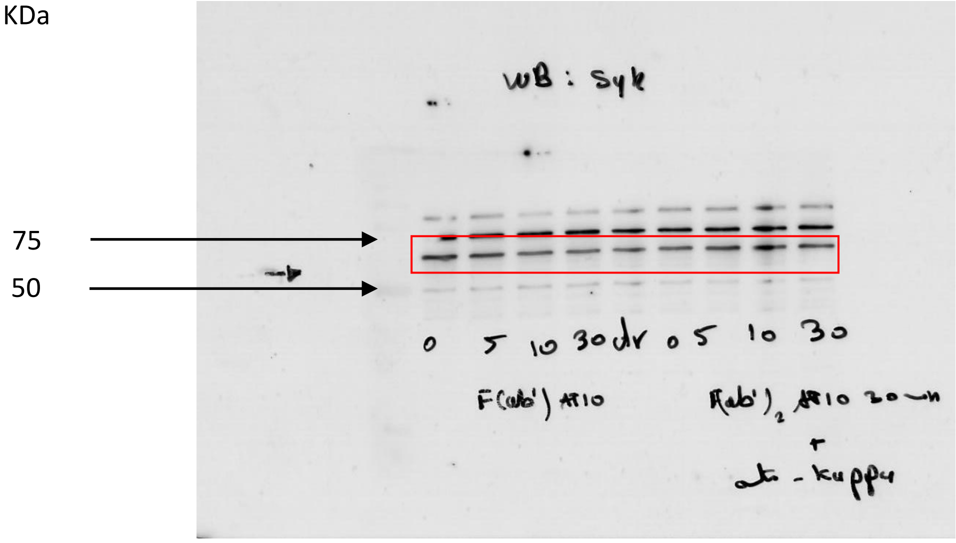

Supplementary Figure 1a

Lysate, IB: SHP-1 (control siRNA)

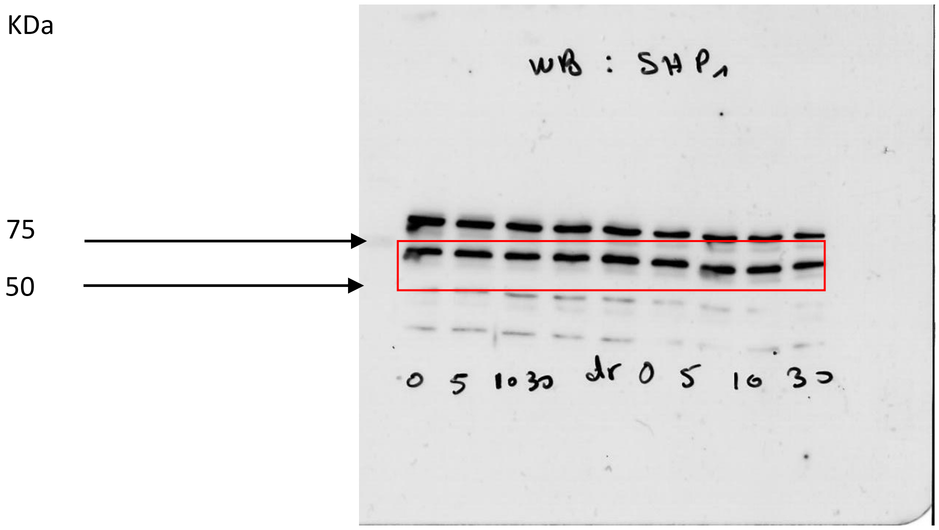

Supplementary Figure 1a

Lysate, IB: Lyn (Fyn siRNA)

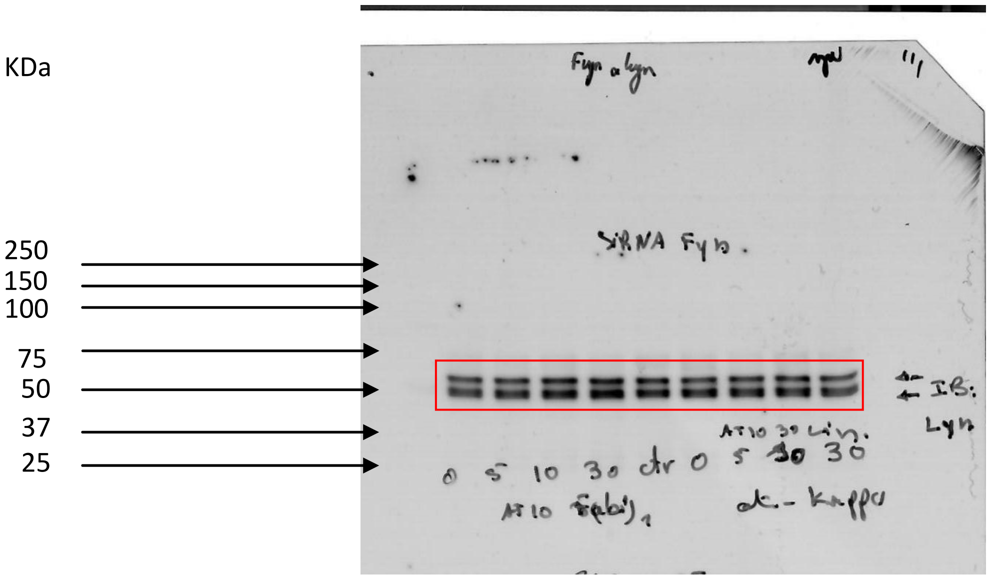

Supplementary Figure 1a

Lysate, IB: Fyn (Lyn siRNA)

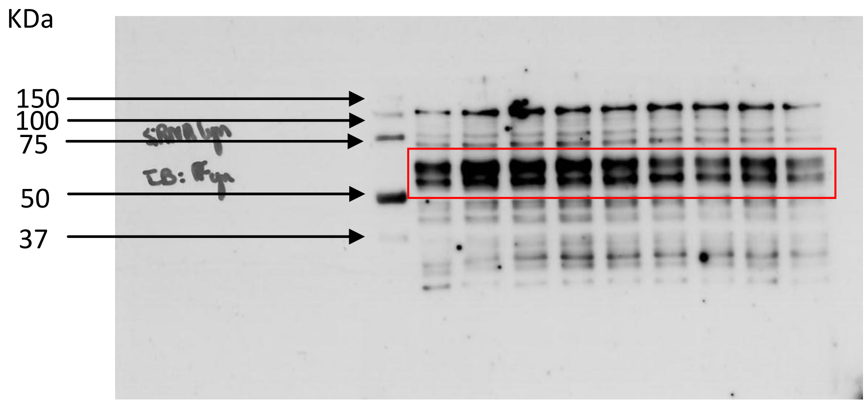

Supplementary Figure 1a

Lysate, IB: Fyn (Fyn siRNA)

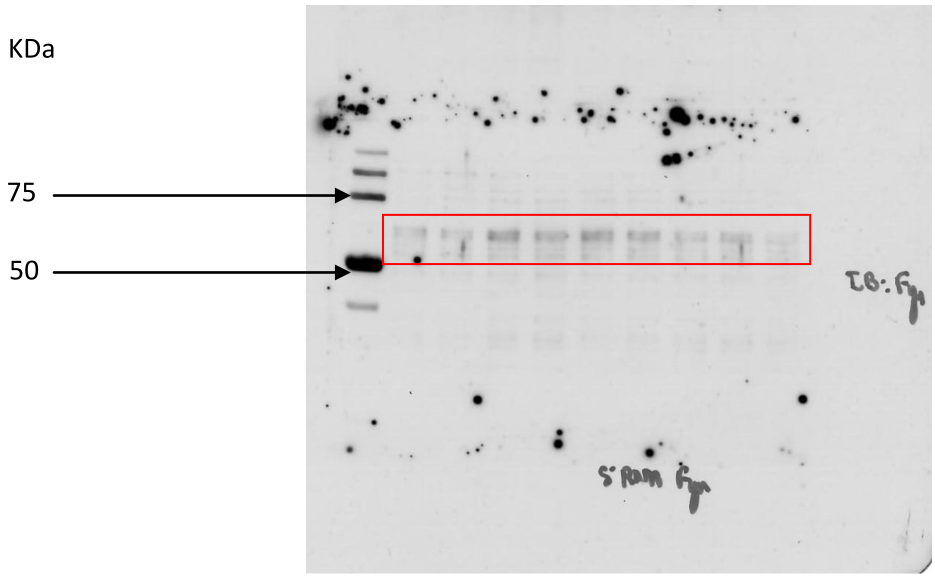

Supplementary Figure 1a

Lysate, IB: Lyn (Lyn siRNA)

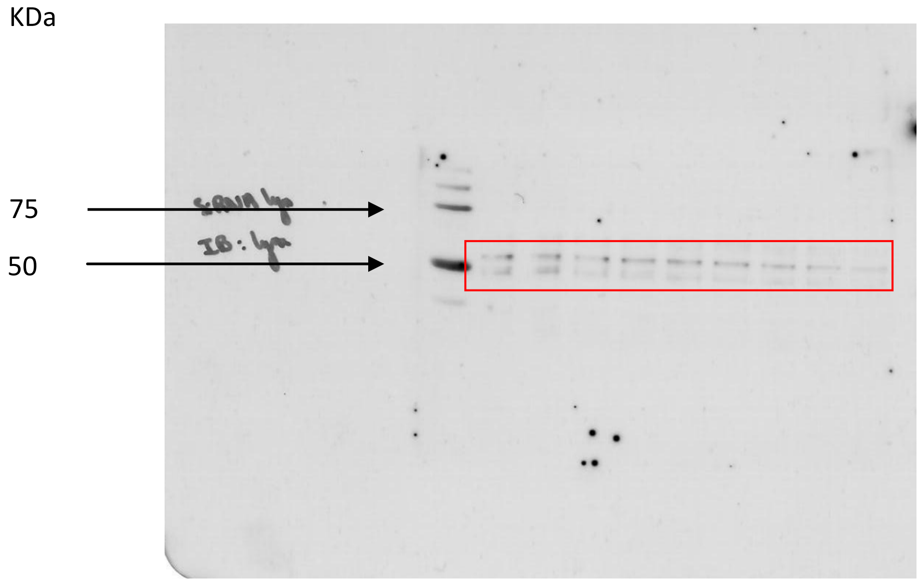

Supplementary Figure 1a

Lysate, IB: SHP-1 (Lyn siRNA)

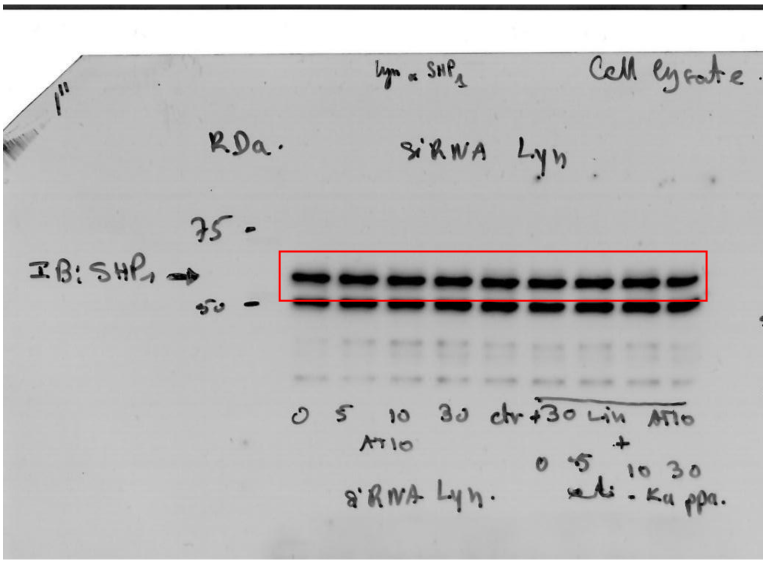

Supplementary Figure 1a

Lysate, IB: SHP-1 (Fyn siRNA)

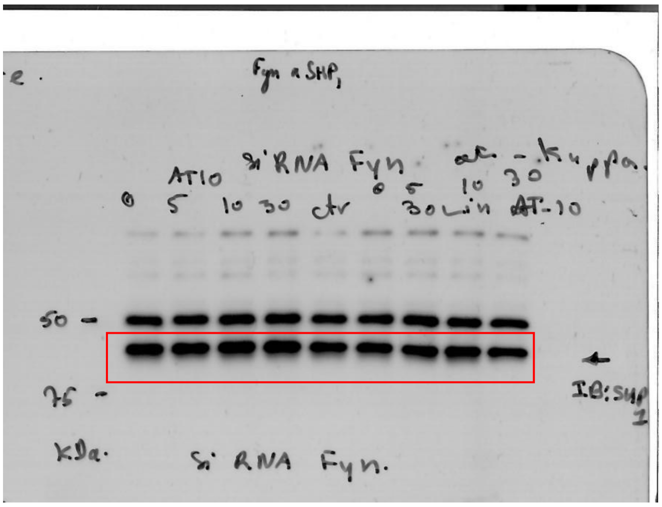

Supplementary Figure 1a

Lysate, IB: Syk (Lyn siRNA)

KDa

75

50

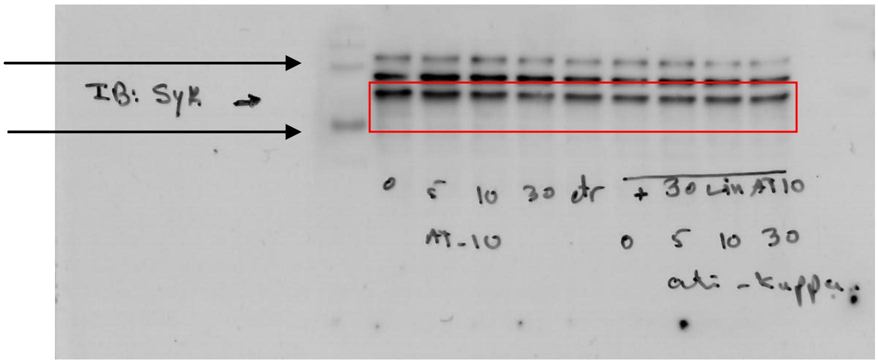

Supplementary Figure 1a

Lysate, IB: Syk (Fyn siRNA)

KDa

75

50

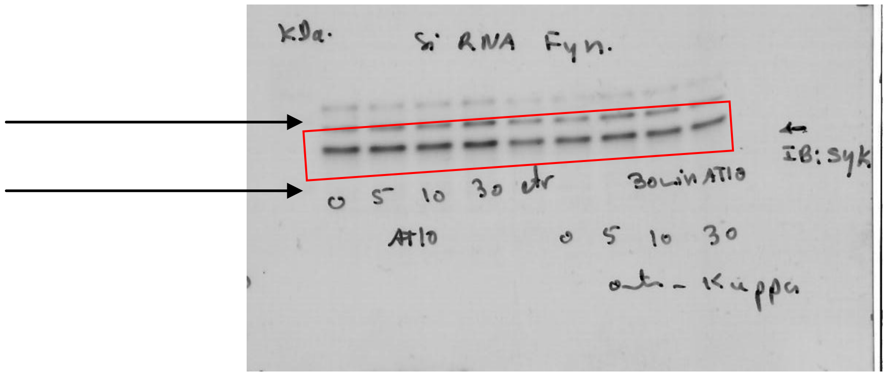

Supplementary Figure 1b

Lysate, IB: Lyn (Fyn siRNA)

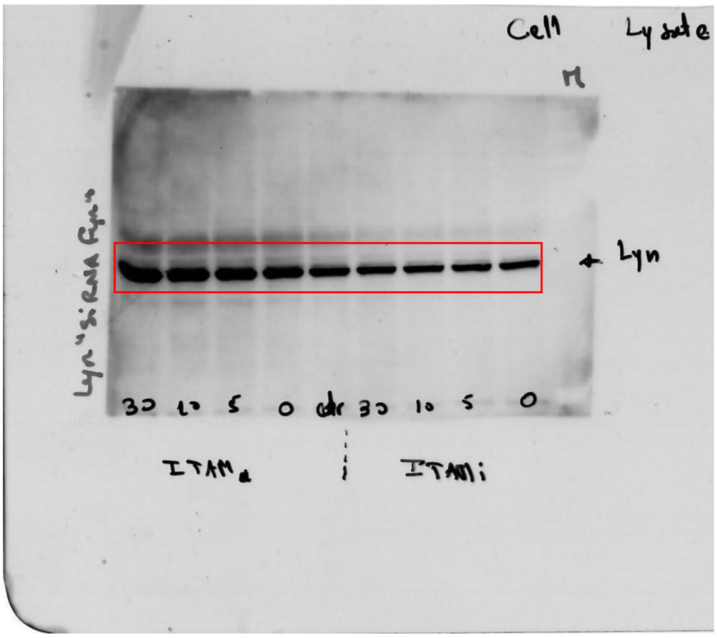

Supplementary Figure 1b

Lysate, IB: Lyn (Control siRNA)

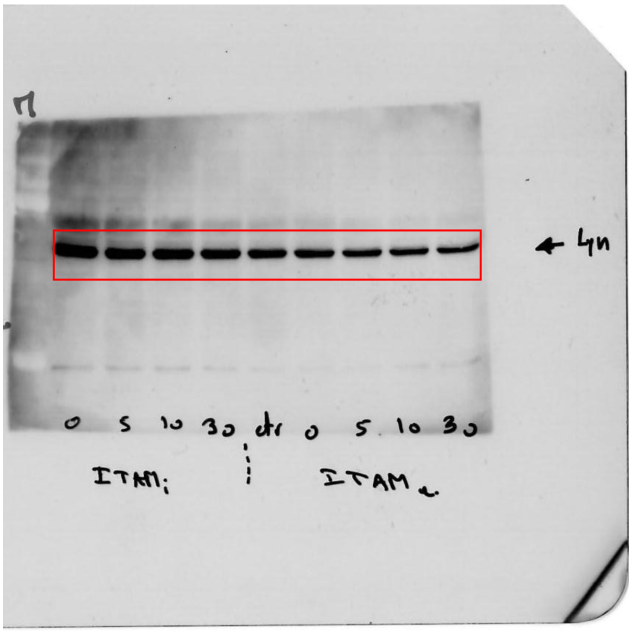

Supplementary Figure 1b

Lysate, IB: Fyn (Lyn siRNA)

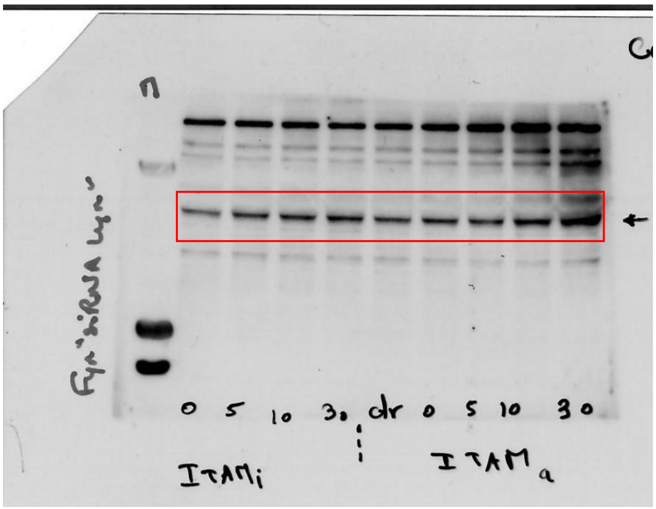

Supplementary Figure 1b

Lysate, IB: SHP-1 (Control siRNA)

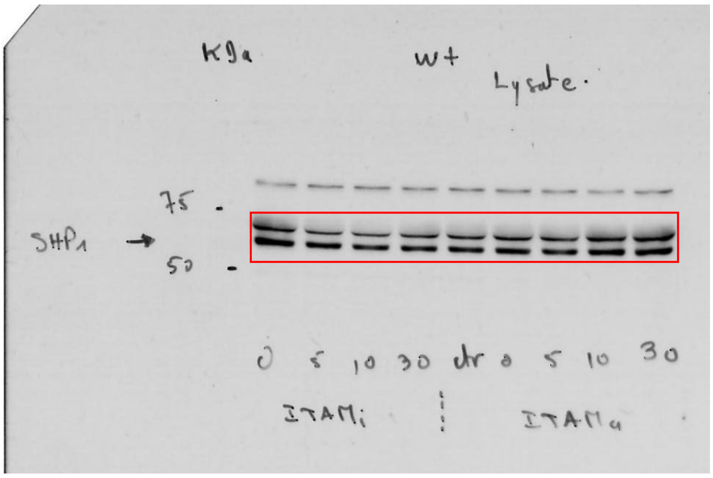

Supplementary Figure 1b

Lysate, IB: SHP-1 (Fyn siRNA)

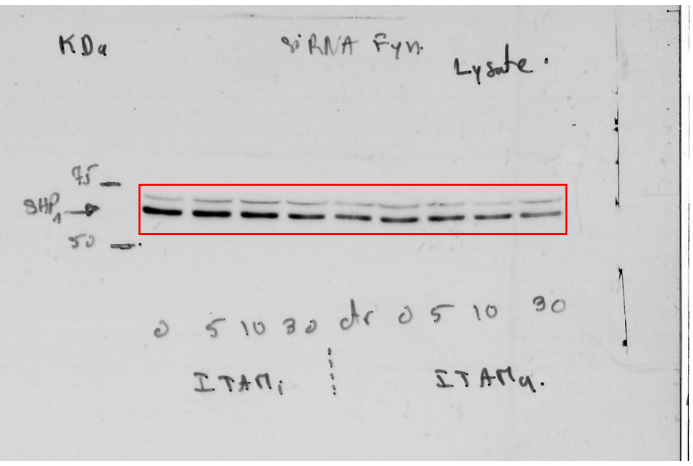

Supplementary Figure 1b

Lysate, IB: Syk (Control siRNA)

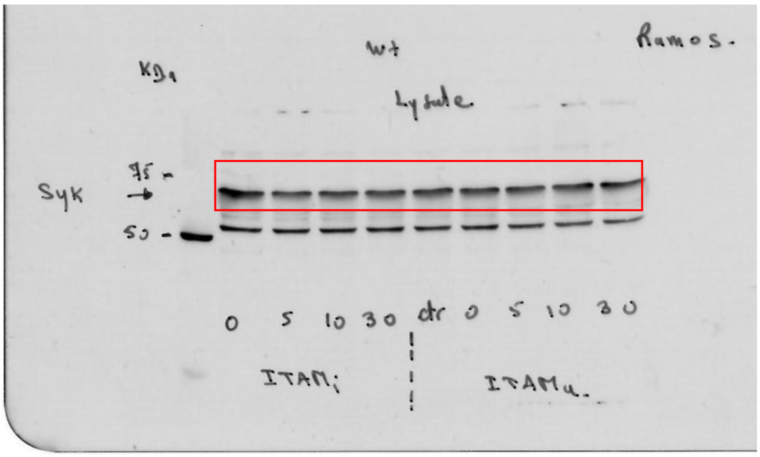

Supplementary Figure 1b

Lysate, IB: Syk (Fyn siRNA)

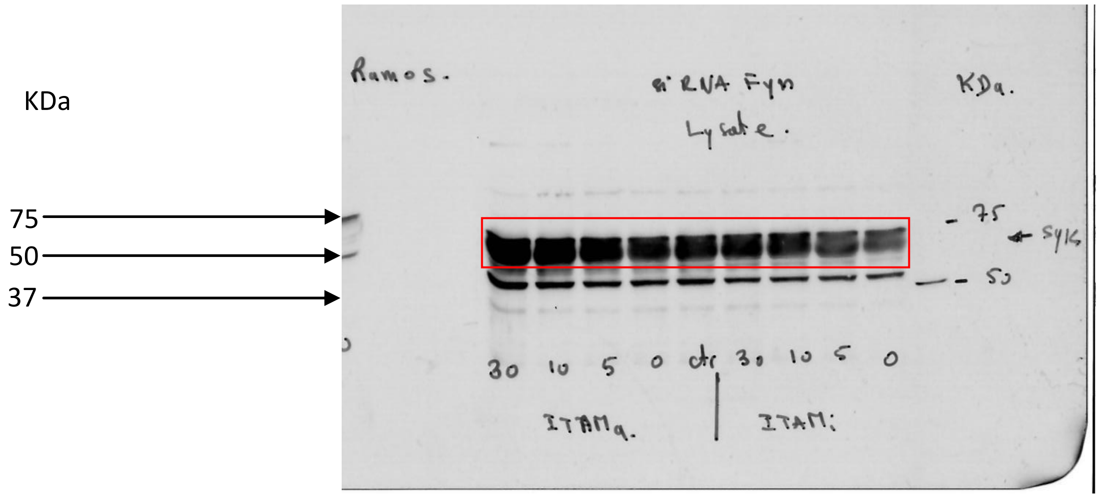

Supplementary Figure 1b

Lysate, IB: Syk (Lyn siRNA)

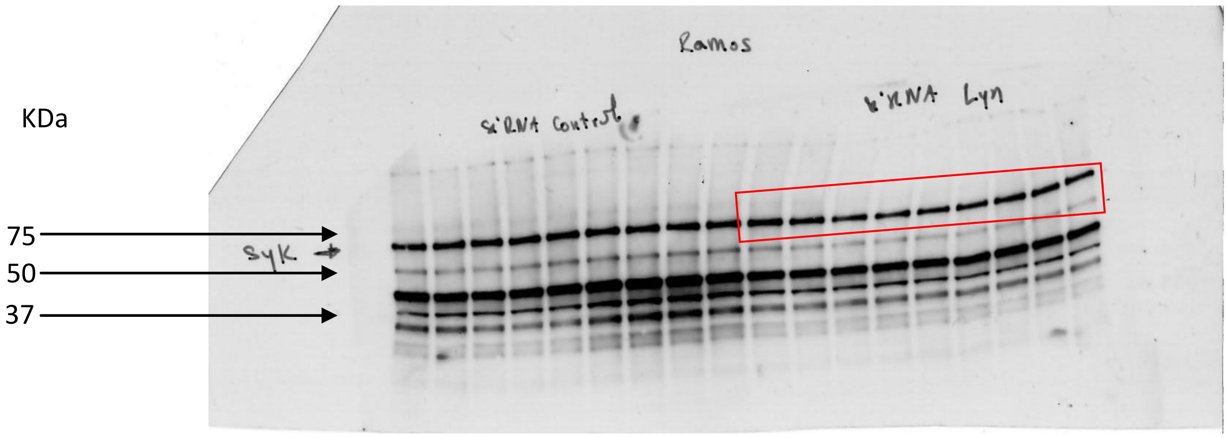

Supplementary Figure 1b

Lysate, IB: Fyn (Control siRNA)

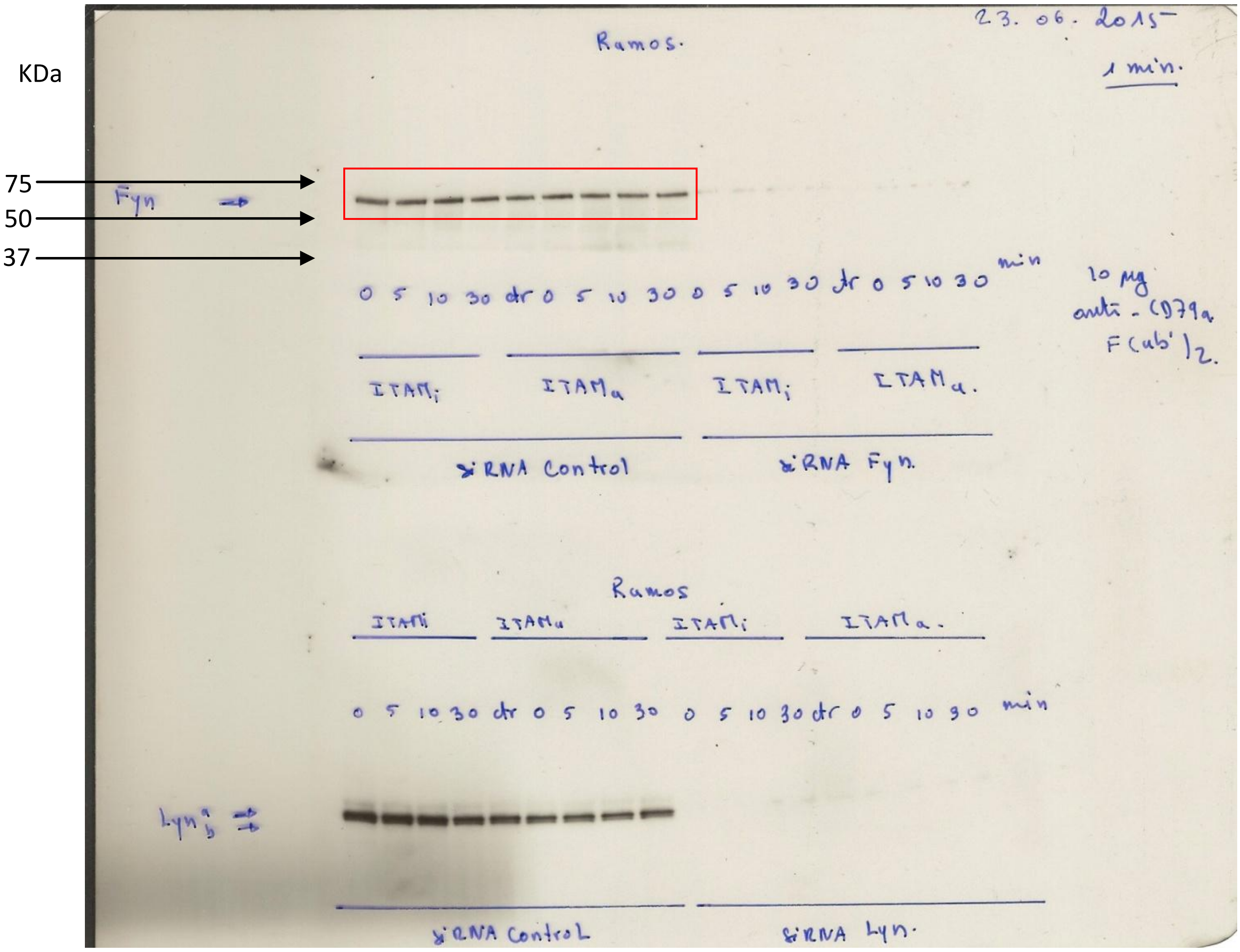

Supplementary Figure 1b

Lysate, IB: Fyn (Fyn siRNA)

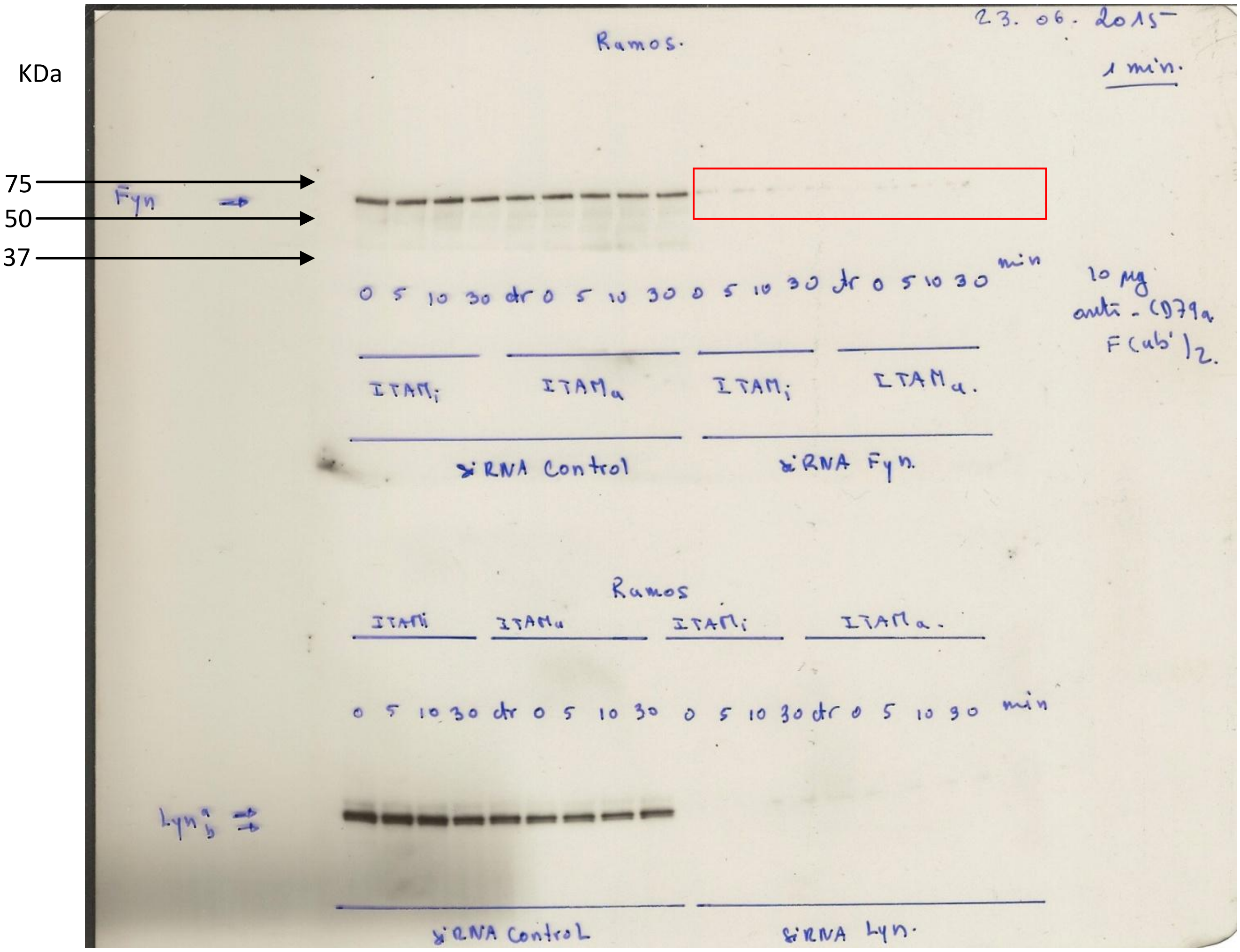

Supplementary Figure 1b

Lysate, IB: Lyn (Lyn siRNA)

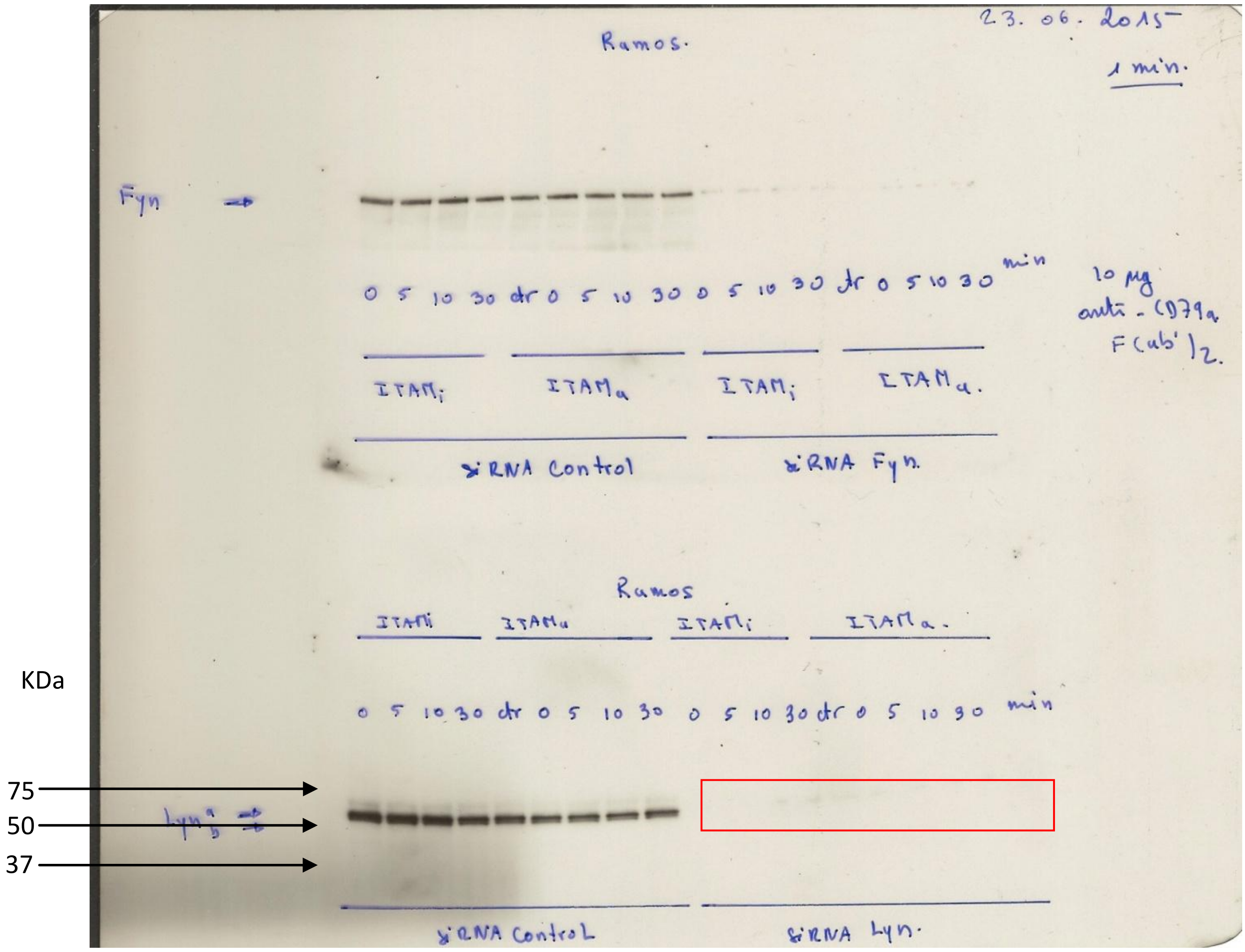

Supplementary Figure 1b

Lysate, IB: SHP-1 (Lyn siRNA)

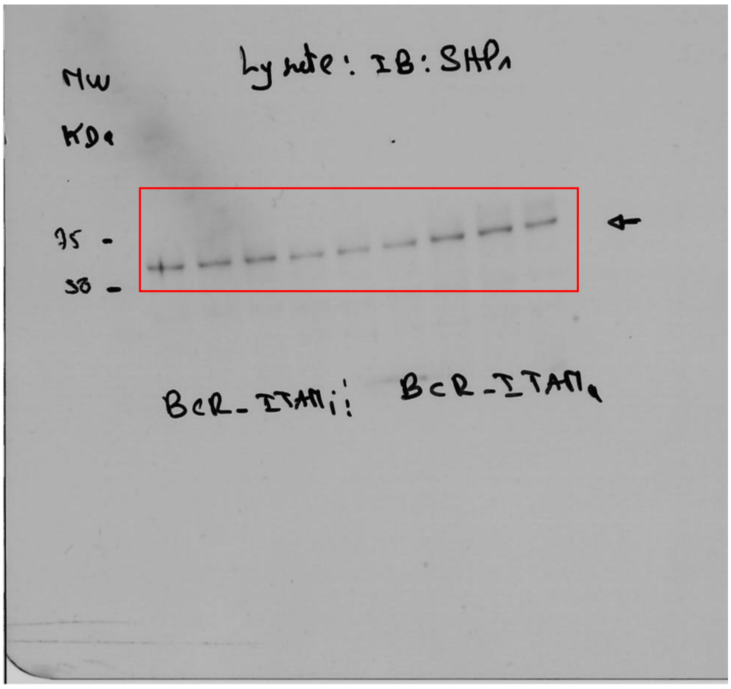

Supplementary Figure 2a

Lysate, IB: Lyn (control siRNA)

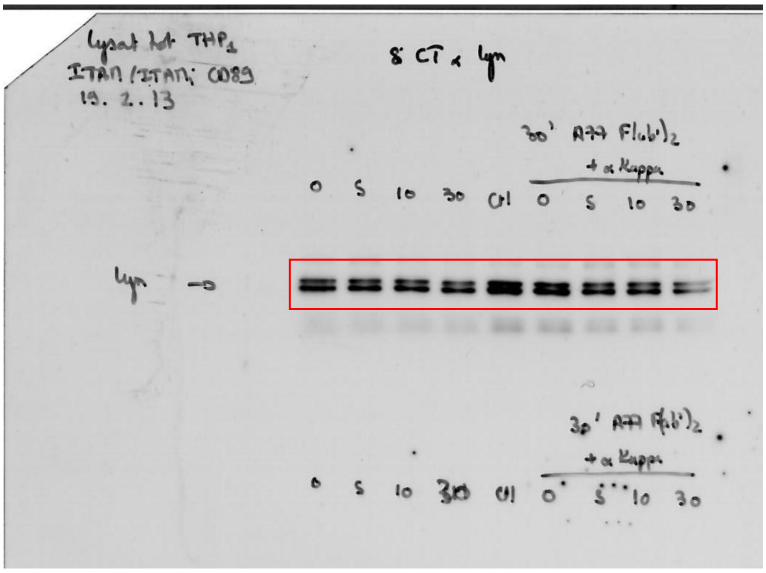

Supplementary Figure 2a

Lysate, IB: Lyn (Fyn siRNA)

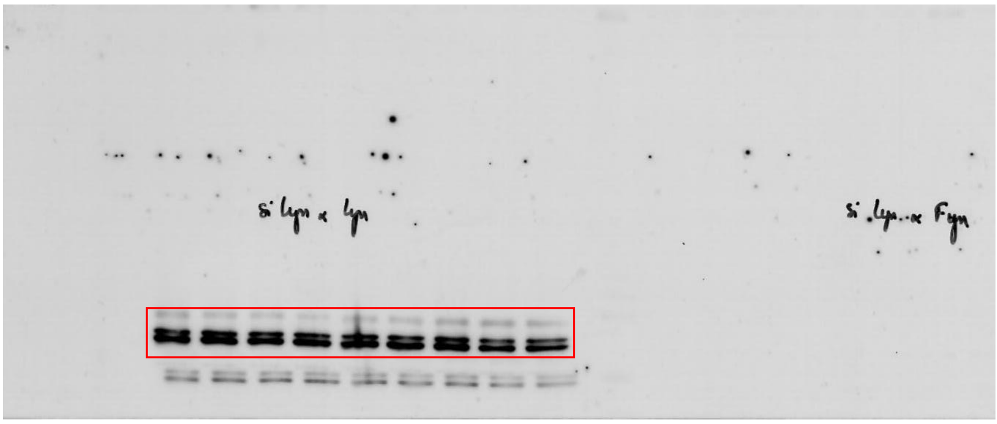

Supplementary Figure 2a

Lysate, IB: Fyn (Control siRNA)

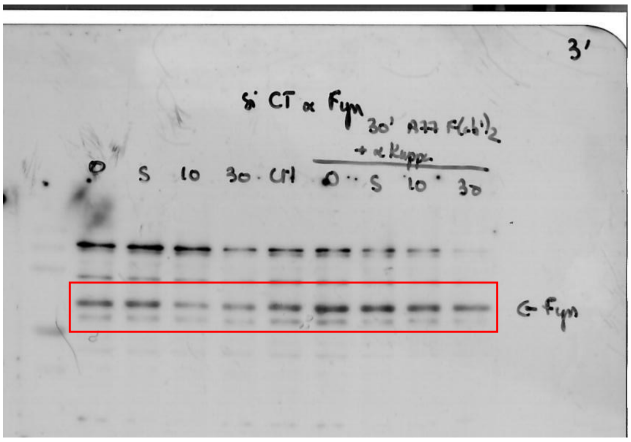

Supplementary Figure 2a

Lysate, IB: Fyn (Lyn siRNA)

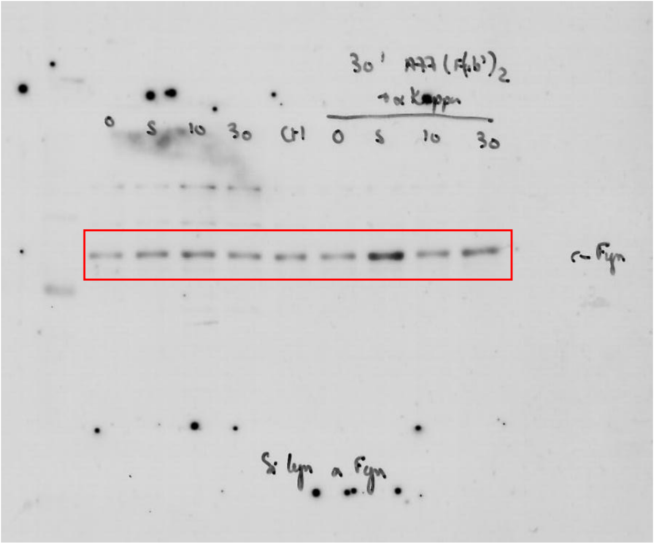

Supplementary Figure 2a

Lysate, IB: Lyn (Lyn siRNA)

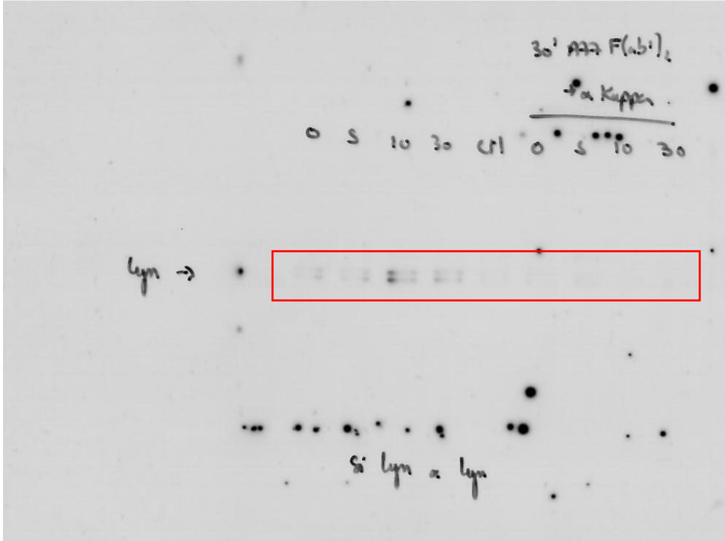

Supplementary Figure 2a

Lysate, IB: SHP1 (control siRNA)

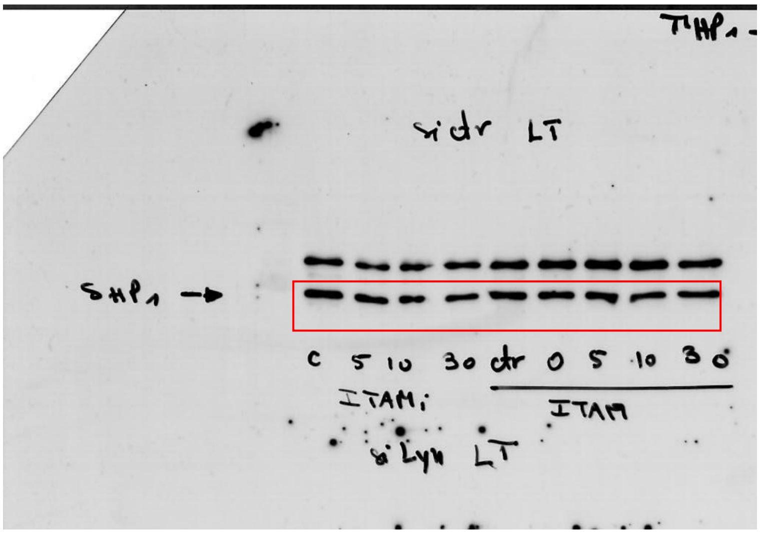

Supplementary Figure 2a

Lysate, IB: SHP1 (Lyn siRNA)

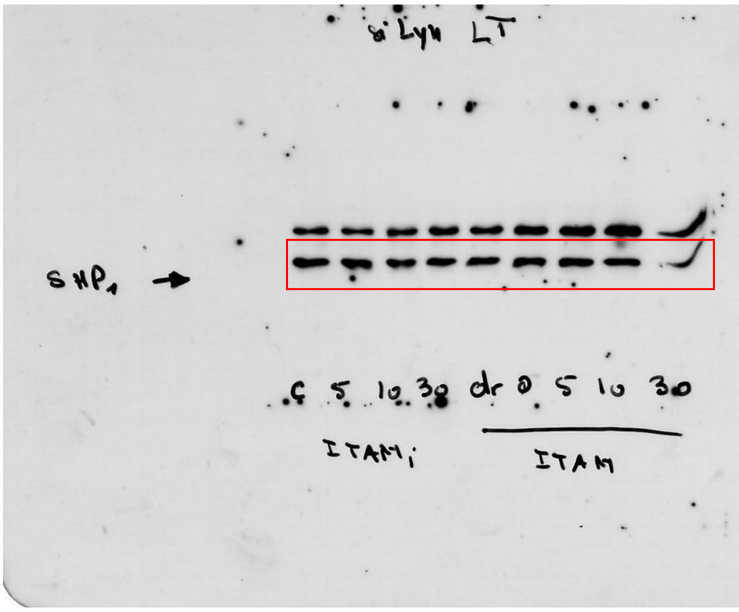

Supplementary Figure 2a

Lysate, IB: SHP1 (Fyn siRNA)

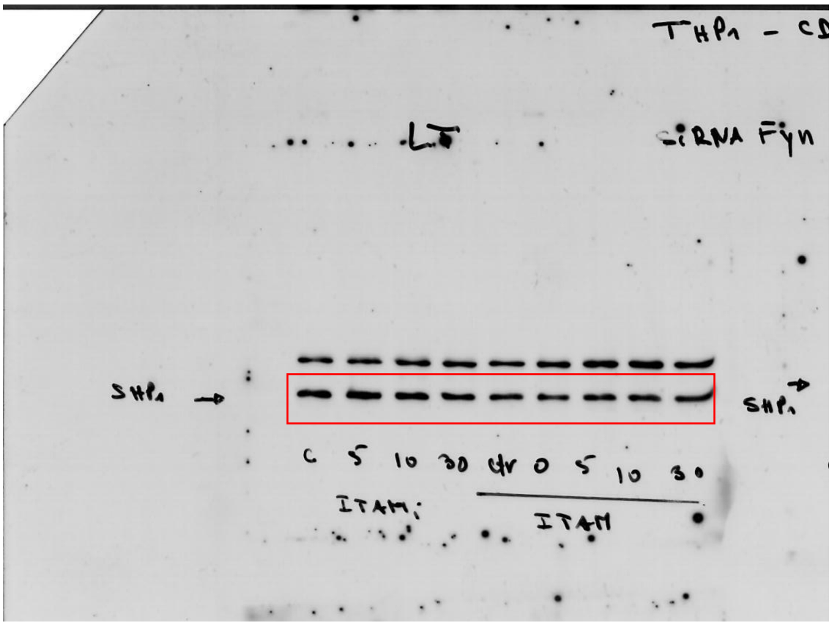

Supplementary Figure 2a

IP: FcαRI, IB: Syk (Fyn siRNA)

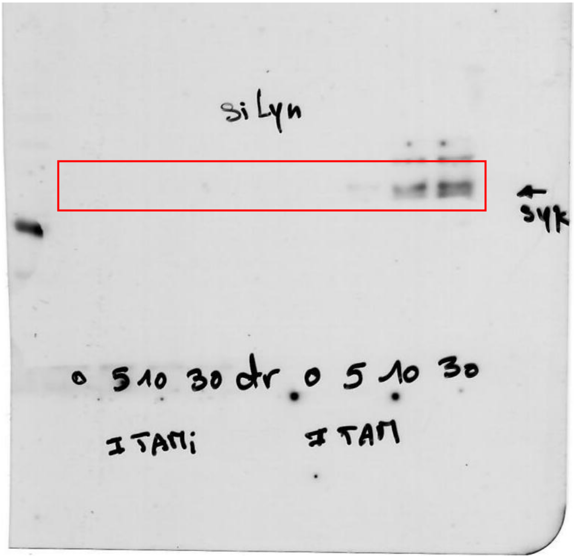

Supplementary Figure 2a

IP: FcαRI, IB: SHP1 (Fyn siRNA)

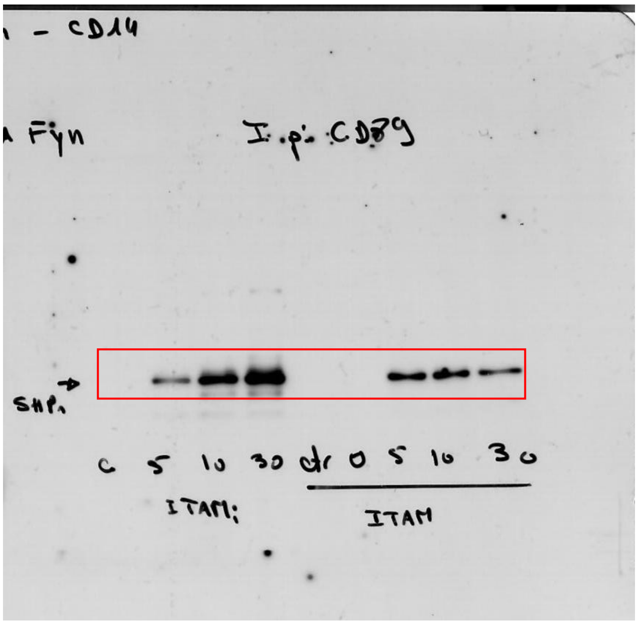

Supplementary Figure 2a

Lysate, IB: Syk (Control siRNA)

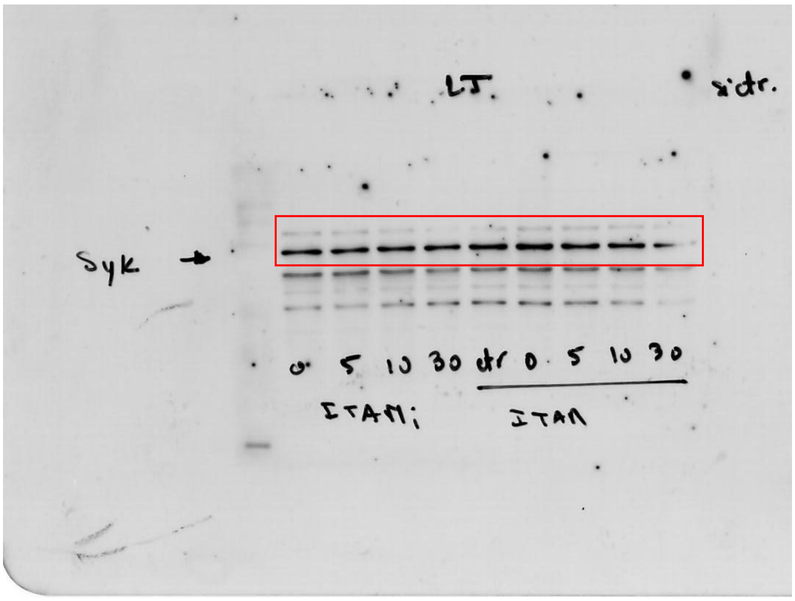

Supplementary Figure 2a

IP: FcαRI, IB: Fyn (Control siRNA)

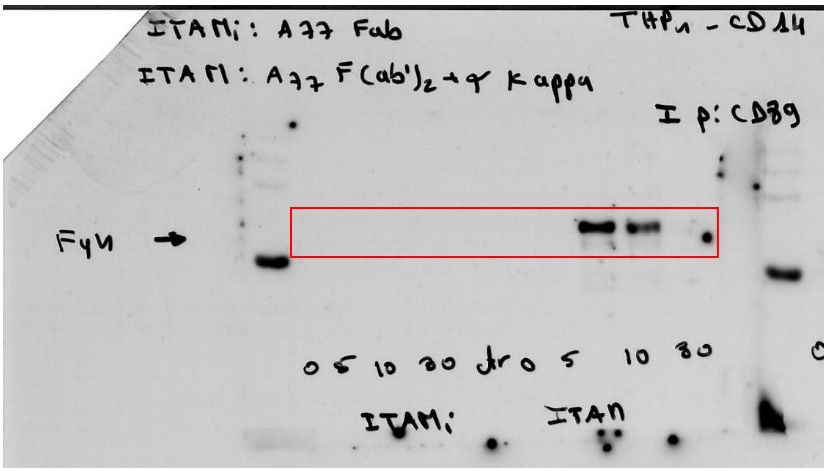

Supplementary Figure 2a

IP: FcαRI, IB: Syk (Control siRNA)

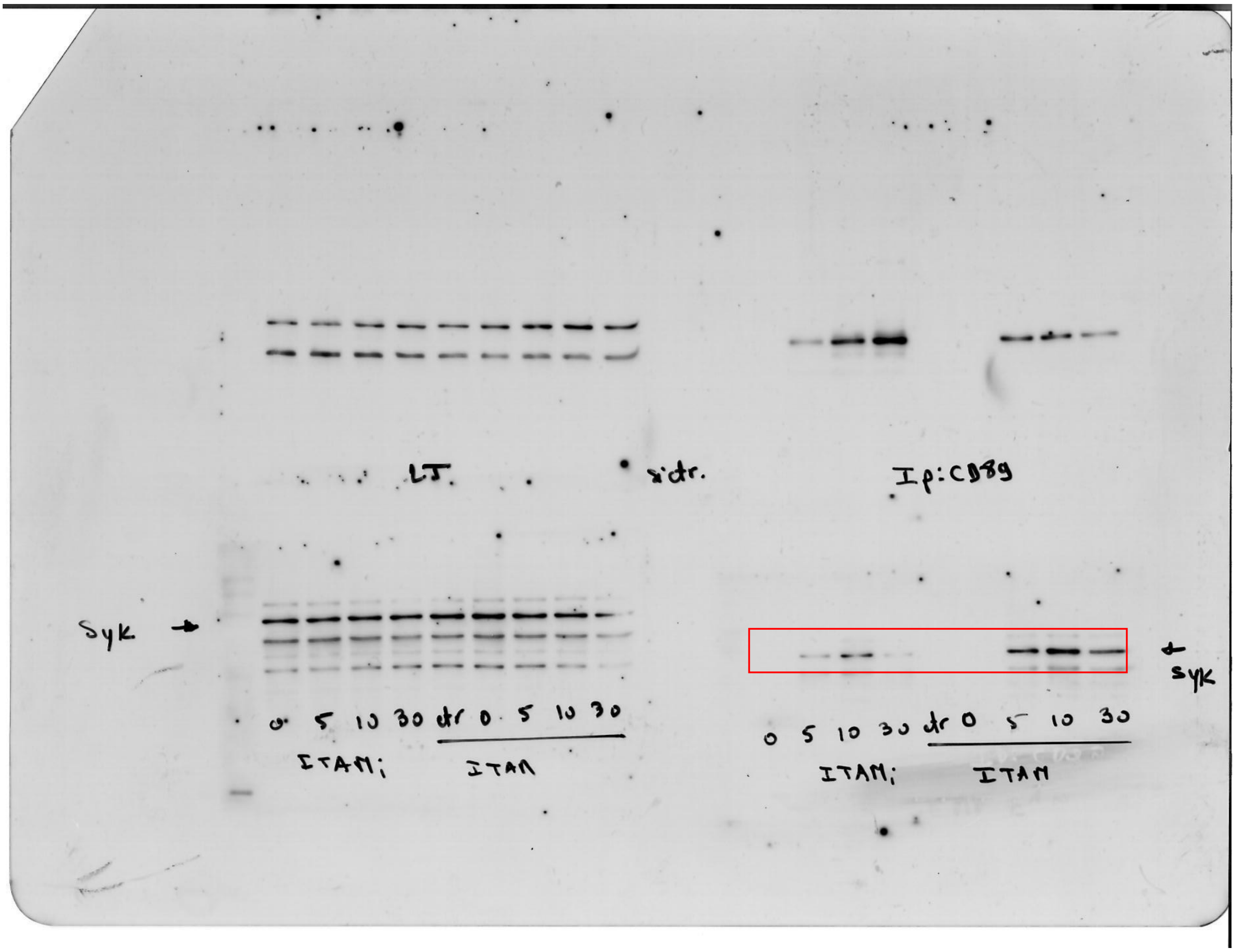

Supplementary Figure 2a

IP: FcαRI, IB: Lyn (Control siRNA)

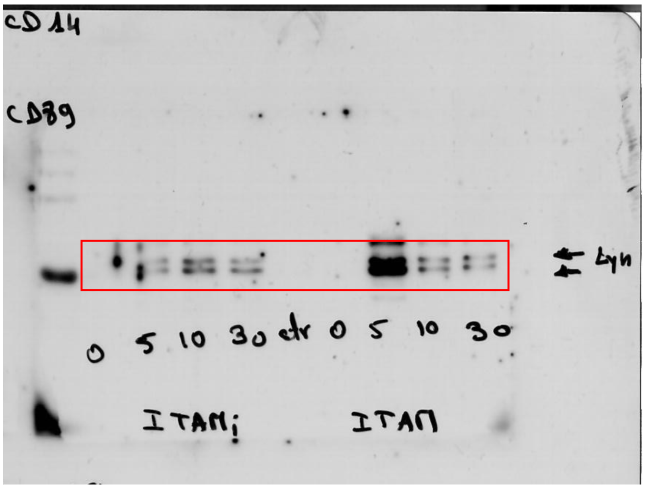

Supplementary Figure 2a

IP: FcαRI, IB: Fyn (Lyn siRNA)

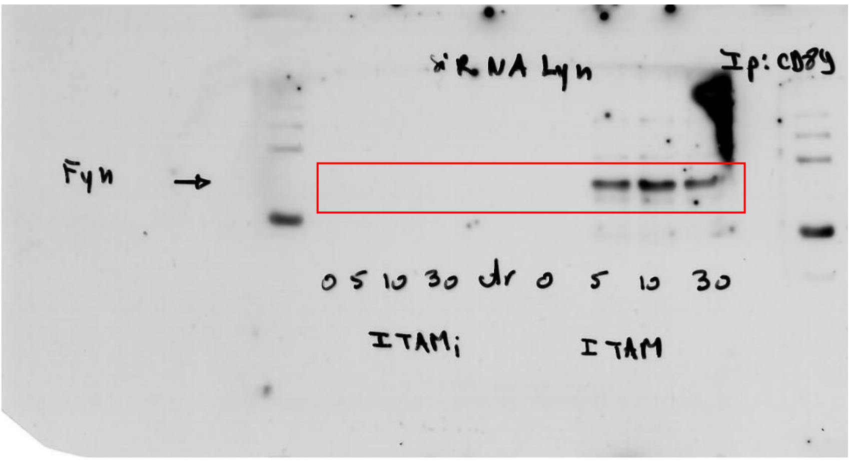

Supplementary Figure 2a

IP: FcαRI, IB: Lyn (Lyn siRNA)

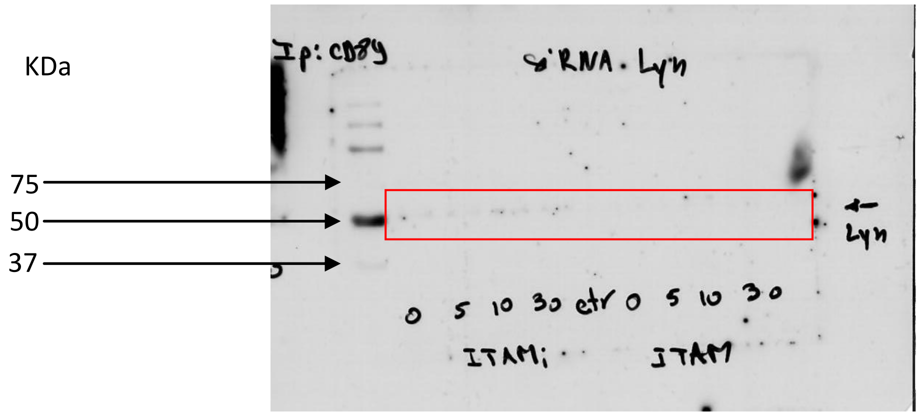

Supplementary Figure 2a

IP: FcαRI, IB: Lyn (Fyn siRNA)

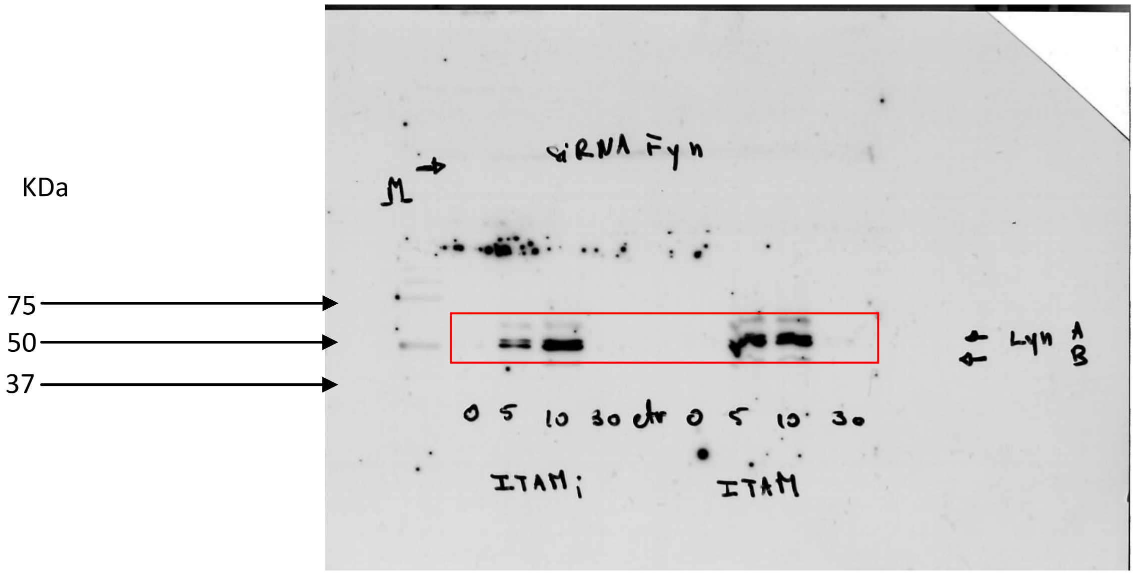

Supplementary Figure 2a

IP: FcαRI, IB: Fyn (Fyn siRNA)

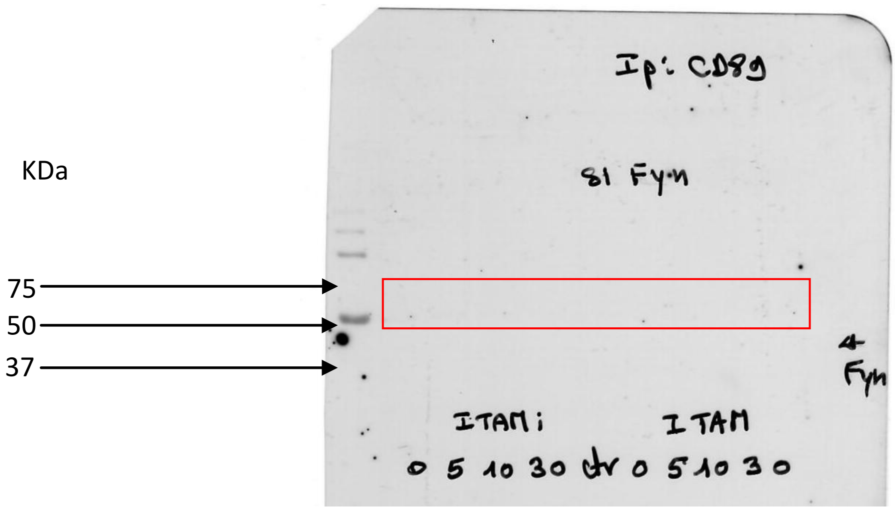

Supplementary Figure 2a

Lysate, IB: Fyn (Fyn siRNA)

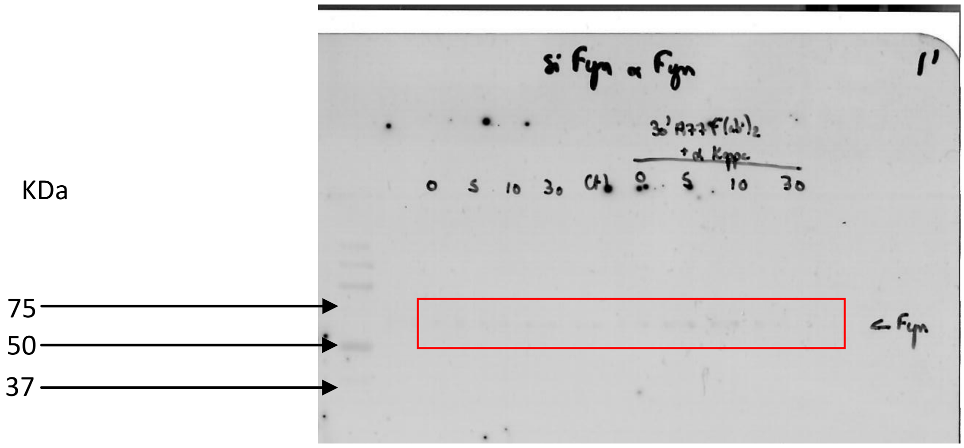

Supplementary Figure 2a

IP: FcαRI, IB: Syk (Fyn siRNA)

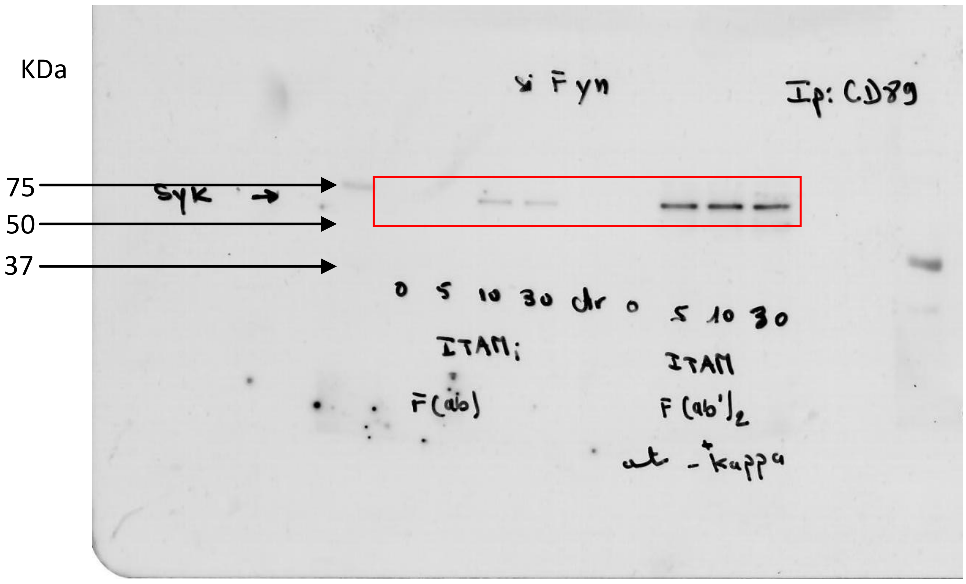

Supplementary Figure 2a

Lysate, IB: Syk (Fyn siRNA)

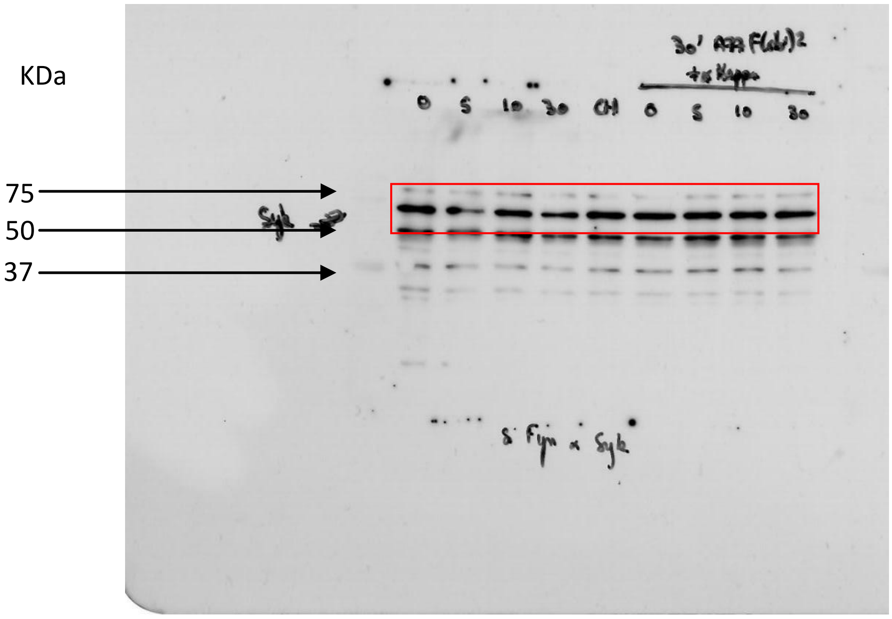

Supplementary Figure 2a

Lysate, IB: Syk (Lyn siRNA)

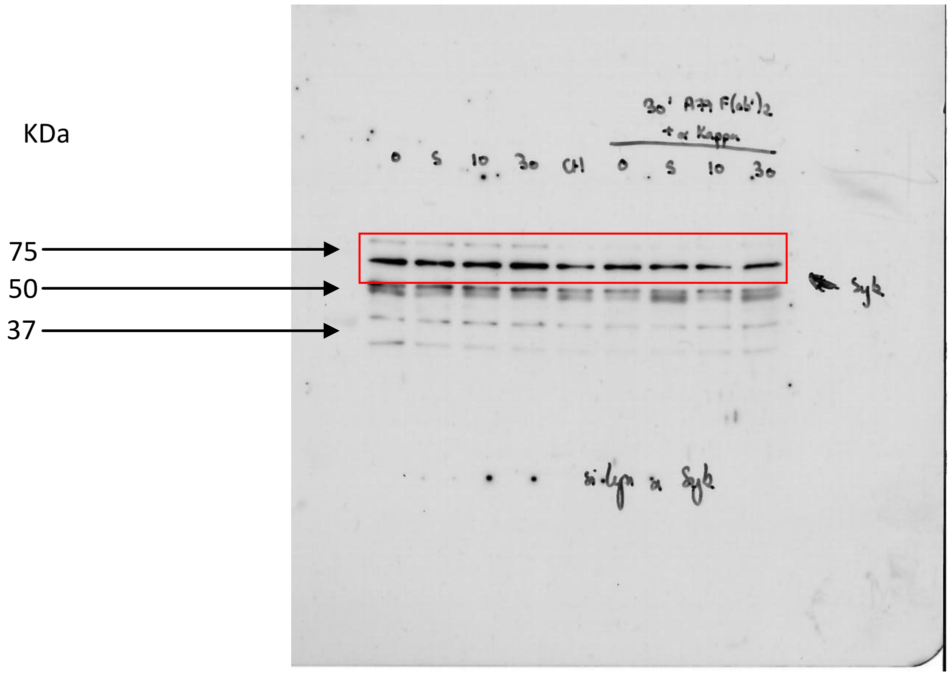

Supplementary Figure 2a

IP: FcαRI, IB: SHP-1 (Control siRNA)

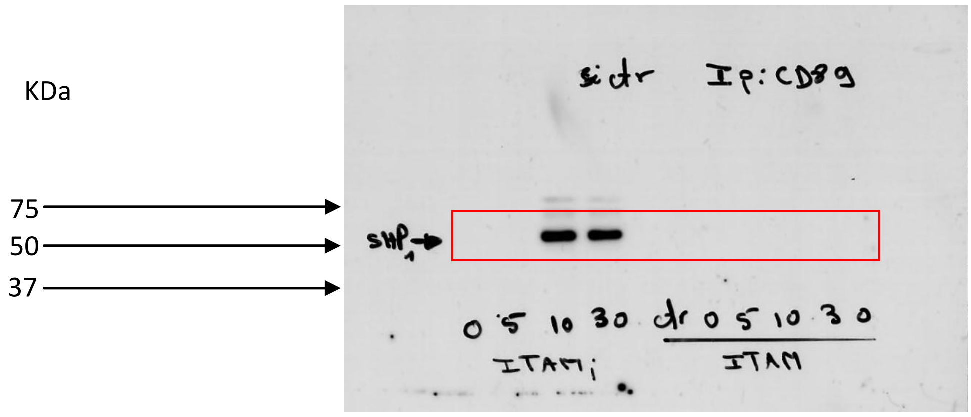

Supplementary Figure 2a

IP: FcαRI, IB: SHP-1 (Lyn siRNA)

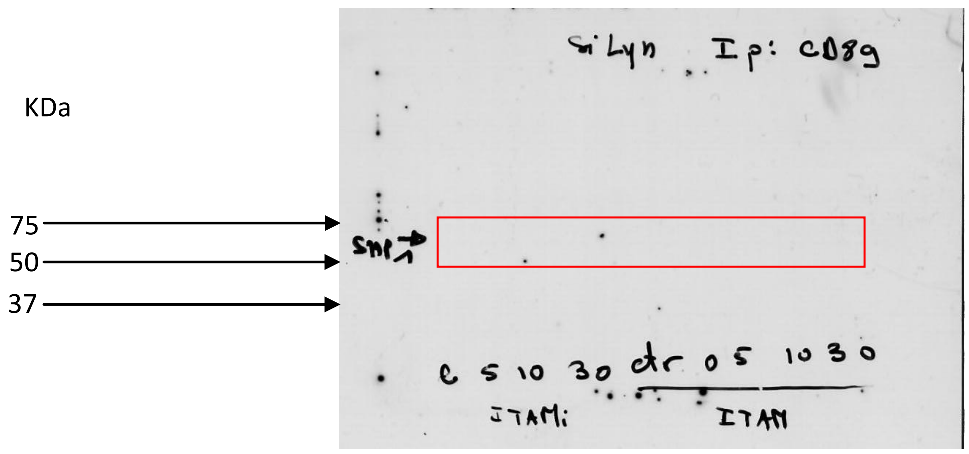

Supplementary Figure 3b

Lysate, IB: SHP-1 (BCR-ITAMi)

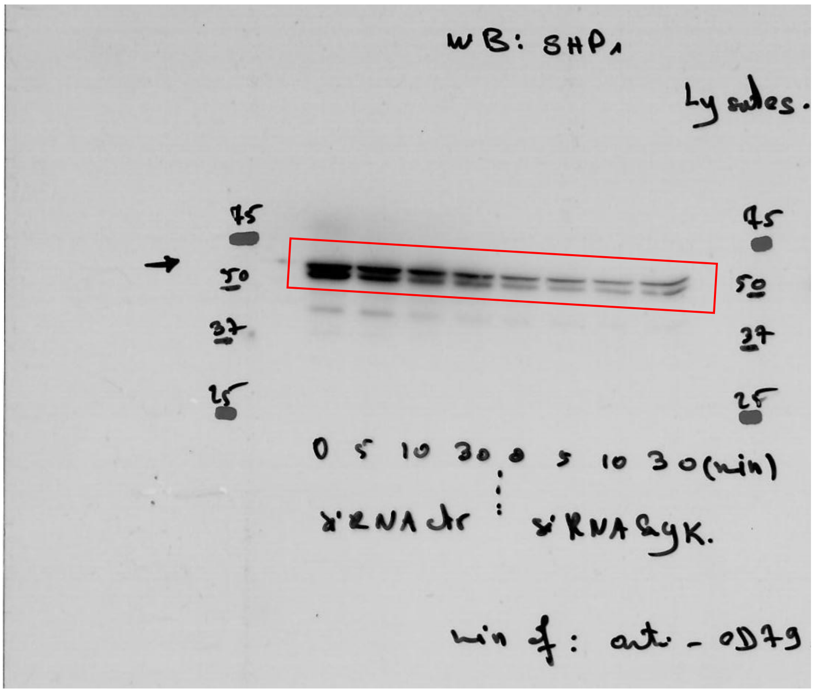

Supplementary Figure 3b

Lysate, IB: Lyn (BCR-ITAMi)

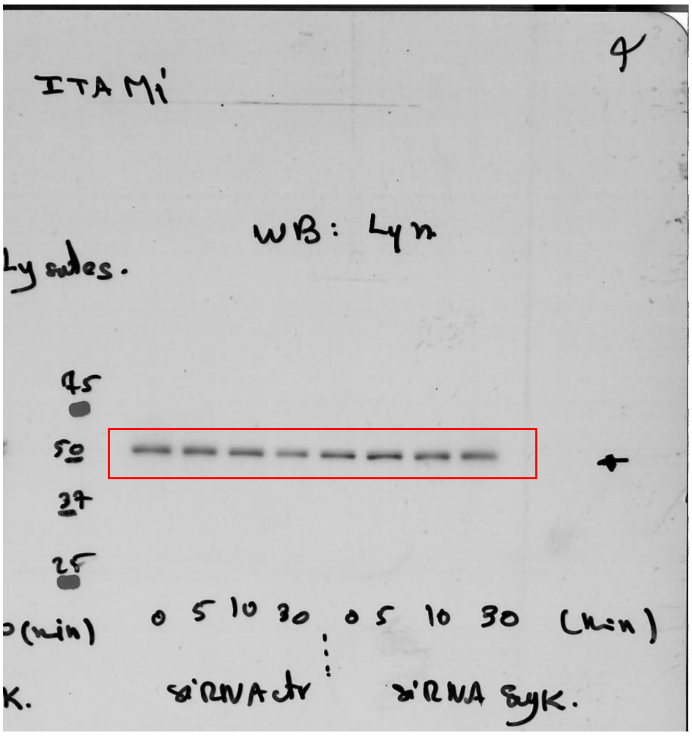

Supplementary Figure 3b

IP: CD79a, IB: Fyn (BCR-ITAMi)

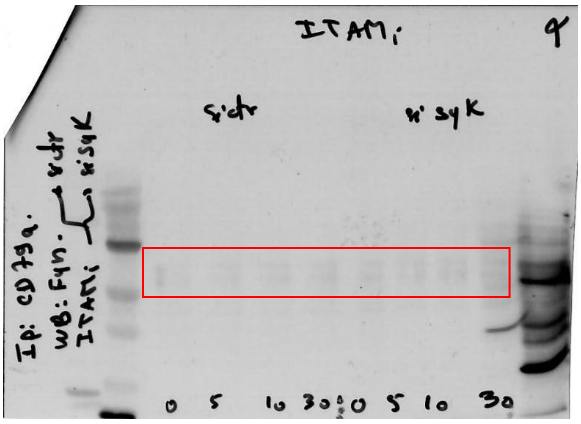

Supplementary Figure 3b

Lysate, IB: Fyn (BCR-ITAMi)

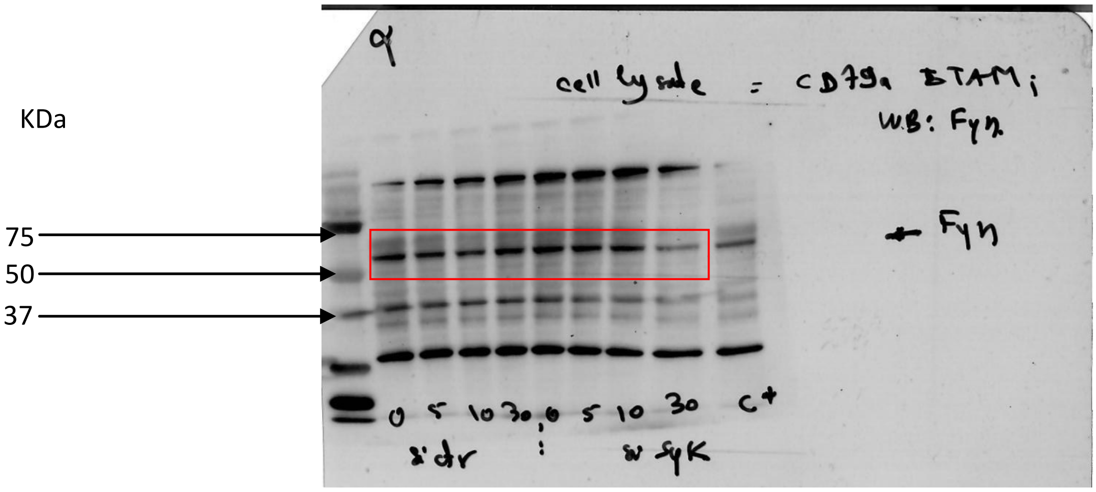

Supplementary Figure 3b

IP: CD79a, IB: Syk (BCR-ITAMi)

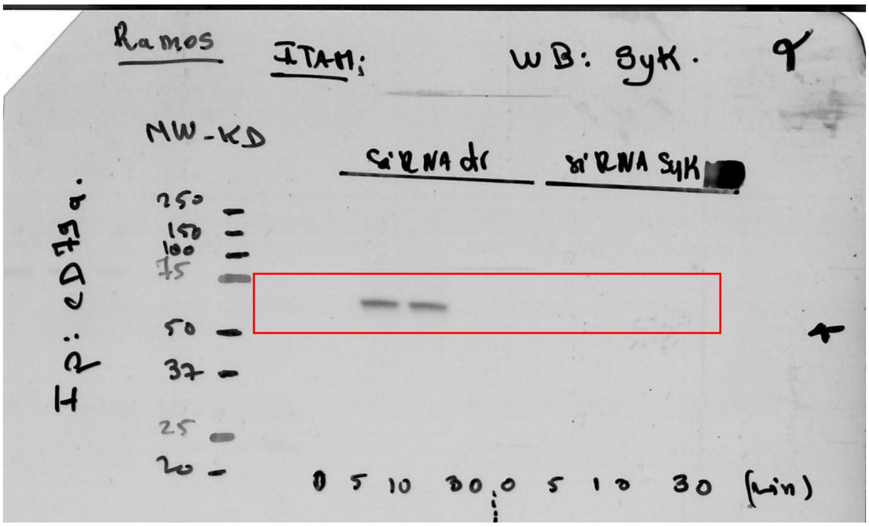

Supplementary Figure 3b

Lysate, IB: Syk (BCR-ITAMi)

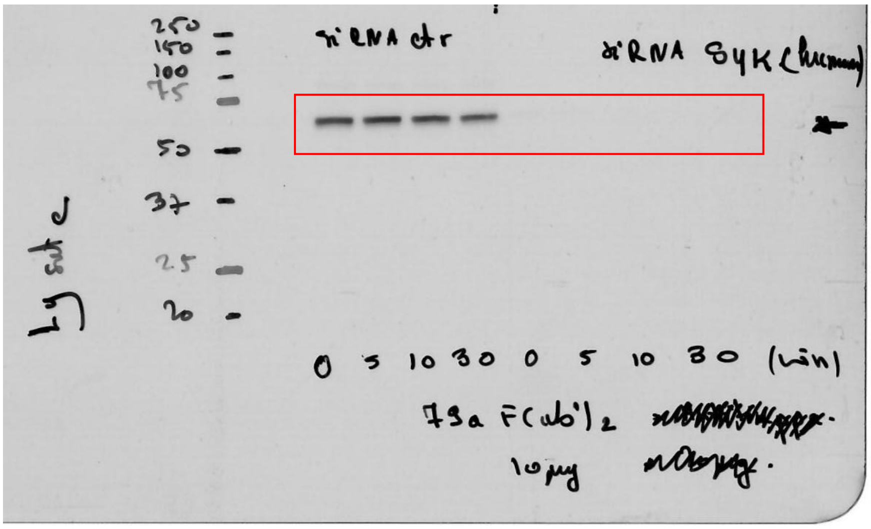

Supplementary Figure 3b

IP: CD79a, IB: SHP-1 (BCR-ITAM)

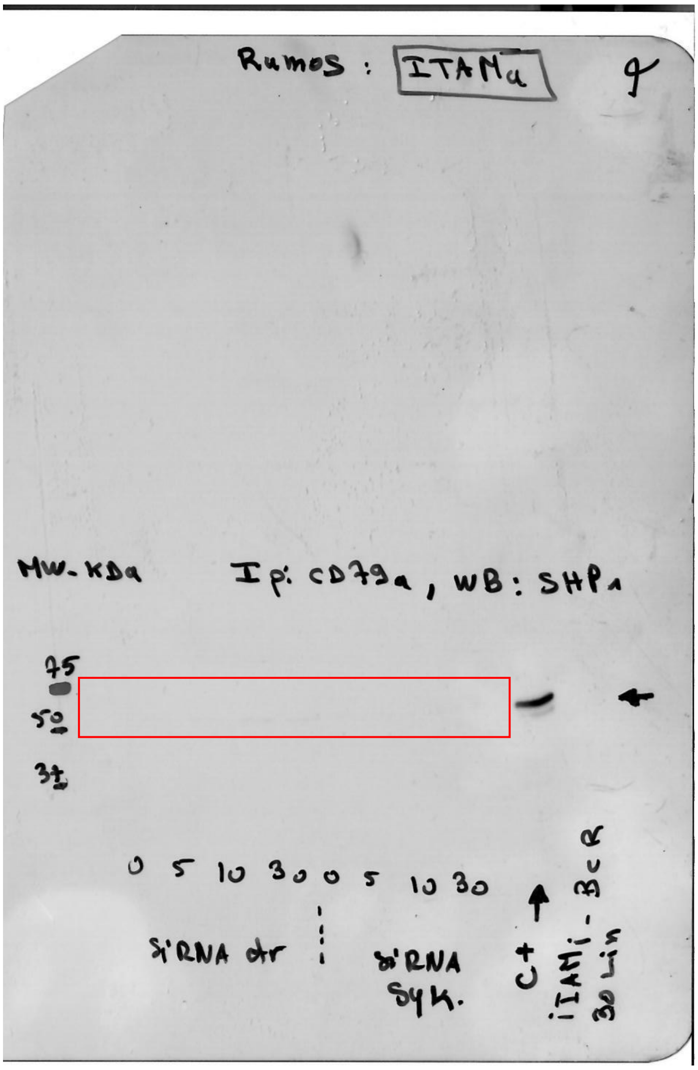

Supplementary Figure 3b

IP: CD79a, IB: Fyn (BCR-ITAM)

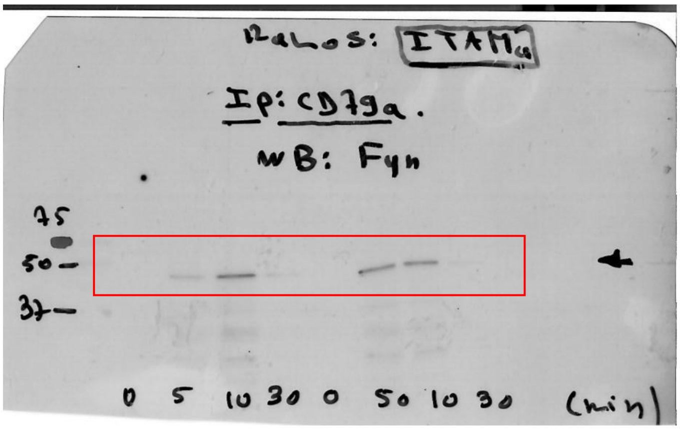

Supplementary Figure 3b

IP: CD79a, IB: Syk (BCR-ITAM)

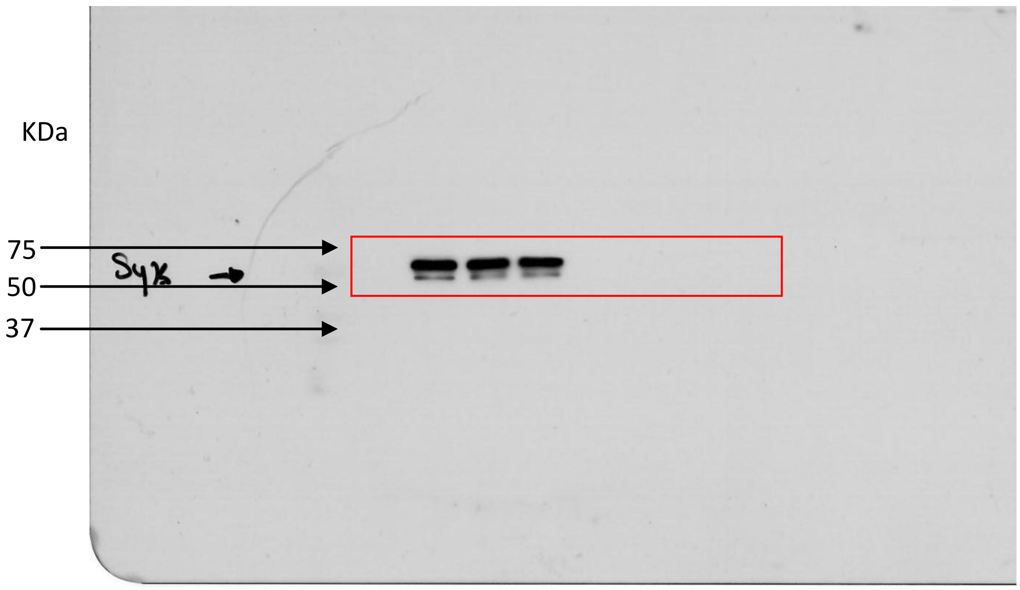

Supplementary Figure 3b

IP: CD79a, IB: Lyn (BCR-ITAMi)

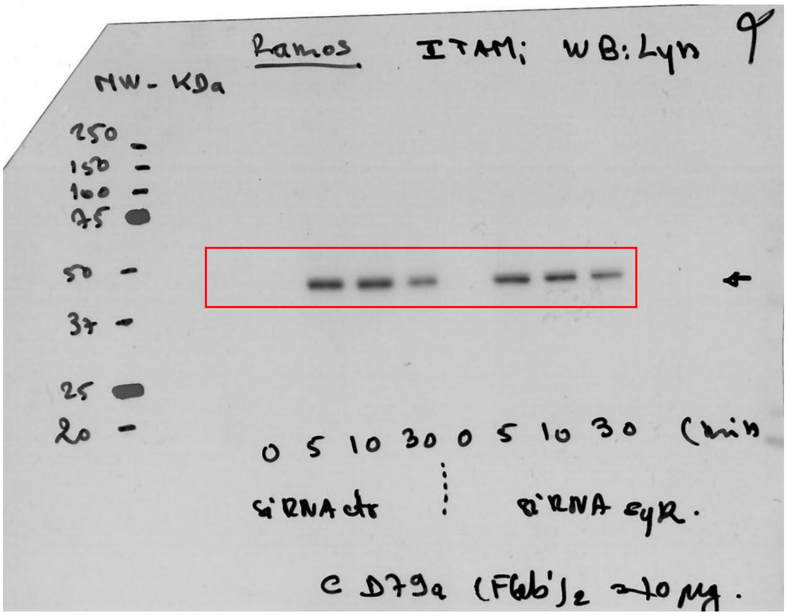

Supplementary Figure 3b

IP: CD79a, IB: SHP-1 (BCR-ITAMi)

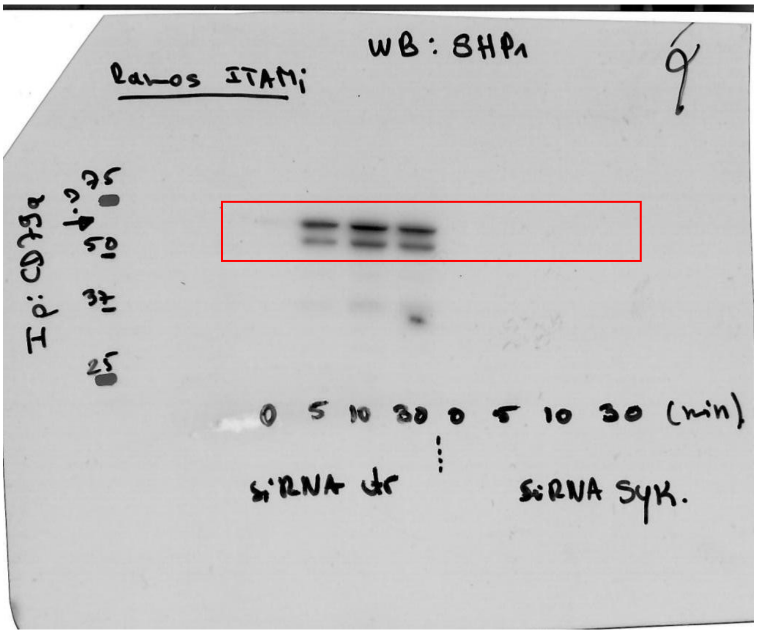

Supplementary Figure 3b

Lysate, IB: Lyn (BCR-ITAM)

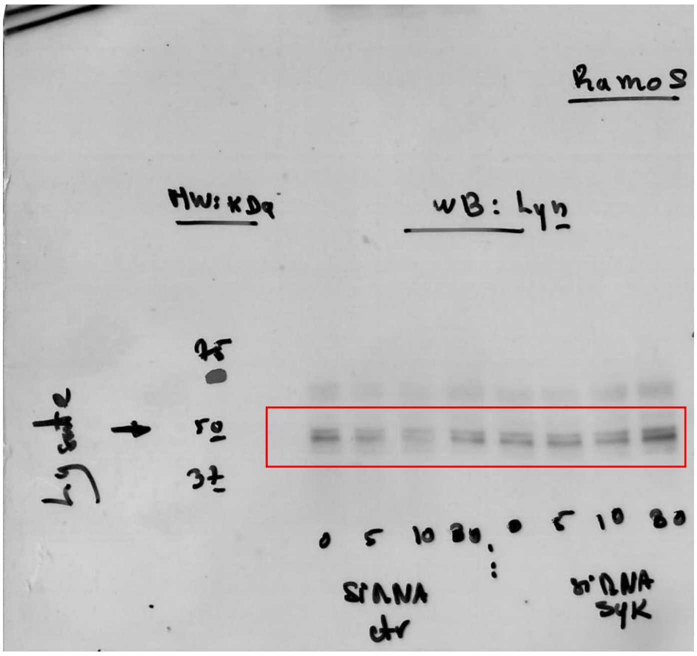

Supplementary Figure 3b

Lysate, IB: Fyn (BCR-ITAM)

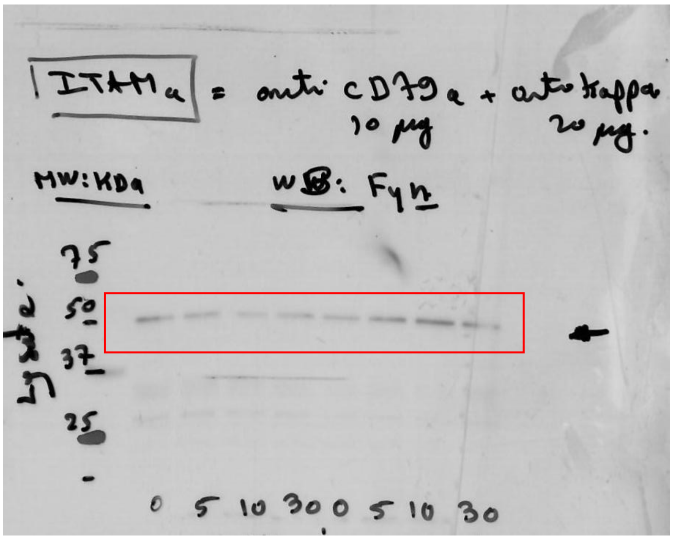

Supplementary Figure 3b

Lysate, IB: SHP-1 (BCR-ITAM)

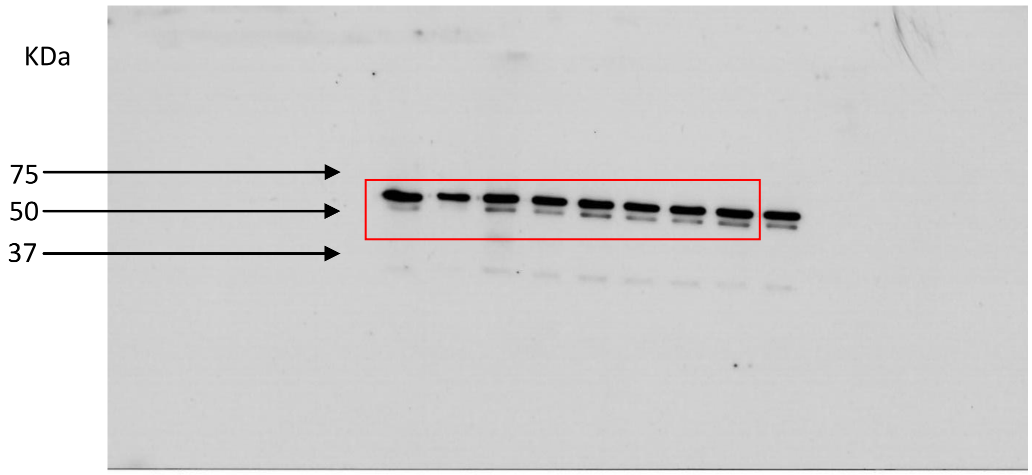

Supplementary Figure 3b

Lysate, IB: Syk (BCR-ITAM)

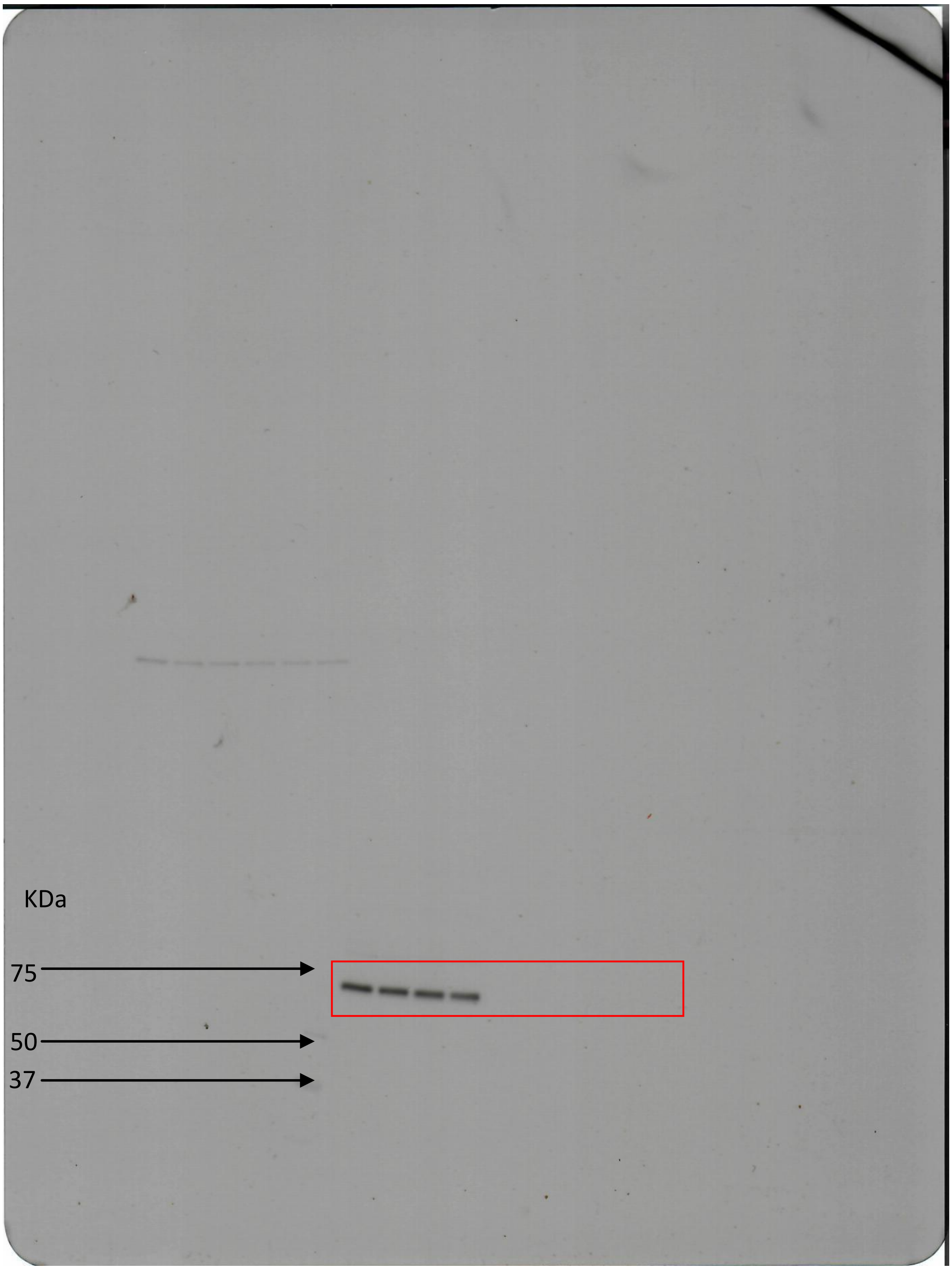

Supplementary Figure 3b

IP: CD79a, IB: Lyn (BCR-ITAM)

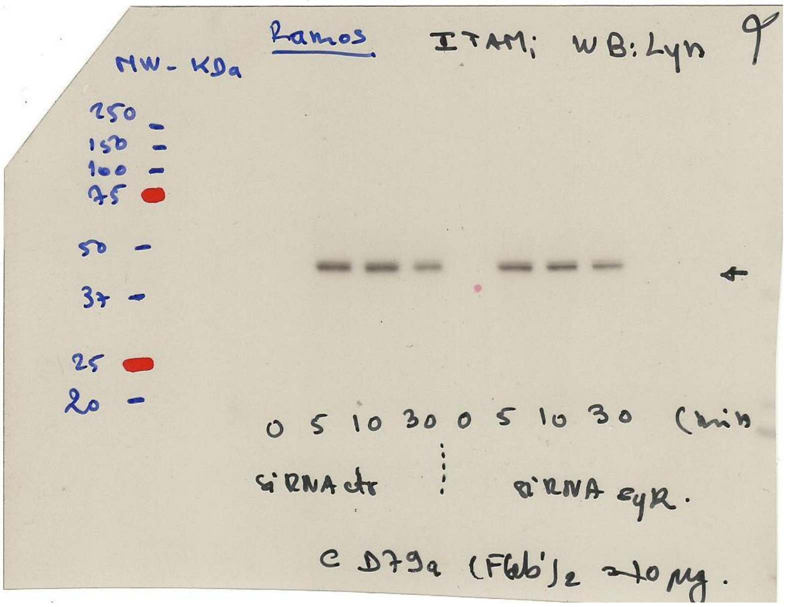

Supplementary Figure 3a

Lysate, IB: Fyn (FcγRIIA-ITAM)

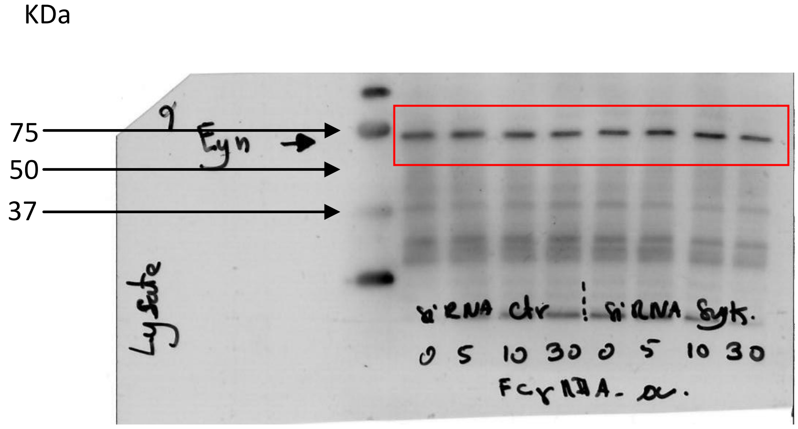

Supplementary Figure 3a

Lysate, IB: Lyn (FcγRIIA-ITAM)

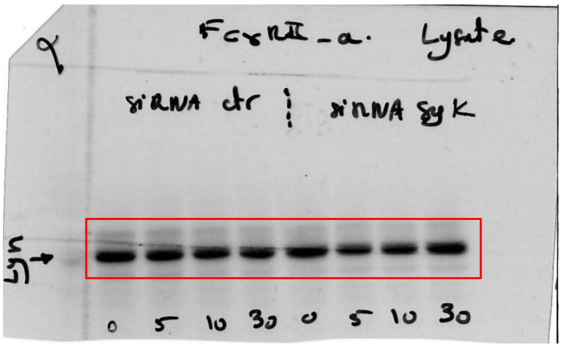

Supplementary Figure 3a

Lysate, IB: Lyn (FcγRIIA-ITAMi)

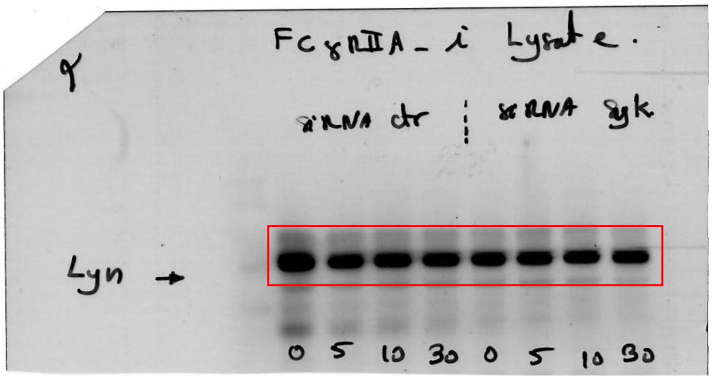

Supplementary Figure 3a

IP: FcγRIIA, IB: Lyn (FcγRIIA-ITAM)

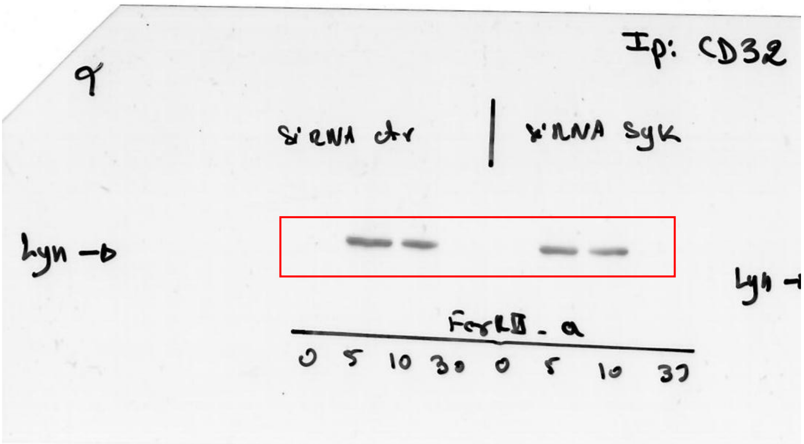

Supplementary Figure 3a

IP: FcγRIIA, IB: Lyn (FcγRIIA-ITAM)

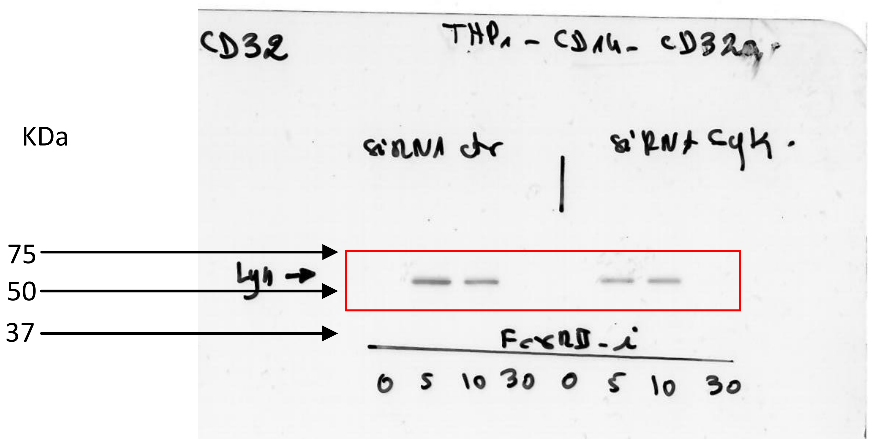

Supplementary Figure 3a

IP: FcγRIIA, IB: Fyn (FcγRIIA-ITAM)

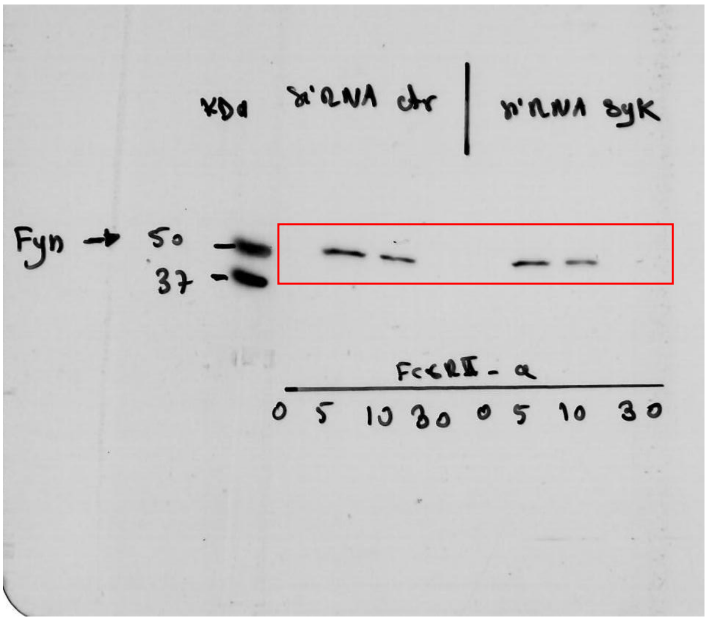

Supplementary Figure 3a

IP: FcγRIIA, IB: SHP-1 (FcγRIIA-ITAMi)

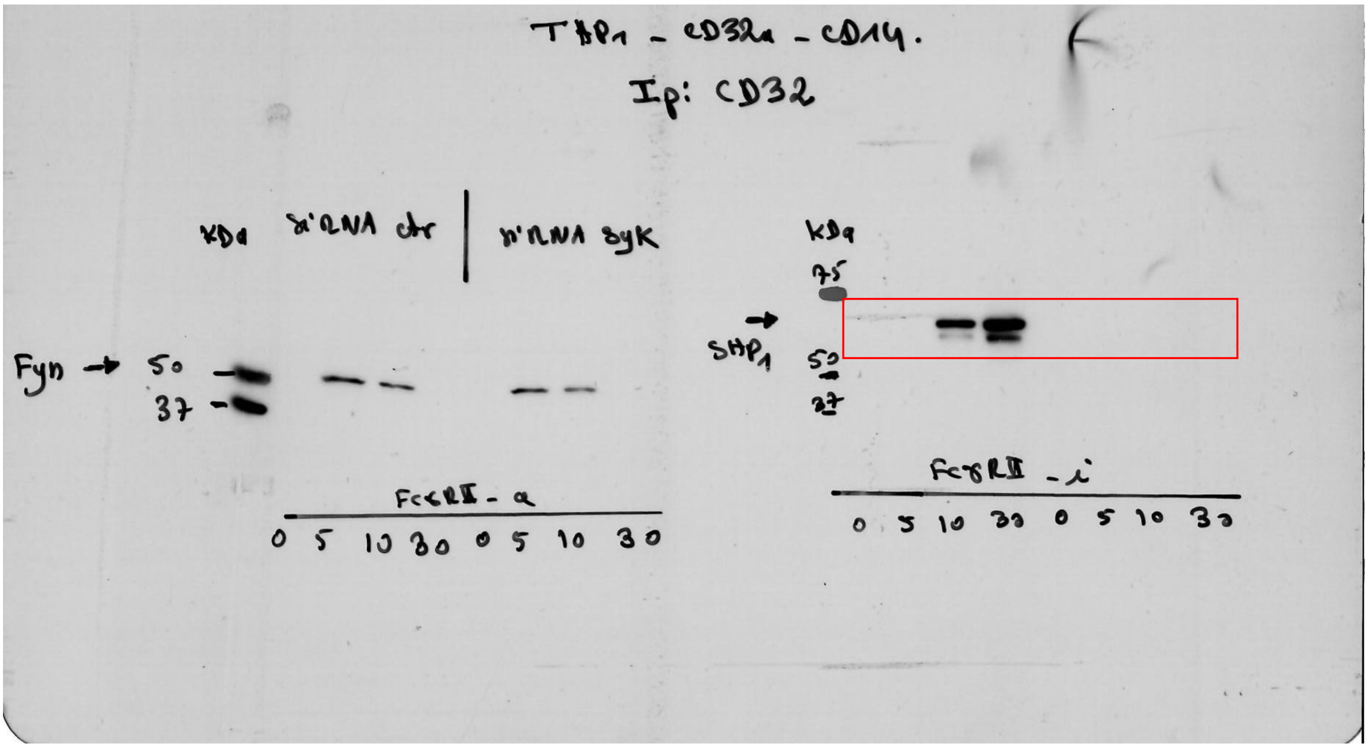

Supplementary Figure 3a

IP: FcγRIIA, IB: Syk (FcγRIIA-ITAM)

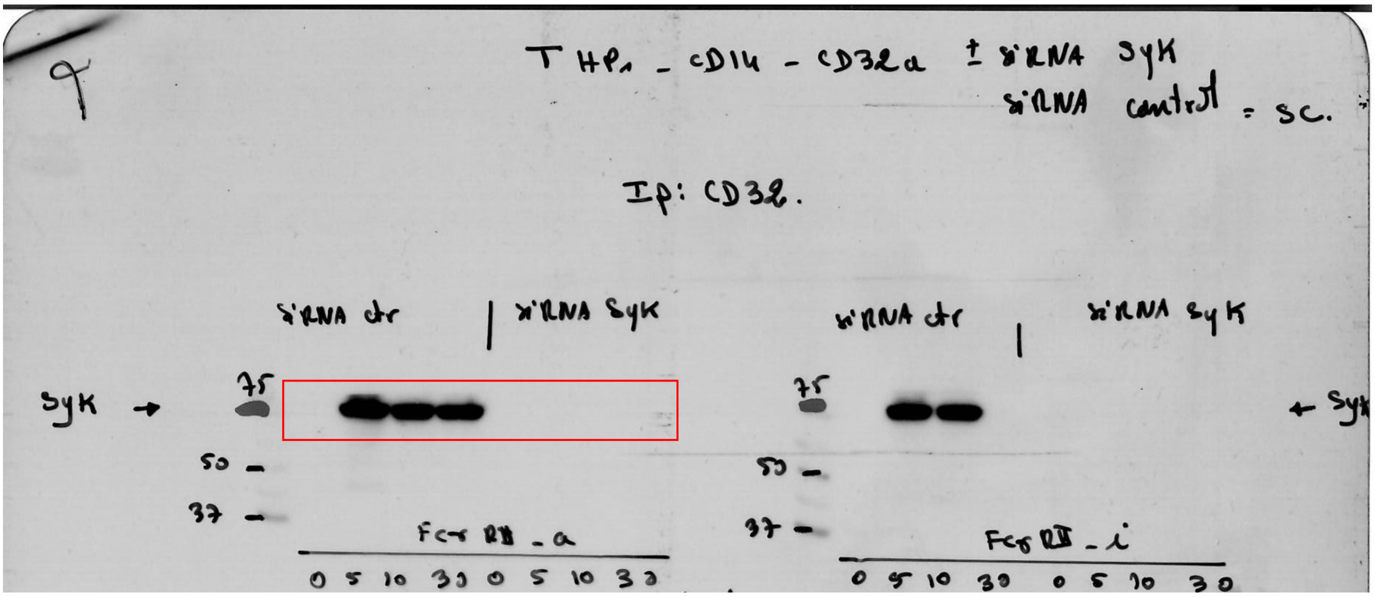

Supplementary Figure 3a

IP: FcγRIIA, IB: Syk (FcγRIIA-ITAMi)

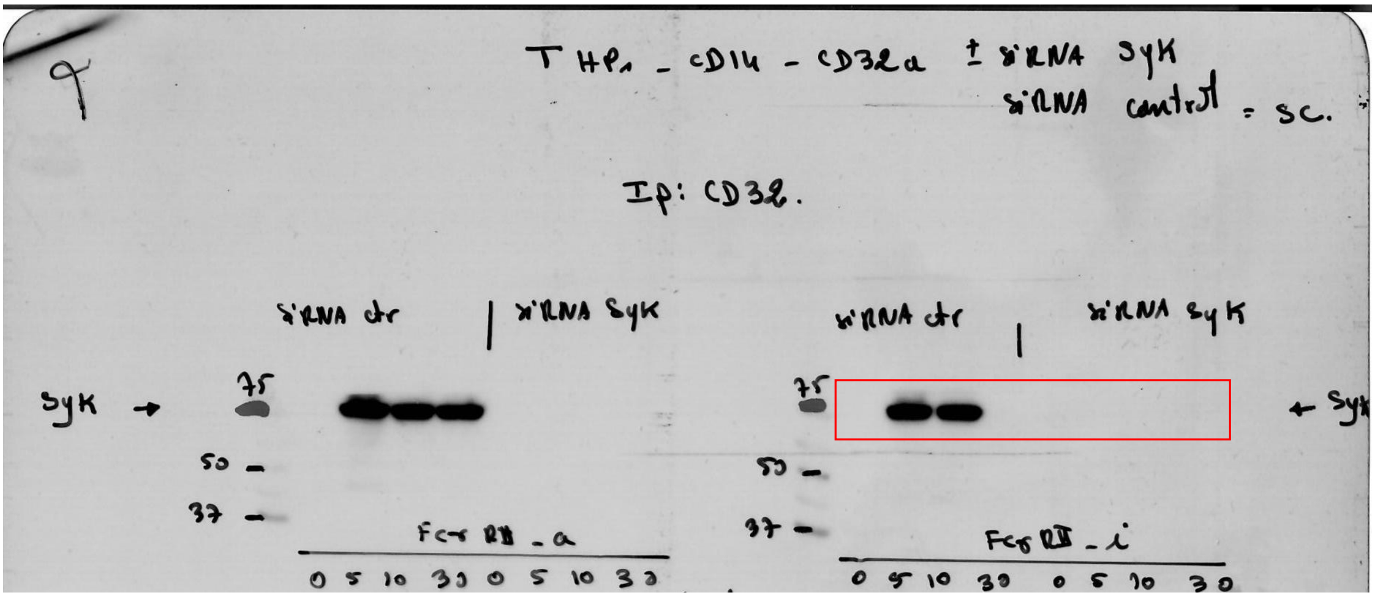

Supplementary Figure 3a

Lysate, IB: Syk (FcγRIIA-ITAM)

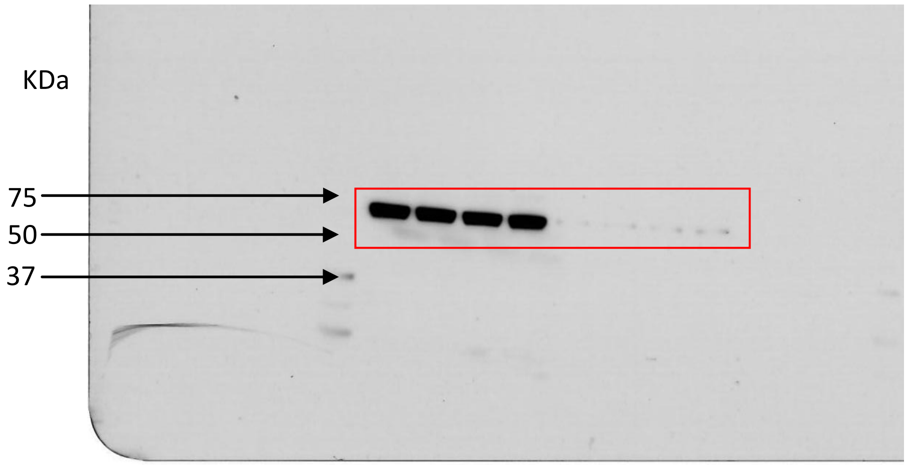

Supplementary Figure 3a

Lysate, IB: Syk (FcγRIIA-ITAM)

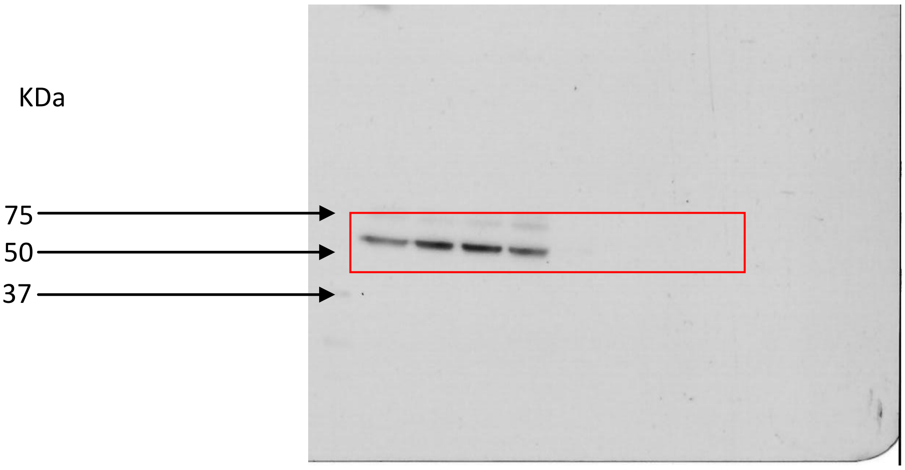

Supplementary Figure 3a

Lysate, IB: SHP-1 (FcγRIIA-ITAM)

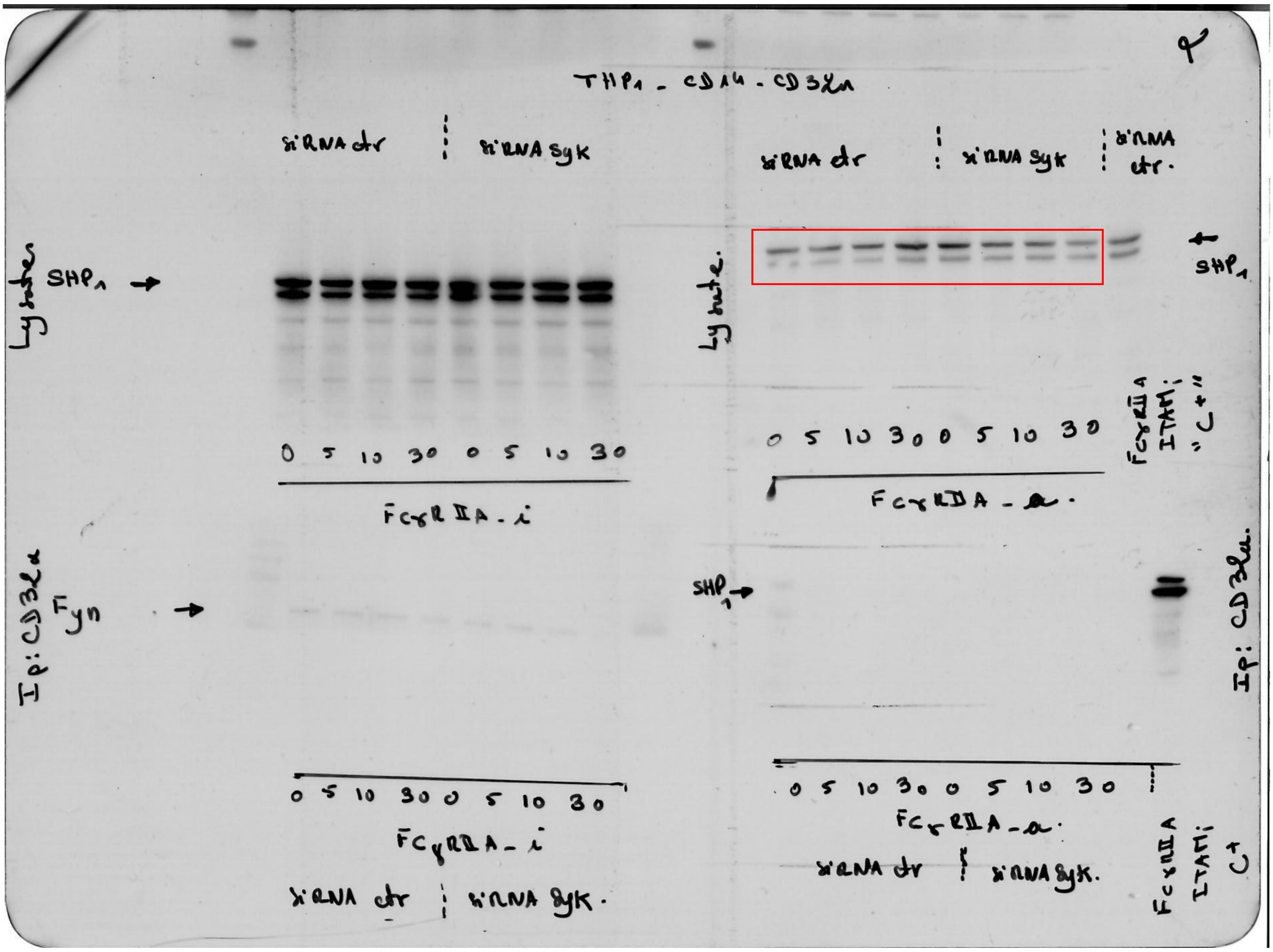

Supplementary Figure 3a

Lysate, IB: SHP-1 (FcγRIIA-ITAMi)

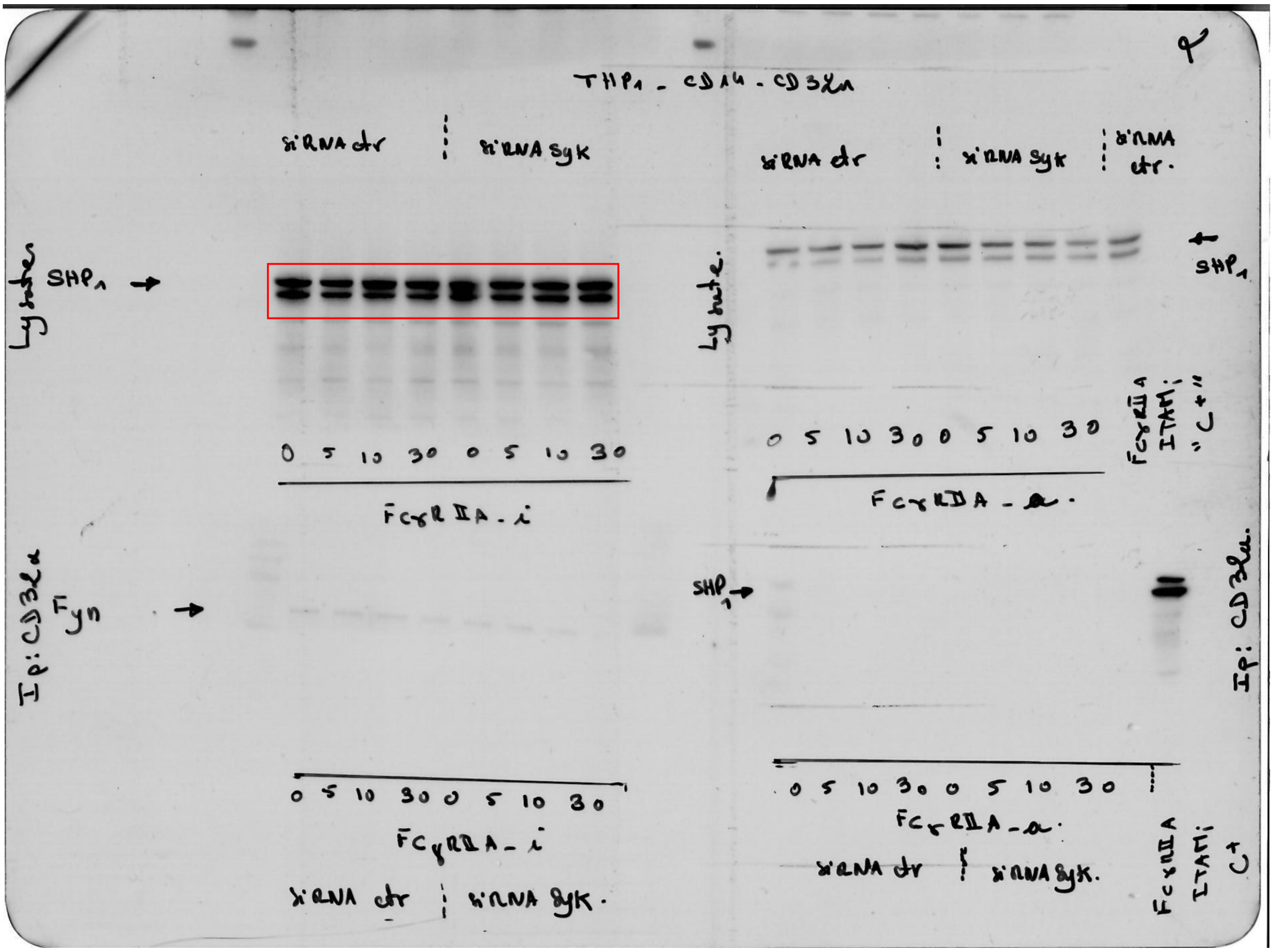

Supplementary Figure 3a

IP: FcγRIIA, IB: SHP-1 (FcγRIIA-ITAM)

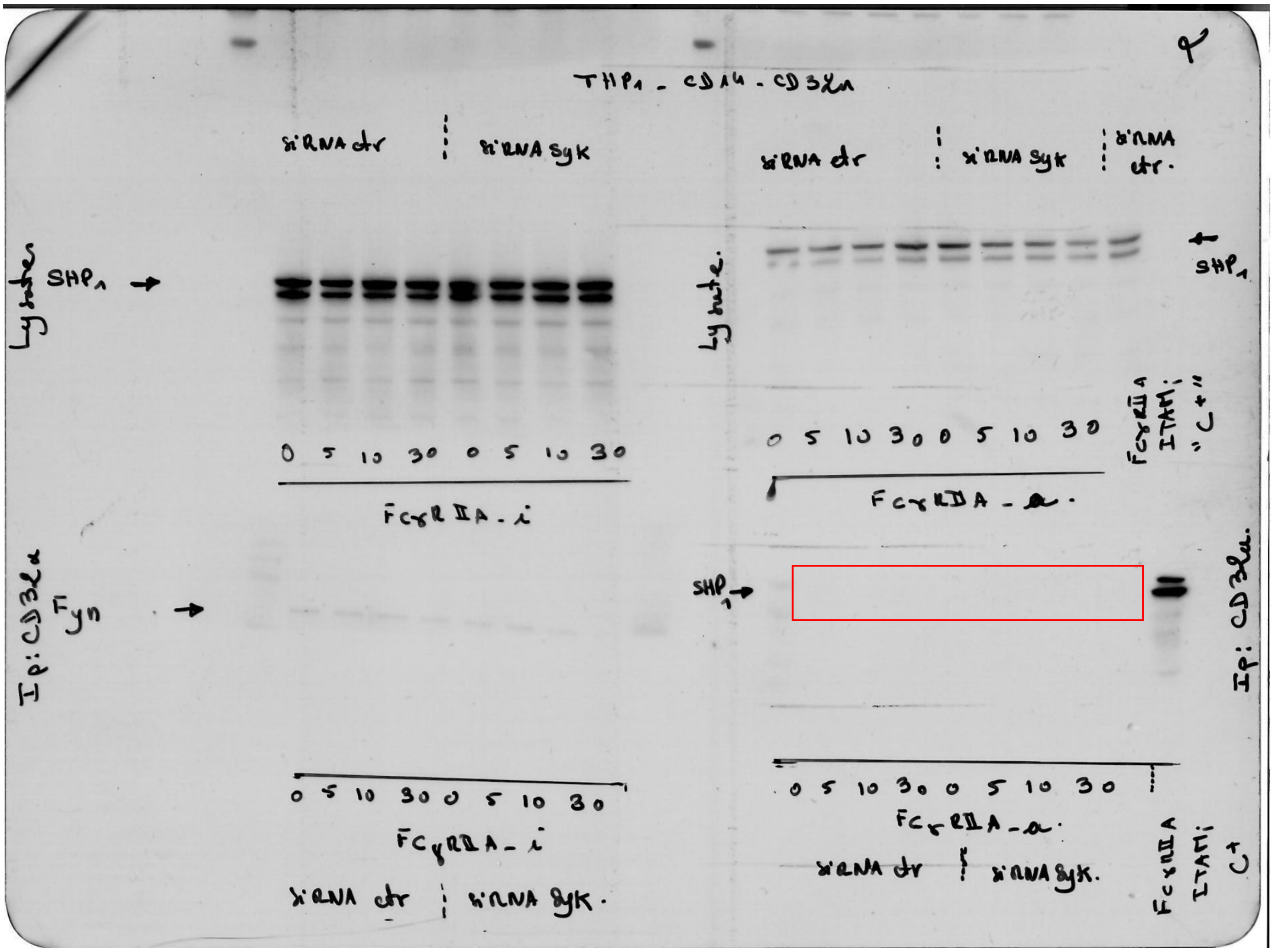

Supplementary Figure 3a

IP: FcγRIIA, IB: Fyn (FcγRIIA-ITAMi)

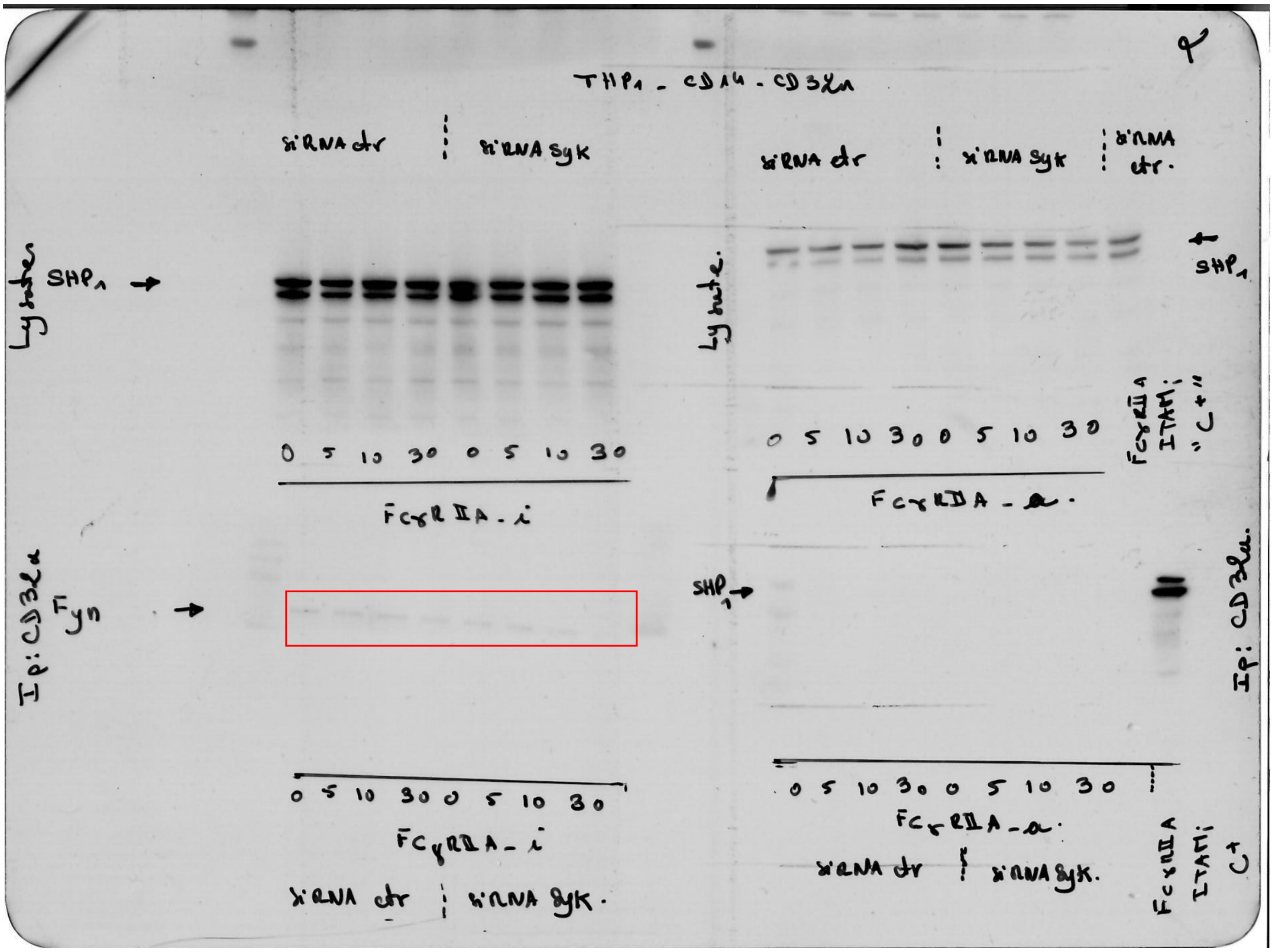

Supplementary Figure 3a

Lysate, IB: Fyn (FcγRIIA-ITAMi)

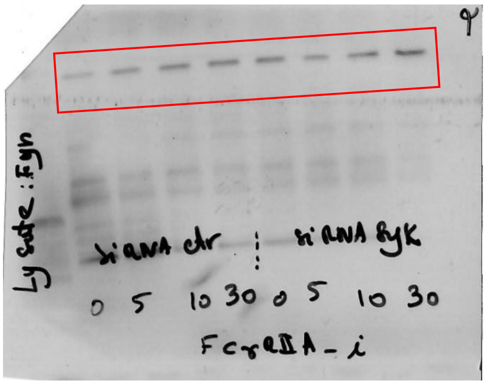

Supplementary Figure 3c

IB: pSyk$^{Y525/526}$ (PI3K inhibitor)

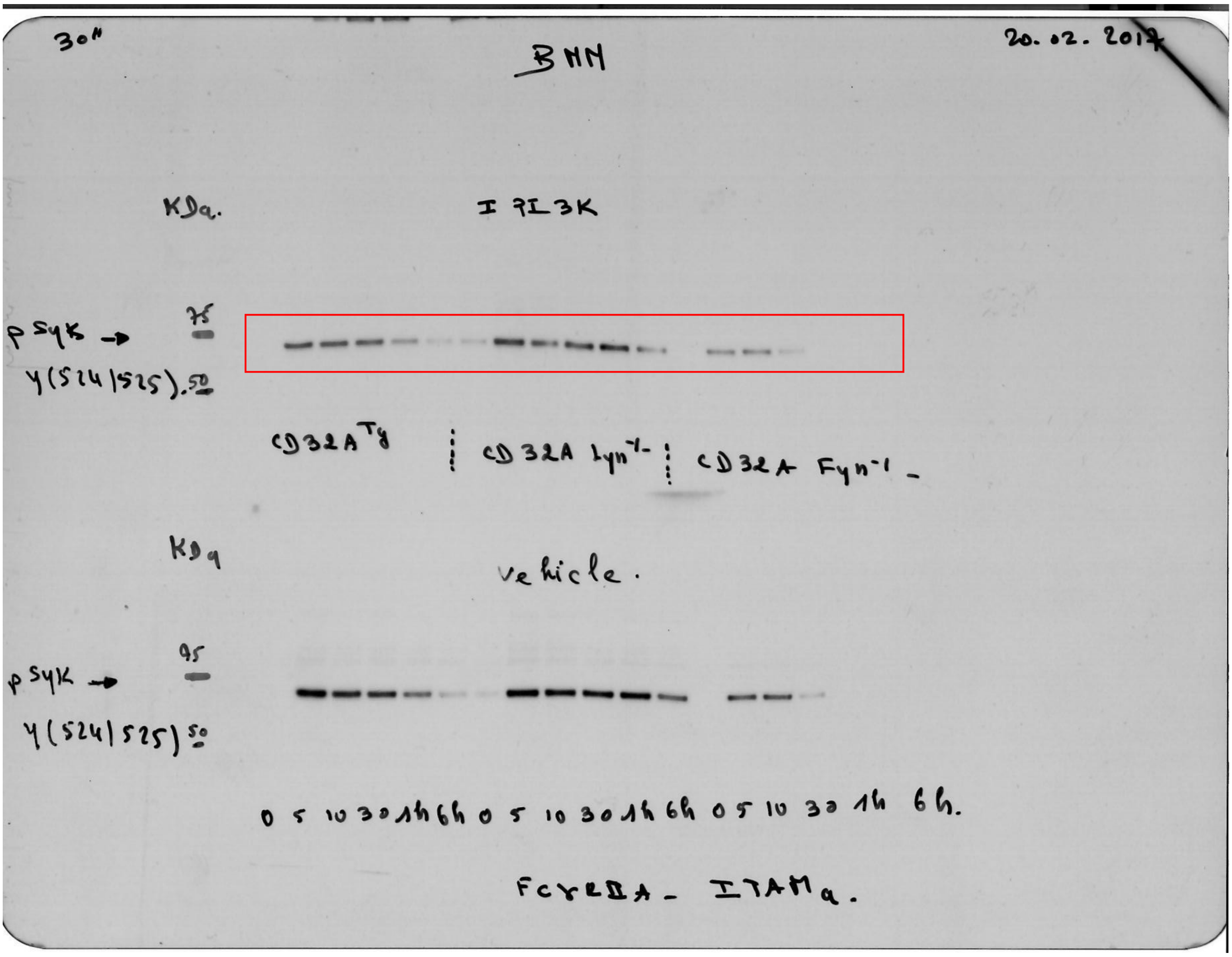

Supplementary Figure 3c

IB: pSyk$^{Y525/526}$ (Vehicle)

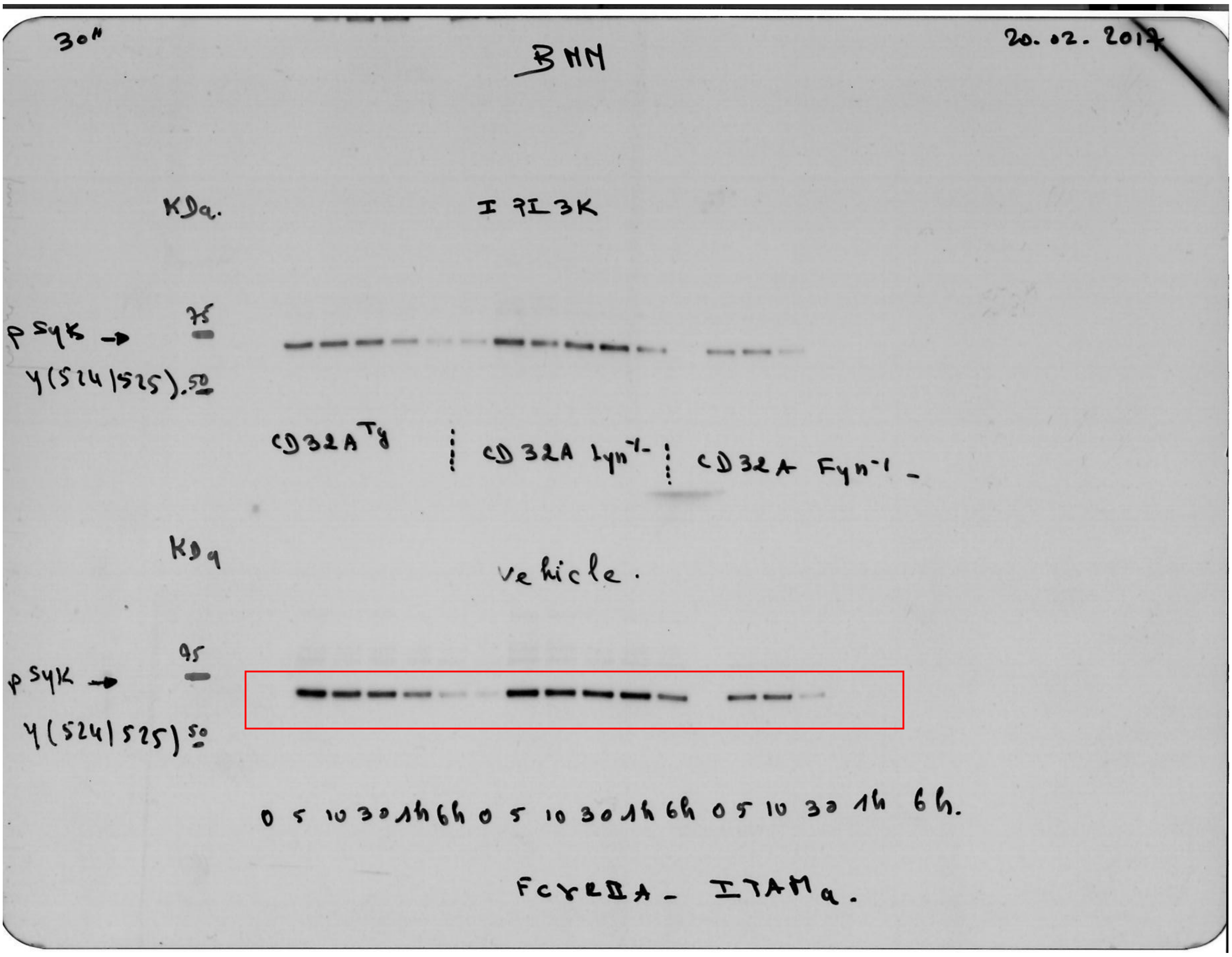

Supplementary Figure 3c

IB: Syk (Vehicle)

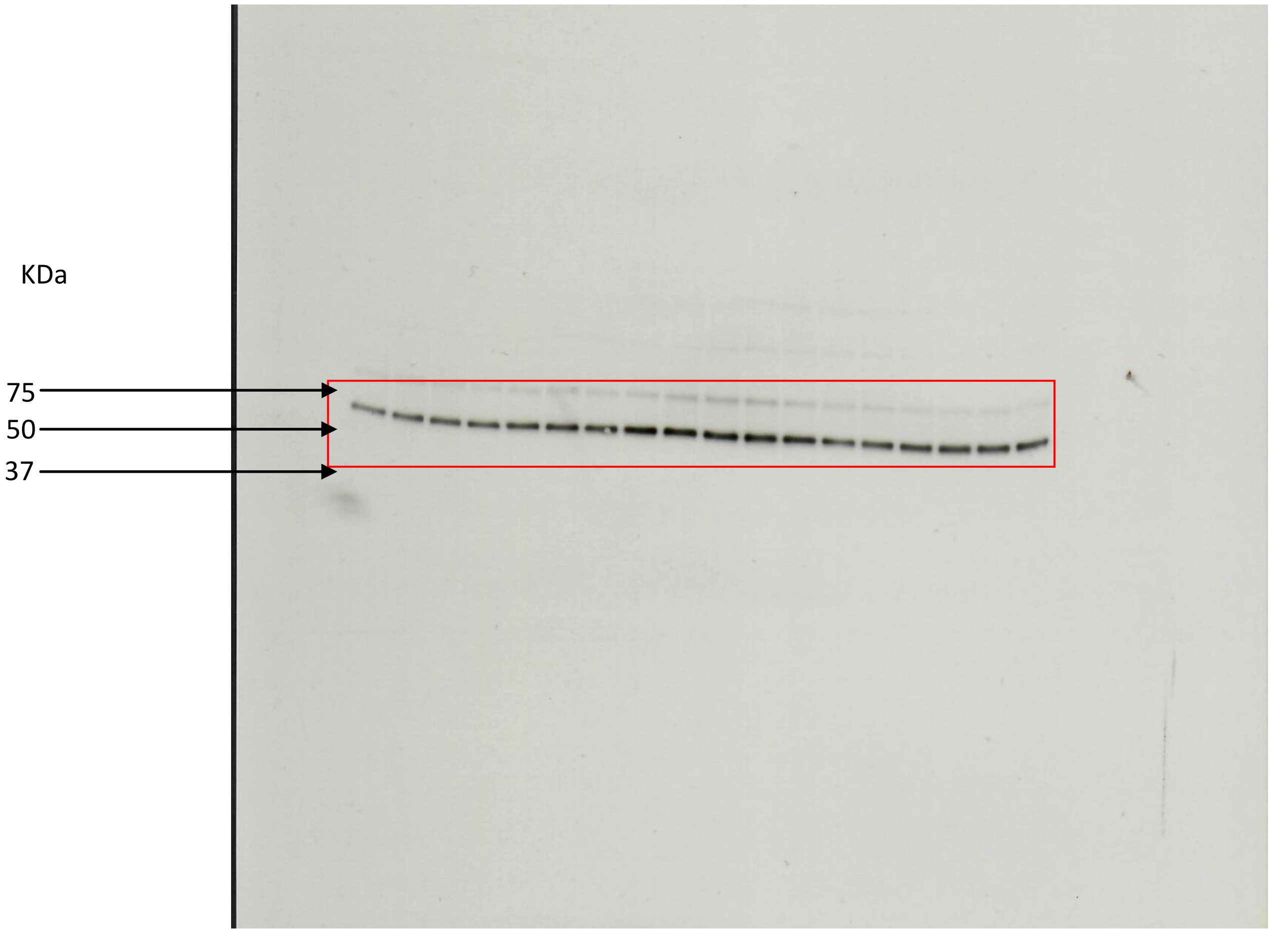

Supplementary Figure 3c

IB: pSyk$^{Y525/526}$ (control siRNA)

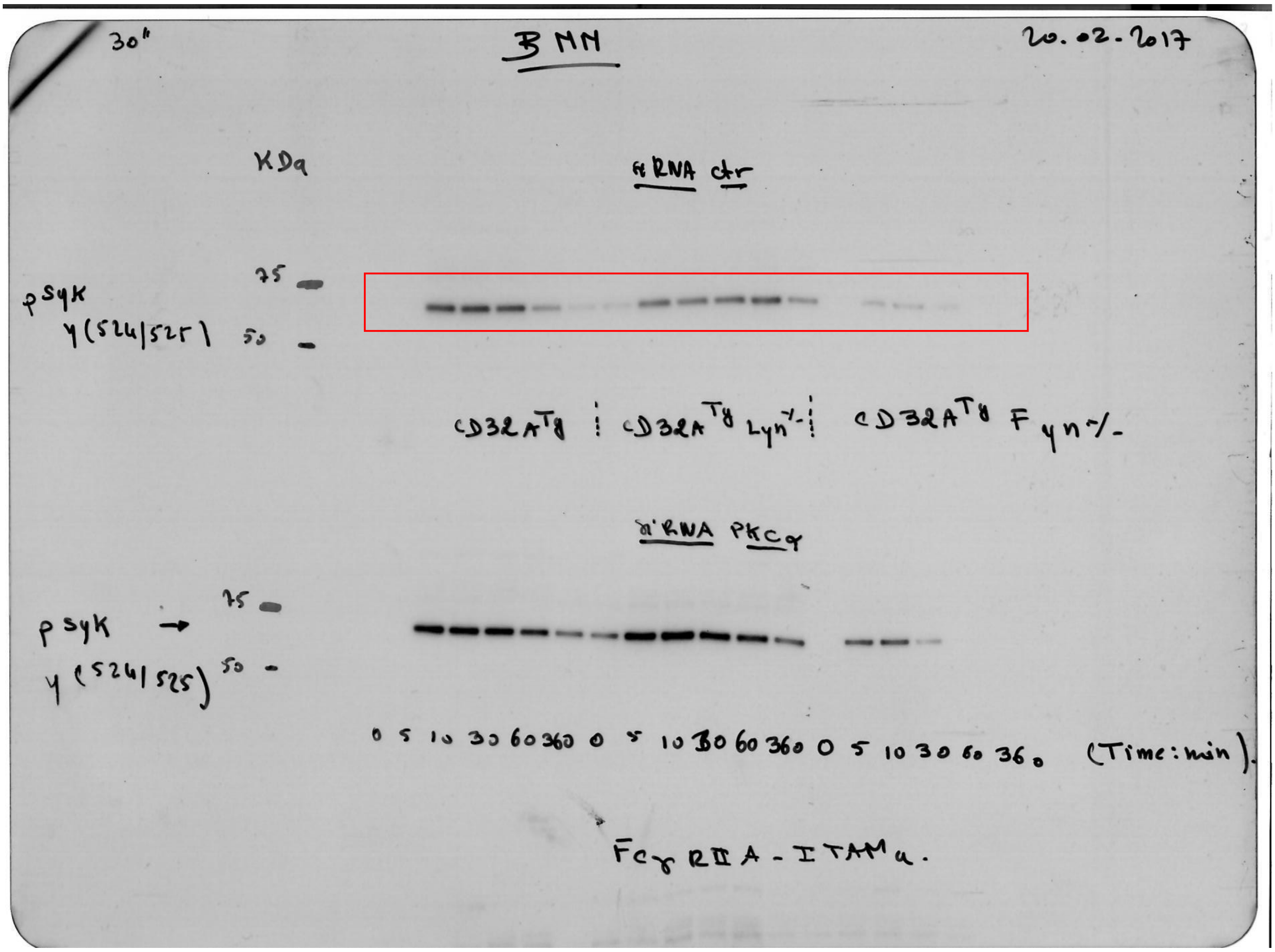

Supplementary Figure 3c

IB: pSyk$^{Y525/526}$ (PKCα siRNA)

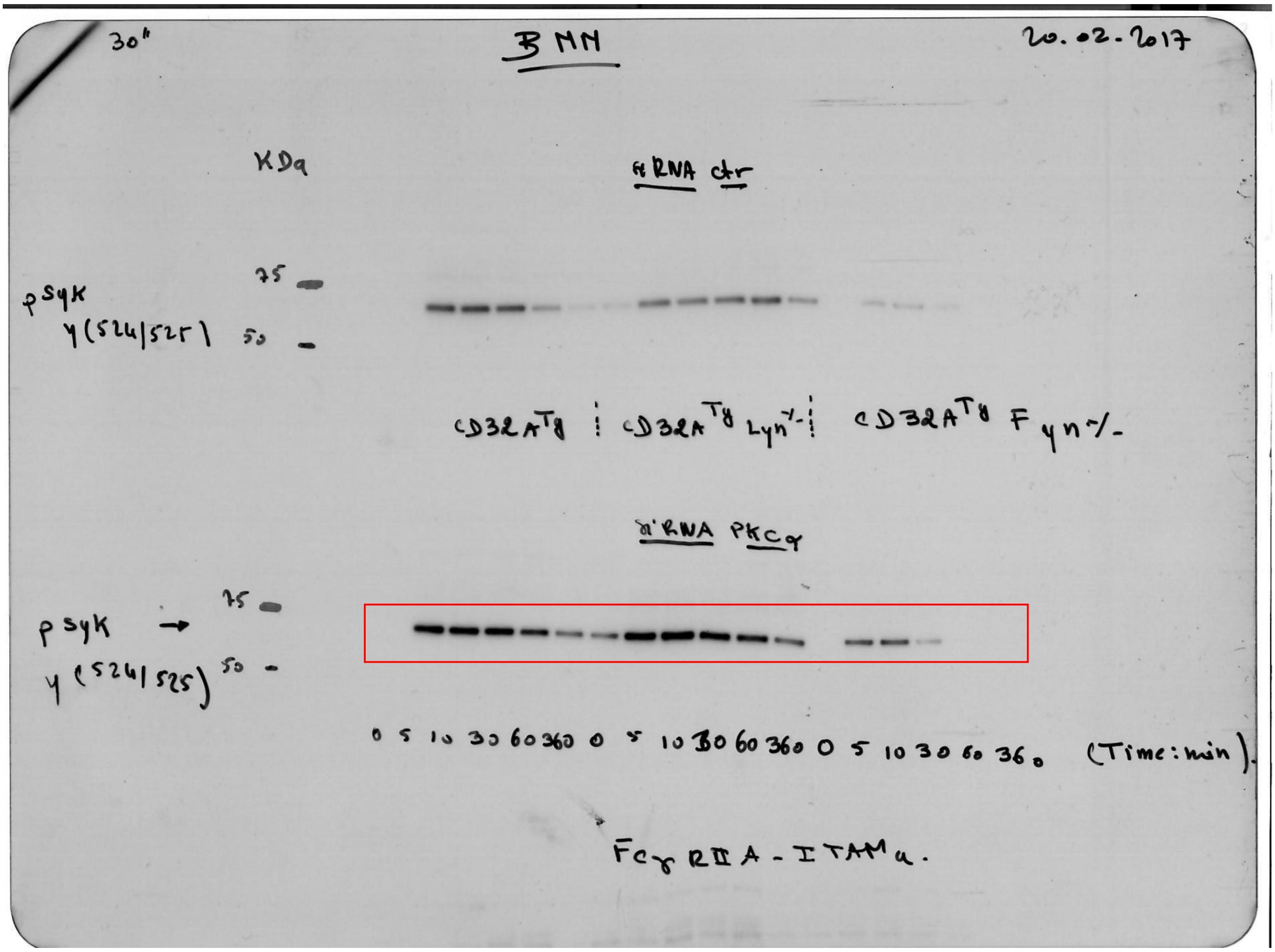

Supplementary Figure 3c

IB: Syk (PKCα siRNA)

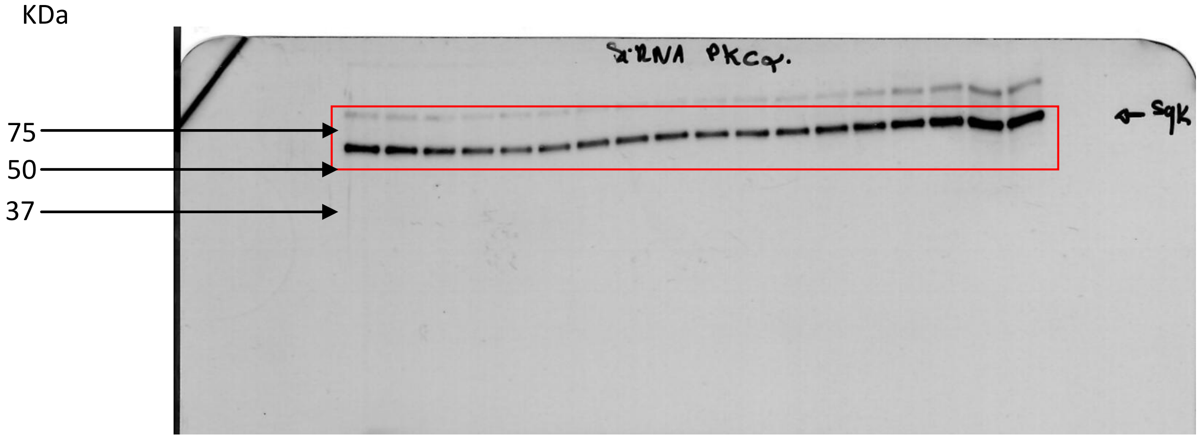

Supplementary Figure 3c

IB: PKCα (PKCα siRNA)

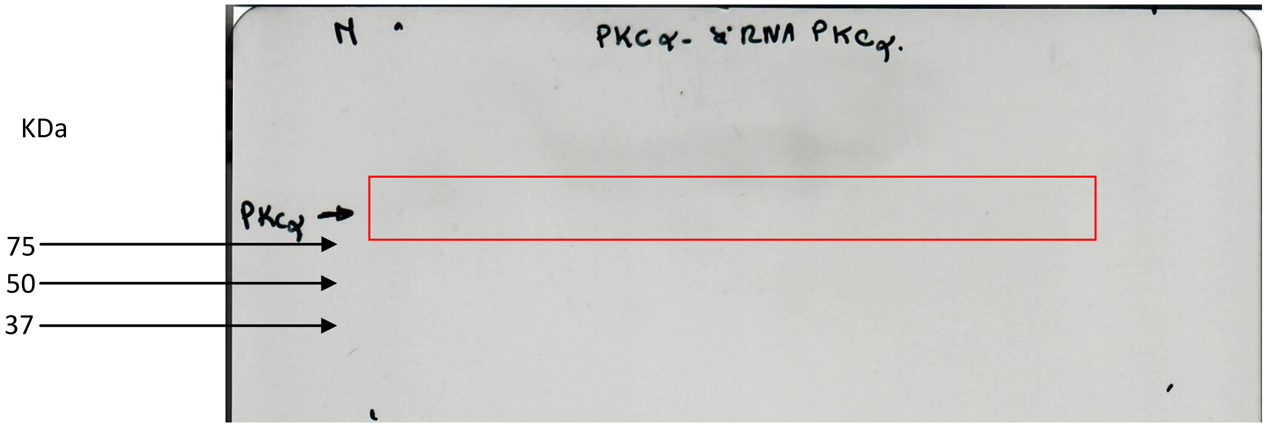

Supplementary Figure 3c

IB: PKC (Control siRNA)

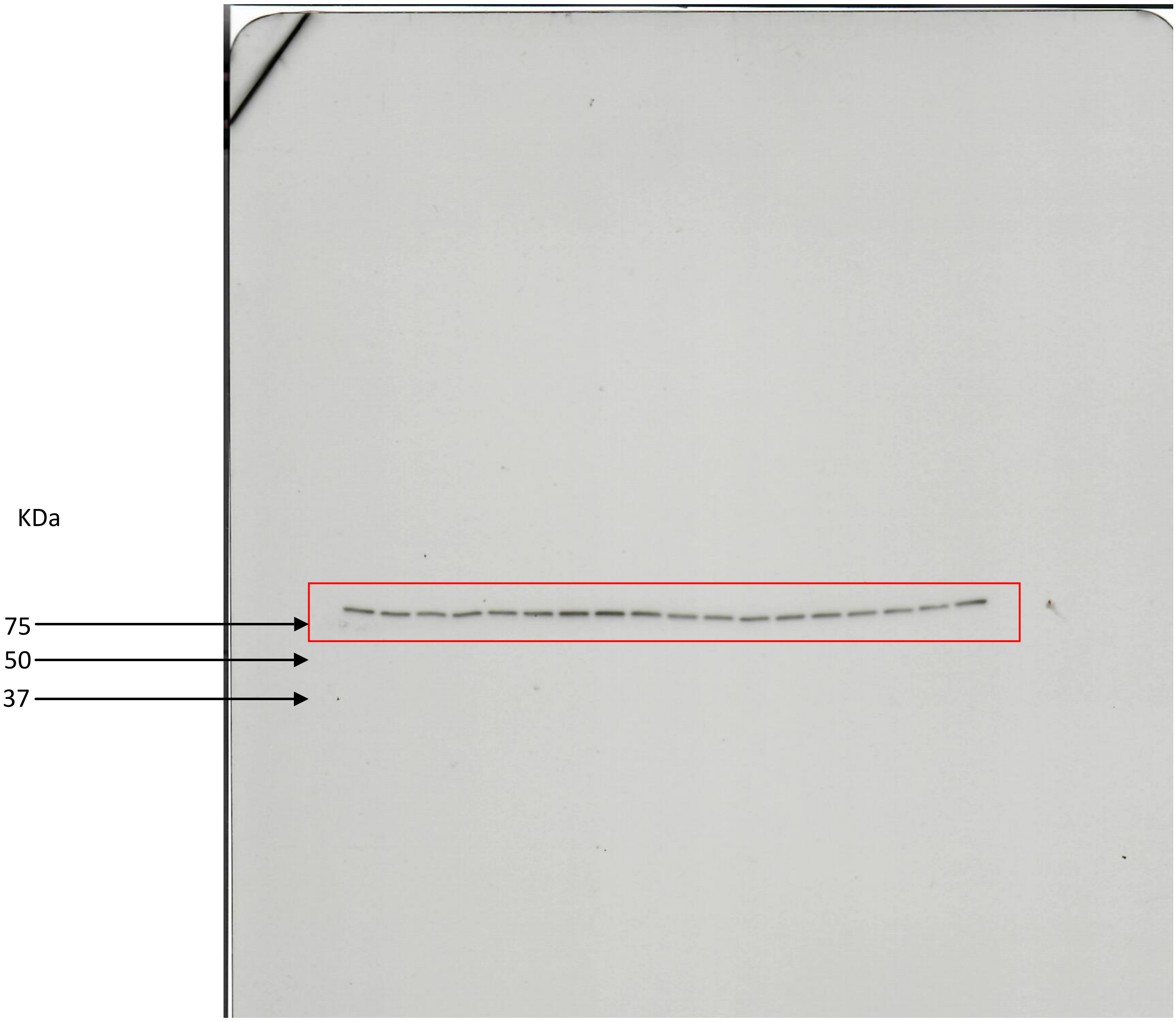

Supplementary Figure 3c

IB: Syk (Control siRNA)

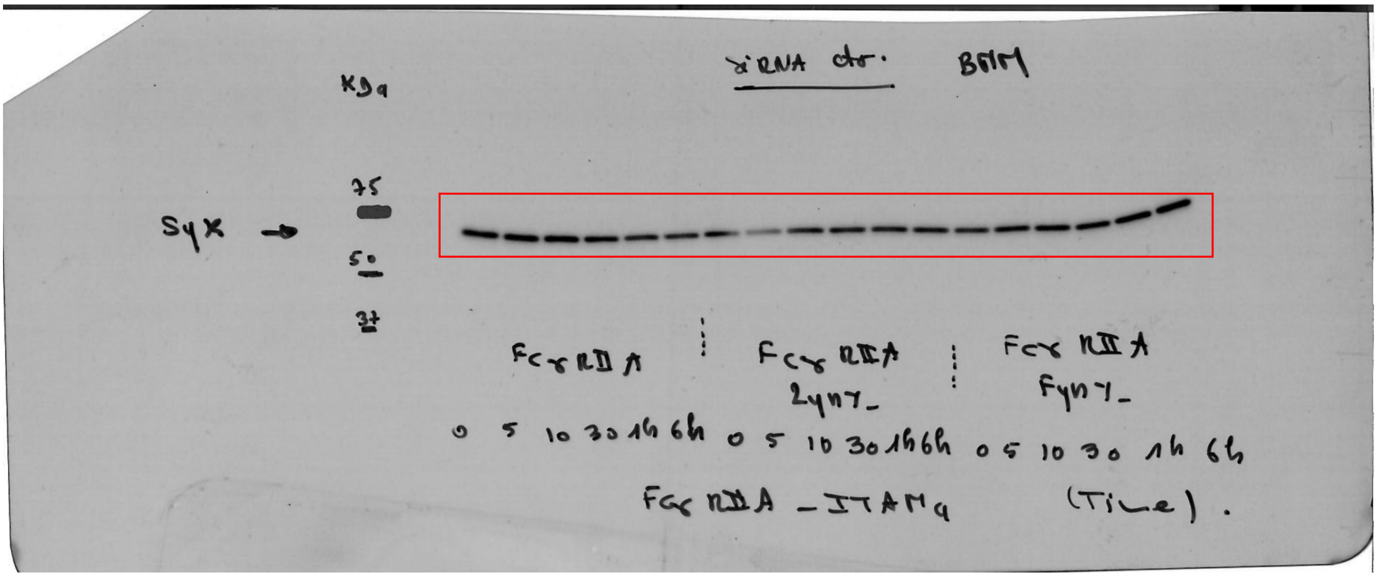

Supplementary Figure 3c

IB: Syk (PI3K inhibitor)

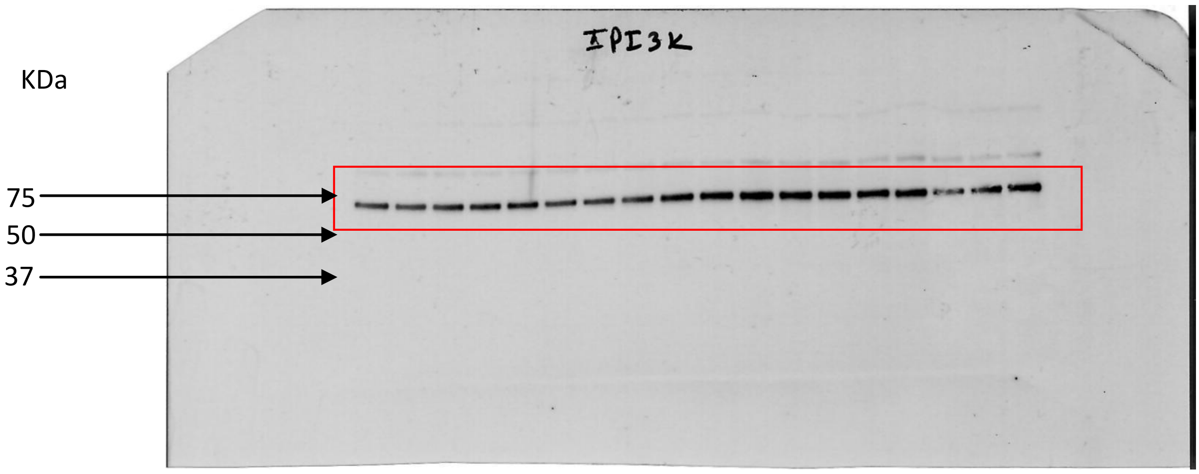

Supplementary Figure 4g

IB: actin

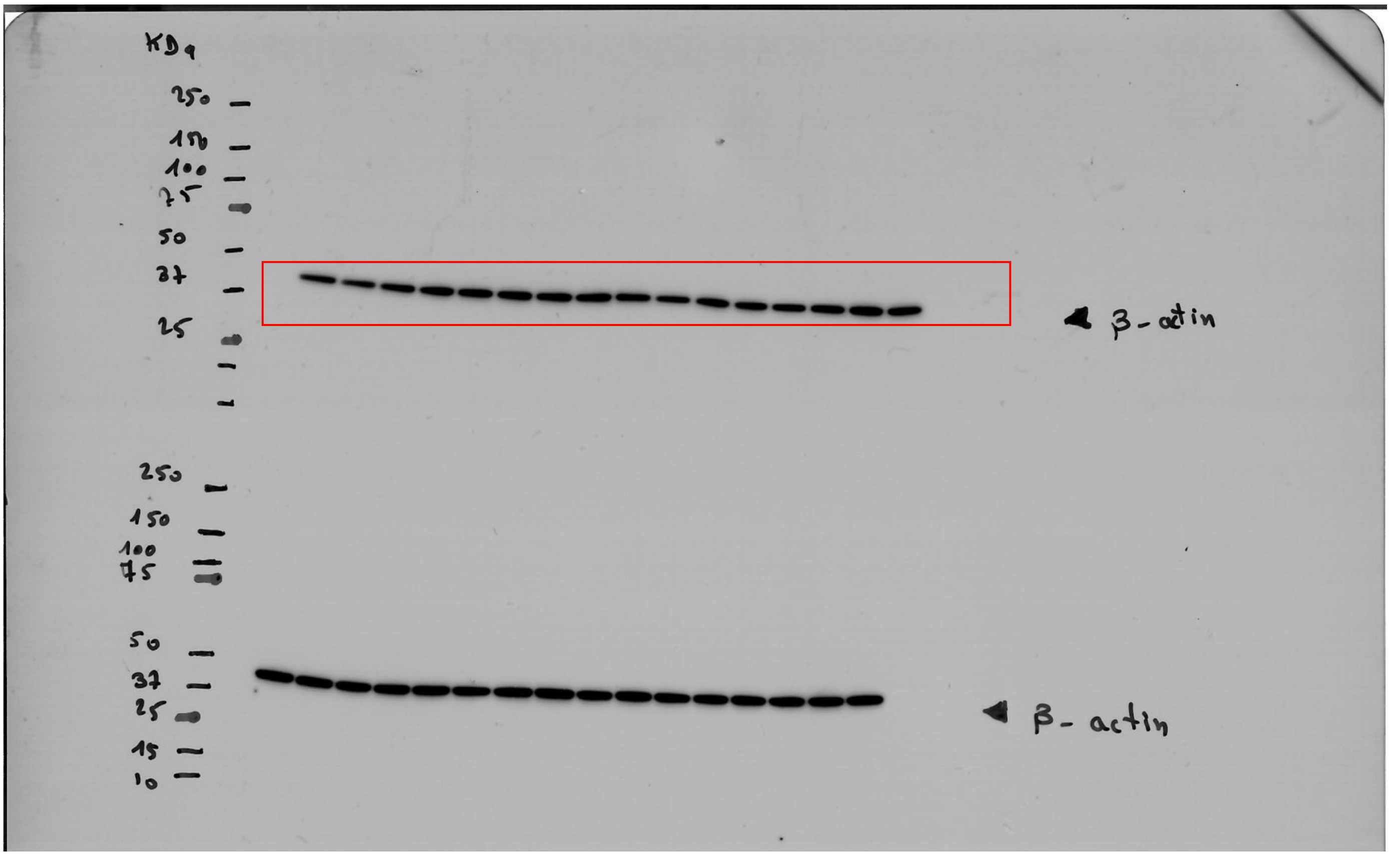

Supplementary Figure 4f

IB: Fyn, right panel

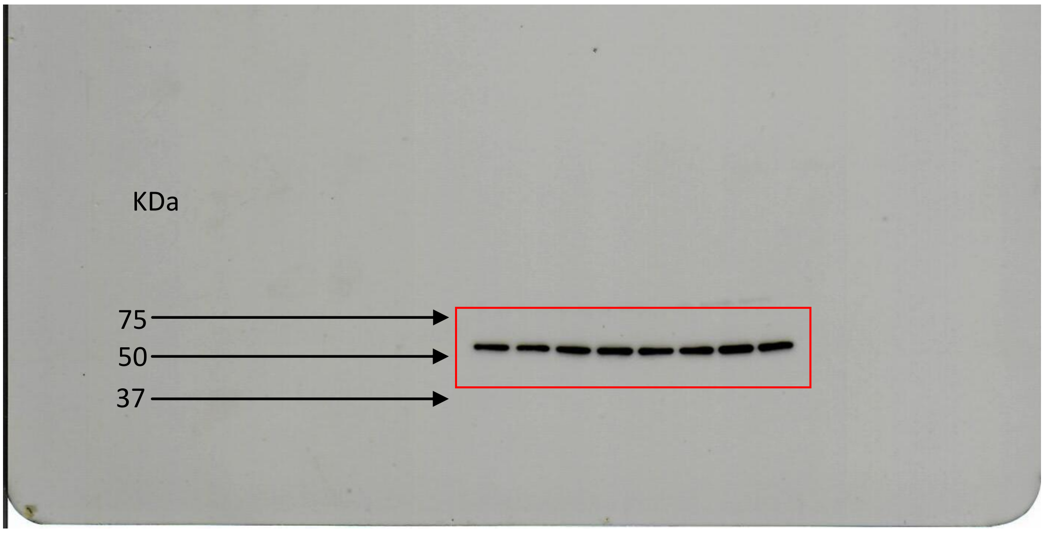

Supplementary Figure 4f

IB: Lyn, right panel

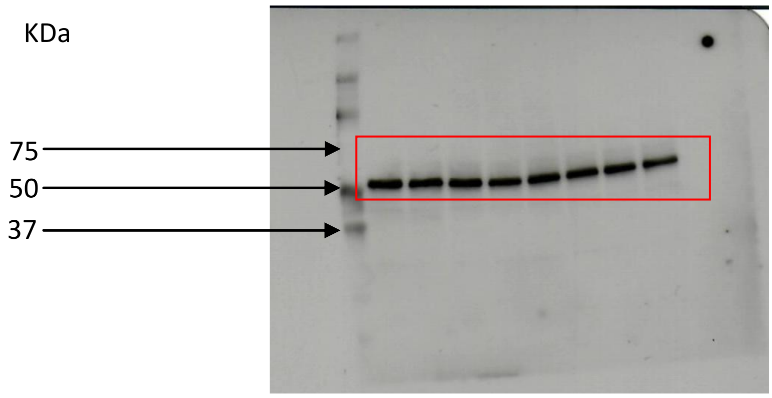

Supplementary Figure 4c

IB: Lyn, bottom panel

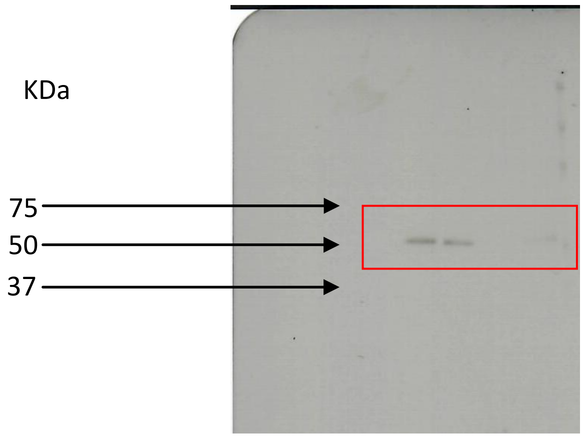

Supplementary Figure 4c

IB: Hck, bottom panel

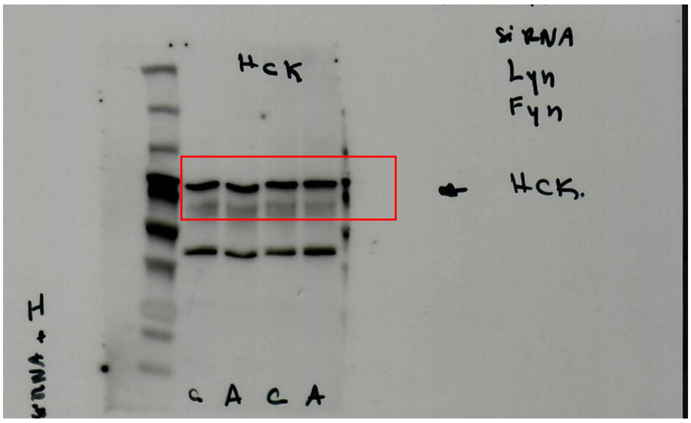

Supplementary Figure 4c

IB: Fgr, bottom panel

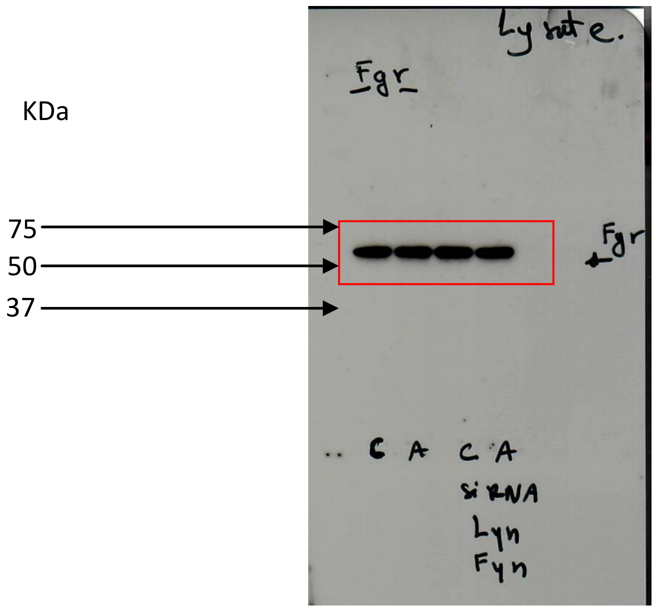

Supplementary Figure 4f

IB: Fyn, bottom panel

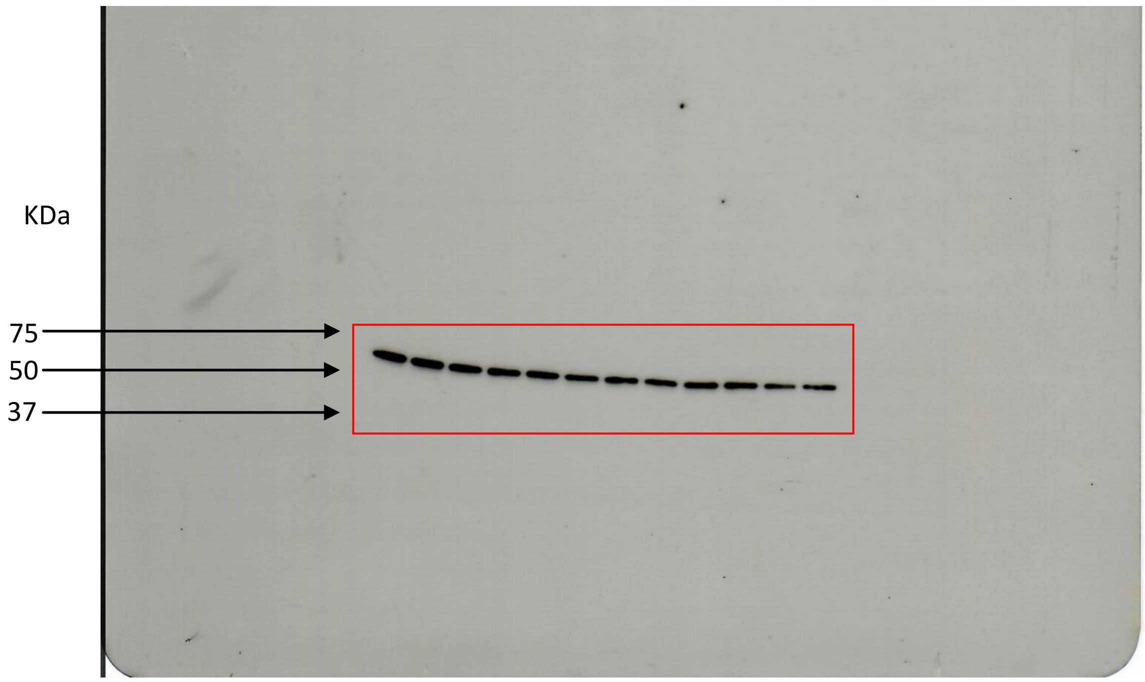

Supplementary Figure 4c

IB: Fyn, Top panel

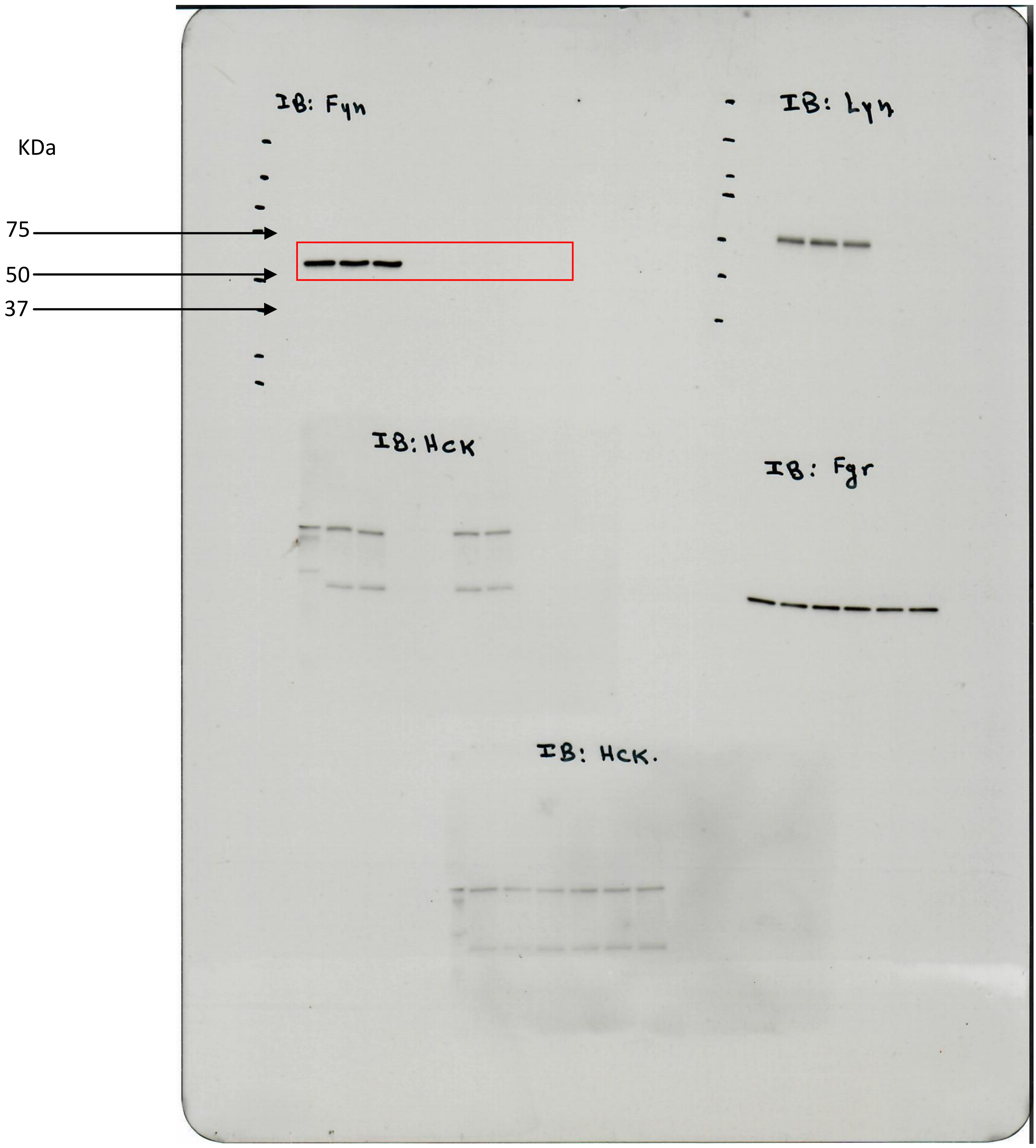

Supplementary Figure 4c

IB: Lyn, Top panel

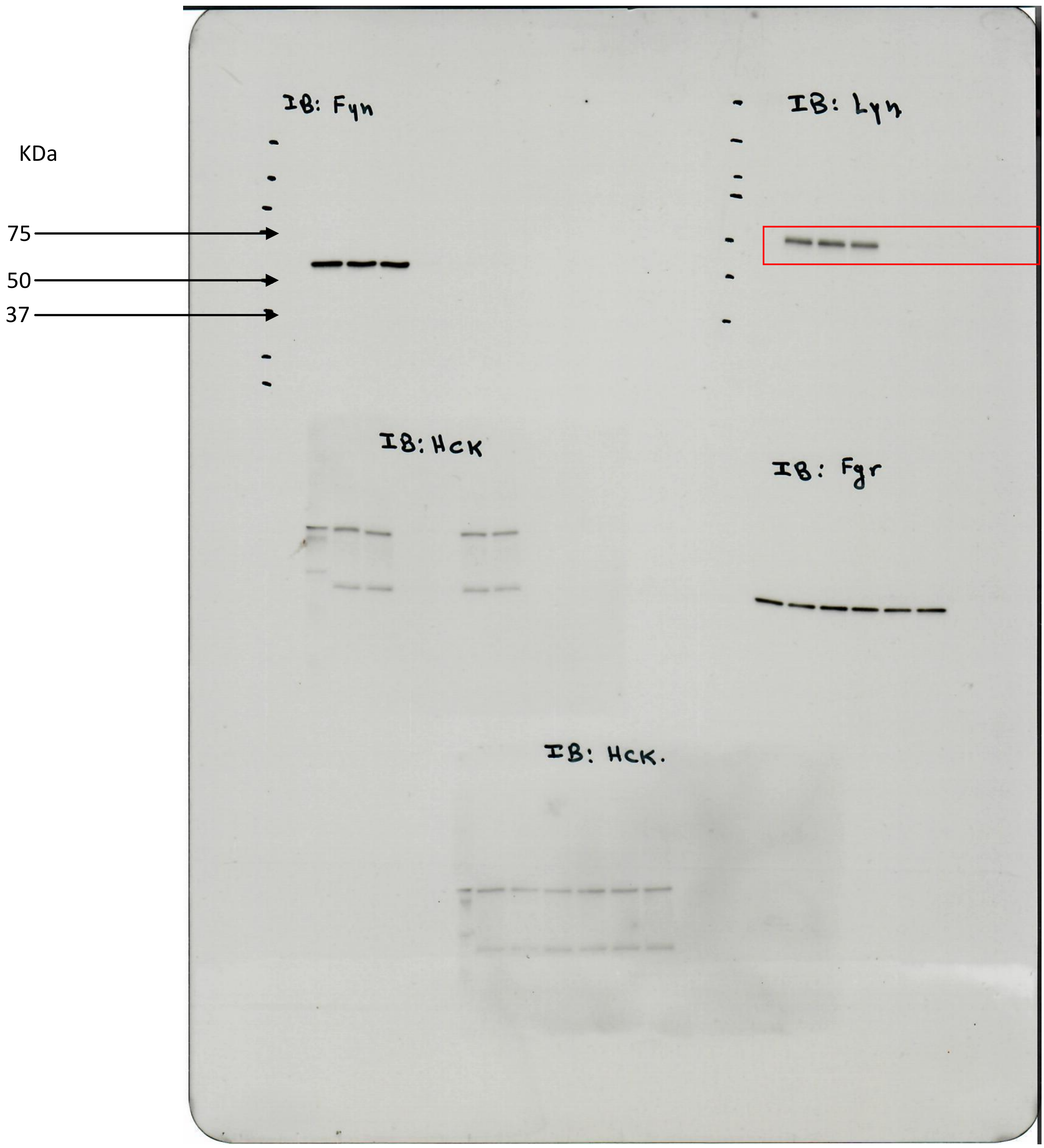

Supplementary Figure 4c

IB: Fgr, Top panel

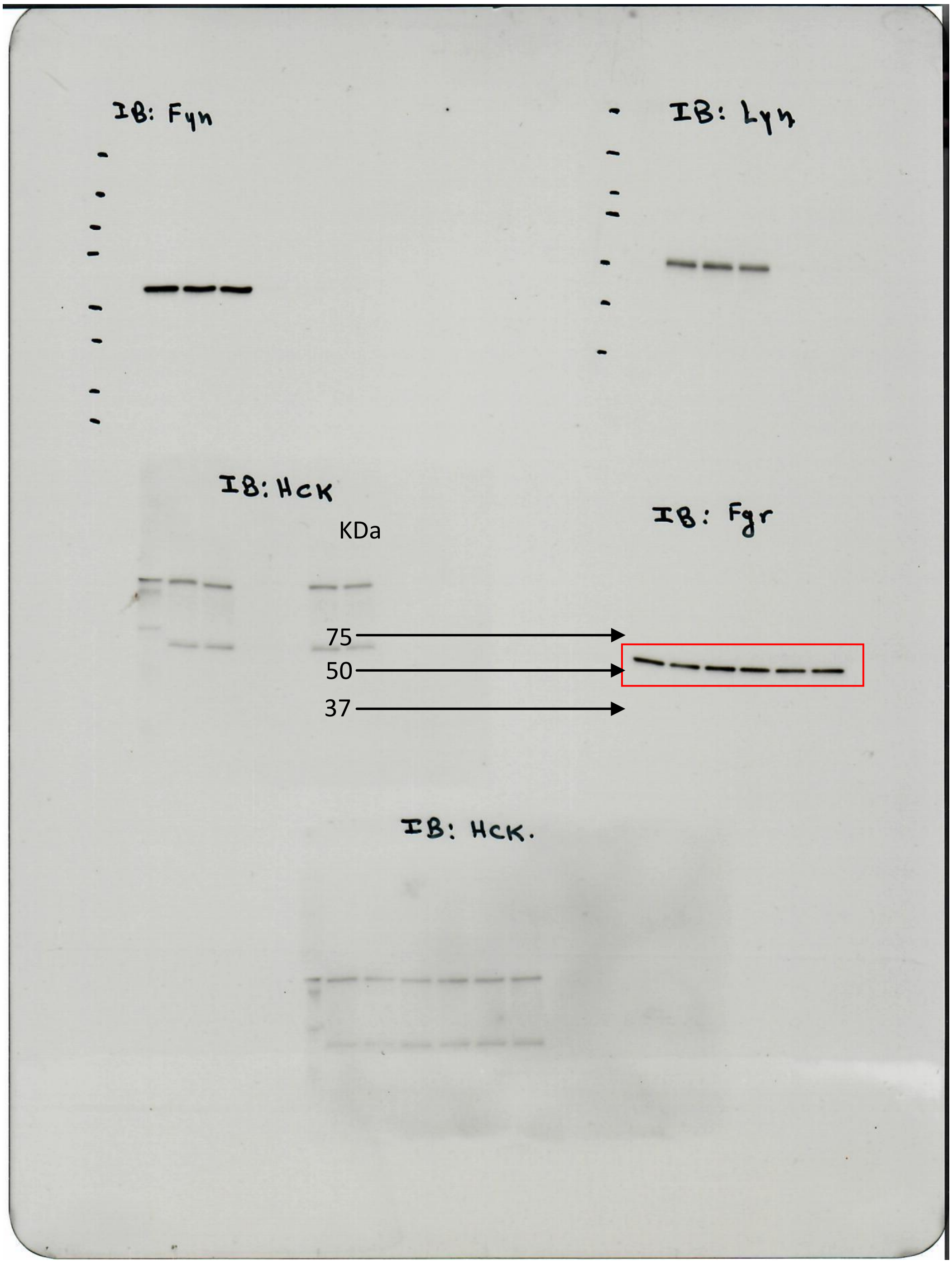

Supplementary Figure 4c

IB: Hck, Top panel

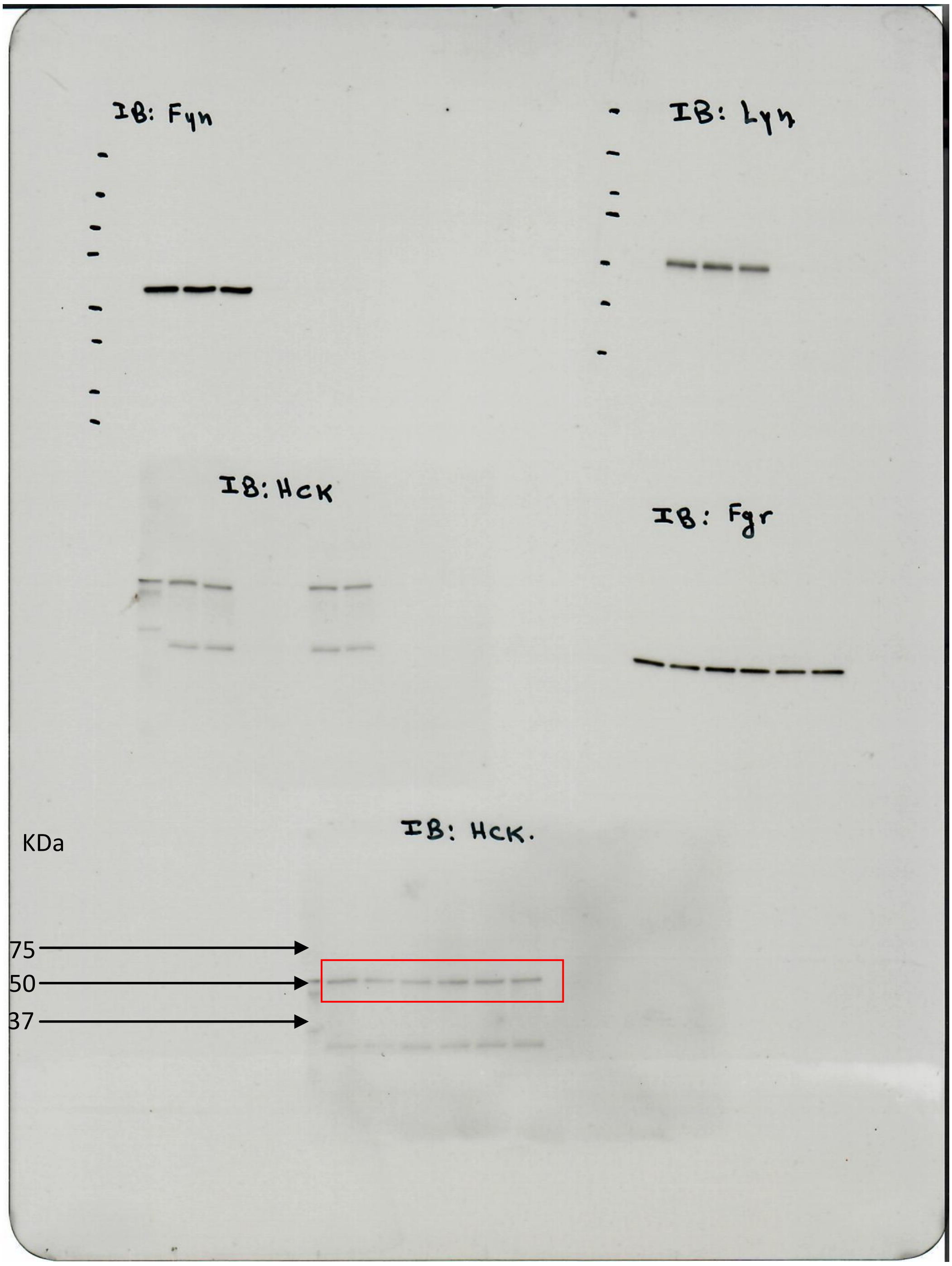

Supplementary Figure 4c

IB: Fyn, Bottom panel

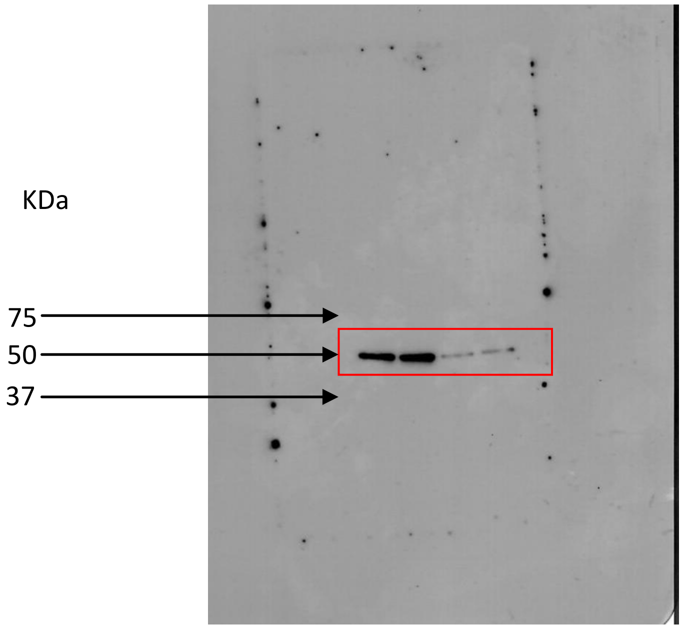

Supplementary Figure 4f

IB: Hck, right panel

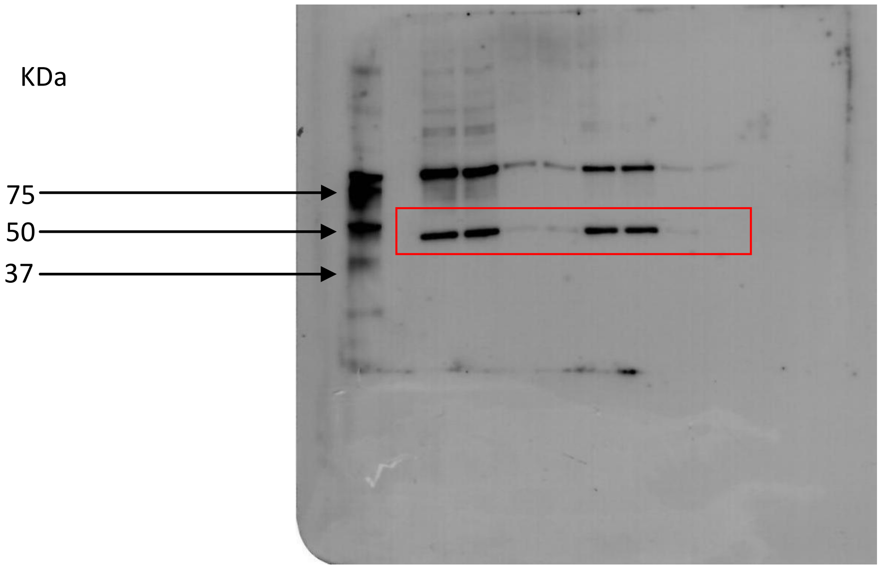

Supplementary Figure 4f

IB: Lyn, left panel

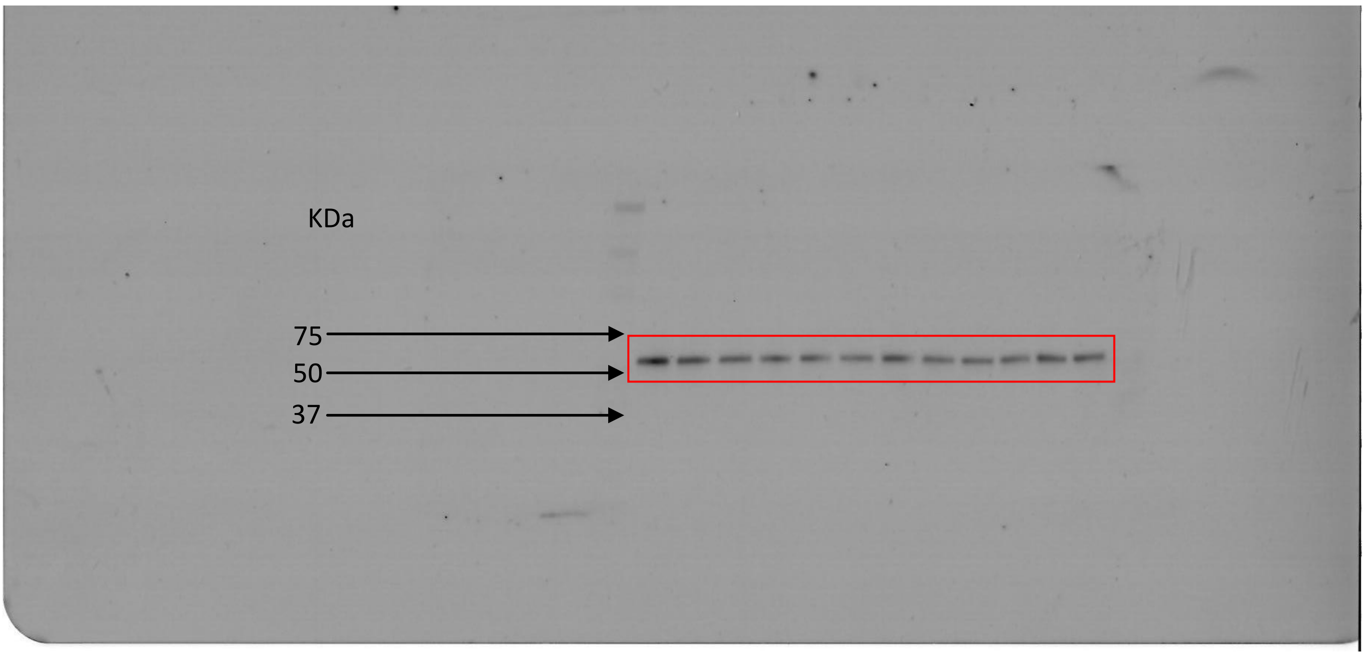

Supplementary Figure 4f

IB: Fgr, left panel

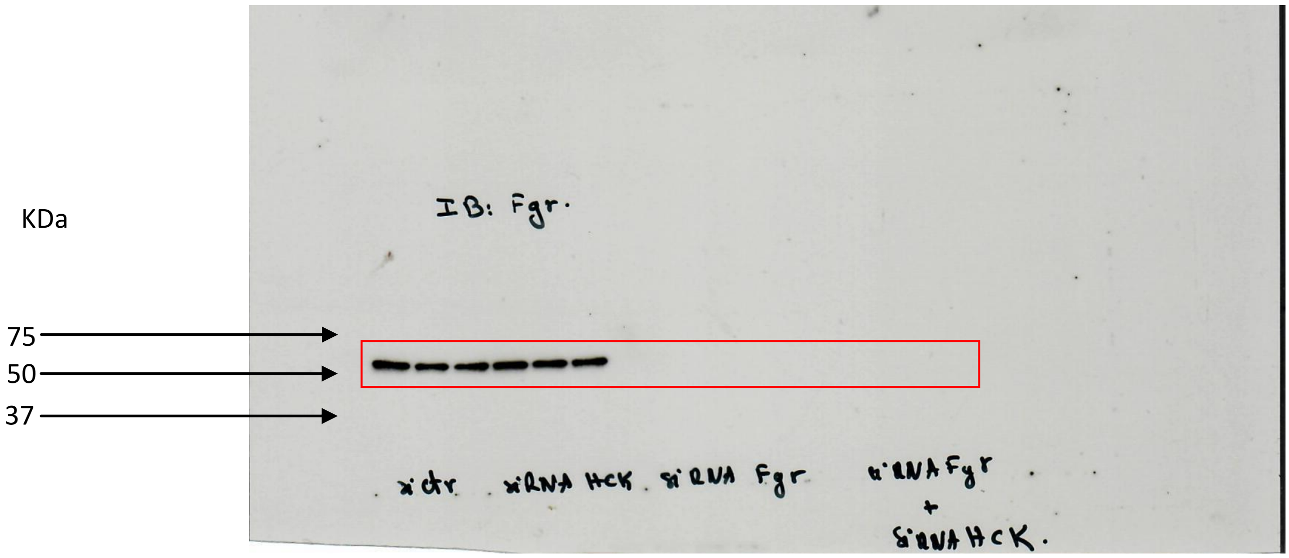

Supplementary Figure 4f

IB: Fyn, right panel

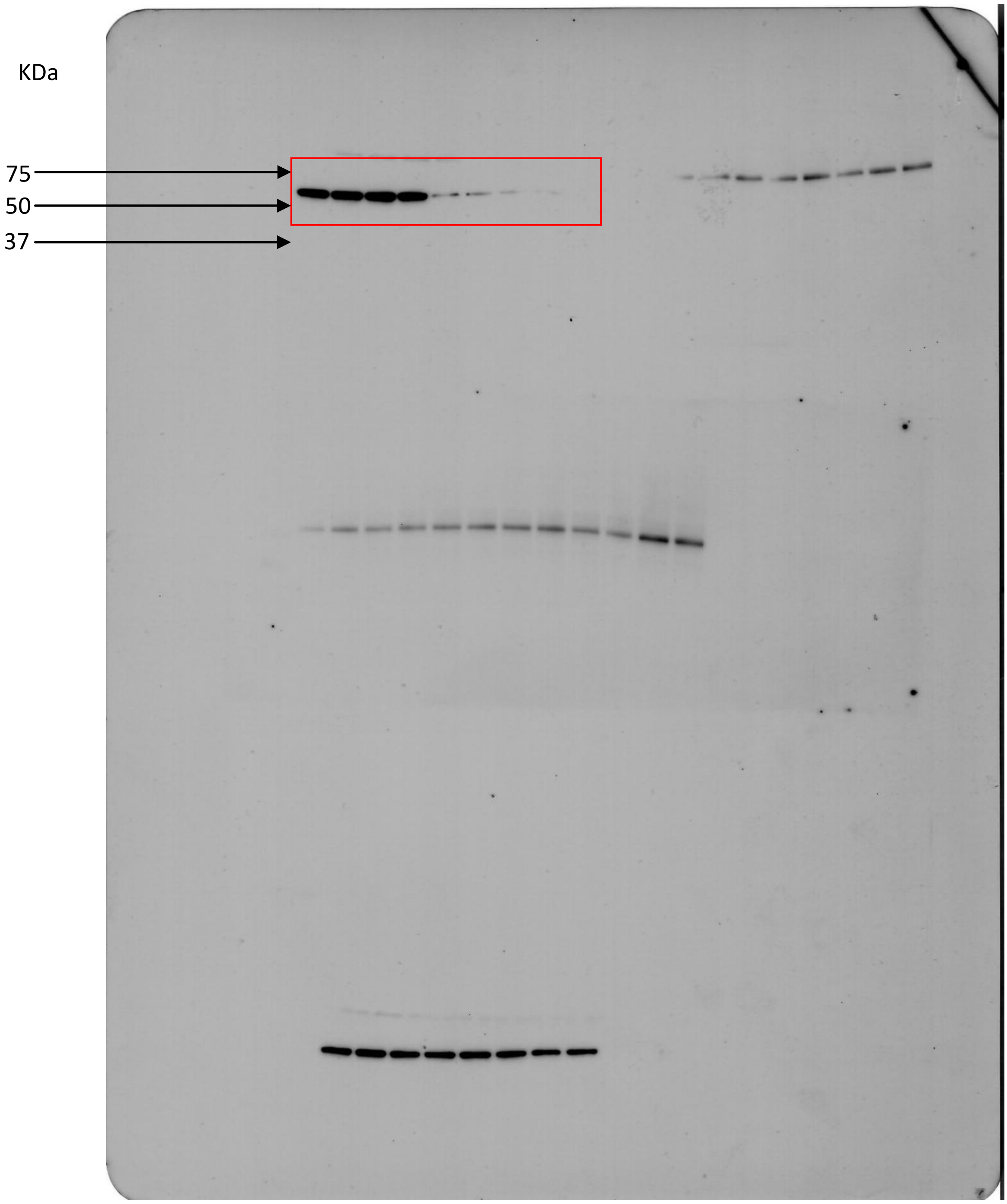

Supplementary Figure 4f

IB: Hck, left panel

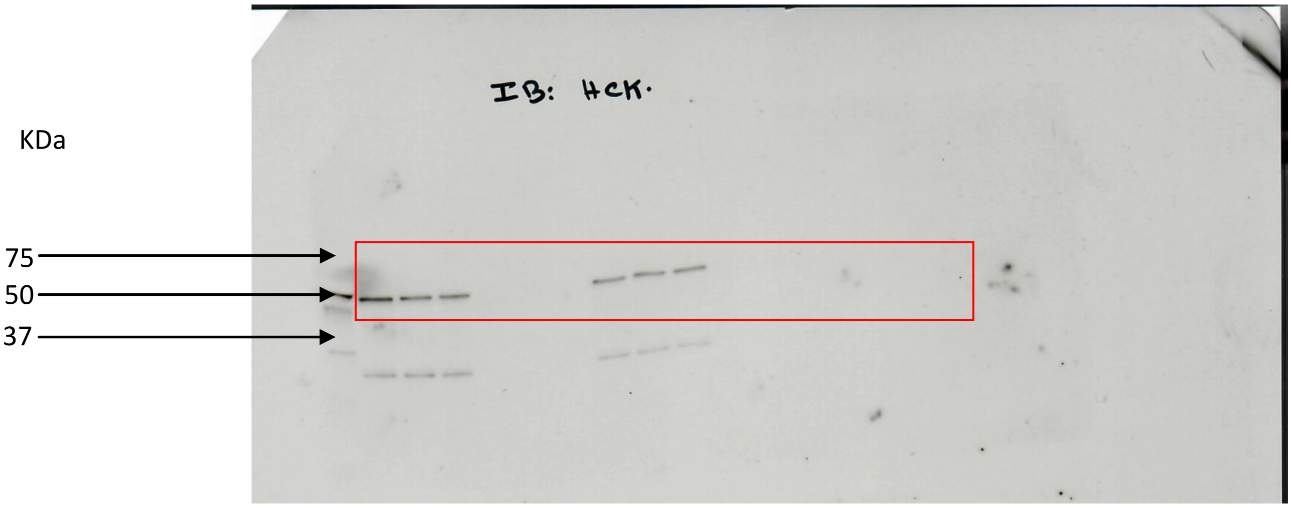

Supplementary Figure 4h

IB: actin

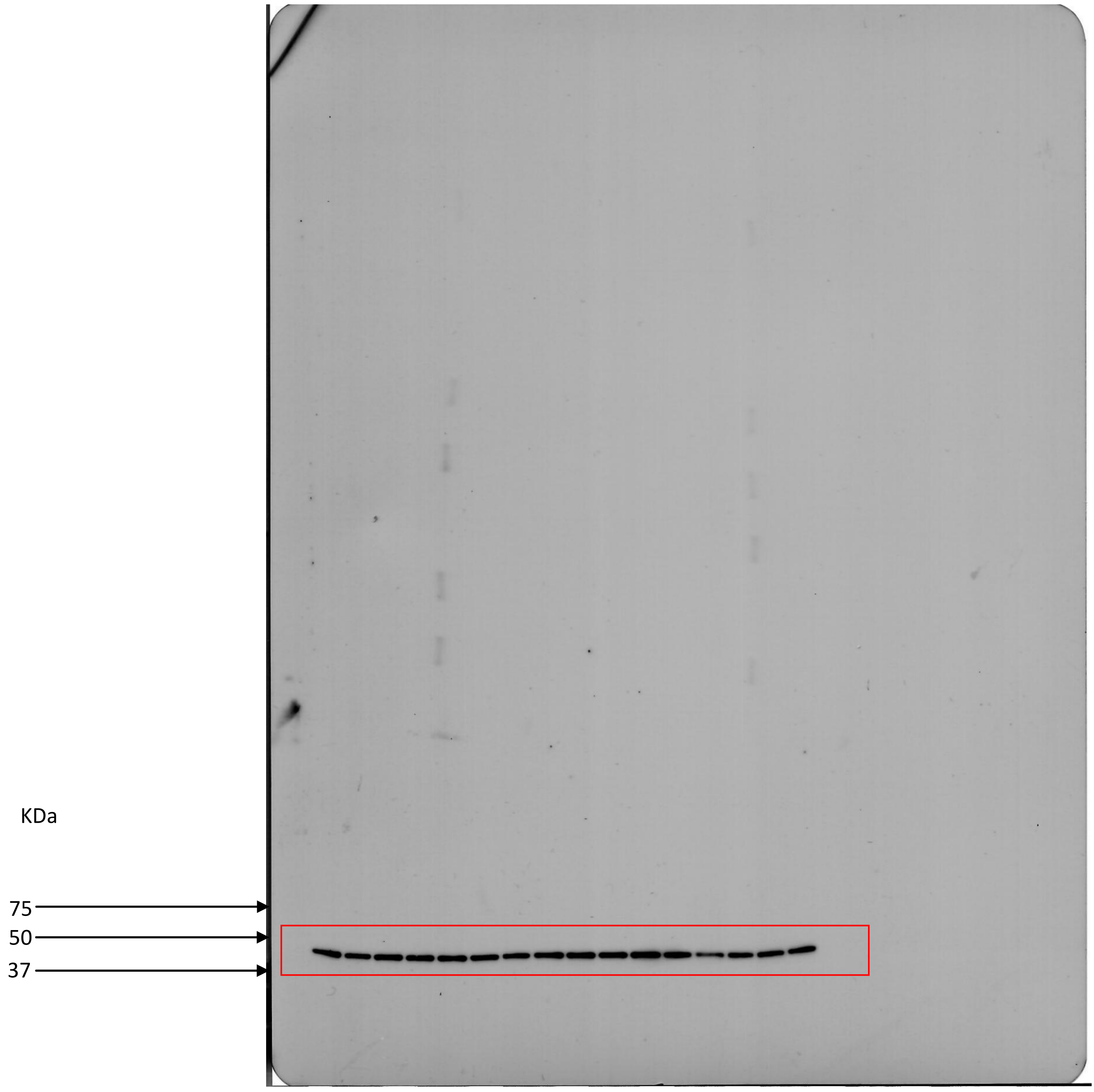

Supplementary Figure 5a

IB: SHP-1 (SHP-1 siRNA)

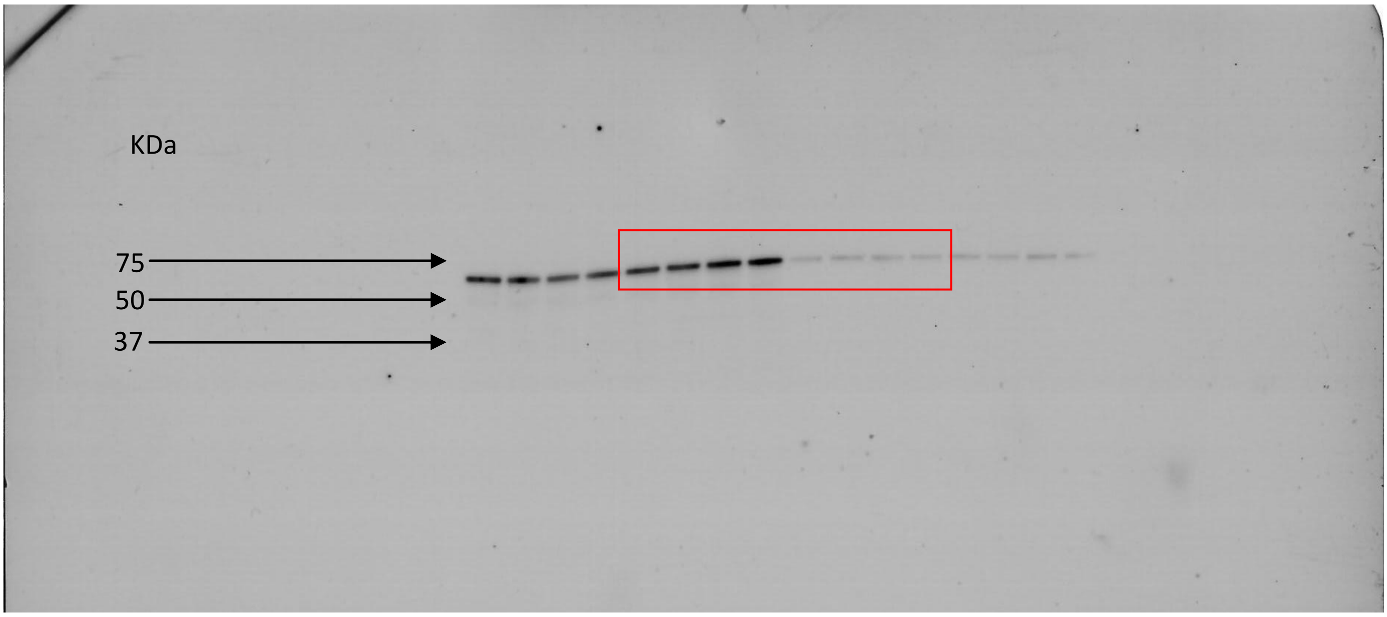

Supplementary Figure 5a

IB: actin (SHP-1 siRNA)

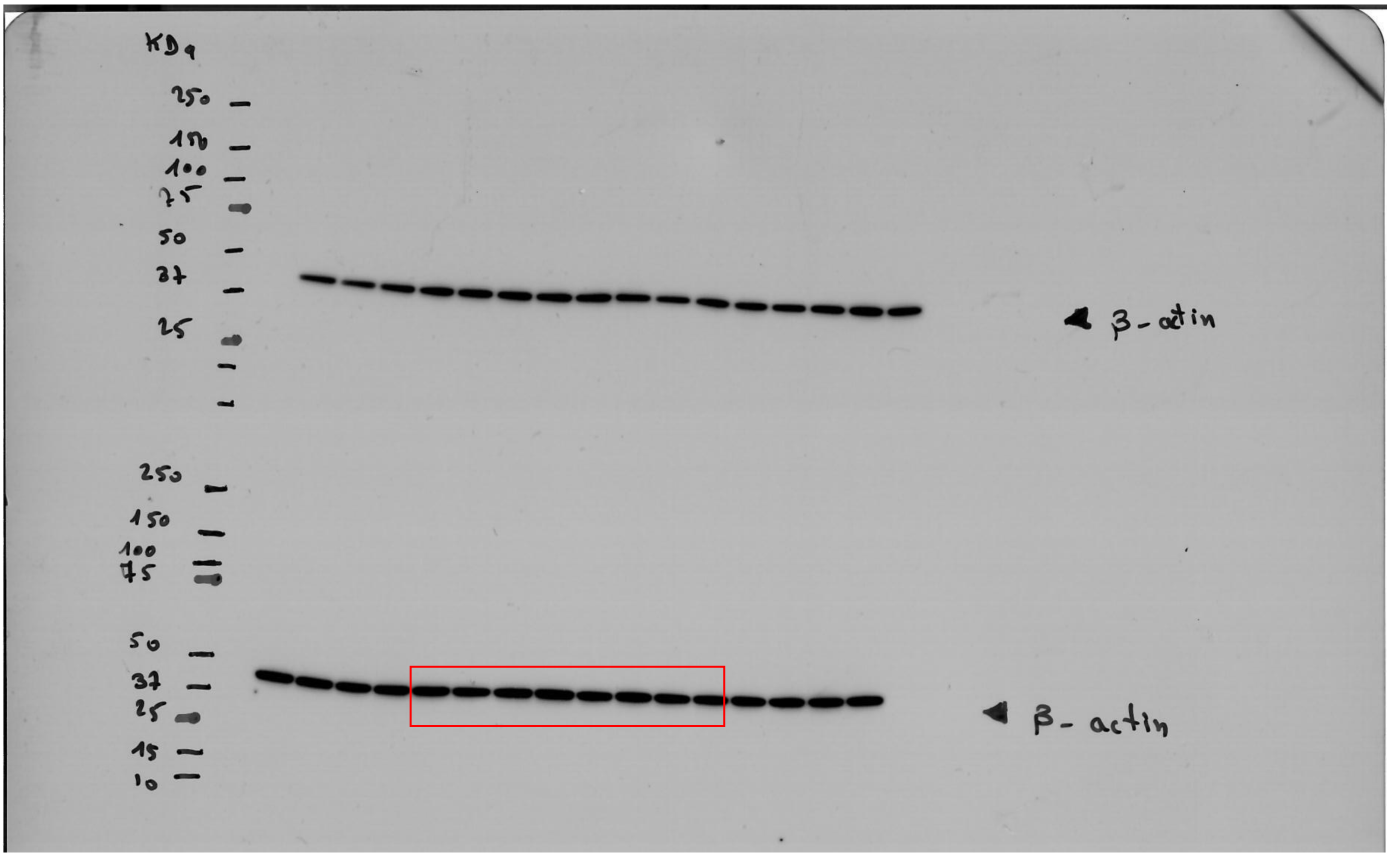

Supplementary Figure 5b

IB: SHP-1 (Vehicle)

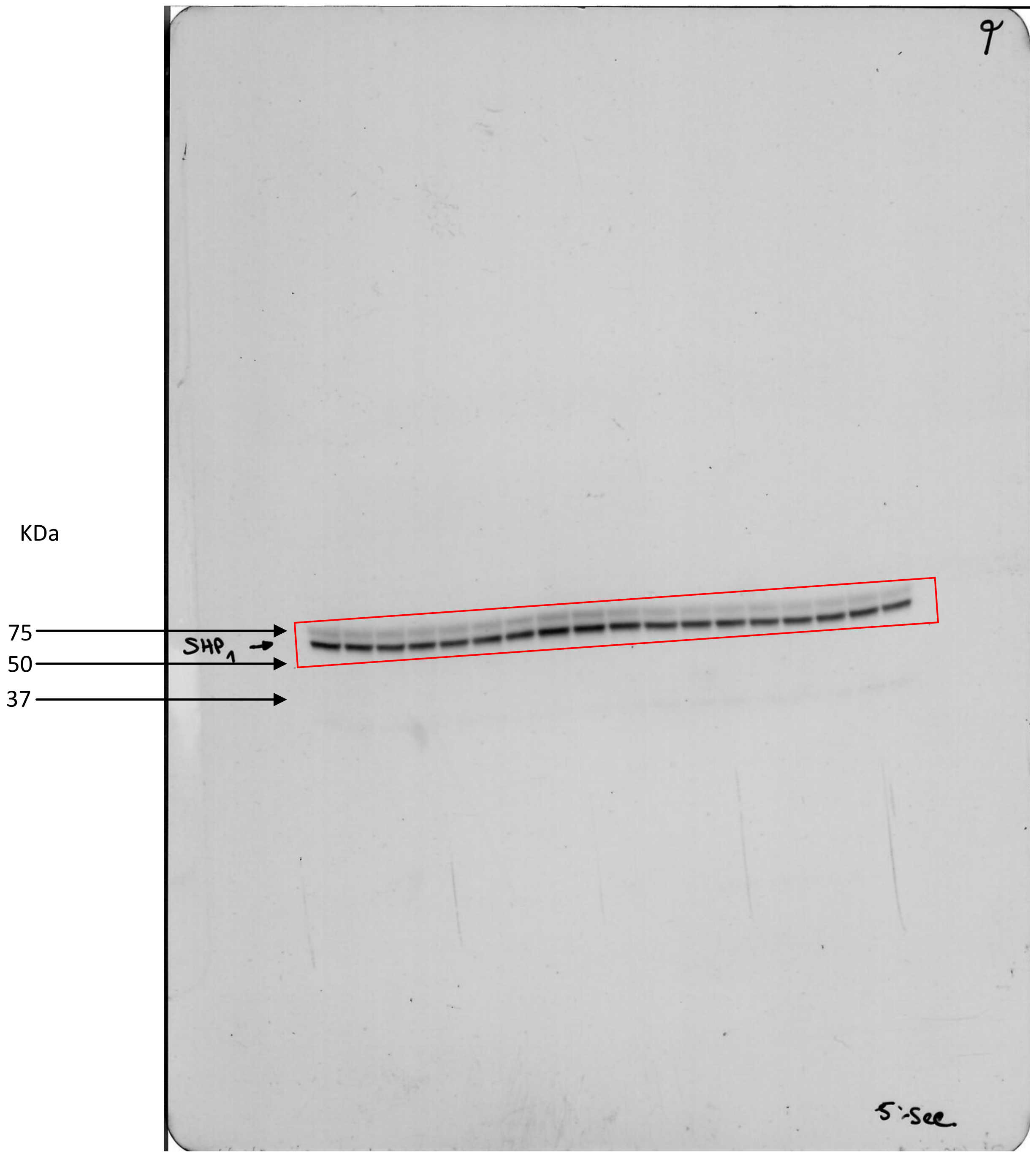

Supplementary Figure 5b

IB: SHP-1 (ERK inhibitor)

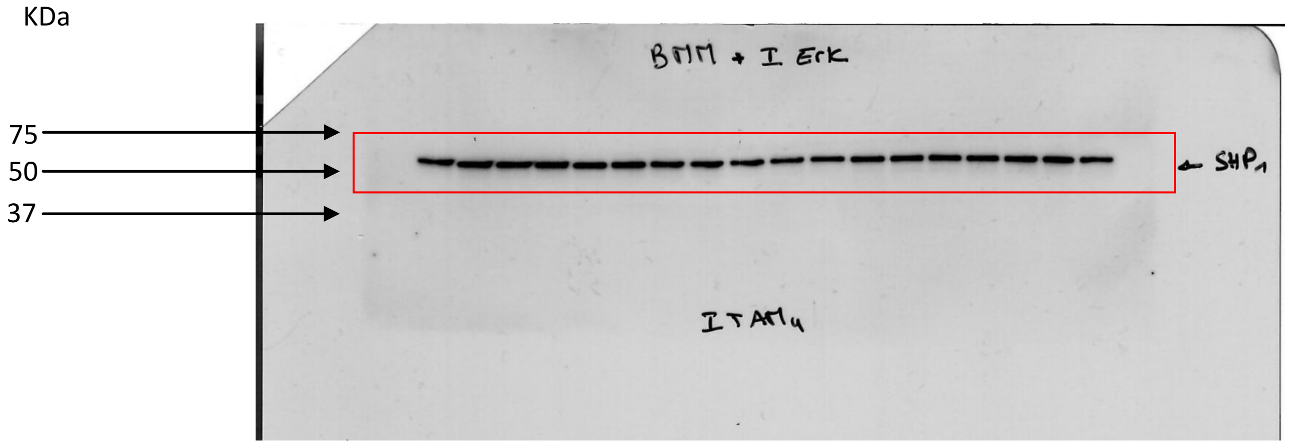

Supplementary Figure 5b

IB: SHP-1 (PI3K inhibitor)

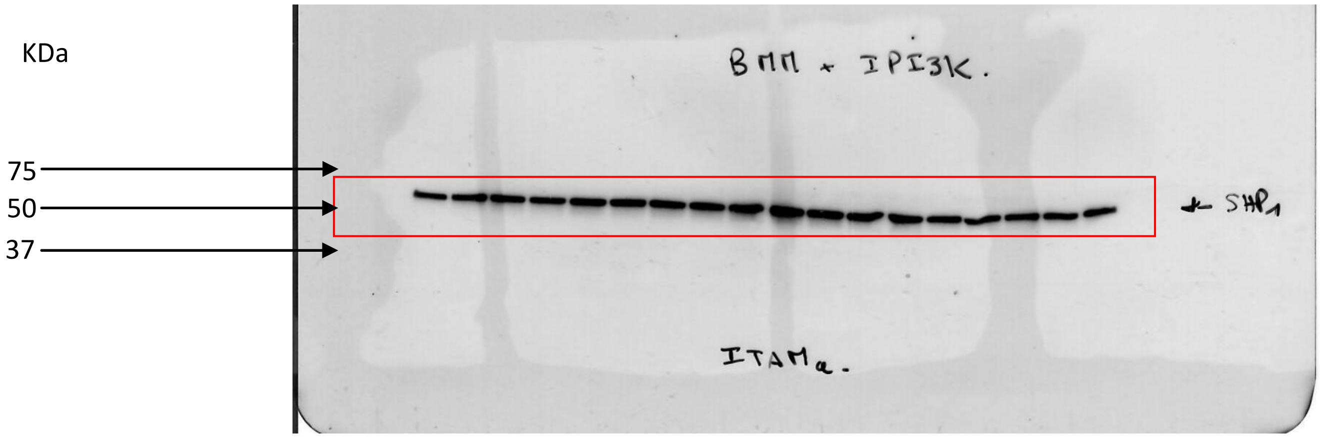

Supplementary Figure 5b

IB: PKC (PKC inhibitor)

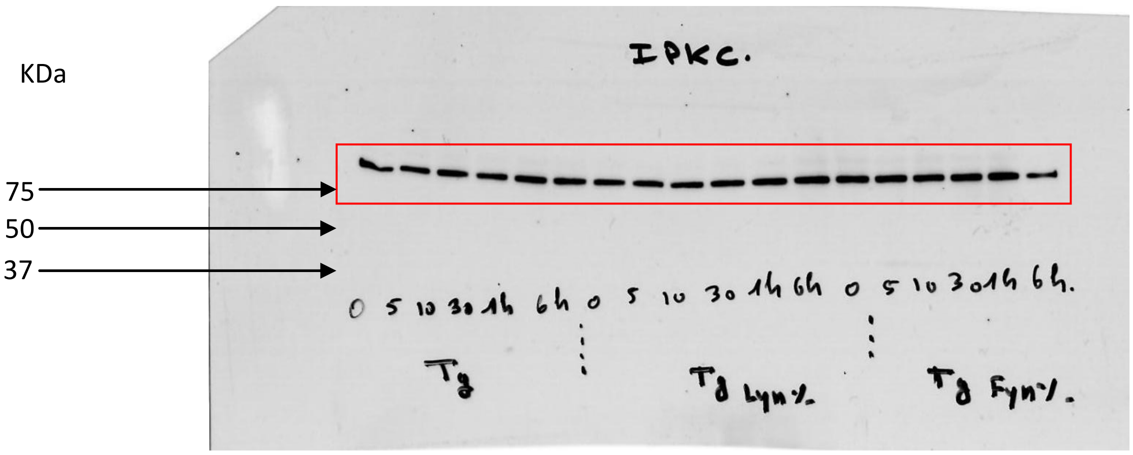

Supplementary Figure 5b

IB: AKT (PKC inhibitor)

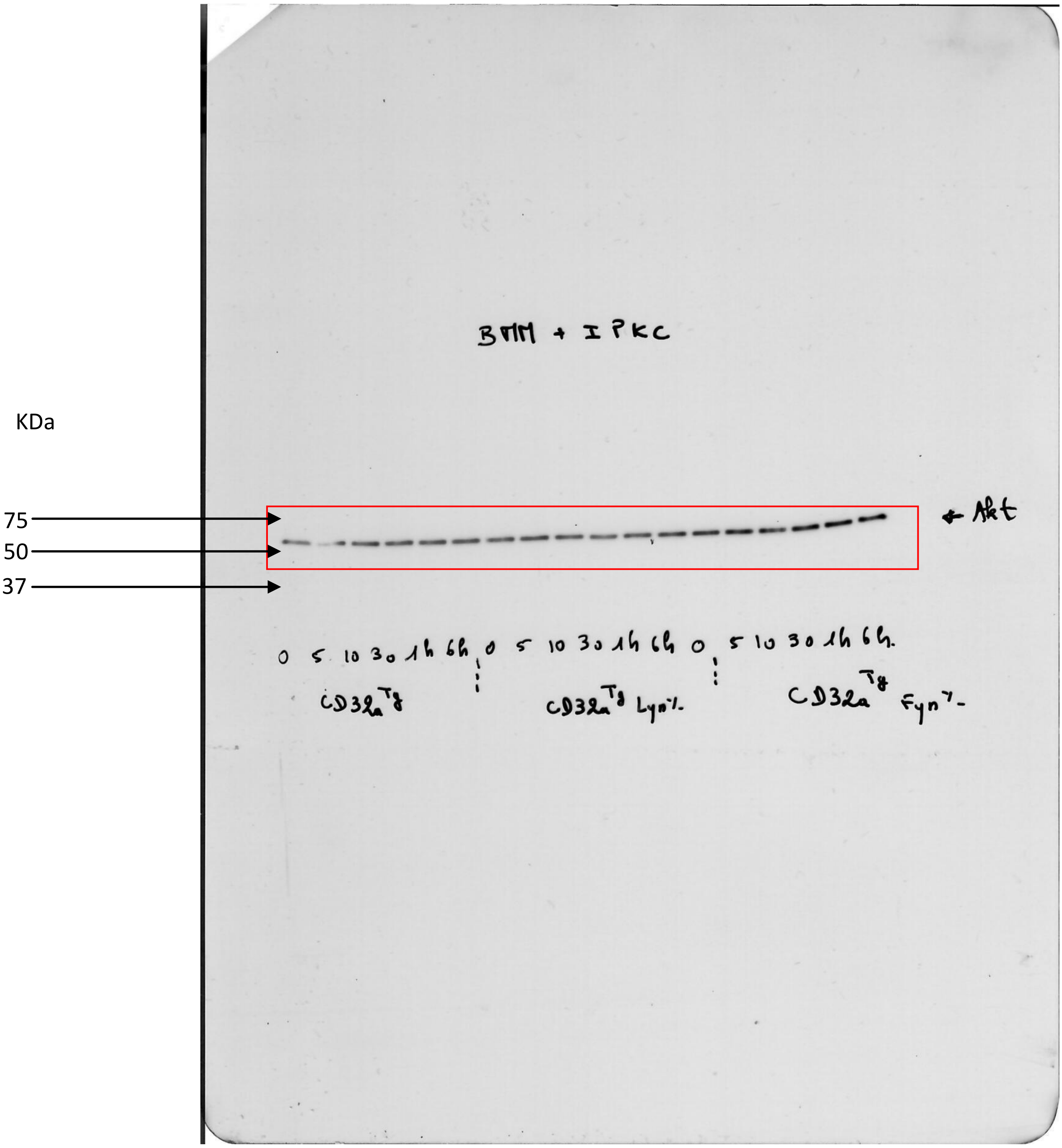

Supplementary Figure 5b

IB: AKT (PI3K inhibitor)

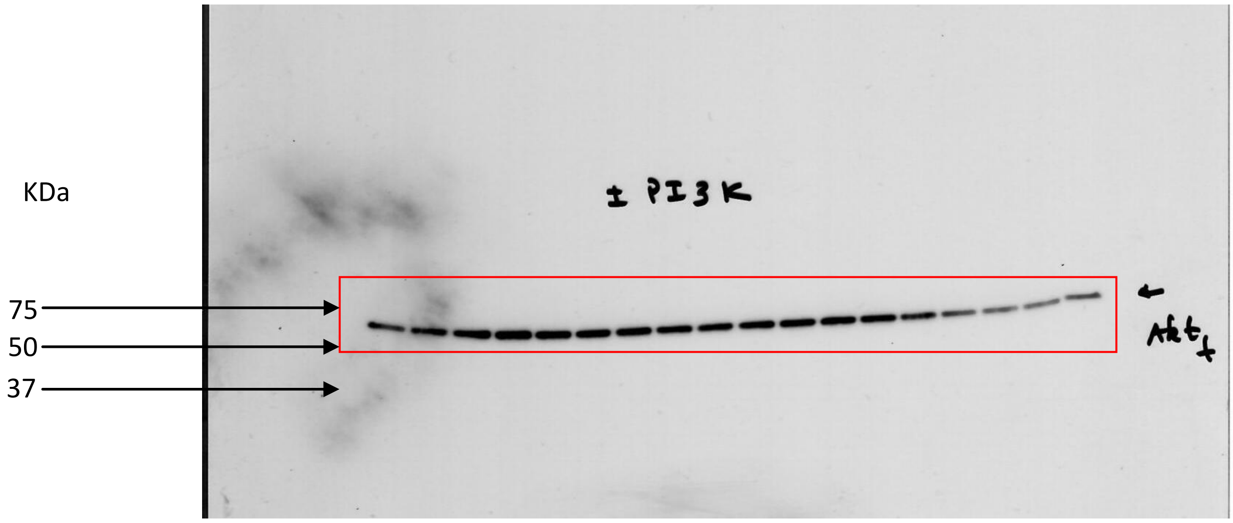

Supplementary Figure 5b

IB: PKC (PI3K inhibitor)

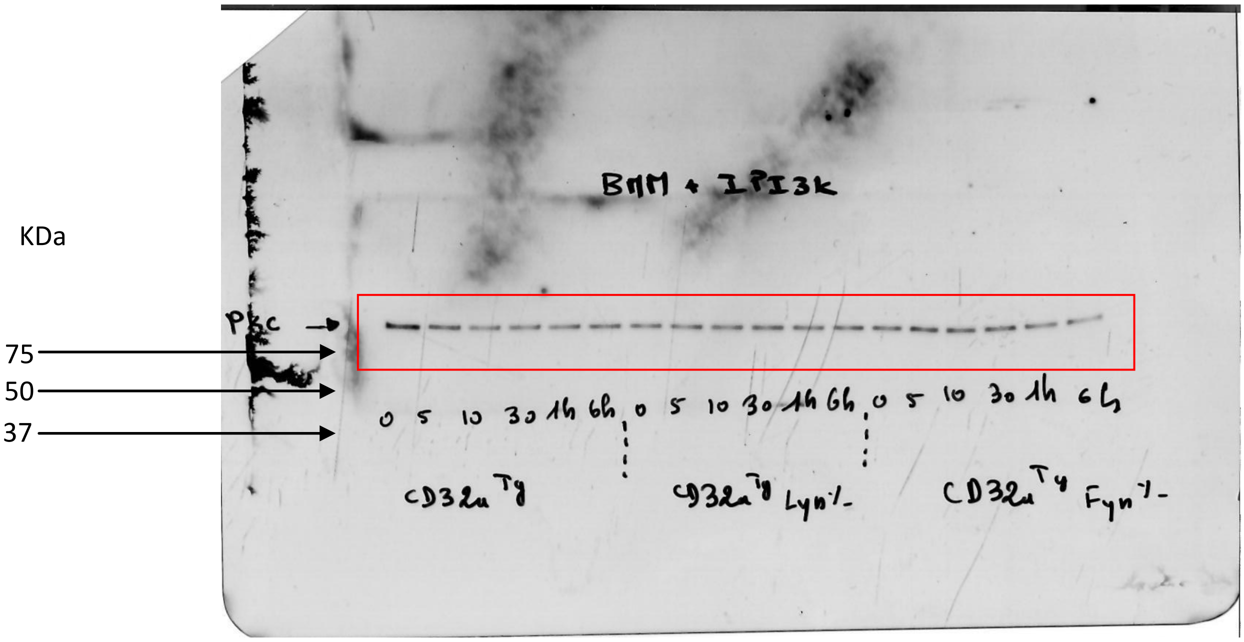

Supplementary Figure 5b

IB: ERK (Vehicle)

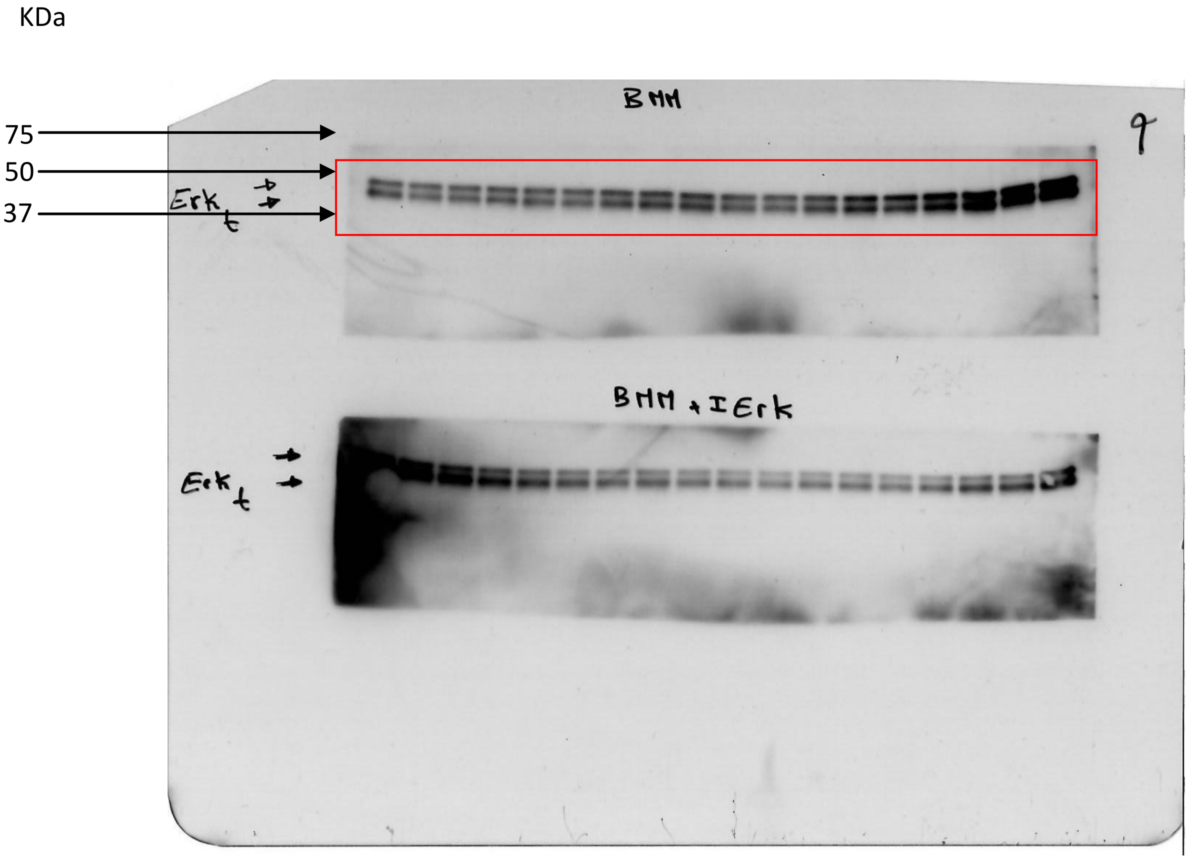

Supplementary Figure 5b

IB: ERK (ERK inhibitor)

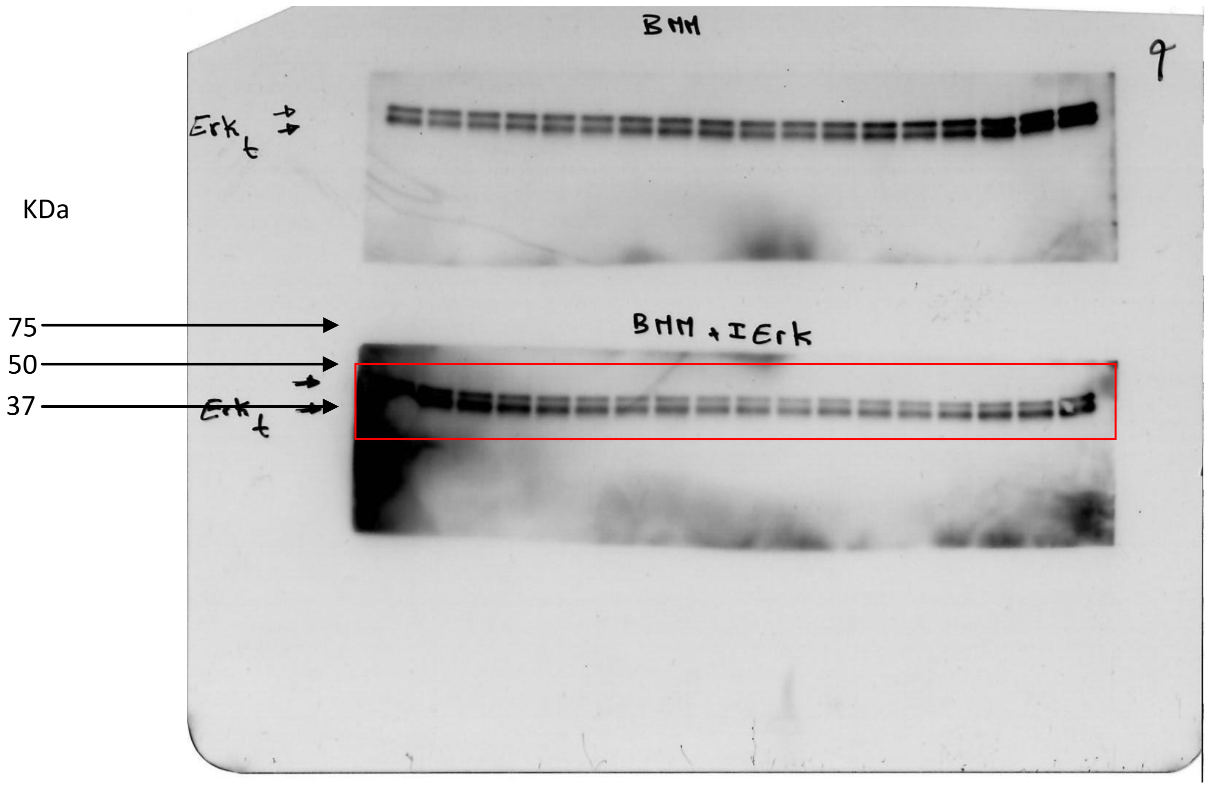

Supplementary Figure 5b

IB: PKC (Vehicle)

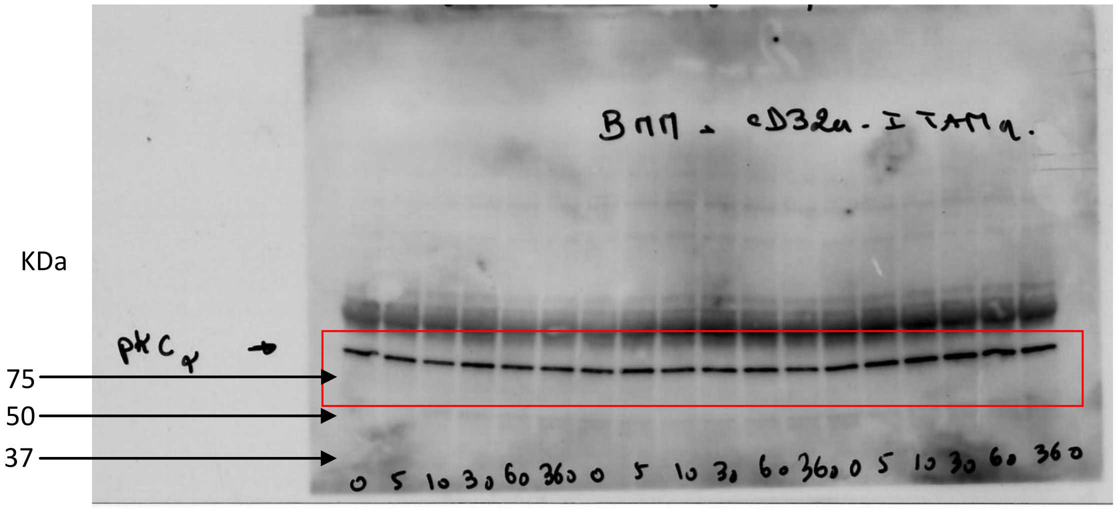

Supplementary Figure 5b

IB: AKT (Vehicle)

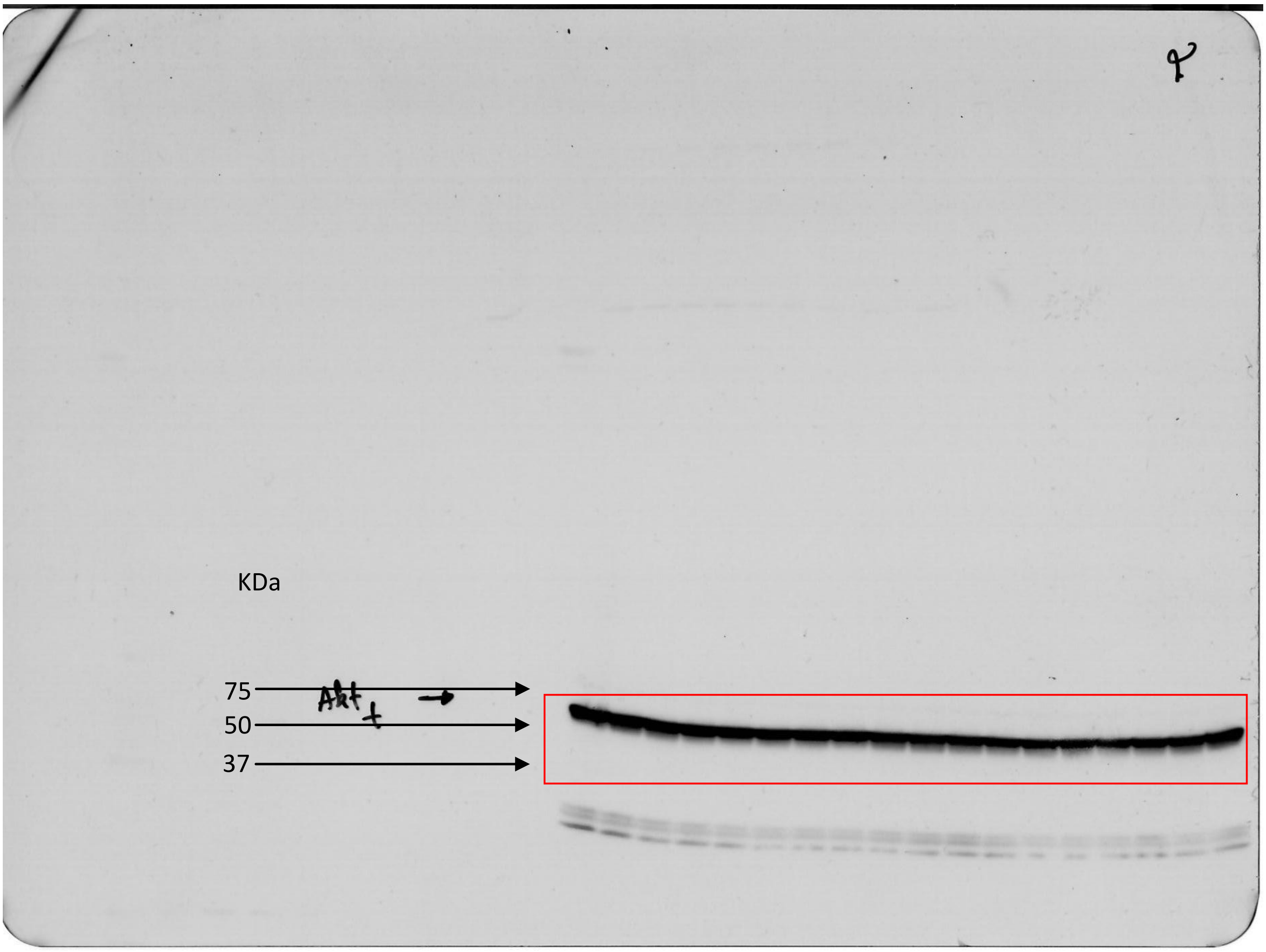

Supplementary Figure 5b

IB : ERK (PKC inhibitor)

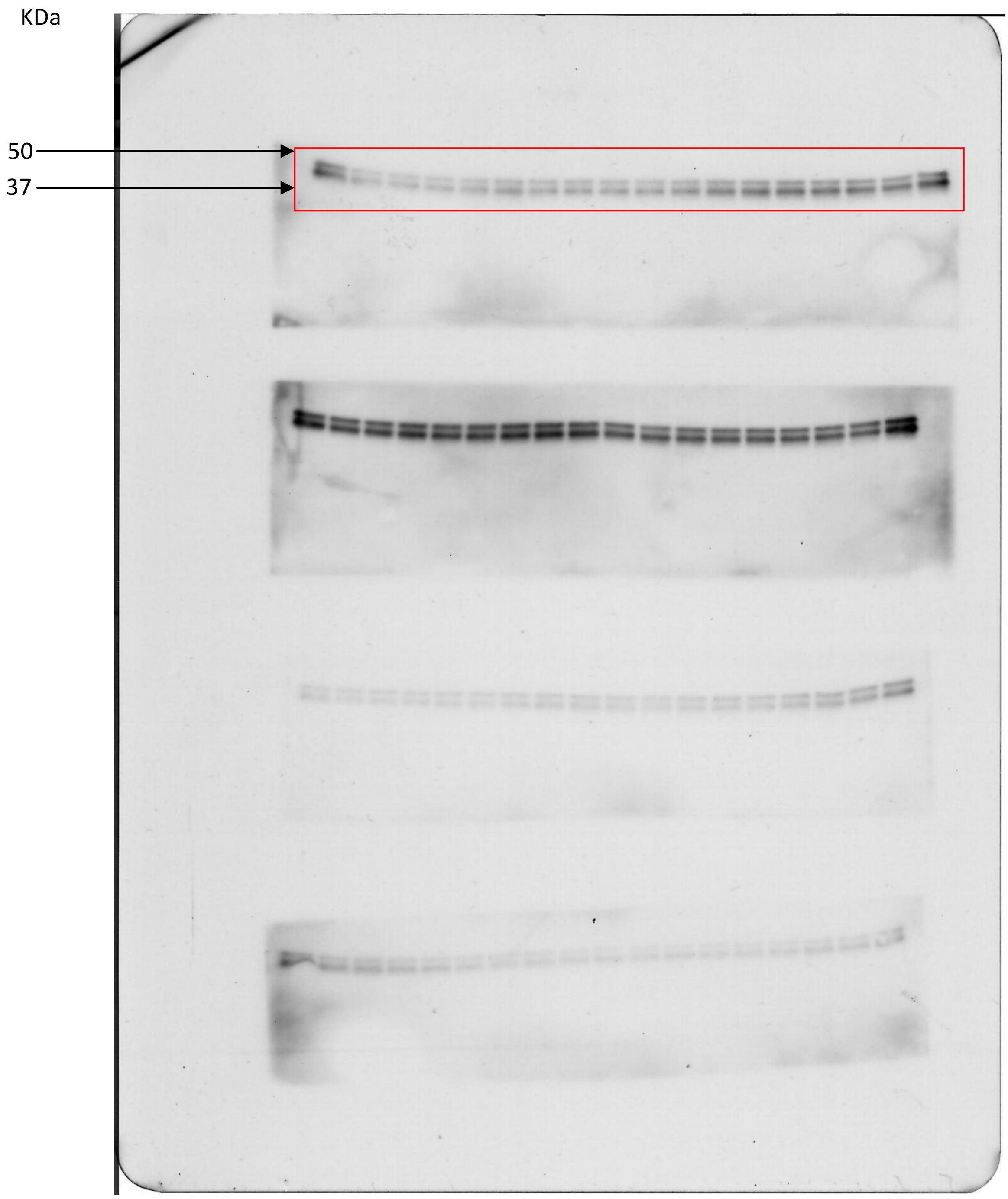

Supplementary Figure 5b

IB : ERK (PI3k inhibitor)

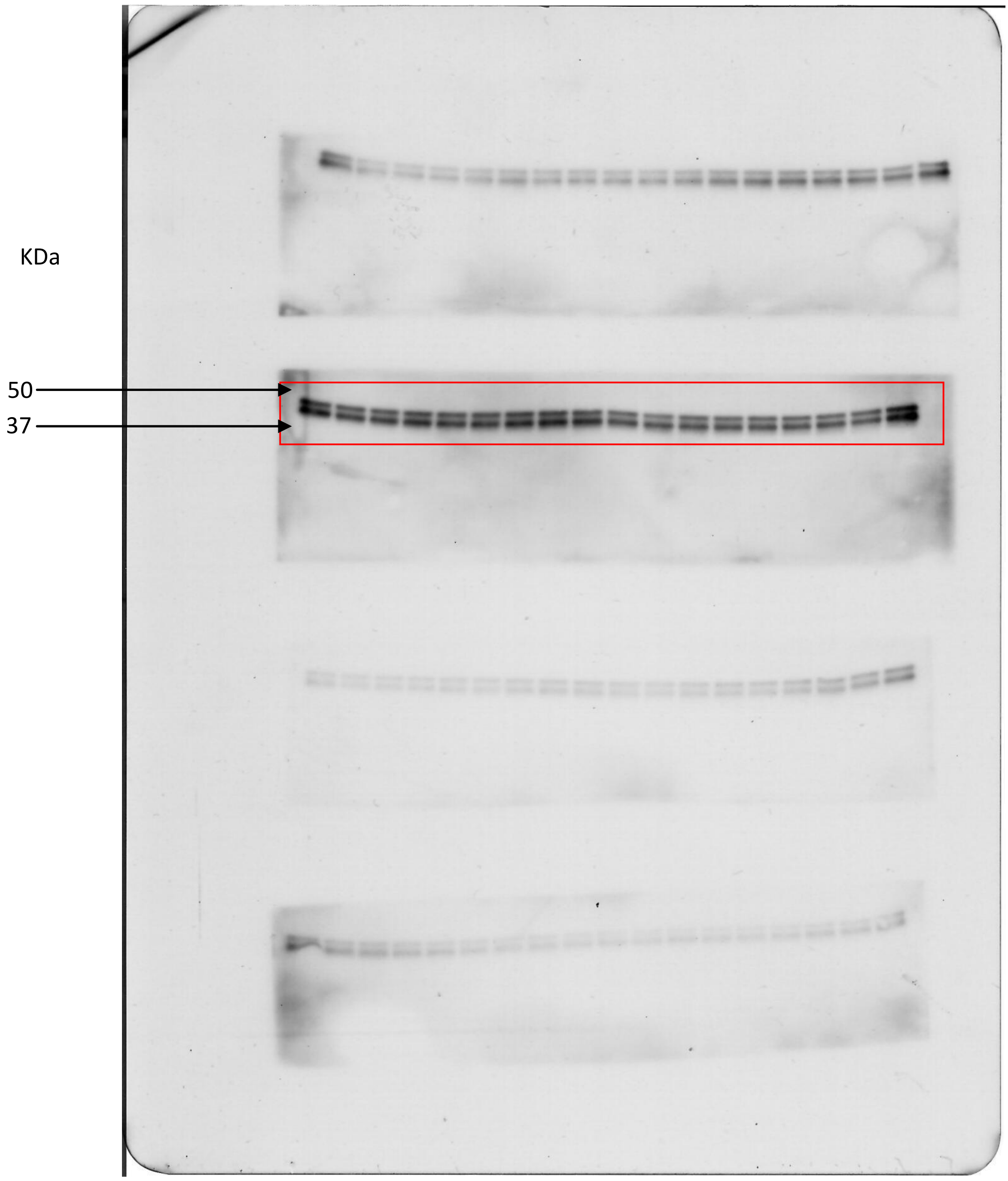

Supplementary Figure 5b

IB : PKC (ERK inhibitor)

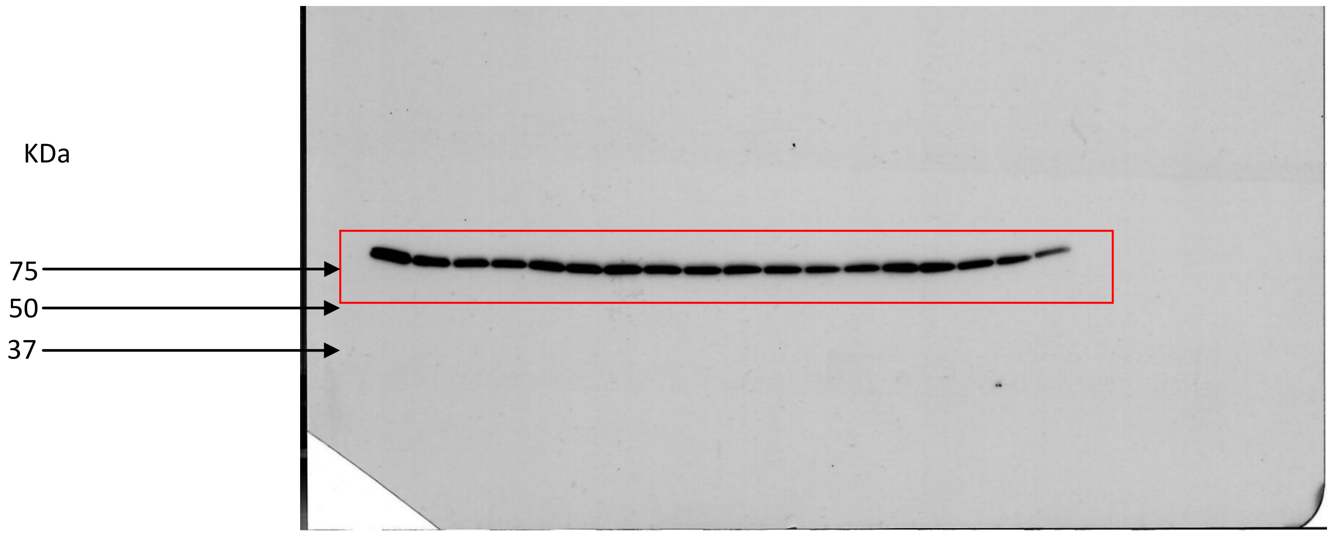

Supplementary Figure 5b

IB : AKT (ERK inhibitor)

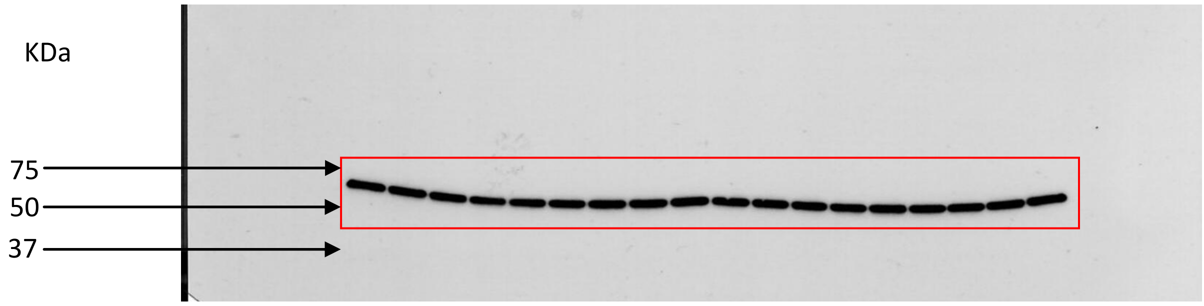

Supplementary Figure 5b

IB : SHP-1 (PKC inhibitor)

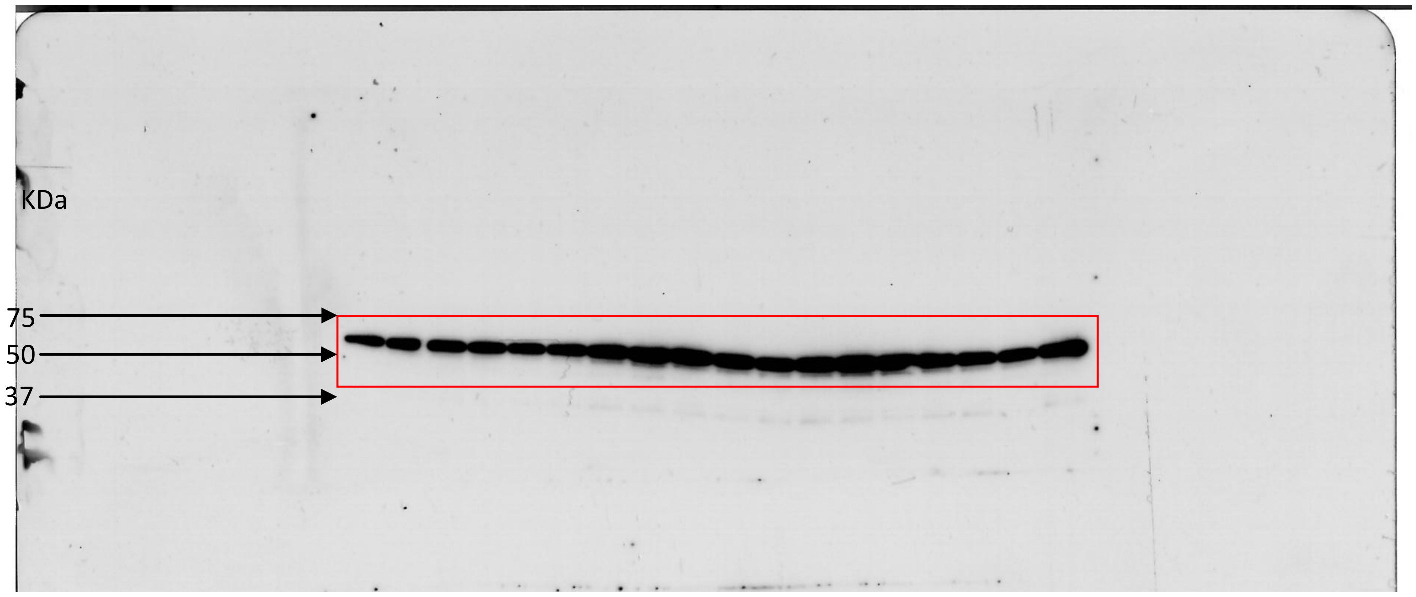

Supplementary References:

1. Pfirsch-Maisonnas, S., *et al.* Inhibitory ITAM signaling traps activating receptors with the phosphatase SHP-1 to form polarized "inhibisome" clusters. *Sci Signal* **4**, ra24 (2011).
